# Forensic Species Identification: A Case Involving Trafficked Fish Maws and Shark Fins

**DOI:** 10.3390/ijms27135813

**Published:** 2026-06-27

**Authors:** Hui Li, Xufeng Chu, Weiheng Xiao, Jinxu Jiang, Ruocheng Xia, Man Chen, Ya Di, Yukun Liu, Xiling Liu

**Affiliations:** 1 Shanghai Key Laboratory of Forensic Medicine, Shanghai Forensic Service Platform, Key Laboratory of Forensic Science, Ministry of Justice, Academy of Forensic Sciences, Shanghai 200063, China; lihui@ssfjd.cn (H.L.); chuxf@ssfjd.cn (X.C.);; 2 School of Forensic Medicine, Kunming Medical University, Kunming 650500, China; 3 KLATASDS—MOE, School of Statistics, East China Normal University, Shanghai 200062, China

**Keywords:** mass wildlife trafficking, forensic species identification, DNA barcoding, high-volume sample, CITES

## Abstract

Illegal wildlife trade and other forms of wildlife crime pose significant threats to global biodiversity. Accurate species identification of wildlife products is crucial for effectively combating illegal wildlife trade. In recent years, forensic species identification using molecular methods has gained increasing attention. Here, we report a case involving 1777 trafficked fish maws and 18,170 trafficked shark fins. We employed DNA barcoding for forensic species identification and a large-sample inference strategy for population estimation. A total of 325 samples (197 fish maws and 128 shark fins) were randomly selected for DNA testing based on their pre-classified morphological categories. Two DNA barcodes (COI and 16S) were used, and sequence alignment was conducted using the NCBI GenBank and BOLD systems. Phylogenetic analyses were performed based on sequences detected from the samples and reference sequences, with results consistent with the sequence alignment. Consequently, seven species were identified, four of which, corresponding to approximately 17,351 to 18,170 specimens, are listed in Appendix II of the Convention on International Trade in Endangered Species of Wild Fauna and Flora (CITES).

## 1. Introduction

Illegal wildlife trade represents one of the most pressing threats to global biodiversity, contributing significantly to the decline of many species worldwide [1,2,3]. To support the protection of threatened species and ensure that international trade in wildlife does not threaten their survival, the Convention on International Trade in Endangered Species of Wild Fauna and Flora (CITES) was established in 1973 and entered into force in 1975 [4]. To date, CITES includes 185 contracting countries [5]. CITES categorizes endangered wildlife into three appendices (I, II, and III) based on their levels of endangerment. Currently, wildlife crime constitutes a highly lucrative global illegal industry, with an estimated annual value reaching billions of dollars. However, due to the covert and illicit nature of these activities, determining the precise value remains a significant challenge [6,7,8]. Moreover, the illegal wildlife trade poses a severe threat to ecological balance and biodiversity. Consequently, rapid and accurate species identification is critical for guiding conservation efforts and enforcing legal frameworks aimed at combating the illicit exploitation of wildlife resources [9].

In recent years, the forensic community has increasingly focused on nonhuman biological materials, as molecular methods have been demonstrated to be reliable and effective for forensic species identification, with DNA barcoding serving as one of the most commonly used techniques [10,11,12]. DNA barcoding employs short, standardized gene regions for species identification. For animals, mitochondrial markers are predominant due to their high copy number and rapid evolution. COI (Cytochrome c Oxidase I) is widely regarded as the gold standard for DNA barcoding, supported by a comprehensive and globally accessible reference database. Its primary limitations involve potential misidentification due to reduced efficacy in distinguishing closely related or recently diverged species caused by insufficient genetic divergence. Cytochrome b (Cytb) is a well-established marker with good discriminatory power, but its lack of a standardized region limits direct comparability. Ribosomal RNA genes, 16S and 12S, are highly conserved, allowing for the design of universal primers that work across broad taxonomic groups, making them ideal for diverse samples. Current research efforts tend to combine multiple DNA barcodes to overcome the limitations of any single marker. DNA barcoding is playing an increasingly crucial role in forensic investigations [13]. Utilizing standardized genetic markers, it provides rapid and accurate species identification from degraded or fragmentary evidence where traditional morphology proves inadequate [14]. This capability significantly enhances the robustness of evidentiary chains in court proceedings. Consequently, an increasing number of cases have been reported in which DNA barcoding has been employed as a tool to facilitate the prosecution of wildlife trafficking, particularly for CITES-listed species.

In this forensic case, the coast guard seized an illicit shipment of trafficked wildlife derivatives, including suspected protected species products (fish maws and shark fins). To provide the definitive, scientific evidence required for legal prosecution, our laboratory was judicially commissioned to conduct species identification with legal implications under CITES. The primary objective was to determine whether the confiscated biological tissues originated from species listed under CITES Appendix, thereby providing crucial evidence to support prosecutorial decisions in cases of wildlife trafficking. The number of specimens belonging to CITES-listed species involved in the case directly influences the sentencing and the severity of the punishment. Compared to other wildlife forensics studies, the total number of seized specimens involved in this case reached 19,947, which far exceeds the routine processing capacity of most forensic laboratories. Given the strict time constraints imposed by law enforcement operations, conducting DNA testing on every single specimen was neither practical nor necessary. Therefore, a Bayesian statistical estimation framework was introduced in this case to infer population composition from a subset of samples. In addition, two major general species databases, NCBI GenBank and the BOLD system, were both utilized for species identification.

## 2. Results

### 2.1. Sample Collection and DNA Barcoding

**Figure 1:**
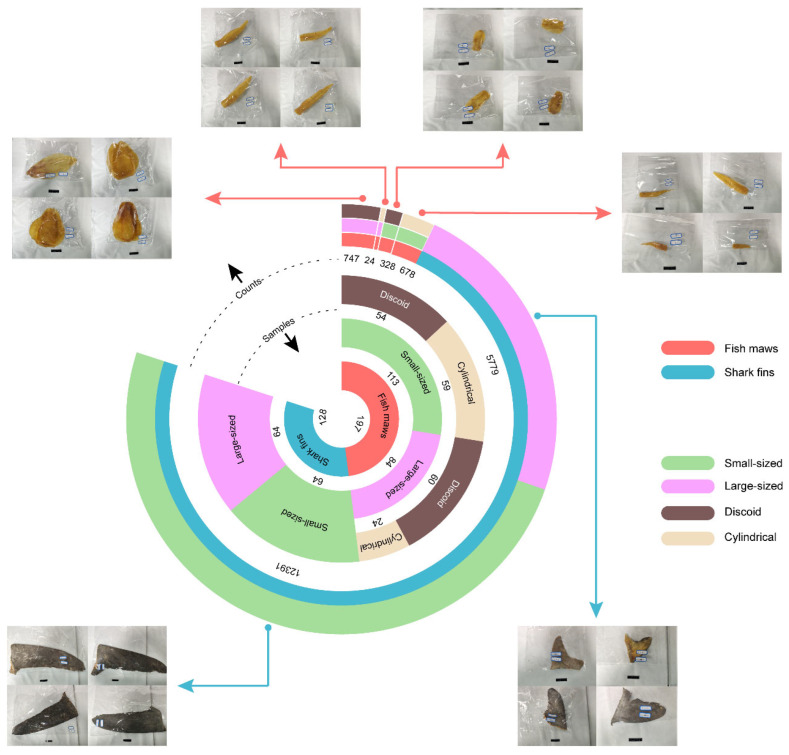
Distribution of sample counts by morphological category (Scale bars: 5 cm).

In this suspected case involving illegal wildlife products, a total of 19,947 specimens were seized. Given the substantial quantity, a systematic random sampling strategy was developed. All seized fish maws and shark fins were categorized and counted based on their sizes and morphological characteristics, and the sample size was determined based on the pre-classified population size. Following this strategy, a total of 325 samples, including 197 fish maws and 128 shark fins, were randomly selected for testing (Figure 1). The images of the selected fish maws and shark fins are presented in Supplementary Figures S1 and S2, respectively.

Two DNA barcodes, COI and 16S, were employed for species identification. The COI barcode was selected as the primary marker due to its high resolution, and the 16S barcode was used as a supplementary marker to resolve ambiguous identifications when required. The classical Sanger sequencing method was employed to ensure reliability. Among the 325 randomly selected samples, adequate DNA could not be extracted from two samples owing to severe degradation. The remaining 323 samples were successfully amplified and sequenced in the COI barcode fragment, and 158 of these samples were further analyzed in the 16S barcode fragment, including all 128 shark fin samples and 30 fish maw samples, as presented in Table 1. The mean sequence length for COI was 559.9 ± 18.3 bp (range 504–652 bp), and for 16S was 531.5 ± 8.2 bp (range 510–554 bp). All accepted sequences had Phred scores >25 at >90% of base positions, with no ambiguous bases in the consensus region.

### 2.2. Species Identification by Sequence Alignment

Comparing the COI sequence alignments across different databases, the species identification results for all samples obtained using Web BLAST (https://blast.ncbi.nlm.nih.gov/) on GenBank were consistent with those obtained using the IDENTIFICATION tool on the BOLD system (version 5). After the COI sequence alignment, further 16S sequence alignment was conducted for samples whose species remained undetermined and those suspected to be potential endangered species. Since the BOLD system only includes COI barcodes, supplementary 16S sequence alignment was conducted using BLAST against GenBank.

A total of 30 fish maw samples exhibited high genetic similarity to two species, *Pseudotolithus typus* and *Pseudotolithus elongatus*, in the COI sequence. After conducting further 16S sequence alignment, all these samples were conclusively identified as *Pseudotolithus typus*. It should be noted that 103 samples failed to match any species in GenBank (the identity was all below 90%). In contrast, these samples showed a high degree of identity (ranging from 99.82% to 100%) with the *Galeoides decadactylus* in the BOLD system. The remaining 62 fish maw samples were identified as *Pseudotolithus brachygnathus*. Among the 128 randomly selected shark fin samples, four shark species were identified based on the COI barcode: *Alopias pelagicus* (38 specimens), *Alopias superciliosus* (31 specimens), *Carcharhinus falciformis* (48 specimens), and *Isurus oxyrinchus* (11 specimens). All of these species are listed in Appendix II of the CITES. This result was further confirmed with 16S sequence alignment. The species distribution of samples from pre-classified morphological categories is summarized in Table 2.

### 2.3. Phylogenetic Analysis

**Figure 2:**
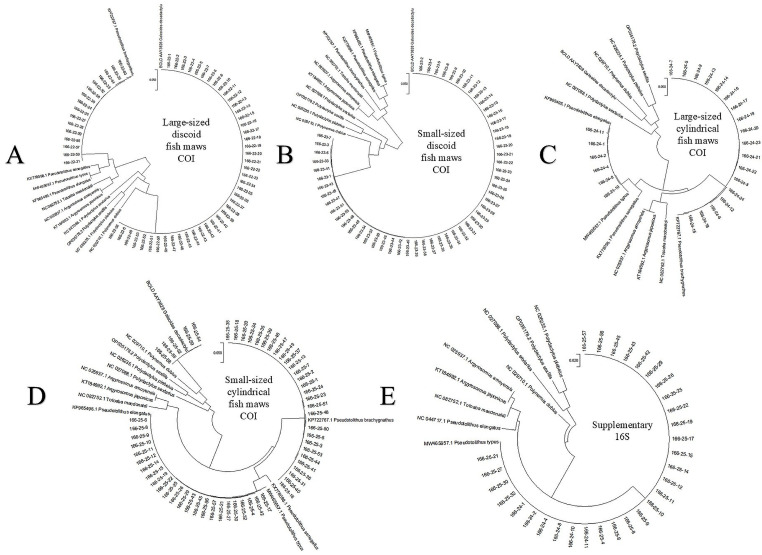
The phylogenetic trees constructed from fish maw sequences across four sample categories. (**A**) COI gene—Large-sized discoid samples (*n* = 60); (**B**) COI gene—Small-sized discoid samples (*n* = 54); (**C**) COI gene—Large-sized cylindrical samples (*n* = 24); (**D**) COI gene—Small-sized cylindrical samples (*n* = 57); (**E**) 16S rRNA gene—Supplementary samples (*n* = 30).

**Figure 3:**
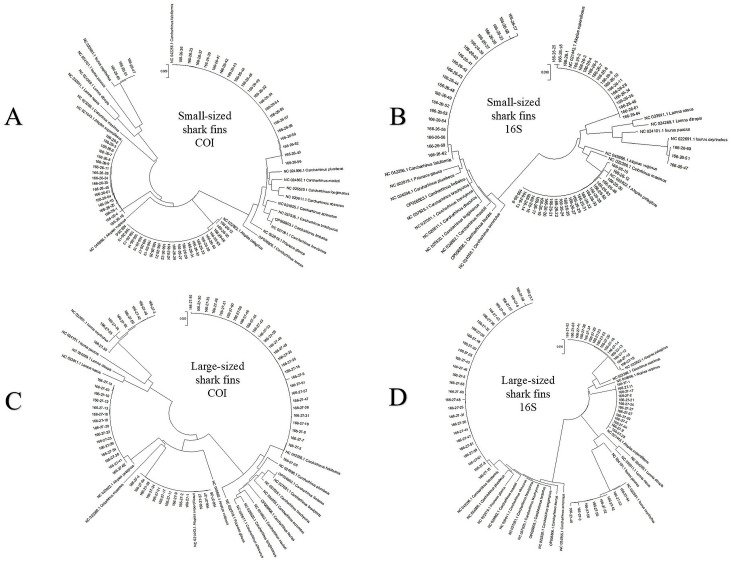
The phylogenetic trees constructed from shark fin sequences across two sample categories. (**A**) COI gene—Small-sized samples (*n* = 64); (**B**) 16S rRNA gene—Small-sized samples (*n* = 64); (**C**) COI gene—Large-sized samples (*n* = 64); (**D**) 16S rRNA gene—Large-sized samples (*n* = 64).

Following the sequence alignment and species identification, phylogenetic analyses were performed based on the sequences detected from samples and reference sequences. Phylogenetic trees were constructed separately according to pre-classified morphological categories. Reference sequences were downloaded from GenBank, except for that of *Galeoides decadactylus*, which was downloaded from the BOLD system. As illustrated in Figure 2 and Figure 3, each sample was found to cluster closely with the corresponding reference of the identified species and was located on its corresponding clade, following the phylogenetic classification of species. The phylogenetic tree results were concordant with the sequence alignment results, confirming that species identification for samples was reliable.

### 2.4. Statistical Estimation of Endangered Species Proportion

Analysis of the 323 randomly selected samples (excluding two degraded specimens) identified seven distinct species. All sampled shark fin specimens in both “Large-sized” and “Small-sized” categories were identified as originating from species listed in Appendix II of CITES, with an estimated proportion of 100%. However, since 100% corresponds to a degenerate proportion, traditional frequentist uncertainty analysis may no longer be applicable. To address this, a Bayesian statistical framework was employed, modeling the proportion of endangered species within each category as a random variable. Given the absence of prior information, an uninformative prior was adopted, specifically a standard uniform distribution (Beta(1,1)). Based on the 64-sample dataset, the posterior distribution for p is derived as Beta(65,1). The resulting 95% Bayesian credible interval for p is [0.955, 1]. Consequently, the 95% confidence intervals for the estimated counts of CITES Appendix II-listed specimens are as follows: Large-sized category: [5518, 5779], and Small-sized category: [11,833, 12,391]. In total, approximately 17,351 to 18,170 specimens are inferred to have originated from species listed in CITES Appendix II, with a 95% confidence interval.

## 3. Discussion

Traditionally, species identification in wildlife forensic science has predominantly depended on morphological characteristics. Nevertheless, morphological analysis is inherently subjective, requiring specialized expertise, and is often limited in its applicability, particularly when confronted with fragmented or processed samples. Molecular methods have proven to be reliable and effective for species identification, leading the forensic community to increasingly focus on nonhuman biological materials in recent years [11]. DNA barcoding is a widely adopted molecular technique for species identification and is considered to have significant potential applications in forensic wildlife crime investigations [14].

In this case, although several novel technologies, such as massively parallel sequencing (MPS) [15,16] and nanopore sequencing [17,18], have been reported to be applicable for DNA barcoding species identification, the classical Sanger sequencing method was still employed to ensure reliability. The current gold standard approach for forensic molecular species identification involves DNA extraction and quantification, PCR amplification, and Sanger sequencing of highly interspecies-variable regions using conserved primers, followed by sequence alignment and comparison with sequences available in the reference databases [11,19].

Two databases were employed for species identification in this case. NCBI GenBank is a comprehensive and versatile repository for nucleotide sequences, providing access to genomic and transcriptomic data across all domains of life [20]. In contrast, the BOLD system serves as a specialized platform exclusively dedicated to standardized DNA barcodes for species identification and discovery [21]. These two databases are capable of complementing each other in terms of functionality and data coverage. For animal species identification, the BOLD system only provides COI barcodes, which enable rapid screening but may also introduce limitations due to reliance on a single genetic marker. While GenBank provides more extensive DNA barcodes in mtDNA, enabling the use of supplementary markers to resolve taxa when COI is insufficient and supporting multi-locus verification. In this case, *Pseudotolithus typus* and *Pseudotolithus elongatus* could not be distinguished using COI alone. Therefore, 16S was employed to complement the identification process. Furthermore, these two DNA barcodes (COI and 16S) were employed to cross-validate and ensure the accuracy of the identification results for four endangered shark species. However, the BOLD system may contain some species that are not recorded in GenBank, as demonstrated by the successful identification of *Galeoides decadactylus*. Another important point to emphasize is that the BOLD system enforces the linkage of sequences to specimen information, including georeferenced collection localities, specimen images, and taxonomic verifications. This not only ensures traceability but also enhances reliability by establishing a robust framework for data verification and validation. Nevertheless, GenBank and BOLD do not provide comprehensive coverage for all species, particularly those that are taxonomically underrepresented. This limitation may undermine the accuracy of species identification, especially in cases where novel or rare species are involved. In the present case, the reliability of the identification was supported by two independent DNA barcodes showing nearly 100% sequence identity. Due to the low-throughput limitation of Sanger sequencing, no additional DNA barcodes were sequenced. Furthermore, phylogenetic analysis was performed not only to confirm species identification but also to detect possible misidentifications due to database errors or incomplete reference sequences. Sequence matching alone can be affected by non-specific hits or incomplete databases. Phylogenetic trees provide a visual confirmation that each sample clusters with its expected reference sequence in a monophyletic clade, supporting the reliability of the identification. In this case, all 323 samples clustered with their respective reference sequences, indicating robust support.

Although DNA barcoding provides accuracy in species identification, its practical implementation in routine customs operations encounters substantial challenges. The most pressing challenge is the delay in obtaining results, particularly when sample sizes are large. Customs authorities often operate under strict time constraints and cannot detain shipments indefinitely awaiting laboratory analysis. To overcome this barrier, it is essential to establish an appropriate approach that identifies robust evidence from a subset of seizures, effectively balancing enforcement efficiency with judicial rigor to ensure the process is both feasible and effective. In this case, a sample of 325 specimens was used to represent a population of nearly 20,000 specimens. The margin of error d = 0.1225 (12.25%) was selected as a pragmatic trade-off between statistical precision and laboratory feasibility given the large seizure size. This margin ensures that the estimated proportion p will be within ±12.25% of the true proportion with 95% confidence. For categories with small population sizes (e.g., large-sized cylindrical fish maws, *n* = 24), a full census was conducted instead of sampling, eliminating sampling error for that category. As indicated in Table 2, the analysis identified seven distinct species. All sampled shark fin specimens in both “Large-sized” and “Small-sized” categories were identified as originating from species listed in Appendix II of CITES. Based on the estimation strategy, approximately 17,351 to 18,170 of all the seized trafficked biological materials in the case are likely to have originated from species listed in Appendix II of CITES. A potential limitation of this study is the reliance on preliminary morphological classification (size and shape) to define strata for random sampling. While this stratification improves efficiency by reducing variance, it may introduce bias if the morphological categories do not perfectly correlate with species identity. For example, some small-sized cylindrical fish maws might belong to a different species than large-sized discoid ones. Future studies could use a fully random sample without stratification to verify representativeness, but given the logistical constraints of a large seizure, the stratified approach was the most practical.

**Figure 4:**
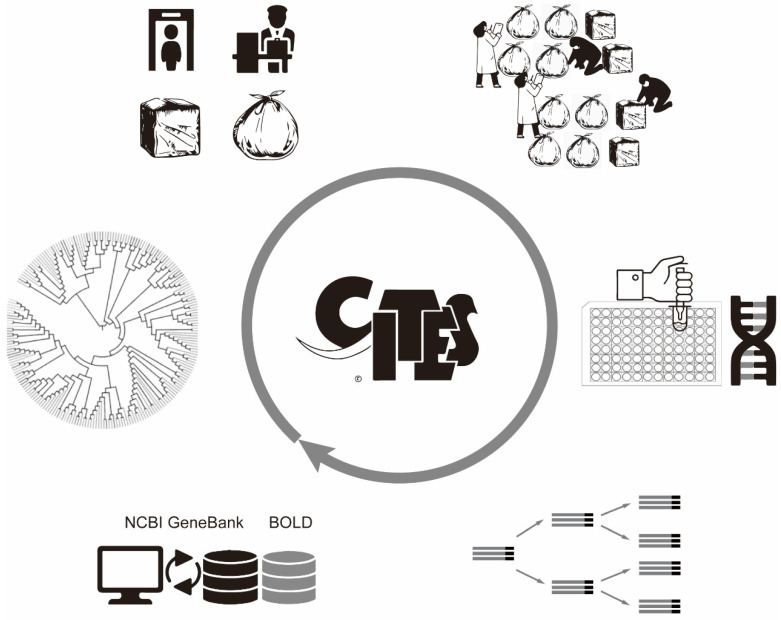
Schematic diagram of the entire case process.

CITES plays a crucial role in regulating the global trade of wildlife to ensure its survival [22]. Under this international agreement, more than 40,000 species are categorized into one of three appendices [23], each indicating the level of protection required to prevent overexploitation of these species. Appendix I includes species that are threatened with extinction. Trade in these species is permitted only under exceptional circumstances, such as for scientific research purposes. For instance, species like the Siberian tiger and the giant panda fall under this category, where strict regulations are enforced to prevent any commercial trade that might further threaten their survival. Appendix II consists of species that are not necessarily threatened with extinction but may become threatened if their trade is not strictly regulated. This appendix allows for regulated trade through a system of permits and quotas. Appendix III contains species that are protected in at least one country, which has requested assistance from other parties in controlling trade. This appendix often includes regional species for which local conservation efforts need international cooperation to be effective. In this case, the shark fins seized by the coast guard were identified as originating from four species: *Alopias pelagicus*, *Alopias superciliosus*, *Carcharhinus falciformis*, and *Isurus oxyrinchus*. All of these species are listed in Appendix II of CITES. Notably, *Alopias pelagicus* and *Alopias superciliosus* belong to the genus *Alopias*, all species of which are included in Appendix II. Additionally, *Carcharhinus falciformis* belongs to the family *Carcharhinidae*, which also has all its species listed in Appendix II. Trade involving these species must be strictly controlled to ensure their survival. Therefore, as illustrated in the entire process of the case presented in Figure 4, accurate forensic species identification played a crucial role in this case, providing essential support for subsequent legal procedures.

The forensic significance of this study lies in its ability to provide a quantitative estimate of the number of CITES-listed specimens in a large seizure using a minimal number of DNA tests. In wildlife trafficking cases, the magnitude of the offense, which is directly related to the number of protected species, influences the severity of criminal penalties, including fines and imprisonment. Traditional morphological identification would require examining every single specimen, which is impractical for shipments of nearly 20,000 items. By applying a statistically validated sampling strategy (*n* = 325) combined with Bayesian inference, we estimated with a 95% credible interval that between 17,351 and 18,170 specimens are CITES Appendix II species. This estimate provides prosecutors and judges with a legally defensible quantitative basis for sentencing. Moreover, the same approach can be adapted to other large-scale wildlife seizures to support forensic casework.

## 4. Materials and Methods

### 4.1. Morphometric Inventory and Categorization

With assistance from professionals, all seized fish maws and shark fins involved in the case were categorized and counted based on size (length > 30 cm/length ≤ 30 cm) and general shape (discoid/cylindrical). The classification results presented are as follows:

Fish maws:

Large-sized discoid: 747 specimens

Small-sized discoid: 328 specimens

Large-sized cylindrical: 24 specimens

Small-sized cylindrical: 678 specimens

Total: 1777 specimens.

Shark fins:

Large-sized: 5779 specimens

Small-sized: 12,391 specimens

Total: 18,170 specimens.

### 4.2. Sampling Design

#### 4.2.1. Sample Size Determination

To estimate the proportion p of specimens classified as endangered wildlife within each morphological category, a simple random sampling (SRS) strategy was implemented. The minimum sample size per category was derived to ensure a 95% confidence level with an absolute error margin of d = 0.1225 (12.25%). Ideally, the sample size needed depends on the true proportion to be estimated. As the proportion is unknown, to be conservative, we determine the needed sample size using the largest variance. The variance p(1−p) takes its maximum at p=0.5. Therefore, we determine the needed sample size by the following formula

$$n=\frac{z_{a/2}^{2}\cdot p(1-p)}{d^{2}}=\frac{1.96^{2}\cdot 0.5\cdot 0.5}{0.1225^{2}}\approx 64$$

#### 4.2.2. Sample Size Adjustment

When the population size N was finite and the sample size $n_{initial}$ exceeded 5% of N, a finite population correction (FPC) was applied to refine the required sample size:

$$n_{adjusted}=\frac{n_{initial}\cdot N}{n_{initial}+N-1}$$

Note: In cases where the morphological category’s population size N was smaller than the initially determined sample size $n_{initial}$, a full census was conducted to ensure statistical validity.

#### 4.2.3. Random Sampling

After the sample size for each category was determined and adjusted, a systematic random sampling approach was utilized. For large-sized cylindrical fish maws, all 24 available samples were selected due to the limited sample size in this category. For other categories, a multi-stage random sampling procedure was implemented to ensure randomness within each morphological category. First, each category was subdivided into several smaller groups based on the total quantity of specimens. Subsequently, 2 to 3 individual containers were randomly selected from each group using a simple random sampling method through a lottery draw from shuffled lots. For each subgroup, a simple random sampling method was applied by drawing lots; all containers were assigned numbers, and numbers were drawn randomly from a shuffled set. The selected containers were then opened, and all specimens within those containers were pooled as the composite sample for that category. This approach avoids selection bias that might arise from manually picking individual specimens. Furthermore, to mitigate potential bias introduced by the preliminary morphological classification, the sampling was performed after classification but was blind to any other information. All specimens within a selected container were taken, regardless of their individual appearance, thus ensuring that the sample was representative of that morphological class.

### 4.3. DNA Extraction

After conducting and cleaning all the random sampling, approximately 20–25 mg of the chopped tissue was processed for genomic DNA extraction using the DNeasy Blood & Tissue Kit (Qiagen, Hilden, Germany) according to the manufacturer’s protocol. The extracted DNA was subsequently quantified using the Qubit dsDNA HS Assay Kit (Thermo Fisher Scientific, Waltham, MA, USA), following the manufacturer’s instructions.

### 4.4. Polymerase Chain Reaction (PCR) and Sanger Sequencing

Extracted genomic DNA templates underwent PCR amplification targeting the mitochondrial COI barcode fragment (primary and mandatory target) using primers COI-F: 5′-TCAACCAACCACAAAGACATTGGCAC-3′ and COI-R: 5′-TAGACTTCTGGGTGGCCAAAGAATCA-3′ [24]. The mitochondrial 16S barcode fragment was amplified as a supplementary target only when either (a) COI provided insufficient taxonomic resolution, or (b) COI-based identification indicated a potentially endangered species, using primers 16S-F: 5′-CGCCTGTTTATCAAAAACAT-3′ and 16S-R: 5′-CCGGTCTGAACTCAGATCACGT-3′ [25]. Zebrafish DNA and deionized water were used as the positive control and negative control, respectively. PCRs were performed in 50 μL volumes containing 25 μL 2× Platinum™ Taq PCR Master Mix (Thermo Fisher Scientific), 0.2 μM of each primer, and 1 ng DNA template. Thermocycling conditions comprised: initial denaturation at 94 °C for 3 min; 35 cycles of 94 °C for 30 s, 52 °C (COI) or 53 °C (16S) for 1 min, and 72 °C for 1 min; followed by a final extension at 72 °C for 10 min. Amplicons were purified using the QIAquick PCR Purification Kit (Qiagen) and bidirectionally sequenced on an Applied Biosystems™ 3730xl Genetic Analyzer (Thermo Fisher Scientific) with BigDye™ Terminator v3.1 chemistry (Thermo Fisher Scientific). All PCR amplicons were verified by agarose gel electrophoresis (1.5% agarose) to confirm the expected fragment lengths (approximately 500–600 bp). Sanger sequencing chromatograms were analyzed using Sequencing Analysis Software 7 (Thermo Fisher Scientific). Sequences were retained only when (i) at least 90% of nucleotide positions had Phred quality scores ≥20, (ii) forward and reverse reads showed >99% agreement across the trimmed overlapping region, and (iii) no ambiguous bases were present in the final consensus sequence. Sequences that failed these criteria were re-amplified and re-sequenced.

### 4.5. Sequence Alignment, Species Identification, and Phylogenetic Tree Construction

Two databases were employed for sequence alignment and species identification. The COI and 16S barcode sequences were aligned using the Basic Local Alignment Search Tool (BLAST: https://blast.ncbi.nlm.nih.gov/) against the GenBank database hosted by the National Center for Biotechnology Information (NCBI). For species-level validation, the COI barcode sequences were additionally queried through the Barcode of Life Data Systems (BOLD: http://www.boldsystems.org/) using its integrated IDENTIFICATION engine, which specializes in DNA barcode comparisons. The threshold for species identification is set at an identification score exceeding 99%. The MEGA X software (version 10, https://www.megasoftware.net) was employed for sequence alignment and comparison, with reference sequences retrieved from the GenBank or BOLD database. Subsequently, phylogenetic trees were constructed using the Neighbor-Joining method based on the Kimura two-parameter model to ensure robust evolutionary distance estimation.

## 5. Conclusions

In summary, we describe the application of DNA barcoding for forensic species identification and a large-sample inference strategy for population estimation. This approach enabled the successful completion of a forensic investigation involving trafficked fish maws and shark fins, thereby providing a critical and valuable case study for combating illegal wildlife trade. The successful identification in this case indicates that the integration of multiple DNA barcode markers, sequence alignment across various DNA databases, and the construction of phylogenetic trees for analysis significantly enhance the accuracy of species identification. This cumulative analysis of species identification can provide reliable evidence for recognizing species diversity, which is crucial for combating illegal wildlife trade and other wildlife crimes. Furthermore, the development of rapid and portable DNA-based screening devices could allow for preliminary on-site testing to justify the seizure of unknown and suspected wildlife products for more comprehensive laboratory confirmation. However, it should be acknowledged that the accuracy of forensic species identification relies heavily on the completeness and quality of reference sequences in GenBank and BOLD.

Overall, this case report illustrates an application of classical DNA barcoding using Sanger sequencing for forensic species identification, involving a large sample size, in support of global biodiversity conservation under the framework of CITES.

## Figures and Tables

**Table 1:** The categories, sampling, and detection of the overall specimens.

Trafficked Biological Materials	Morphological Categories	Counts	Samples	COI	16S (Supplementary Marker)
Fish maws	Large-sized discoid	747	60	60	0
	Small-sized discoid	328	54	54	0
	Large-sized cylindrical	24	24	24	6
	Small-sized cylindrical	678	59	57	24
Shark fins	Large-sized	5779	64	64	64
	Small-sized	12,391	64	64	64

**Table 2:** The species distribution of samples classified according to morphological categories.

Order	Family	Genera	Species	Large-Sized Discoid Fish Maws	Small-Sized Discoid Fish Maws	Large-Sized Cylindrical Fish Maws	Small-Sized Cylindrical Fish Maws	Large-Sized Shark Fins	Small-Sized Shark Fins	Total
*Perciformes*	*Polynemidae*	*Galeoides*	*Galeoides decadactylus*	44	54		5			103
*Perciformes*	*Sciaenidae*	*Pseudotolithus*	*Pseudotolithus brachygnathus*	16		18	28			62
*Perciformes*	*Sciaenidae*	*Pseudotolithus*	*Pseudotolithus typus*			6	24			30
*Lamniformes*	*Alopiidae*	*Alopias*	*Alopias pelagicus*					23	15	38
*Lamniformes*	*Alopiidae*	*Alopias*	*Alopias superciliosus*					18	13	31
*Lamniformes*	*Lamnidae*	*Isurus*	*Isurus oxyrinchus*					3	8	11
*Carcharhiniformes*	*Carcharhinidae*	*Carcharhinus*	*Carcharhinus falciformis*					20	28	48
			Total	60	54	24	57 (2)	64	64	323 (2)

## Data Availability

The original contributions presented in this study are included in the article/Supplementary Material. Further inquiries can be directed to the corresponding authors.

## Supplementary Materials

The following supporting information can be downloaded at: https://www.mdpi.com/article/10.3390/ijms27135813/s1.

## Abbreviations

The following abbreviations are used in this manuscript:

CITES	Convention on International Trade in Endangered Species of Wild Fauna and Flora
COI	Cytochrome c Oxidase I
Cytb	Cytochrome b
16S rRNA	16S ribosomal RNA
BLAST	Basic Local Alignment Search Tool
NCBI	National Center for Biotechnology Information
BOLD	Barcode of Life Data System
MPS	massively parallel sequencing
