# Supplementary material for: Forensic Species Identification: A Case Involving Trafficked Fish Maws and Shark Fins
Source: Int J Mol Sci. 2026 Jun 27;27(13):5813. doi: 10.3390/ijms27135813 (PMC13361761; doi:10.3390/ijms27135813)
Supplement: Supplementary file 1 [file ijms-27-05813-s001.zip › Supplementary materials.pdf]

Fish maw: COI gene - Large-sized discoid samples (n=60)

No.2025-166-22-1

ATAGTGGGGACGGCCCTAAGCCTTTTAATTCGTGCGGAACTTAGCCAACCCGGCGCCCTCCTAGGAGACGACCAAATATATAAT
GTTATCGTTACTGCACACGCCTTCGTAATAATCTTTTTTATAGTTATGCCAATTATAATTGGAGGTTTCGGGAACTGACTTGTACCT
TTAATAATTGGCGCCCCTGACATGGCATTCCCTCGAATAAACAACATGAGCTTCTGACTACTCCCTCCTTCCTTTCTGCTCCTTCTC
GCCTCCTCTGGGGTTGAAGCTGGAGCCGGAACAGGTTGGACCGTATATCCTCCTCTAGCAAGCAACGTCGCCCACTCCAGGAG
CATCAGTTGACTTAACTATTTTTTCCCTCCACTTAGCGGGTGTTTCATCCATTCTTGGGGCAATTAATTTTATCACAACCATCTTTAA
TATAAAACCTGCCGCTGCTTCAATATATCAAATACCCCTGTTCGTTTGGGCCGTTCTAGTTACAGCAGTACTACTTCTTCTTTCTCT
CCCTGTCTTAGCCGCTGGCATCACTATACTAT

No.2025-166-22-2

ATAGTGGGGACGGCCCTAAGCCTTTTAATTCGTGCGGAACTTAGCCAACCCGGCGCCCTCCTAGGAGACGACCAAATATATAAT
GTTATCGTTACTGCACACGCCTTCGTAATAATCTTTTTTATAGTTATGCCAATTATAATTGGAGGTTTCGGGAACTGACTTGTACCT
TTAATAATTGGCGCCCCTGACATGGCATTCCCTCGAATAAACAACATGAGCTTCTGACTACTCCCTCCTTCCTTTCTGCTCCTTCTC
GCCTCCTCTGGGGTTGAAGCTGGAGCCGGAACAGGTTGGACCGTATATCCTCCTCTAGCAAGCAACGTCGCCCACTCCAGGAG
CATCAGTTGACTTAACTATTTTTTCCCTCCACTTAGCGGGTGTTTCATCCATTCTTGGGGCAATTAATTTTATCACAACCATCTTTAA
TATAAAACCTGCCGCTGCTTCAATATATCAAATACCCCTGTTCGTTTGGGCCGTTCTAGTTACAGCAGTACTACTTCTTCTTTCTCT
CCCTGTCTTAGCCGCTGGCATCACTATACTATTAACGGATCGAA

No.2025-166-22-3

TGAGCCGGAATAGTGGGGACGGCCCTAAGCCTTTTAATTCGTGCGGAACTTAGCCAACCCGGCGCCCTCCTAGGAGACGACCA
AATATATAATGTTATCGTTACTGCACACGCCTTCGTAATAATCTTTTTTATAGTTATGCCAATTATAATTGGAGGTTTCGGGAACTGA
CTTGTACCTTTAATAATTGGCGCCCCTGACATGGCATTCCCTCGAATAAACAACATGAGCTTCTGACTACTCCCTCCTTCCTTTCTG
CTCCTTCTCGCCTCCTCTGGGGTTGAAGCTGGAGCCGGAACAGGTTGGACCGTATATCCTCCTCTAGCAAGCAACGTCGCCCAC
TCCAGGAGCATCAGTTGACTTAACTATTTTTTCCCTCCACTTAGCGGGTGTTTCATCCATTCTTGGGGCAATTAATTTTATCACAA
CCATCTTTAATATAAAACCTGCCGCTGCTTCAATATATCAAATACCCCTGTTCGTTTGGGCCGTTCTAGTTACAGCAGTACTACTTC
TTCTTTCTCTCCCTGTCTTAGCCGCTGGCATCACTATACTATTAACGGATC

No.2025-166-22-4

TGAGCCGGAATAGTGGGGACGGCCCTAAGCCTTTTAATTCGTGCGGAACTTAGCCAACCCGGCGCCCTCCTAGGAGACGACCA
AATATATAATGTTATCGTTACTGCACACGCCTTCGTAATAATCTTTTTTATAGTTATGCCAATTATAATTGGAGGTTTCGGGAACTGA
CTTGTACCTTTAATAATTGGCGCCCCTGACATGGCATTCCCTCGAATAAACAACATGAGCTTCTGACTACTCCCTCCTTCCTTTCTG
CTCCTTCTCGCCTCCTCTGGGGTTGAAGCTGGAGCCGGAACAGGTTGGACCGTATATCCTCCTCTAGCAAGCAACGTCGCCCAC
TCCAGGAGCATCAGTTGACTTAACTATTTTTTCCCTCCACTTAGCGGGTGTTTCATCCATTCTTGGGGCAATTAATTTTATCACAA
CCATCTTTAATATAAAACCTGCCGCTGCTTCAATATATCAAATACCCCTGTTCGTTTGGGCCGTTCTAGTTACAGCAGTACTACTTC
TTCTTTCTCTCCCTGTCTTAGCCGCTGGCATCACTATACTATTAACGGATCGAA

No.2025-166-22-5

TGAGCCGGAATAGTGGGGACGGCCCTAAGCCTTTTAATTCGTGCGGAACTTAGCCAACCCGGCGCCCTCCTAGGAGACGACCA
AATATATAATGTTATCGTTACTGCACACGCCTTCGTAATAATCTTTTTTATAGTTATGCCAATTATAATTGGAGGTTTCGGGAACTGA
CTTGTACCTTTAATAATTGGCGCCCCTGACATGGCATTCCCTCGAATAAACAACATGAGCTTCTGACTACTCCCTCCTTCCTTTCTG
CTCCTTCTCGCCTCCTCTGGGGTTGAAGCTGGAGCCGGAACAGGTTGGACCGTATATCCTCCTCTAGCAAGCAACGTCGCCCAC
TCCAGGAGCATCAGTTGACTTAACTATTTTTTCCCTCCACTTAGCGGGTGTTTCATCCATTCTTGGGGCAATTAATTTTATCACAA
CCATCTTTAATATAAAACCTGCCGCTGCTTCAATATATCAAATACCCCTGTTCGTTTGGGCCGTTCTAGTTACAGCAGTACTACTTC
TTCTTTCTCTCCCTGTCTTAGCCGCTGGCATCACTATACTATTAACGGATC

No.2025-166-22-6

TGAGCCGGAATAGTGGGGACGGCCCTAAGCCTTTTAATTCGTGCGGAACTTAGCCAACCCGGCGCCCTCCTAGGAGACGACCA
AATATATAATGTTATCGTTACTGCACACGCCTTCGTAATAATCTTTTTTATAGTTATGCCAATTATAATTGGAGGTTTCGGGAACTGA
CTTGTACCTTTAATAATTGGCGCCCCTGACATGGCATTCCCTCGAATAAACAACATGAGCTTCTGACTACTCCCTCCTTCCTTTCTG
CTCCTTCTCGCCTCCTCTGGGGTTGAAGCTGGAGCCGGAACAGGTTGGACCGTATATCCTCCTCTAGCAAGCAACGTCGCCCAC
TCCAGGAGCATCAGTTGACTTAACTATTTTTTCCCTCCACTTAGCGGGTGTTTCATCCATTCTTGGGGCAATTAATTTTATCACAA
CCATCTTTAATATAAAACCTGCCGCTGCTTCAATATATCAAATACCCCTGTTCGTTTGGGCCGTTCTAGTTACAGCAGTACTACTTC
TTCTTTCTCTCCCTGTCTTAGCCGCTGGCATCACTATACTAT

No.2025-166-22-7

TGAGCCGGAATAGTGGGGACGGCCCTAAGCCTTTTAATTCGTGCGGAACTTAGCCAACCCGGCGCCCTCCTAGGAGACGACCA
AATATATAATGTTATCGTTACTGCACACGCCTTCGTAATAATCTTTTTTATAGTTATGCCAATTATAATTGGAGGTTTCGGGAACTGA
CTTGTACCTTTAATAATTGGCGCCCCTGACATGGCATTCCCTCGAATAAACAACATGAGCTTCTGACTACTCCCTCCTTCCTTTCTG
CTCCTTCTCGCCTCCTCTGGGGTTGAAGCTGGAGCCGGAACAGGTTGGACCGTATATCCTCCTCTAGCAAGCAACGTCGCCCAC
TCCAGGAGCATCAGTTGACTTAACTATTTTTTCCCTCCACTTAGCGGGTGTTTCATCCATTCTTGGGGCAATTAATTTTATCACAA
CCATCTTTAATATAAAACCTGCCGCTGCTTCAATATATCAAATACCCCTGTTCGTTTGGGCCGTTCTAGTTACAGCAGTACTACTTC
TTCTTTCTCTCCCTGTCTTAGCCGCTGGCATCACTATACTATTAACG

No.2025-166-22-8

TGAGCCGGAATAGTGGGGACGGCCCTAAGCCTTTTAATTCGTGCGGAACTTAGCCAACCCGGCGCCCTCCTAGGAGACGACCA
AATATATAATGTTATCGTTACTGCACACGCCTTCGTAATAATCTTTTTTATAGTTATGCCAATTATAATTGGAGGTTTCGGGAACTGA
CTTGTACCTTTAATAATTGGCGCCCCTGACATGGCATTCCCTCGAATAAACAACATGAGCTTCTGACTACTCCCTCCTTCCTTTCTG
CTCCTTCTCGCCTCCTCTGGGGTTGAAGCTGGAGCCGGAACAGGTTGGACCGTATATCCTCCTCTAGCAAGCAACGTCGCCCAC
TCCAGGAGCATCAGTTGACTTAACTATTTTTTCCCTCCACTTAGCGGGTGTTTCATCCATTCTTGGGGCAATTAATTTTATCACAA

CCATCTTTAATATAAAACCTGCCGCTGCTTCAATATATCAAATACCCCTGTTCGTTTGGGCCGTTCTAGTTACAGCAGTACTACTTC
TTCTTTCTCTCCCTGTCTTAGCCGCTGGCATCACTATACTAT

No.2025-166-22-9

ATAGTGGGGACGGCCCTAAGCCTTTTAATTCGTGCGGAACTTAGCCAACCCGGCGCCCTCCTAGGAGACGACCAAATATATAAT
GTTATCGTTACTGCACACGCCTTCGTAATAATCTTTTTTATAGTTATGCCAATTATAATTGGAGGTTTCGGGAACTGACTTGTACCT
TTAATAATTGGCGCCCCTGACATGGCATTCCCTCGAATAAACAACATGAGCTTCTGACTACTCCCTCCTTCCTTTCTGCTCCTTCTC
GCCTCCTCTGGGGTTGAAGCTGGAGCCGGAACAGGTTGGACCGTATATCCTCCTCTAGCAAGCAACGTCGCCCACTCCAGGAG
CATCAGTTGACTTAACTATTTTTTCCCTCCACTTAGCGGGTGTTTCATCCATTCTTGGGGCAATTAATTTTATCACAACCATCTTTAA
TATAAAACCTGCCGCTGCTTCAATATATCAAATACCCCTGTTCGTTTGGGCCGTTCTAGTTACAGCAGTACTACTTCTTCTTTCTCT
CCCTGTCTTAGCCGCTGGCATCACTATACTAT

No.2025-166-22-10

ATAGTGGGGACGGCCCTAAGCCTTTTAATTCGTGCGGAACTTAGCCAACCCGGCGCCCTCCTAGGAGACGACCAAATATATAAT
GTTATCGTTACTGCACACGCCTTCGTAATAATCTTTTTTATAGTTATGCCAATTATAATTGGAGGTTTCGGGAACTGACTTGTACCT
TTAATAATTGGCGCCCCTGACATGGCATTCCCTCGAATAAACAACATGAGCTTCTGACTACTCCCTCCTTCCTTTCTGCTCCTTCTC
GCCTCCTCTGGGGTTGAAGCTGGAGCCGGAACAGGTTGGACCGTATATCCTCCTCTAGCAAGCAACGTCGCCCACTCCAGGAG
CATCAGTTGACTTAACTATTTTTTCCCTCCACTTAGCGGGTGTTTCATCCATTCTTGGGGCAATTAATTTTATCACAACCATCTTTAA
TATAAAACCTGCCGCTGCTTCAATATATCAAATACCCCTGTTCGTTTGGGCCGTTCTAGTTACAGCAGTACTACTTCTTCTTTCTCT
CCCTGTCTTAGCCGCTGGCATCACTATACTATTAACG

No.2025-166-22-11

ATAGTGGGGACGGCCCTAAGCCTTTTAATTCGTGCGGAACTTAGCCAACCCGGCGCCCTCCTAGGAGACGACCAAATATATAAT
GTTATCGTTACTGCACACGCCTTCGTAATAATCTTTTTTATAGTTATGCCAATTATAATTGGAGGTTTCGGGAACTGACTTGTACCT
TTAATAATTGGCGCCCCTGACATGGCATTCCCTCGAATAAACAACATGAGCTTCTGACTACTCCCTCCTTCCTTTCTGCTCCTTCTC
GCCTCCTCTGGGGTTGAAGCTGGAGCCGGAACAGGTTGGACCGTATATCCTCCTCTAGCAAGCAACGTCGCCCACTCCAGGAG
CATCAGTTGACTTAACTATTTTTTCCCTCCACTTAGCGGGTGTTTCATCCATTCTTGGGGCAATTAATTTTATCACAACCATCTTTAA
TATAAAACCTGCCGCTGCTTCAATATATCAAATACCCCTGTTCGTTTGGGCCGTTCTAGTTACAGCAGTACTACTTCTTCTTTCTCT
CCCTGTCTTAGCCGCTGGCATCACTATACTAT

No.2025-166-22-12

TGAGCCGGAATAGTGGGGACGGCCCTAAGCCTTTTAATTCGTGCGGAACTTAGCCAACCCGGCGCCCTCCTAGGAGACGACCA
AATATATAATGTTATCGTTACTGCACACGCCTTCGTAATAATCTTTTTTATAGTTATGCCAATTATAATTGGAGGTTTCGGGAACTGA
CTTGTACCTTTAATAATTGGCGCCCCTGACATGGCATTCCCTCGAATAAACAACATGAGCTTCTGACTACTCCCTCCTTCCTTTCTG
CTCCTTCTCGCCTCCTCTGGGGTTGAAGCTGGAGCCGGAACAGGTTGGACCGTATATCCTCCTCTAGCAAGCAACGTCGCCCAC
TCCAGGAGCATCAGTTGACTTAACTATTTTTTCCCTCCACTTAGCGGGTGTTTCATCCATTCTTGGGGCAATTAATTTTATCACAA
CCATCTTTAATATAAAACCTGCCGCTGCTTCAATATATCAAATACCCCTGTTCGTTTGGGCCGTTCTAGTTACAGCAGTACTACTTC
TTCTTTCTCTCCCTGTCTTAGCCGCTGGCATCACTATACTAT

No.2025-166-22-13

TGAGCCGGAATAGTGGGGACGGCCCTAAGCCTTTTAATTCGTGCGGAACTTAGCCAACCCGGCGCCCTCCTAGGAGACGACCA
AATATATAATGTTATCGTTACTGCACACGCCTTCGTAATAATCTTTTTTATAGTTATGCCAATTATAATTGGAGGTTTCGGGAACTGA
CTTGTACCTTTAATAATTGGCGCCCCTGACATGGCATTCCCTCGAATAAACAACATGAGCTTCTGACTACTCCCTCCTTCCTTTCTG
CTCCTTCTCGCCTCCTCTGGGGTTGAAGCTGGAGCCGGAACAGGTTGGACCGTATATCCTCCTCTAGCAAGCAACGTCGCCCAC
TCCAGGAGCATCAGTTGACTTAACTATTTTTTCCCTCCACTTAGCGGGTGTTTCATCCATTCTTGGGGCAATTAATTTTATCACAA
CCATCTTTAATATAAAACCTGCCGCTGCTTCAATATATCAAATACCCCTGTTCGTTTGGGCCGTTCTAGTTACAGCAGTACTACTTC
TTCTTTCTCTCCCTGTCTTAGCCGCTGGCATCACTATACTAT

No.2025-166-22-14

ATAGTGGGGACGGCCCTAAGCCTTTTAATTCGTGCGGAACTTAGCCAACCCGGCGCCCTCCTAGGAGACGACCAAATATATAAT
GTTATCGTTACTGCACACGCCTTCGTAATAATCTTTTTTATAGTTATGCCAATTATAATTGGAGGTTTCGGGAACTGACTTGTACCT
TTAATAATTGGCGCCCCTGACATGGCATTCCCTCGAATAAACAACATGAGCTTCTGACTACTCCCTCCTTCCTTTCTGCTCCTTCTC
GCCTCCTCTGGGGTTGAAGCTGGAGCCGGAACAGGTTGGACCGTATATCCTCCTCTAGCAAGCAACGTCGCCCACTCCAGGAG
CATCAGTTGACTTAACTATTTTTTCCCTCCACTTAGCGGGTGTTTCATCCATTCTTGGGGCAATTAATTTTATCACAACCATCTTTAA
TATAAAACCTGCCGCTGCTTCAATATATCAAATACCCCTGTTCGTTTGGGCCGTTCTAGTTACAGCAGTACTACTTCTTCTTTCTCT
CCCTGTCTTAGCCGCTGGCATCACTATACTAT

No.2025-166-22-15

ATAGTGGGGACGGCCCTAAGCCTTTTAATTCGTGCGGAACTTAGCCAACCCGGCGCCCTCCTAGGAGACGACCAAATATATAAT
GTTATCGTTACTGCACACGCCTTCGTAATAATCTTTTTTATAGTTATGCCAATTATAATTGGAGGTTTCGGGAACTGACTTGTACCT
TTAATAATTGGCGCCCCTGACATGGCATTCCCTCGAATAAACAACATGAGCTTCTGACTACTCCCTCCTTCCTTTCTGCTCCTTCTC
GCCTCCTCTGGGGTTGAAGCTGGAGCCGGAACAGGTTGGACCGTATATCCTCCTCTAGCAAGCAACGTCGCCCACTCCAGGAG
CATCAGTTGACTTAACTATTTTTTCCCTCCACTTAGCGGGTGTTTCATCCATTCTTGGGGCAATTAATTTTATCACAACCATCTTTAA
TATAAAACCTGCCGCTGCTTCAATATATCAAATACCCCTGTTCGTTTGGGCCGTTCTAGTTACAGCAGTACTACTTCTTCTTTCTCT
CCCTGTCTTAGCCGCTGGCATCACTATACTAT

No.2025-166-22-16

TGAGCCGGAATAGTGGGGACGGCCCTAAGCCTTTTAATTCGTGCGGAACTTAGCCAACCCGGCGCCCTCCTAGGAGACGACCA
AATATATAATGTTATCGTTACTGCACACGCCTTCGTAATAATCTTTTTTATAGTTATGCCAATTATAATTGGAGGTTTCGGGAACTGA
CTTGTACCTTTAATAATTGGCGCCCCTGACATGGCATTCCCTCGAATAAACAACATGAGCTTCTGACTACTCCCTCCTTCCTTTCTG
CTCCTTCTCGCCTCCTCTGGGGTTGAAGCTGGAGCCGGAACAGGTTGGACCGTATATCCTCCTCTAGCAAGCAACGTCGCCCAC

TCCAGGAGCATCAGTTGACTTAACTATTTTTTCCCTCCACTTAGCGGGTGTTTCATCCATTCTTGGGGCAATTAATTTTATCACAA
CCATCTTTAATATAAAACCTGCCGCTGCTTCAATATATCAAATACCCCTGTTCGTTTGGGCCGTTCTAGTTACAGCAGTACTACTTC
TTCTTTCTCTCCCTGTCTTAGCCGCTGGCATCACTATACTATTAACGGATCGAA

No.2025-166-22-17

ATAGTGGGGACGGCCCTAAGCCTTTTAATTCGTGCGGAACTTAGCCAACCCGGCGCCCTCCTAGGAGACGACCAAATATATAAT
GTTATCGTTACTGCACACGCCTTCGTAATAATCTTTTTTATAGTTATGCCAATTATAATTGGAGGTTTCGGGAACTGACTTGTACCT
TTAATAATTGGCGCCCCTGACATGGCATTCCCTCGAATAAACAACATGAGCTTCTGACTACTCCCTCCTTCCTTTCTGCTCCTTCTC
GCCTCCTCTGGGGTTGAAGCTGGAGCCGGAACAGGTTGGACCGTATATCCTCCTCTAGCAAGCAACGTCGCCCACTCCAGGAG
CATCAGTTGACTTAACTATTTTTTCCCTCCACTTAGCGGGTGTTTCATCCATTCTTGGGGCAATTAATTTTATCACAACCATCTTTAA
TATAAAACCTGCCGCTGCTTCAATATATCAAATACCCCTGTTCGTTTGGGCCGTTCTAGTTACAGCAGTACTACTTCTTCTTTCTCT
CCCTGTCTTAGCCGCTGGCATCACTATACTATTAACGGATCGAAAC

No.2025-166-22-18

TGAGCCGGAATAGTGGGGACGGCCCTAAGCCTTTTAATTCGTGCGGAACTTAGCCAACCCGGCGCCCTCCTAGGAGACGACCA
AATATATAATGTTATCGTTACTGCACACGCCTTCGTAATAATCTTTTTTATAGTTATGCCAATTATAATTGGAGGTTTCGGGAACTGA
CTTGTACCTTTAATAATTGGCGCCCCTGACATGGCATTCCCTCGAATAAACAACATGAGCTTCTGACTACTCCCTCCTTCCTTTCTG
CTCCTTCTCGCCTCCTCTGGGGTTGAAGCTGGAGCCGGAACAGGTTGGACCGTATATCCTCCTCTAGCAAGCAACGTCGCCCAC
TCCAGGAGCATCAGTTGACTTAACTATTTTTTCCCTCCACTTAGCGGGTGTTTCATCCATTCTTGGGGCAATTAATTTTATCACAA
CCATCTTTAATATAAAACCTGCCGCTGCTTCAATATATCAAATACCCCTGTTCGTTTGGGCCGTTCTAGTTACAGCAGTACTACTTC
TTCTTTCTCTCCCTGTCTTAGCCGCTGGCATCACTATACTATTAACGGATCGA

No.2025-166-22-19

TGAGCCGGAATAGTGGGGACGGCCCTAAGCCTTTTAATTCGTGCGGAACTTAGCCAACCCGGCGCCCTCCTAGGAGACGACCA
AATATATAATGTTATCGTTACTGCACACGCCTTCGTAATAATCTTTTTTATAGTTATGCCAATTATAATTGGAGGTTTCGGGAACTGA
CTTGTACCTTTAATAATTGGCGCCCCTGACATGGCATTCCCTCGAATAAACAACATGAGCTTCTGACTACTCCCTCCTTCCTTTCTG
CTCCTTCTCGCCTCCTCTGGGGTTGAAGCTGGAGCCGGAACAGGTTGGACCGTATATCCTCCTCTAGCAAGCAACGTCGCCCAC
TCCAGGAGCATCAGTTGACTTAACTATTTTTTCCCTCCACTTAGCGGGTGTTTCATCCATTCTTGGGGCAATTAATTTTATCACAA
CCATCTTTAATATAAAACCTGCCGCTGCTTCAATATATCAAATACCCCTGTTCGTTTGGGCCGTTCTAGTTACAGCAGTACTACTTC
TTCTTTCTCTCCCTGTCTTAGCCGCTGGCATCACTATACTATTAACGGATCGAA

No.2025-166-22-20

TGAGCCGGAATAGTGGGGACGGCCCTAAGCCTTTTAATTCGTGCGGAACTTAGCCAACCCGGCGCCCTCCTAGGAGACGACCA
AATATATAATGTTATCGTTACTGCACACGCCTTCGTAATAATCTTTTTTATAGTTATGCCAATTATAATTGGAGGTTTCGGGAACTGA
CTTGTACCTTTAATAATTGGCGCCCCTGACATGGCATTCCCTCGAATAAACAACATGAGCTTCTGACTACTCCCTCCTTCCTTTCTG
CTCCTTCTCGCCTCCTCTGGGGTTGAAGCTGGAGCCGGAACAGGTTGGACCGTATATCCTCCTCTAGCAAGCAACGTCGCCCAC
TCCAGGAGCATCAGTTGACTTAACTATTTTTTCCCTCCACTTAGCGGGTGTTTCATCCATTCTTGGGGCAATTAATTTTATCACAA
CCATCTTTAATATAAAACCTGCCGCTGCTTCAATATATCAAATACCCCTGTTCGTTTGGGCCGTTCTAGTTACAGCAGTACTACTTC
TTCTTTCTCTCCCTGTCTTAGCCGCTGGCATCACTATACTATTAACGGATCGAA

No.2025-166-22-21

ATAGTGGGGACGGCCCTAAGCCTTTTAATTCGTGCGGAACTTAGCCAACCCGGCGCCCTCCTAGGAGACGACCAAATATATAAT
GTTATCGTTACTGCACACGCCTTCGTAATAATCTTTTTTATAGTTATGCCAATTATAATTGGAGGTTTCGGGAACTGACTTGTACCT
TTAATAATTGGCGCCCCTGACATGGCATTCCCTCGAATAAACAACATGAGCTTCTGACTACTCCCTCCTTCCTTTCTGCTCCTTCTC
GCCTCCTCTGGGGTTGAAGCTGGAGCCGGAACAGGTTGGACCGTATATCCTCCTCTAGCAAGCAACGTCGCCCACTCCAGGAG
CATCAGTTGACTTAACTATTTTTTCCCTCCACTTAGCGGGTGTTTCATCCATTCTTGGGGCAATTAATTTTATCACAACCATCTTTAA
TATAAAACCTGCCGCTGCTTCAATATATCAAATACCCCTGTTCGTTTGGGCCGTTCTAGTTACAGCAGTACTACTTCTTCTTTCTCT
CCCTGTCTTAGCCGCTGGCATCACTATACTAT

No.2025-166-22-22

ATAGTGGGGACGGCCCTAAGCCTTTTAATTCGTGCGGAACTTAGCCAACCCGGCGCCCTCCTAGGAGACGACCAAATATATAAT
GTTATCGTTACTGCACACGCCTTCGTAATAATCTTTTTTATAGTTATGCCAATTATAATTGGAGGTTTCGGGAACTGACTTGTACCT
TTAATAATTGGCGCCCCTGACATGGCATTCCCTCGAATAAACAACATGAGCTTCTGACTACTCCCTCCTTCCTTTCTGCTCCTTCTC
GCCTCCTCTGGGGTTGAAGCTGGAGCCGGAACAGGTTGGACCGTATATCCTCCTCTAGCAAGCAACGTCGCCCACTCCAGGAG
CATCAGTTGACTTAACTATTTTTTCCCTCCACTTAGCGGGTGTTTCATCCATTCTTGGGGCAATTAATTTTATCACAACCATCTTTAA
TATAAAACCTGCCGCTGCTTCAATATATCAAATACCCCTGTTCGTTTGGGCCGTTCTAGTTACAGCAGTACTACTTCTTCTTTCTCT
CCCTGTCTTAGCCGCTGGCATCACTATACTATTAACG

No.2025-166-22-23

ATAGTGGGGACGGCCCTAAGCCTTTTAATTCGTGCGGAACTTAGCCAACCCGGCGCCCTCCTAGGAGACGACCAAATATATAAT
GTTATCGTTACTGCACACGCCTTCGTAATAATCTTTTTTATAGTTATGCCAATTATAATTGGAGGTTTCGGGAACTGACTTGTACCT
TTAATAATTGGCGCCCCTGACATGGCATTCCCTCGAATAAACAACATGAGCTTCTGACTACTCCCTCCTTCCTTTCTGCTCCTTCTC
GCCTCCTCTGGGGTTGAAGCTGGAGCCGGAACAGGTTGGACCGTATATCCTCCTCTAGCAAGCAACGTCGCCCACTCCAGGAG
CATCAGTTGACTTAACTATTTTTTCCCTCCACTTAGCGGGTGTTTCATCCATTCTTGGGGCAATTAATTTTATCACAACCATCTTTAA
TATAAAACCTGCCGCTGCTTCAATATATCAAATACCCCTGTTCGTTTGGGCCGTTCTAGTTACAGCAGTACTACTTCTTCTTTCTCT
CCCTGTCTTAGCCGCTGGCATCACTATACTAT

No.2025-166-22-24

AATAGTAGGCACAGCCTTAAGCCTCCTAATCCGAGCAGAACTAAGTCAGCCAGGCTCACTCCTCGGAGATGACCAGATTTTTAA
CGTAATTGTTACGGCACATGCCTTTGTCATGATTTTCTTTATAGTAATACCCGTCATGATTGGAGGGTTCGGAAACTGACTTGTAC
CCCTAATGATCGGAGCCCCCGACATGGCCTTTCCCCGGATGAACAACATAAGCTTCTGGCTTCTCCCCCCTTCTTTCCTGCTACTC

CTCACTTCTTCCGGAGTAGAGGCAGGTGCCGGGACAGGGTGAACAGTCTACCCCCCGCTCGCTGGTAACCTCGCACACGCAG
GTGCTTCTGTCGACCTGGCTATCTTTTCCCTCCATCTCGCAGGTGTATCCTCGATTCTAGGGGCTATTAACTTTATTACAACGATTA
TTAATATGAAGCCCCCAGCAATTTCTCAGTACCAGACACCTTTATTCGTATGGGCCGTCTTAATTACTGCTGTCCTCCTACTACTCT
CACTCCCTGTCCTAGCTGCCGGCATTACAATGCTTCTAACA

No.2025-166-22-25

ATAGTAGGCACAGCCTTAAGCCTCCTAATCCGAGCAGAACTAAGTCAGCCAGGCTCACTCCTCGGAGATGACCAGATTTTTAAC
GTAATTGTTACGGCACATGCCTTTGTCATGATTTTCTTTATAGTAATACCCGTCATGATTGGAGGGTTCGGAAACTGACTTGTACC
CCTAATGATCGGAGCCCCCGACATGGCCTTTCCCCGGATGAACAACATAAGCTTCTGGCTTCTCCCCCCTTCTTTCCTGCTACTCC
TCACTTCTTCCGGAGTAGAGGCAGGTGCCGGGACAGGGTGAACAGTCTACCCCCCGCTCGCTGGTAATCTCGCACACGCAGGT
GCTTCTGTCGACCTGGCTATCTTTTCCCTCCATCTCGCAGGTGTATCCTCGATTCTAGGGGCTATTAACTTTATTACAACGATTATTA
ATATGAAGCCCCCAGCAATTTCTCAGTACCAGACACCTTTATTCGTATGGGCCGTCTTAATTACTGCTGTCCTCCTACTACTCTCAC
TCCCTGTCCTAGCTGCCGGCATTACAA

No.2025-166-22-26

TGGGCCGGAATAGTAGGCACAGCCTTAAGCCTCCTAATCCGAGCAGAACTAAGTCAGCCAGGCTCACTCCTCGGAGATGACCA
GATTTTTAACGTAATTGTTACGGCACATGCCTTTGTCATGATTTTCTTTATAGTAATACCCGTCATGATTGGAGGGTTCGGAAACT
GACTTGTACCCCTAATGATCGGAGCCCCCGACATGGCCTTTCCCCGGATGAACAACATAAGCTTCTGGCTTCTCCCCCCTTCTTT
CCTGCTACTCCTCACTTCTTCCGGAGTAGAGGCAGGTGCCGGGACAGGGTGAACAGTCTACCCCCCGCTCGCTGGTAACCTCG
CACACGCAGGTGCTTCTGTCGACCTGGCTATCTTTTCCCTCCATCTCGCAGGTGTATCCTCGATTCTAGGGGCTATTAACTTTATTA
CAACGATTATTAATATGAAGCCCCCAGCAATTTCTCAGTACCAGACACCTTTATTCGTATGGGCCGTCTTAATTACTGCTGTCCTCC
TACTACTCTCACTCCCTGTCCTAGCTGCCGGCATTACAA

No.2025-166-22-27

TGGGCCGGAATAGTAGGCACAGCCTTAAGCCTCCTAATCCGAGCAGAACTAAGTCAGCCAGGCTCACTCCTCGGAGATGACCA
GATTTTTAACGTAATTGTTACGGCACATGCCTTTGTCATGATTTTCTTTATAGTAATACCCGTCATGATTGGAGGGTTCGGAAACT
GACTTGTACCCCTAATGATCGGAGCCCCCGACATGGCCTTTCCCCGGATGAACAACATAAGCTTCTGGCTTCTCCCCCCTTCTTT
CCTGCTACTCCTCACTTCTTCCGGAGTAGAGGCAGGTGCCGGGACAGGGTGAACAGTCTACCCCCCGCTCGCTGGTAACCTCG
CACACGCAGGTGCTTCTGTCGACCTGGCTATCTTTTCCCTCCATCTCGCAGGTGTATCCTCGATTCTAGGGGCTATTAACTTTATTA
CAACGATTATTAATATGAAGCCCCCAGCAATTTCTCAGTACCAGACACCTTTATTCGTATGGGCCGTCTTAATTACTGCTGTCCTCC
TACTACTCTCACTCCCTGTCCTAGCTGCCGGCATTACAATGCTTCTAACAGACCGCA

No.2025-166-22-28

AATAGTAGGCACAGCCTTAAGCCTCCTAATCCGAGCAGAACTAAGTCAGCCAGGCTCACTCCTCGGAGATGACCAGATTTTTAA
CGTAATTGTTACGGCACATGCCTTTGTCATGATTTTCTTTATAGTAATACCCGTCATGATTGGAGGGTTCGGAAACTGACTTGTAC
CCCTAATGATCGGAGCCCCCGACATGGCCTTTCCCCGGATGAACAACATAAGCTTCTGGCTTCTCCCCCCTTCTTTCCTGCTACTC
CTCACTTCTTCCGGAGTAGAGGCAGGTGCCGGGACAGGGTGAACAGTCTACCCCCCGCTCGCTGGTAACCTCGCACACGCAG
GTGCTTCTGTCGACCTGGCTATCTTTTCCCTCCATCTCGCAGGTGTATCCTCGATTCTAGGGGCTATTAACTTTATTACAACGATTA
TTAATATGAAGCCCCCAGCAATTTCTCAGTACCAGACACCTTTATTCGTATGGGCCGTCTTAATTACTGCTGTCCTCCTACTACTCT
CACTCCCTGTCCTAGCTGCCGGCATTACAATGCTTCTAACA

No.2025-166-22-29

GCATGGGCCGGAATAGTAGGCACAGCCTTAAGCCTCCTAATCCGAGCAGAACTAAGTCAGCCAGGCTCACTCCTCGGAGATGA
CCAGATTTTTAACGTAATTGTTACGGCACATGCCTTTGTCATGATTTTCTTTATAGTAATACCCGTCATGATTGGAGGGTTCGGAA
ACTGACTTGTACCCCTAATGATCGGAGCCCCCGACATGGCCTTTCCCCGGATGAACAACATAAGCTTCTGGCTTCTCCCCCCTTC
TTTCCTGCTACTCCTCACTTCTTCCGGAGTAGAGGCAGGTGCCGGGACAGGGTGAACAGTCTACCCCCCGCTCGCTGGTAACCT
CGCACACGCAGGTGCTTCTGTCGACCTGGCTATCTTTTCCCTCCATCTCGCAGGTGTATCCTCGATTCTAGGGGCTATTAACTTTA
TTACAACGATTATTAATATGAAGCCCCCAGCAATTTCTCAGTACCAGACACCTTTATTCGTATGGGCCGTCTTAATTACTGCTGTCC
TCCTACTACTCTCACTCCCTGTCCTAGCTGCCGGCATTACAATGCTTCTAACAGACCGC

No.2025-166-22-30

TCTACCTAGTTTTCGGTGCATGGGCCGGAATAGTAGGCACAGCCTTAAGCCTCCTAATCCGAGCAGAACTAAGTCAGCCAGGCT
CACTCCTCGGAGATGACCAGATTTTTAACGTAATTGTTACGGCACATGCCTTTGTCATGATTTTCTTTATAGTAATACCCGTCATGA
TTGGAGGGTTCGGAAACTGAGTTGTACCCCTAATGATCGGAGCCCCCGACATGGCCTTTCCCCGGATGAACAACATAAGCTTCT
GGCTTCTCCCCCCTTCTTTCCTGCTACTCCTCACTTCTTCCGGAGTAGAGGCAGGTGCCGGGACAGGGTGAACAGTCTACCCCC
CGCTCGCTGGTAACCTCGCACACGCAGGTGCTTCTGTCGACCTGGCTATCTTTTCCCTCCATCTCGCAGGTGTATCCTCGATTCTA
GGGGCTATTAACTTTATTACAACGATTATTAATATGAAGCCCCCAGCAATTTCTCAGTACCAGACACCTTTATTCGTATGGGCCGTC
TTAATTACTGCTGTCCTCCTACTACTCTCACTCCCTGTCCTAGCTGCCGGCATTACAATGCTTCTAACAGACCGCAACCTCAACACA
ACCTTCTTT

No.2025-166-22-31

TTCGGTGCATGGGCCGGAATAGTAGGCACAGCCTTAAGCCTCCTAATCCGAGCAGAACTAAGTCAGCCAGGCTCACTCCTCGG
AGATGACCAGATTTTTAACGTAATTGTTACGGCACATGCCTTTGTCATGATTTTCTTTATAGTAATACCCGTCATGATTGGAGGGTT
CGGAAACTGACTTGTACCCCTAATGATCGGAGCCCCCGACATGGCCTTTCCCCGGATGAACAACATAAGCTTCTGGCTTCTCCCC
CCTTCTTTCCTGCTACTCCTCACTTCTTCCGGAGTAGAGGCAGGTGCCGGGACAGGGTGAACAGTCTACCCCCCGCTCGCTGGT
AACCTCGCACACGCAGGTGCTTCTGTCGACCTGGCTATCTTTTCCCTCCATCTCGCAGGTGTATCCTCGATTCTAGGGGCTATTAA
CTTTATTACAACGATTATTAATATGAAGCCCCCAGCAATTTCTCAGTACCAGACACCTTTATTCGTATGGGCCGTCTTAATTACTGC
TGTCCTCCTACTACTCTCACTCCCTGTCCTAGCTGCCGGCATTACAATGCTTCTAACAGACCGCAACCTCAACACAACCTTCTTCG
ACCCAGCAGGCGGAGGTGACCCCAT

No.2025-166-22-32

AGTAGGCACAGCCTTAAGCCTCCTAATCCGAGCAGAACTAAGTCAGCCAGGCTCACTCCTCGGAGATGACCAGATTTTTAACGT
AATTGTTACGGCACATGCCTTTGTCATGATTTTCTTTATAGTAATACCCGTCATGATTGGAGGGTTCGGAAACTGACTTGTACCCC
TAATGATCGGAGCCCCCGACATGGCCTTTCCCCGGATGAACAACATAAGCTTCTGGCTTCTCCCCCCTTCTTTCCTGCTACTCCTC
ACTTCTTCCGGAGTAGAGGCAGGTGCCGGGACAGGGTGAACAGTCTACCCCCCGCTCGCTGGTAACCTCGCACACGCAGGTG
CTTCTGTCGACCTGGCTATCTTTTCCCTCCATCTCGCAGGTGTATCCTCGATTCTAGGGGCTATTAACTTTATTACAACGATTATTAA
TATGAAGCCCCCAGCAATTTCTCAGTACCAGACACCTTTATTCGTATGGGCCGTCTTAATTACTGCTGTCCTCCTACTACTCTCACT
CCCTGTCCTAGCTGCCGGCATTACAATGCTTCTAACAG

No.2025-166-22-33

TAGTAGGCACAGCCTTAAGCCTCCTAATCCGAGCAGAACTAAGTCAGCCAGGCTCACTCCTCGGAGATGACCAGATTTTTAACG
TAATTGTTACGGCACATGCCTTTGTCATGATTTTCTTTATAGTAATACCCGTCATGATTGGAGGGTTCGGAAACTGACTTGTACCC
CTAATGATCGGAGCCCCCGACATGGCCTTTCCCCGGATGAACAACATAAGCTTCTGGCTTCTCCCCCCTTCTTTCCTGCTACTCCT
CACTTCTTCCGGAGTAGAGGCAGGTGCCGGGACAGGGTGAACAGTCTACCCCCCGCTCGCTGGTAACCTCGCACACGCAGGT
GCTTCTGTCGACCTGGCTATCTTTTCCCTCCATCTCGCAGGTGTATCCTCGATTCTAGGGGCTATTAACTTTATTACAACGATTATTA
ATATGAAGCCCCCAGCAATTTCTCAGTACCAGACACCTTTATTCGTATGGGCCGTCTTAATTACTGCTGTCCTCCTACTACTCTCAC
TCCCTGTCCTAGCTGCCGGCATTACAATGCTTCTAACAG

No.2025-166-22-34

TGAGCCGGAATAGTGGGGACGGCCCTAAGCCTTTTAATTCGTGCGGAACTTAGCCAACCCGGCGCCCTCCTAGGAGACGACCA
AATATATAATGTTATCGTTACTGCACACGCCTTCGTAATAATCTTTTTTATAGTTATGCCAATTATAATTGGAGGTTTCGGGAACTGA
CTTGTACCTTTAATAATTGGCGCCCCTGACATGGCATTCCCTCGAATAAACAACATGAGCTTCTGACTACTCCCTCCTTCCTTTCTG
CTCCTTCTCGCCTCCTCTGGGGTTGAAGCTGGAGCCGGAACAGGTTGGACCGTATATCCTCCTCTAGCAAGCAACGTCGCCCAC
TCCAGGAGCATCAGTTGACTTAACTATTTTTTCCCTCCACTTAGCGGGTGTTTCATCCATTCTTGGGGCAATTAATTTTATCACAA
CCATCTTTAATATAAAACCTGCCGCTGCTTCAATATATCAAATACCCCTGTTCGTTTGGGCCGTTCTAGTTACAGCAGTACTACTTC
TTCTTTCTCTCCCTGTCT-TAGCCGCTGGCATCACTATACTATTAACGGATCGAA

No.2025-166-22-35

TGAGCCGGAATAGTGGGGACGGCCCTAAGCCTTTTAATTCGTGCGGAACTTAGCCAACCCGGCGCCCTCCTAGGAGACGACCA
AATATATAATGTTATCGTTACTGCACACGCCTTCGTAATAATCTTTTTTATAGTTATGCCAATTATAATTGGAGGTTTCGGGAACTGA
CTTGTACCTTTAATAATTGGCGCCCCTGACATGGCATTCCCTCGAATAAACAACATGAGCTTCTGACTACTCCCTCCTTCCTTTCTG
CTCCTTCTCGCCTCCTCTGGGGTTGAAGCTGGAGCCGGAACAGGTTGGACCGTATATCCTCCTCTAGCAAGCAACGTCGCCCAC
TCCAGGAGCATCAGTTGACTTAACTATTTTTTCCCTCCACTTAGCGGGTGTTTCATCCATTCTTGGGGCAATTAATTTTATCACAA
CCATCTTTAATATAAAACCTGCCGCTGCTTCAATATATCAAATACCCCTGTTCGTTTGGGCCGTTCTAGTTACAGCAGTACTACTTC
TTCTTTCTCTCCCTGTCTTAGCCGCTGGCATCACTATACTATTAACGGATCGA

No.2025-166-22-36

ATAGTGGGGACGGCCCTAAGCCTTTTAATTCGTGCGGAACTTAGCCAACCCGGCGCCCTCCTAGGAGACGACCAAATATATAAT
GTTATCGTTACTGCACACGCCTTCGTAATAATCTTTTTTATAGTTATGCCAATTATAATTGGAGGTTTCGGGAACTGACTTGTACCT
TTAATAATTGGCGCCCCTGACATGGCATTCCCTCGAATAAACAACATGAGCTTCTGACTACTCCCTCCTTCCTTTCTGCTCCTTCTC
GCCTCCTCTGGGGTTGAAGCTGGAGCCGGAACAGGTTGGACCGTATATCCTCCTCTAGCAAGCAACGTCGCCCACTCCAGGAG
CATCAGTTGACTTAACTATTTTTTCCCTCCACTTAGCGGGTGTTTCATCCATTCTTGGGGCAATTAATTTTATCACAACCATCTTTAA
TATAAAACCTGCCGCTGCTTCAATATATCAAATACCCCTGTTCGTTTGGGCCGTTCTAGTTACAGCAGTACTACTTCTTCTTTCTCT
CCCTGTCTTTAGCCGCTGGCATCACTATACTATTAACGGATCGAA

No.2025-166-22-37

TGAGCCGGAATAGTGGGGACGGCCCTAAGCCTTTTAATTCGTGCGGAACTTAGCCAACCCGGCGCCCTCCTAGGAGACGACCA
AATATATAATGTTATCGTTACTGCACACGCCTTCGTAATAATCTTTTTTATAGTTATGCCAATTATAATTGGAGGTTTCGGGAACTGA
CTTGTACCTTTAATAATTGGCGCCCCTGACATGGCATTCCCTCGAATAAACAACATGAGCTTCTGACTACTCCCTCCTTCCTTTCTG
CTCCTTCTCGCCTCCTCTGGGGTTGAAGCTGGAGCCGGAACAGGTTGGACCGTATATCCTCCTCTAGCAAGCAACGTCGCCCAC
TCCAGGAGCATCAGTTGACTTAACTATTTTTTCCCTCCACTTAGCGGGTGTTTCATCCATTCTTGGGGCAATTAATTTTATCACAA
CCATCTTTAATATAAAACCTGCCGCTGCTTCAATATATCAAATACCCCTGTTCGTTTGGGCCGTTCTAGTTACAGCAGTACTACTTC
TTCTTTCTCTCCCTGTCTTAGCCGCTGGCATCACTATACTAT

No.2025-166-22-38

TGAGCCGGAATAGTGGGGACGGCCCTAAGCCTTTTAATTCGTGCGGAACTTAGCCAACCCGGCGCCCTCCTAGGAGACGACCA
AATATATAATGTTATCGTTACTGCACACGCCTTCGTAATAATCTTTTTTATAGTTATGCCAATTATAATTGGAGGTTTCGGGAACTGA
CTTGTACCTTTAATAATTGGCGCCCCTGACATGGCATTCCCTCGAATAAACAACATGAGCTTCTGACTACTCCCTCCTTCCTTTCTG
CTCCTTCTCGCCTCCTCTGGGGTTGAAGCTGGAGCCGGAACAGGTTGGACCGTATATCCTCCTCTAGCAAGCAACGTCGCCCAC
TCCAGGAGCATCAGTTGACTTAACTATTTTTTCCCTCCACTTAGCGGGTGTTTCATCCATTCTTGGGGCAATTAATTTTATCACAA
CCATCTTTAATATAAAACCTGCCGCTGCTTCAATATATCAAATACCCCTGTTCGTTTGGGCCGTTCTAGTTACAGCAGTACTACTTC
TTCTTTCTCTCCCTGTCTTAGCCGCTGGCATCACTATACTATTAACGGATC

No.2025-166-22-39

TGAGCCGGAATAGTGGGGACGGCCCTAAGCCTTTTAATTCGTGCGGAACTTAGCCAACCCGGCGCCCTCCTAGGAGACGACCA
AATATATAATGTTATCGTTACTGCACACGCCTTCGTAATAATCTTTTTTATAGTTATGCCAATTATAATTGGAGGTTTCGGGAACTGA
CTTGTACCTTTAATAATTGGCGCCCCTGACATGGCATTCCCTCGAATAAACAACATGAGCTTCTGACTACTCCCTCCTTCCTTTCTG
CTCCTTCTCGCCTCCTCTGGGGTTGAAGCTGGAGCCGGAACAGGTTGGACCGTATATCCTCCTCTAGCAAGCAACGTCGCCCAC
TCCAGGAGCATCAGTTGACTTAACTATTTTTTCCCTCCACTTAGCGGGTGTTTCATCCATTCTTGGGGCAATTAATTTTATCACAA
CCATCTTTAATATAAAACCTGCCGCTGCTTCAATATATCAAATACCCCTGTTCGTTTGGGCCGTTCTAGTTACAGCAGTACTACTTC
TTCTTTCTCTCCCTGTCTTAGCCGCTGGCATCACTATACTATTAACGGATC

No.2025-166-22-40

TGAGCCGGAATAGTGGGGACGGCCCTAAGCCTTTTAATTCGTGCGGAACTTAGCCAACCCGGCGCCCTCCTAGGAGACGACCA
AATATATAATGTTATCGTTACTGCACACGCCTTCGTAATAATCTTTTTTATAGTTATGCCAATTATAATTGGAGGTTTCGGGAACTGA
CTTGTACCTTTAATAATTGGCGCCCCTGACATGGCATTCCCTCGAATAAACAACATGAGCTTCTGACTACTCCCTCCTTCCTTTCTG
CTCCTTCTCGCCTCCTCTGGGGTTGAAGCTGGAGCCGGAACAGGTTGGACCGTATATCCTCCTCTAGCAAGCAACGTCGCCCAC
TCCAGGAGCATCAGTTGACTTAACTATTTTTTCCCTCCACTTAGCGGGTGTTTCATCCATTCTTGGGGCAATTAATTTTATCACAA
CCATCTTTAATATAAAACCTGCCGCTGCTTCAATATATCAAATACCCCTGTTCGTTTGGGCCGTTCTAGTTACAGCAGTACTACTTC
TTCTTTCTCTCCCTGTCTTAGCCGCTGGCATCACTATACTATTAACGGATCGAA

No.2025-166-22-41

ATAGTGGGGACGGCCCTAAGCCTTTTAATTCGTGCGGAACTTAGCCAACCCGGCGCCCTCCTAGGAGACGACCAAATATATAAT
GTTATCGTTACTGCACACGCCTTCGTAATAATCTTTTTTATAGTTATGCCAATTATAATTGGAGGTTTCGGGAACTGACTTGTACCT
TTAATAATTGGCGCCCCTGACATGGCATTCCCTCGAATAAACAACATGAGCTTCTGACTACTCCCTCCTTCCTTTCTGCTCCTTCTC
GCCTCCTCTGGGGTTGAAGCTGGAGCCGGAACAGGTTGGACCGTATATCCTCCTCTAGCAAGCAACGTCGCCCACTCCAGGAG
CATCAGTTGACTTAACTATTTTTTCCCTCCACTTAGCGGGTGTTTCATCCATTCTTGGGGCAATTAATTTTATCACAACCATCTTTAA
TATAAAACCTGCCGCTGCTTCAATATATCAAATACCCCTGTTCGTTTGGGCCGTTCTAGTTACAGCAGTACTACTTCTTCTTTCTCT
CCCTGTCTTAGCCGCTGGCATCACTATACTATTAACGGATCGAA

No.2025-166-22-42

TGAGCCGGAATAGTGGGGACGGCCCTAAGCCTTTTAATTCGTGCGGAACTTAGCCAACCCGGCGCCCTCCTAGGAGACGACCA
AATATATAATGTTATCGTTACTGCACACGCCTTCGTAATAATCTTTTTTATAGTTATGCCAATTATAATTGGAGGTTTCGGGAACTGA
CTTGTACCTTTAATAATTGGCGCCCCTGACATGGCATTCCCTCGAATAAACAACATGAGCTTCTGACTACTCCCTCCTTCCTTTCTG
CTCCTTCTCGCCTCCTCTGGGGTTGAAGCTGGAGCCGGAACAGGTTGGACCGTATATCCTCCTCTAGCAAGCAACGTCGCCCAC
TCCAGGAGCATCAGTTGACTTAACTATTTTTTCCCTCCACTTAGCGGGTGTTTCATCCATTCTTGGGGCAATTAATTTTATCACAA
CCATCTTTAATATAAAACCTGCCGCTGCTTCAATATATCAAATACCCCTGTTCGTTTGGGCCGTTCTAGTTACAGCAGTACTACTTC
TTCTTTCTCTCCCTGTCTTAGCCGCTGGCATCACTATACTATTAACGGATCGAAACCTAA

No.2025-166-22-43

ATAGTGGGGACGGCCCTAAGCCTTTTAATTCGTGCGGAACTTAGCCAACCCGGCGCCCTCCTAGGAGACGACCAAATATATAAT
GTTATCGTTACTGCACACGCCTTCGTAATAATCTTTTTTATAGTTATGCCAATTATAATTGGAGGTTTCGGGAACTGACTTGTACCT
TTAATAATTGGCGCCCCTGACATGGCATTCCCTCGAATAAACAACATGAGCTTCTGACTACTCCCTCCTTCCTTTCTGCTCCTTCTC
GCCTCCTCTGGGGTTGAAGCTGGAGCCGGAACAGGTTGGACCGTATATCCTCCTCTAGCAAGCAACGTCGCCCACTCCAGGAG
CATCAGTTGACTTAACTATTTTTTCCCTCCACTTAGCGGGTGTTTCATCCATTCTTGGGGCAATTAATTTTATCACAACCATCTTTAA
TATAAAACCTGCCGCTGCTTCAATATATCAAATACCCCTGTTCGTTTGGGCCGTTCTAGTTACAGCAGTACTACTTCTTCTTTCTCT
CCCTGTCTTAGCCGCTGGCATCACTATACTATTAACGGATCGAA

No.2025-166-22-44

TGAGCCGGAATAGTGGGGACGGCCCTAAGCCTTTTAATTCGTGCGGAACTTAGCCAACCCGGCGCCCTCCTAGGAGACGACCA
AATATATAATGTTATCGTTACTGCACACGCCTTCGTAATAATCTTTTTTATAGTTATGCCAATTATAATTGGAGGTTTCGGGAACTGA
CTTGTACCTTTAATAATTGGCGCCCCTGACATGGCATTCCCTCGAATAAACAACATGAGCTTCTGACTACTCCCTCCTTCCTTTCTG
CTCCTTCTCGCCTCCTCTGGGGTTGAAGCTGGAGCCGGAACAGGTTGGACCGTATATCCTCCTCTAGCAAGCAACGTCGCCCAC
TCCAGGAGCATCAGTTGACTTAACTATTTTTTCCCTCCACTTAGCGGGTGTTTCATCCATTCTTGGGGCAATTAATTTTATCACAA
CCATCTTTAATATAAAACCTGCCGCTGCTTCAATATATCAAATACCCCTGTTCGTTTGGGCCGTTCTAGTTACAGCAGTACTACTTC
TTCTTTCTCTCCCTGTCTTAGCCGCTGGCATCACTATAC

No.2025-166-22-45

ATAGTGGGGACGGCCCTAAGCCTTTTAATTCGTGCGGAACTTAGCCAACCCGGCGCCCTCCTAGGAGACGACCAAATATATAAT
GTTATCGTTACTGCACACGCCTTCGTAATAATCTTTTTTATAGTTATGCCAATTATAATTGGAGGTTTCGGGAACTGACTTGTACCT
TTAATAATTGGCGCCCCTGACATGGCATTCCCTCGAATAAACAACATGAGCTTCTGACTACTCCCTCCTTCCTTTCTGCTCCTTCTC
GCCTCCTCTGGGGTTGAAGCTGGAGCCGGAACAGGTTGGACCGTATATCCTCCTCTAGCAAGCAACGTCGCCCACTCCAGGAG
CATCAGTTGACTTAACTATTTTTTCCCTCCACTTAGCGGGTGTTTCATCCATTCTTGGGGCAATTAATTTTATCACAACCATCTTTAA
TATAAAACCTGCCGCTGCTTCAATATATCAAATACCCCTGTTCGTTTGGGCCGTTCTAGTTACAGCAGTACTACTTCTTCTTTCTCT
CCCTGTCTTAGCCGCTGGCATCACTATACTATTAACGGATC

No.2025-166-22-46

TGAGCCGGAATAGTGGGGACGGCCCTAAGCCTTTTAATTCGTGCGGAACTTAGCCAACCCGGCGCCCTCCTAGGAGACGACCA
AATATATAATGTTATCGTTACTGCACACGCCTTCGTAATAATCTTTTTTATAGTTATGCCAATTATAATTGGAGGTTTCGGGAACTGA
CTTGTACCTTTAATAATTGGCGCCCCTGACATGGCATTCCCTCGAATAAACAACATGAGCTTCTGACTACTCCCTCCTTCCTTTCTG
CTCCTTCTCGCCTCCTCTGGGGTTGAAGCTGGAGCCGGAACAGGTTGGACCGTATATCCTCCTCTAGCAAGCAACGTCGCCCAC
TCCAGGAGCATCAGTTGACTTAACTATTTTTTCCCTCCACTTAGCGGGTGTTTCATCCATTCTTGGGGCAATTAATTTTATCACAA
CCATCTTTAATATAAAACCTGCCGCTGCTTCAATATATCAAATACCCCTGTTCGTTTGGGCCGTTCTAGTTACAGCAGTACTACTTC
TTCTTTCTCTCCCTGTCTTAGCCGCTGGCATCACTATACTAT

No.2025-166-22-47

TGAGCCGGAATAGTGGGGACGGCCCTAAGCCTTTTAATTCGTGCGGAACTTAGCCAACCCGGCGCCCTCCTAGGAGACGACCA
AATATATAATGTTATCGTTACTGCACACGCCTTCGTAATAATCTTTTTTATAGTTATGCCAATTATAATTGGAGGTTTCGGGAACTGA
CTTGTACCTTTAATAATTGGCGCCCCTGACATGGCATTCCCTCGAATAAACAACATGAGCTTCTGACTACTCCCTCCTTCCTTTCTG
CTCCTTCTCGCCTCCTCTGGGGTTGAAGCTGGAGCCGGAACAGGTTGGACCGTATATCCTCCTCTAGCAAGCAACGTCGCCCAC
TCCAGGAGCATCAGTTGACTTAACTATTTTTTCCCTCCACTTAGCGGGTGTTTCATCCATTCTTGGGGCAATTAATTTTATCACAA
CCATCTTTAATATAAAACCTGCCGCTGCTTCAATATATCAAATACCCCTGTTCGTTTGGGCCGTTCTAGTTACAGCAGTACTACTTC
TTCTTTCTCTCCCTGTCTTAGCCGCTGGCATCACTATACTATTAACGGATC

No.2025-166-22-48

TTTTGGGGCATGAGCCGGAATAGTGGGGACGGCCCTAAGCCTTTTAATTCGTGCGGAACTTAGCCAACCCGGCGCCCTCCTAG
GAGACGACCAAATATATAATGTTATCGTTACTGCACACGCCTTCGTAATAATCTTTTTTATAGTTATGCCAATTATAATTGGAGGTTT
CGGGAACTGACTTGTACCTTTAATAATTGGCGCCCCTGACATGGCATTCCCTCGAATAAACAACATGAGCTTCTGACTACTCCCTC
CTTCCTTTCTGCTCCTTCTCGCCTCCTCTGGGGTTGAAGCTGGAGCCGGAACAGGTTGGACCGTATATCCTCCTCTAGCAAGCAA
CGTCGCCCACTCCAGGAGCATCAGTTGACTTAACTATTTTTTCCCTCCACTTAGCGGGTGTTTCATCCATTCTTGGGGCAATTAAT
TTTATCACAACCATCTTTAATATAAAACCTGCCGCTGCTTCAATATATCAAATACCCCTGTTCGTTTGGGCCGTTCTAGTTACAGCA
GTACTACTTCTTCTTTCTCTCCCTGTCTTAGCCGCTGGCATCACTATACTATTAACGGATCGAAACCTAAATACTGCCTTCTTTGACC
CTGCAGGCGGCGGAGACCCGATCCTCTACCAACACCTA

No.2025-166-22-49

TGAGCCGGAATAGTGGGGACGGCCCTAAGCCTTTTAATTCGTGCGGAACTTAGCCAACCCGGCGCCCTCCTAGGAGACGACCA
AATATATAATGTTATCGTTACTGCACACGCCTTCGTAATAATCTTTTTTATAGTTATGCCAATTATAATTGGAGGTTTCGGGAACTGA
CTTGTACCTTTAATAATTGGCGCCCCTGACATGGCATTCCCTCGAATAAACAACATGAGCTTCTGACTACTCCCTCCTTCCTTTCTG
CTCCTTCTCGCCTCCTCTGGGGTTGAAGCTGGAGCCGGAACAGGTTGGACCGTATATCCTCCTCTAGCAAGCAACGTCGCCCAC
TCCAGGAGCATCAGTTGACTTAACTATTTTTTCCCTCCACTTAGCGGGTGTTTCATCCATTCTTGGGGCAATTAATTTTATCACAA
CCATCTTTAATATAAAACCTGCCGCTGCTTCAATATATCAAATACCCCTGTTCGTTTGGGCCGTTCTAGTTACAGCAGTACTACTTC
TTCTTTCTCTCCCTGTCTTAGCCGCTGGCATCACTATACTATTAACG

No.2025-166-22-50

TGAGCCGGAATAGTGGGGACGGCCCTAAGCCTTTTAATTCGTGCGGAACTTAGCCAACCCGGCGCCCTCCTAGGAGACGACCA
AATATATAATGTTATCGTTACTGCACACGCCTTCGTAATAATCTTTTTTATAGTTATGCCAATTATAATTGGAGGTTTCGGGAACTGA
CTTGTACCTTTAATAATTGGCGCCCCTGACATGGCATTCCCTCGAATAAACAACATGAGCTTCTGACTACTCCCTCCTTCCTTTCTG
CTCCTTCTCGCCTCCTCTGGGGTTGAAGCTGGAGCCGGAACAGGTTGGACCGTATATCCTCCTCTAGCAAGCAACGTCGCCCAC
TCCAGGAGCATCAGTTGACTTAACTATTTTTTCCCTCCACTTAGCGGGTGTTTCATCCATTCTTGGGGCAATTAATTTTATCACAA
CCATCTTTAATATAAAACCTGCCGCTGCTTCAATATATCAAATACCCCTGTTCGTTTGGGCCGTTCTAGTTACAGCAGTACTACTTC
TTCTTTCTCTCCCTGTCTTAGCCGCTGGCATCACTATACTATTAACGGATC

No.2025-166-22-51

TGAGCCGGAATAGTGGGGACGGCCCTAAGCCTTTTAATTCGTGCGGAACTTAGCCAACCCGGCGCCCTCCTAGGAGACGACCA
AATATATAATGTTATCGTTACTGCACACGCCTTCGTAATAATCTTTTTTATAGTTATGCCAATTATAATTGGAGGTTTCGGGAACTGA
CTTGTACCTTTAATAATTGGCGCCCCTGACATGGCATTCCCTCGAATAAACAACATGAGCTTCTGACTACTCCCTCCTTCCTTTCTG
CTCCTTCTCGCCTCCTCTGGGGTTGAAGCTGGAGCCGGAACAGGTTGGACCGTATATCCTCCTCTAGCAAGCAACGTCGCCCAC
TCCAGGAGCATCAGTTGACTTAACTATTTTTTCCCTCCACTTAGCGGGTGTTTCATCCATTCTTGGGGCAATTAATTTTATCACAA
CCATCTTTAATATAAAACCTGCCGCTGCTTCAATATATCAAATACCCCTGTTCGTTTGGGCCGTTCTAGTTACAGCAGTACTACTTC
TTCTTTCTCTCCCTGTCTTAGCCGCTGGCATCACTATACTATTAACG

No.2025-166-22-52

TGAGCCGGAATAGTGGGGACGGCCCTAAGCCTTTTAATTCGTGCGGAACTTAGCCAACCCGGCGCCCTCCTAGGAGACGACCA
AATATATAATGTTATCGTTACTGCACACGCCTTCGTAATAATCTTTTTTATAGTTATGCCAATTATAATTGGAGGTTTCGGGAACTGA
CTTGTACCTTTAATAATTGGCGCCCCTGACATGGCATTCCCTCGAATAAACAACATGAGCTTCTGACTACTCCCTCCTTCCTTTCTG
CTCCTTCTCGCCTCCTCTGGGGTTGAAGCTGGAGCCGGAACAGGTTGGACCGTATATCCTCCTCTAGCAAGCAACGTCGCCCAC
TCCAGGAGCATCAGTTGACTTAACTATTTTTTCCCTCCACTTAGCGGGTGTTTCATCCATTCTTGGGGCAATTAATTTTATCACAA
CCATCTTTAATATAAAACCTGCCGCTGCTTCAATATATCAAATACCCCTGTTCGTTTGGGCCGTTCTAGTTACAGCAGTACTACTTC
TTCTTTCTCTCCCTGTCTTAGCCGCTGGCATCACTATACTAT

No.2025-166-22-53

TGAGCCGGAATAGTGGGGACGGCCCTAAGCCTTTTAATTCGTGCGGAACTTAGCCAACCCGGCGCCCTCCTAGGAGACGACCA
AATATATAATGTTATCGTTACTGCACACGCCTTCGTAATAATCTTTTTTATAGTTATGCCAATTATAATTGGAGGTTTCGGGAACTGA
CTTGTACCTTTAATAATTGGCGCCCCTGACATGGCATTCCCTCGAATAAACAACATGAGCTTCTGACTACTCCCTCCTTCCTTTCTG
CTCCTTCTCGCCTCCTCTGGGGTTGAAGCTGGAGCCGGAACAGGTTGGACCGTATATCCTCCTCTAGCAAGCAACGTCGCCCAC
TCCAGGAGCATCAGTTGACTTAACTATTTTTTCCCTCCACTTAGCGGGTGTTTCATCCATTCTTGGGGCAATTAATTTTATCACAA
CCATCTTTAATATAAAACCTGCCGCTGCTTCAATATATCAAATACCCCTGTTCGTTTGGGCCGTTCTAGTTACAGCAGTACTACTTC
TTCTTTCTCTCCCTGTCTTAGCCGCTGGCATCACTATACTAT

No.2025-166-22-54

TAGTAGGCACAGCCTTAAGCCTCCTAATCCGAGCAGAACTAAGTCAGCCAGGCTCACTCCTCGGAGATGACCAGATTTTTAACG
TAATTGTTACGGCACATGCCTTTGTCATGATTTTCTTTATAGTAATACCCGTCATGATTGGAGGGTTCGGAAACTGACTTGTACCC
CTAATGATCGGAGCCCCCGACATGGCCTTTCCCCGGATGAACAACATAAGCTTCTGGCTTCTCCCCCCTTCTTTCCTGCTACTCCT
CACTTCTTCCGGAGTAGAGGCAGGTGCCGGGACAGGGTGAACAGTCTACCCCCCGCTCGCTGGTAATCTCGCACACGCAGGT
GCTTCTGTCGACCTGGCTATCTTTTCCCTCCATCTCGCAGGTGTATCCTCGATTCTAGGGGCTATTAACTTTATTACAACGATTATTA
ATATGAAGCCCCCAGCAATTTCTCAGTACCAGACACCTTTATTCGTATGGGCCGTCTTAATTACTGCTGTCCTCCTACTACTCTCAC
TCCCTGTCCTAGCTGCCGGCATTACAATGCTTCTAACAG

No.2025-166-22-55

ATAGTAGGCACAGCCTTAAGCCTCCTAATCCGAGCAGAACTAAGTCAGCCAGGCTCACTCCTCGGAGATGACCAGATTTTTAAC
GTAATTGTTACGGCACATGCCTTTGTCATGATTTTCTTTATAGTAATACCCGTCATGATTGGAGGGTTCGGAAACTGACTTGTACC
CCTAATGATCGGAGCCCCCGACATGGCCTTTCCCCGGATGAACAACATAAGCTTCTGGCTTCTCCCCCCTTCTTTCCTGCTACTCC
TCACTTCTTCCGGAGTAGAGGCAGGTGCCGGGACAGGGTGAACAGTCTACCCCCCGCTCGCTGGTAACCTCGCACACGCAGGT
GCTTCTGTCGACCTGGCTATCTTTTCCCTCCATCTCGCAGGTGTATCCTCGATTCTAGGGGCTATTAACTTTATTACAACGATTATTA
ATATGAAGCCCCCAGCAATTTCTCAGTACCAGACACCTTTATTCGTATGGGCCGTCTTAATTACTGCTGTCCTCCTACTACTCTCAC
TCCCTGTCCTAGCTGCCGGCATTACAATGCTTCTAACAGACC

No.2025-166-22-56

TGAGCCGGAATAGTGGGGACGGCCCTAAGCCTTTTAATTCGTGCGGAACTTAGCCAACCAGGCGCCCTCCTAGGAGACGACCA
AATATATAATGTTATCGTTACTGCACACGCCTTCGTAATAATCTTTTTTATAGTTATGCCAATTATAATTGGAGGTTTCGGGAACTGA
CTTGTACCTTTAATAATTGGCGCCCCTGACATGGCATTCCCTCGAATAAACAACATGAGCTTCTGACTACTCCCTCCTTCCTTTCTG
CTCCTTCTCGCCTCCTCTGGGGTTGAAGCTGGAGCCGGAACAGGTTGGACCGTATATCCTCCTCTAGCAAGCAACGTCGCCCAC
TCCAGGAGCATCAGTTGACTTAACTATTTTTTCCCTCCACTTAGCGGGTGTTTCATCCATTCTTGGGGCAATTAATTTTATCACAA
CCATCTTTAATATAAAACCTGCCGCTGCTTCAATATATCAAATACCCCTGTTCGTTTGGGCCGTTCTAGTTACAGCAGTACTACTTC
TTCTTTCTCTCCCTGTCTTAGCCGCTGGCATCACTATACTATTAACGGATCGAA

No.2025-166-22-57

ATGGGCCGGAATAGTAGGCACAGCCTTAAGCCTCCTAATCCGAGCAGAACTAAGTCAGCCAGGCTCACTCCTCGGAGATGACC
AGATTTTTAACGTAATTGTTACGGCACATGCCTTTGTCATGATTTTCTTTATAGTAATACCCGTCATGATTGGAGGGTTCGGAAAC
TGACTTGTACCCCTAATGATCGGAGCCCCCGACATGGCCTTTCCCCGGATGAACAACATAAGCTTCTGGCTTCTCCCCCCTTCTTT
CCTGCTACTCCTCACTTCTTCCGGAGTAGAGGCAGGTGCCGGGACAGGGTGAACAGTCTACCCCCCGCTCGCTGGTAACCTCG
CACACGCAGGTGCTTCTGTCGACCTGGCTATCTTTTCCCTCCATCTCGCAGGTGTATCCTCGATTCTAGGGGCTATTAACTTTATTA
CAACGATTATTAATATGAAGCCCCCAGCAATTTCTCAGTACCAGACACCTTTATTCGTATGGGCCGTCTTAATTACTGCTGTCCTCC
TACTACTCTCACTCCCTGTCCTAGCTGCCGGCATTACAATGCTTCTAACAG

No.2025-166-22-58

GGAGATGACCAGATTTTTAACGTAATTGTTACGGCACATGCCTTTGTCATGATTTTCTTTATAGTAATACCCGTCATGATTGGAGG
GTTCGGAAACTGACTTGTACCCCTAATGATCGGAGCCCCCGACATGGCCTTTCCCCGGATGAACAACATAAGCTTCTGGCTTCTC
CCCCCTTCTTTCCTGCTACTCCTCACTTCTTCCGGAGTAGAGGCAGGTGCCGGGACAGGGTGAACAGTCTACCCCCCGCTCGCT
GGTAACCTCGCACACGCAGGTGCTTCTGTCGACCTGGCTATCTTTTCCCTCCATCTCGCAGGTGTATCCTCGATTCTAGGGGCTAT
TAACTTTATTACAACGATTATTAATATGAAGCCCCCAGCAATTTCTCAGTACCAGACACCTTTATTCGTATGGGCCGTCTTAATTACT
GCTGTCCTCCTACTACTCTCACTCCCTGTCCTAGCTGCCGGCATTACAATGCTTCTAACAGACCGCAACCTCAACACAACCTTCTT
T

No.2025-166-22-59

GGAATAGTAGGCACAGCCTTAAGCCTCCTAATCCGAGCAGAACTAAGTCAGCCAGGCTCACTCCTCGGAGATGACCAGATTTTT
AACGTAATTGTTACGGCACATGCCTTTGTCATGATTTTCTTTATAGTAATACCCGTCATGATTGGAGGGTTCGGAAACTGACTTGT
ACCCCTAATGATCGGAGCCCCCGACATGGCCTTTCCCCGGATGAACAACATAAGCTTCTGGCTTCTCCCCCCTTCTTTCCTGCTAC
TCCTCACTTCTTCCGGAGTAGAGGCAGGTGCCGGGACAGGGTGAACAGTCTACCCCCCGCTCGCTGGTAACCTCGCACACGCA
GGTGCTTCTGTCGACCTGGCTATCTTTTCCCTCCATCTCGCAGGTGTATCCTCGATTCTAGGGGCTATTAACTTTATTACAACGATT
ATTAATATGAAGCCCCCAGCAATTTCTCAGTACCAGACACCTTTATTCGTATGGGCCGTCTTAATTACTGCTGTCCTCCTACTACTC
TCACTCCCTGTCCTAGCTGCCGGCATTACAATGCTTCTAACAG

No.2025-166-22-60

GCCTCCTAATCCGAGCAGAACTAAGTCAGCCAGGCTCACTCCTCGGAGATGACCAGATTTTTAACGTAATTGTTACGGCACATG
CCTTTGTCATGATTTTCTTTATAGTAATACCCGTCATGATTGGAGGGTTCGGAAACTGACTTGTGCCCCTAATGATCGGAGCCCCC
GACATGGCCTTTCCCCGGATGAACAACATAAGCTTCTGGCTTCTCCCCCCTTCTTTCCTGCTACTCCTCACTTCTTCCGGAGTAGA
GGCAGGTGCCGGGACAGGGTGAACAGTCTACCCCCCGCTCGCTGGTAATCTCGCACACGCAGGTGCTTCTGTCGACCTGGCTA
TCTTTTCCCTCCATCTCGCAGGTGTGTCCTCGATTCTAGGGGCTATTAACTTTATTACAACGATTATTAATATGAAGCCCCCAGCAA
TTTCTCAGTACCAGACACCTTTATTCGTATGGGCCGTCTTAATTACTGCTGTCCTCCTACTACTCTCACTCCCTGTCCTAGCTGCCG
GCATTACAATGCTTCTAACAGACCGCAACCTCAACACAACCTTCTTTGACCTTGCCGAGGAGGAGGTGACCCCATTCTTTACCA
ACACTTATTCTGATTCTTTGACCC

Fish maw: COI gene - Small-sized discoid samples (n=54)

No.2025-166-23-1

ATAGTGGGGACGGCCCTAAGCCTTTTAATTCGTGCGGAACTTAGCCAACCCGGCGCCCTCCTAGGAGACGACCAAATATATAAT
GTTATCGTTACTGCACACGCCTTCGTAATAATCTTTTTTATAGTTATGCCAATTATAATTGGAGGTTTCGGGAACTGACTTGTACCT
TTAATAATTGGCGCCCCTGACATGGCATTCCCTCGAATAAACAACATGAGCTTCTGACTACTCCCTCCTTCCTTTCTGCTCCTTCTC
GCCTCCTCTGGGGTTGAAGCTGGAGCCGGAACAGGTTGGACCGTATATCCTCCTCTAGCAAGCAACGTCGCCCACTCCAGGAG
CATCAGTTGACTTAACTATTTTTTCCCTCCACTTAGCGGGTGTTTCATCCATTCTTGGGGCAATTAATTTTATCACAACCATCTTTAA
TATAAAACCTGCCGCTGCTTCAATATATCAAATACCCCTGTTCGTTTGGGCCGTTCTAGTTACAGCAGTACTACTTCTTCTTTCTCT
CCCTGTCTTAGCCGCTGGCATCACTATACTAT

No.2025-166-23-2

TGAGCCGGAATAGTGGGGACGGCCCTAAGCCTTTTAATTCGTGCGGAACTTAGCCAACCCGGCGCCCTCCTAGGAGACGACCA
AATATATAATGTTATCGTTACTGCACACGCCTTCGTAATAATCTTTTTTATAGTTATGCCAATTATAATTGGAGGTTTCGGGAACTGA
CTTGTACCTTTAATAATTGGCGCCCCTGACATGGCATTCCCTCGAATAAACAACATGAGCTTCTGACTACTCCCTCCTTCCTTTCTG
CTCCTTCTCGCCTCCTCTGGGGTTGAAGCTGGAGCCGGAACAGGTTGGACCGTATATCCTCCTCTAGCAAGCAACGTCGCCCAC
TCCAGGAGCATCAGTTGACTTAACTATTTTTTCCCTCCACTTAGCGGGTGTTTCATCCATTCTTGGGGCAATTAATTTTATCACAA
CCATCTTTAATATAAAACCTGCCGCTGCTTCAATATATCAAATACCCCTGTTCGTTTGGGCCGTTCTAGTTACAGCAGTACTACTTC
TTCTTTCTCTCCCTGTCTTAGCCGCTGGCATCACTATACTATTAACG

No.2025-166-23-3

TGAGCCGGAATAGTGGGGACGGCCCTAAGCCTTTTAATTCGTGCGGAACTTAGCCAACCCGGCGCCCTCCTAGGAGACGACCA
AATATATAATGTTATCGTTACTGCACACGCCTTCGTAATAATCTTTTTTATAGTTATGCCAATTATAATTGGAGGTTTCGGGAACTGA
CTTGTACCTTTAATAATTGGCGCCCCTGACATGGCATTCCCTCGAATAAACAACATGAGCTTCTGACTACTCCCTCCTTCCTTTCTG
CTCCTTCTCGCCTCCTCTGGGGTTGAAGCTGGAGCCGGAACAGGTTGGACCGTATATCCTCCTTTAGCAAGCAACGTCGCCCAC
TCCAGGAGCATCAGTTGACTTAACTATTTTTTCCCTCCACTTAGCGGGTGTTTCATCCATTCTTGGGGCAATTAATTTTATCACAA
CCATCTTTAATATAAAACCTGCCGCTGCTTCAATATATCAAATACCCCTGTTCGTTTGGGCCGTTCTAGTTACAGCAGTACTACTTC
TTCTTTCTCTCCCTGTCTTAGCCGCTGGCATCACTATACTAT

No.2025-166-23-4

TTTTTGGGGCATGAGCCGGAATAGTGGGGACGGCCCTAAGCCTTTTAATTCGTGCGGAACTTAGCCAACCCGGCGCCCTCCTA
GGAGACGACCAAATATATAATGTTATCGTTACTGCACACGCCTTCGTAATAATCTTTTTTATAGTTATGCCAATTATAATTGGAGGT
TTCGGGAACTGACTTGTACCTTTAATAATTGGCGCCCCTGACATGGCATTCCCTCGAATAAACAACATGAGCTTCTGACTACTCCC
TCCTTCCTTTCTGCTCCTTCTCGCCTCCTCTGGGGTTGAAGCTGGAGCCGGAACAGGTTGGACCGTATATCCTCCTCTAGCAAGC
AACGTCGCCCACTCCAGGAGCATCAGTTGACTTAACTATTTTTTCCCTCCACTTAGCGGGTGTTTCATCCATTCTTGGGGCAATTA
ATTTTATCACAACCATCTTTAATATAAAACCTGCCGCTGCTTCAATATATCAAATACCCCTGTTCGTTTGGGCCGTTCTAGTTACAG
CAGTACTACTTCTTCTTTCTCTCCCTGTCTTAGCCGCTGGCATCACTATACTAT

No.2025-166-23-5

TGAGCCGGAATAGTGGGGACGGCCCTAAGCCTTTTAATTCGTGCGGAACTTAGCCAACCCGGCGCCCTCCTAGGAGACGACCA
AATATATAATGTTATCGTTACTGCACACGCCTTCGTAATAATCTTTTTTATAGTTATGCCAATTATAATTGGAGGTTTCGGGAACTGA
CTTGTACCTTTAATAATTGGCGCCCCTGACATGGCATTCCCTCGAATAAACAACATGAGCTTCTGACTACTCCCTCCTTCCTTTCTG
CTCCTTCTCGCCTCCTCTGGGGTTGAAGCTGGAGCCGGAACAGGTTGGACCGTATATCCTCCTCTAGCAAGCAACGTCGCCCAC
TCCAGGAGCATCAGTTGACTTAACTATTTTTTCCCTCCACTTAGCGGGTGTTTCATCCATTCTTGGGGCAATTAATTTTATCACAA
CCATCTTTAATATAAAACCTGCCGCTGCTTCAATATATCAAATACCCCTGTTCGTTTGGGCCGTTCTAGTTACAGCAGTACTACTTC
TTCTTTCTCTCCCTGTCTTAGCCGCTGGCATCACTATACTAT

No.2025-166-23-6

TGAGCCGGAATAGTGGGGACGGCCCTAAGCCTTTTAATTCGTGCGGAACTTAGCCAACCCGGCGCCCTCCTAGGAGACGACCA
AATATATAATGTTATCGTTACTGCACACGCCTTCGTAATAATCTTTTTTATAGTTATGCCAATTATAATTGGAGGTTTCGGGAACTGA
CTTGTACCTTTAATAATTGGCGCCCCTGACATGGCATTCCCTCGAATAAACAACATGAGCTTCTGACTACTCCCTCCTTCCTTTCTG
CTCCTTCTCGCCTCCTCTGGGGTTGAAGCTGGAGCCGGAACAGGTTGGACCGTATATCCTCCTTTAGCAAGCAACGTCGCCCAC
TCCAGGAGCATCAGTTGACTTAACTATTTTTTCCCTCCACTTAGCGGGTGTTTCATCCATTCTTGGGGCAATTAATTTTATCACAA
CCATCTTTAATATAAAACCTGCCGCTGCTTCAATATATCAAATACCCCTGTTCGTTTGGGCCGTTCTAGTTACAGCAGTACTACTTC
TTCTTTCTCTCCCTGTCTTAGCCGCTGGCATCACTATACTATTAACG

No.2025-166-23-7

ATAGTGGGGACGGCCCTAAGCCTTTTAATTCGTGCGGAACTTAGCCAACCCGGCGCCCTCCTAGGAGACGACCAAATATATAAT
GTTATCGTTACTGCACACGCCTTCGTAATAATCTTTTTTATAGTTATGCCAATTATAATTGGAGGTTTCGGGAACTGACTTGTACCT
TTAATAATTGGCGCCCCTGACATGGCATTCCCTCGAATAAACAACATGAGCTTCTGACTACTCCCTCCTTCCTTTCTGCTCCTTCTC
GCCTCCTCTGGGGTTGAAGCTGGAGCCGGAACAGGTTGGACCGTATATCCTCCTCTAGCAAGCAACGTCGCCCACTCCAGGAG
CATCAGTTGACTTAACTATTTTTTCCCTCCACTTAGCAGGTGTTTCATCCATTCTTGGGGCAATTAATTTTATCACAACCATCTTTAA
TATAAAACCTGCCGCTGCTTCAATATATCAAATACCCCTGTTCGTTTGGGCCGTTCTAGTTACAGCAGTACTACTTCTTCTTTCTCT
CCCTGTCTTAGCCGCTGGCATCACTATACTAT

No.2025-166-23-8

TGAGCCGGAATAGTGGGGACGGCCCTAAGCCTTTTAATTCGTGCGGAACTTAGCCAACCCGGCGCCCTCCTAGGAGACGACCA
AATATATAATGTTATCGTTACTGCACACGCCTTCGTAATAATCTTTTTTATAGTTATGCCAATTATAATTGGAGGTTTCGGGAACTGA
CTTGTACCTTTAATAATTGGCGCCCCTGACATGGCATTCCCTCGAATAAACAACATGAGCTTCTGACTACTCCCTCCTTCCTTTCTG

CTCCTTCTCGCCTCCTCTGGGGTTGAAGCTGGAGCCGGAACAGGTTGGACCGTATATCCTCCTCTAGCAAGCAACGTCGCCCAC
TCCAGGAGCATCAGTTGACTTAACTATTTTTTCCCTCCACTTAGCGGGTGTTTCATCCATTCTTGGGGCAATTAATTTTATCACAA
CCATCTTTAATATAAAACCTGCCGCTGCTTCAATATATCAAATACCCCTGTTCGTTTGGGCCGTTCTAGTTACAGCAGTACTACTTC
TTCTTTCTCTCCCTGTCTTAGCCGCTGGCATCACTATACTAT

No.2025-166-23-9

TGAGCCGGAATAGTGGGGACGGCCCTAAGCCTTTTAATTCGTGCGGAACTTAGCCAACCCGGCGCCCTCCTAGGAGACGACCA
AATATATAATGTTATCGTTACTGCACACGCCTTCGTAATAATCTTTTTTATAGTTATGCCAATTATAATTGGAGGTTTCGGGAACTGA
CTTGTACCTTTAATAATTGGCGCCCCTGACATGGCATTCCCTCGAATAAACAACATGAGCTTCTGACTACTCCCTCCTTCCTTTCTG
CTCCTTCTCGCCTCCTCTGGGGTTGAAGCTGGAGCCGGAACAGGTTGGACCGTATATCCTCCTCTAGCAAGCAACGTCGCCCAC
TCCAGGAGCATCAGTTGACTTAACTATTTTTTCCCTCCACTTAGCGGGTGTTTCATCCATTCTTGGGGCAATTAATTTTATCACAA
CCATCTTTAATATAAAACCTGCCGCTGCTTCAATATATCAAATACCCCTGTTCGTTTGGGCCGTTCTAGTTACAGCAGTACTACTTC
TTCTTTCTCTCCCTGTCTTAGCCGCTGGCATCACTATACTAT

No.2025-166-23-10

TGGGGCATGAGCCGGAATAGTGGGGACGGCCCTAAGCCTTTTAATTCGTGCGGAACTTAGCCAACCCGGCGCCCTCCTAGGAG
ACGACCAAATATATAATGTTATCGTTACTGCACACGCCTTCGTAATAATCTTTTTTATAGTTATGCCAATTATAATTGGAGGTTTCGG
GAACTGACTTGTACCTTTAATAATTGGCGCCCCTGACATGGCATTCCCTCGAATAAACAACATGAGCTTCTGACTACTCCCTCCTT
CCTTTCTGCTCCTTCTCGCCTCCTCTGGGGTTGAAGCTGGAGCCGGAACAGGTTGGACCGTATATCCTCCTCTAGCAAGCAACG
TCGCCCACTCCAGGAGCATCAGTTGACTTAACTATTTTTTCCCTCCACTTAGCGGGTGTTTCATCCATTCTTGGGGCAATTAATTT
TATCACAACCATCTTTAATATAAAACCTGCCGCTGCTTCAATATATCAAATACCCCTGTTCGTTTGGGCCGTTCTAGTTACAGCAGT
ACTACTTCTTCTTTCTCTCCCTGTCTTAGCCGCTGGCATCACTATACTAT

No.2025-166-23-11

ATAGTGGGGACGGCCCTAAGCCTTTTAATTCGTGCGGAACTTAGCCAACCCGGCGCCCTCCTAGGAGACGACCAAATATATAAT
GTTATCGTTACTGCACACGCCTTCGTAATAATCTTTTTTATAGTTATGCCAATTATAATTGGAGGTTTCGGGAACTGACTTGTACCT
TTAATAATTGGCGCCCCTGACATGGCATTCCCTCGAATAAACAACATGAGCTTCTGACTACTCCCTCCTTCCTTTCTGCTCCTTCTC
GCCTCCTCTGGGGTTGAAGCTGGAGCCGGAACAGGTTGGACCGTATATCCTCCTCTAGCAAGCAACGTCGCCCACTCCAGGAG
CATCAGTTGACTTAACTATTTTTTCCCTCCACTTAGCGGGTGTTTCATCCATTCTTGGGGCAATTAATTTTATCACAACCATCTTTAA
TATAAAACCTGCCGCTGCTTCAATATATCAAATACCCCTGTTCGTTTGGGCCGTTCTAGTTACAGCAGTACTACTTCTTCTTTCTCT
CCCTGTCTTAGCCGCTGGCATCACTATACTAT

No.2025-166-23-12

ATAGTGGGGACGGCCCTAAGCCTTTTAATTCGTGCGGAACTTAGCCAACCCGGCGCCCTCCTAGGAGACGACCAAATATATAAT
GTTATCGTTACTGCACACGCCTTCGTAATAATCTTTTTTATAGTTATGCCAATTATAATTGGAGGTTTCGGGAACTGACTTGTACCT
TTAATAATTGGCGCCCCTGACATGGCATTCCCTCGAATAAACAACATGAGCTTCTGACTACTCCCTCCTTCCTTTCTGCTCCTTCTC
GCCTCCTCTGGGGTTGAAGCTGGAGCCGGAACAGGTTGGACCGTATATCCTCCTCTAGCAAGCAACGTCGCCCACTCCAGGAG
CATCAGTTGACTTAACTATTTTTTCCCTCCACTTAGCGGGTGTTTCATCCATTCTTGGGGCAATTAATTTTATCACAACCATCTTTAA
TATAAAACCTGCCGCTGCTTCAATATATCAAATACCCCTGTTCGTTTGGGCCGTTCTAGTTACAGCAGTACTACTTCTTCTTTCTCT
CCCTGTCTTAGCCGCTGGCATCACTATACTAT

No.2025-166-23-13

TGAGCCGGAATAGTGGGGACGGCCCTAAGCCTTTTAATTCGTGCGGAACTTAGCCAACCCGGCGCCCTCCTAGGAGACGACCA
AATATATAATGTTATCGTTACTGCACACGCCTTCGTAATAATCTTTTTTATAGTTATGCCAATTATAATTGGAGGTTTCGGGAACTGA
CTTGTACCTTTAATAATTGGCGCCCCTGACATGGCATTCCCTCGAATAAACAACATGAGCTTCTGACTACTCCCTCCTTCCTTTCTG
CTCCTTCTCGCCTCCTCTGGGGTTGAAGCTGGAGCCGGAACAGGTTGGACCGTATATCCTCCTCTAGCAAGCAACGTCGCCCAC
TCCAGGAGCATCAGTTGACTTAACTATTTTTTCCCTCCACTTAGCGGGTGTTTCATCCATTCTTGGGGCAATTAATTTTATCACAA
CCATCTTTAATATAAAACCTGCCGCTGCTTCAATATATCAAATACCCCTGTTCGTTTGGGCCGTTCTAGTTACAGCAGTACTACTTC
TTCTTTCTCTCCCTGTCTTAGCCGCTGGCATCACTATACTAT

No.2025-166-23-14

TGAGCCGGAATAGTGGGGACGGCCCTAAGCCTTTTAATTCGTGCGGAACTTAGCCAACCCGGCGCCCTCCTAGGAGACGACCA
AATATATAATGTTATCGTTACTGCACACGCCTTCGTAATAATCTTTTTTATAGTTATGCCAATTATAATTGGAGGTTTCGGGAACTGA
CTTGTACCTTTAATAATTGGCGCCCCTGACATGGCATTCCCTCGAATAAACAACATGAGCTTCTGACTACTCCCTCCTTCCTTTCTG
CTCCTTCTCGCCTCCTCTGGGGTTGAAGCTGGAGCCGGAACAGGTTGGACCGTATATCCTCCTCTAGCAAGCAACGTCGCCCAC
TCCAGGAGCATCAGTTGACTTAACTATTTTTTCCCTCCACTTAGCGGGTGTTTCATCCATTCTTGGGGCAATTAATTTTATCACAA
CCATCTTTAATATAAAACCTGCCGCTGCTTCAATATATCAAATACCCCTGTTCGTTTGGGCCGTTCTAGTTACAGCAGTACTACTTC
TTCTTTCTCTCCCTGTCTTAGCCGCTGGCATCACTATACTAT

No.2025-166-23-15

ATAGTGGGGACGGCCCTAAGCCTTTTAATTCGTGCGGAACTTAGCCAACCCGGCGCCCTCCTAGGAGACGACCAAATATATAAT
GTTATCGTTACTGCACACGCCTTCGTAATAATCTTTTTTATAGTTATGCCAATTATAATTGGAGGTTTCGGGAACTGACTTGTACCT
TTAATAATTGGCGCCCCTGACATGGCATTCCCTCGAATAAACAACATGAGCTTCTGACTACTCCCTCCTTCCTTTCTGCTCCTTCTC
GCCTCCTCTGGGGTTGAAGCTGGAGCCGGAACAGGTTGGACCGTATATCCTCCTCTAGCAAGCAACGTCGCCCACTCCAGGAG
CATCAGTTGACTTAACTATTTTTTCCCTCCACTTAGCGGGTGTTTCATCCATTCTTGGGGCAATTAATTTTATCACAACCATCTTTAA
TATAAAACCTGCCGCTGCTTCAATATATCAAATACCCCTGTTCGTTTGGGCCGTTCTAGTTACAGCAGTACTACTTCTTCTTTCTCT
CCCTGTCTTAGCCGCTGGCATCACTATACTATTAACGGATCGAAACCTAAATACTGCCTTC

No.2025-166-23-16

TGAGCCGGAATAGTGGGGACGGCCCTAAGCCTTTTAATTCGTGCGGAACTTAGCCAACCCGGCGCCCTCCTAGGAGACGACCA
AATATATAATGTTATCGTTACTGCACACGCCTTCGTAATAATCTTTTTTATAGTTATGCCAATTATAATTGGAGGTTTCGGGAACTGA

CTTGTACCTTTAATAATTGGCGCCCCTGACATGGCATTCCCTCGAATAAACAACATGAGCTTCTGACTACTCCCTCCTTCCTTTCTG
CTCCTTCTCGCCTCCTCTGGGGTTGAAGCTGGAGCCGGAACAGGTTGGACCGTATATCCTCCTCTAGCAAGCAACGTCGCCCAC
TCCAGGAGCATCAGTTGACTTAACTATTTTTTCCCTCCACTTAGCGGGTGTTTCATCCATTCTTGGGGCAATTAATTTTATCACAA
CCATCTTTAATATAAAACCTGCCGCTGCTTCAATATATCAAATACCCCTGTTCGTTTGGGCCGTTCTAGTTACAGCAGTACTACTTC
TTCTTTCTCTCCCTGTCTT-AGCCGCTGGCATCACTATACTAT

No.2025-166-23-17

TGAGCCGGAATAGTGGGGACGGCCCTAAGCCTTTTAATTCGTGCGGAACTTAGCCAACCCGGCGCCCTCCTAGGAGACGACCA
AATATATAATGTTATCGTTACTGCACACGCCTTCGTAATAATCTTTTTTATAGTTATGCCAATTATAATTGGAGGTTTCGGGAACTGA
CTTGTACCTTTAATAATTGGCGCCCCTGACATGGCATTCCCTCGAATAAACAACATGAGCTTCTGACTACTCCCTCCTTCCTTTCTG
CTCCTTCTCGCCTCCTCTGGGGTTGAAGCTGGAGCCGGAACAGGTTGGACCGTATATCCTCCTCTAGCAAGCAACGTCGCCCAC
TCCAGGAGCATCAGTTGACTTAACTATTTTTTCCCTCCACTTAGCGGGTGTTTCATCCATTCTTGGGGCAATTAATTTTATCACAA
CCATCTTTAATATAAAACCTGCCGCTGCTTCAATATATCAAATACCCCTGTTCGTTTGGGCCGTTCTAGTTACAGCAGTACTACTTC
TTCTTTCTCTCCCTGTCT-TAGCCGCTGGCATCACTATACTATTAACGGATCGAA

No.2025-166-23-18

TGAGCCGGAATAGTGGGGACGGCCCTAAGCCTTTTAATTCGTGCGGAACTTAGCCAACCCGGCGCCCTCCTAGGAGACGACCA
AATATATAATGTTATCGTTACTGCACACGCCTTCGTAATAATCTTTTTTATAGTTATGCCAATTATAATTGGAGGTTTCGGGAACTGA
CTTGTACCTTTAATAATTGGCGCCCCTGACATGGCATTCCCTCGAATAAACAACATGAGCTTCTGACTACTCCCTCCTTCCTTTCTG
CTCCTTCTCGCCTCCTCTGGGGTTGAAGCTGGAGCCGGAACAGGTTGGACCGTATATCCTCCTCTAGCAAGCAACGTCGCCCAC
TCCAGGAGCATCAGTTGACTTAACTATTTTTTCCCTCCACTTAGCGGGTGTTTCATCCATTCTTGGGGCAATTAATTTTATCACAA
CCATCTTTAATATAAAACCTGCCGCTGCTTCAATATATCAAATACCCCTGTTCGTTTGGGCCGTTCTAGTTACAGCAGTACTACTTC
TTCTTTCTCTCCCTGTCTTAGCCGCTGGCATCACTATGCTATTAACG

No.2025-166-23-19

ATAGTGGGGACGGCCCTAAGCCTTTTAATTCGTGCGGAACTTAGCCAACCCGGCGCCCTCCTAGGAGACGACCAAATATATAAT
GTTATCGTTACTGCACACGCCTTCGTAATAATCTTTTTTATAGTTATGCCAATTATAATTGGAGGTTTCGGGAACTGACTTGTACCT
TTAATAATTGGCGCCCCTGACATGGCATTCCCTCGAATAAACAACATGAGCTTCTGACTACTCCCTCCTTCCTTTCTGCTCCTTCTC
GCCTCCTCTGGGGTTGAAGCTGGAGCCGGAACAGGTTGGACCGTATATCCTCCTCTAGCAAGCAACGTCGCCCACTCCAGGAG
CATCAGTTGACTTAACTATTTTTTCCCTCCACTTAGCGGGTGTTTCATCCATTCTTGGGGCAATTAATTTTATCACAACCATCTTTAA
TATAAAACCTGCCGCTGCTTCAATATATCAAATACCCCTGTTCGTTTGGGCCGTTCTAGTTACAGCAGTACTACTTCTTCTTTCTCT
CCCTGTCTTAGCCGCTGGCATCACTATACTATTAACG

No.2025-166-23-20

ATAGTGGGGACGGCCCTAAGCCTTTTAATTCGTGCGGAACTTAGCCAACCCGGCGCCCTCCTAGGAGACGACCAAATATATAAT
GTTATCGTTACTGCACACGCCTTCGTAATAATCTTTTTTATAGTTATGCCAATTATAATTGGAGGTTTCGGGAACTGACTTGTACCT
TTAATAATTGGCGCCCCTGACATGGCATTCCCTCGAATAAACAACATGAGCTTCTGACTACTCCCTCCTTCCTTTCTGCTCCTTCTC
GCCTCCTCTGGGGTTGAAGCTGGAGCCGGAACAGGTTGGACCGTATATCCTCCTCTAGCAAGCAACGTCGCCCACTCCAGGAG
CATCAGTTGACTTAACTATTTTTTCCCTCCACTTAGCGGGTGTTTCATCCATTCTTGGGGCAATTAATTTTATCACAACCATCTTTAA
TATAAAACCTGCCGCTGCTTCAATATATCAAATACCCCTGTTCGTTTGGGCCGTTCTAGTTACAGCAGTACTACTTCTTCTTTCTCT
CCCTGTCTTAGCCGCTGGCATCACTATACTATTAACGG

No.2025-166-23-21

TGAGCCGGAATAGTGGGGACGGCCCTAAGCCTTTTAATTCGTGCGGAACTTAGCCAACCCGGCGCCCTCCTAGGAGACGACCA
AATATATAATGTTATCGTTACTGCACACGCCTTCGTAATAATCTTTTTTATAGTTATGCCAATTATAATTGGAGGTTTCGGGAACTGA
CTTGTACCTTTAATAATTGGCGCCCCTGACATGGCATTCCCTCGAATAAACAACATGAGCTTCTGACTACTCCCTCCTTCCTTTCTG
CTCCTTCTCGCCTCCTCTGGGGTTGAAGCTGGAGCCGGAACAGGTTGGACCGTATATCCTCCTCTAGCAAGCAACGTCGCCCAC
TCCAGGAGCATCAGTTGACTTAACTATTTTTTCCCTCCACTTAGCGGGTGTTTCATCCATTCTTGGGGCAATTAATTTTATCACAA
CCATCTTTAATATAAAACCTGCCGCTGCTTCAATATATCAAATACCCCTGTTCGTTTGGGCCGTTCTAGTTACAGCAGTACTACTTC
TTCTTTCTCTCCCTGTCTTAGCCGCTGGCATCACTATACTATTAACGGATCGAAACC

No.2025-166-23-22

TGAGCCGGAATAGTGGGGACGGCCCTAAGCCTTTTAATTCGTGCGGAACTTAGCCAACCCGGCGCCCTCCTAGGAGACGACCA
AATATATAATGTTATCGTTACTGCACACGCCTTCGTAATAATCTTTTTTATAGTTATGCCAATTATAATTGGAGGTTTCGGGAACTGA
CTTGTACCTTTAATAATTGGCGCCCCTGACATGGCATTCCCTCGAATAAACAACATGAGCTTCTGACTACTCCCTCCTTCCTTTCTG
CTCCTTCTCGCCTCCTCTGGGGTTGAAGCTGGAGCCGGAACAGGTTGGACCGTATATCCTCCTCTAGCAAGCAACGTCGCCCAC
TCCAGGAGCATCAGTTGACTTAACTATTTTTTCCCTCCACTTAGCGGGTGTTTCATCCATTCTTGGGGCAATTAATTTTATCACAA
CCATCTTTAATATAAAACCTGCCGCTGCTTCAATATATCAAATACCCCTGTTCGTTTGGGCCGTTCTAGTTACAGCAGTACTACTTC
TTCTTTCTCTCCCTGTCTTAGCCGCTGGCATCACTATACTATTAACGGATCGAA

No.2025-166-23-23

ATAGTGGGGACGGCCCTAAGCCTTTTAATTCGTGCGGAACTTAGCCAACCCGGCGCCCTCCTAGGAGACGACCAAATATATAAT
GTTATCGTTACTGCACACGCCTTCGTAATAATCTTTTTTATAGTTATGCCAATTATAATTGGAGGTTTCGGGAACTGACTTGTACCT
TTAATAATTGGCGCCCCTGACATGGCATTCCCTCGAATAAACAACATGAGCTTCTGACTACTCCCTCCTTCCTTTCTGCTCCTTCTC
GCCTCCTCTGGGGTTGAAGCTGGAGCCGGAACAGGTTGGACCGTATATCCTCCTCTAGCAAGCAACGTCGCCCACTCCAGGAG
CATCAGTTGACTTAACTATTTTTTCCCTCCACTTAGCGGGTGTTTCATCCATTCTTGGGGCAATTAATTTTATCACAACCATCTTTAA
TATAAAACCTGCCGCTGCTTCAATATATCAAATACCCCTGTTCGTTTGGGCCGTTCTAGTTACAGCAGTACTACTTCTTCTTTCTCT
CCCTGTCTTAGCCGCTGGCATCACTATACTATTAACG

No.2025-166-23-24

TGAGCTGGAATAGTGGGGACGGCCCTAAGCCTTTTAATTCGTGCGGAACTTAGCCAACCCGGCGCCCTCCTAGGAGACGACCA

AATATATAATGTTATCGTTACTGCACACGCCTTCGTAATAATCTTTTTTATAGTTATGCCAATTATAATTGGAGGTTTCGGGAACTGA
CTTGTACCTTTAATAATTGGCGCCCCTGACATGGCATTCCCTCGAATAAACAACATGAGCTTCTGACTACTCCCTCCTTCCTTTCTG
CTCCTTCTCGCCTCCTCTGGGGTTGAAGCTGGAGCCGGAACAGGTTGGACCGTATATCCTCCTCTAGCAAGCAACGTCGCCCAC
TCCAGGAGCATCAGTTGACTTAACTATTTTTTCCCTCCACTTAGCGGGTGTTTCATCCATTCTTGGGGCAATTAATTTTATCACAA
CCATCTTTAATATAAAACCTGCCGCTGCTTCAATATATCAAATACCCCTGTTCGTTTGGGCCGTTCTAGTTACAGCAGTACTACTTC
TTCTTTCTCTCCCTGTCTTAGCCGCTGGCATCACTATACTATTAACG

No.2025-166-23-25

TGAGCCGGAATAGTGGGGACGGCCCTAAGCCTTTTAATTCGTGCGGAACTTAGCCAACCCGGCGCCCTCCTAGGAGACGACCA
AATATATAATGTTATCGTTACTGCACACGCCTTCGTAATAATCTTTTTTATAGTTATGCCAATTATAATTGGAGGTTTCGGGAACTGA
CTTGTACCTTTAATAATTGGCGCCCCTGACATGGCATTCCCTCGAATAAACAACATGAGCTTCTGACTACTCCCTCCTTCCTTTCTG
CTCCTTCTCGCCTCCTCTGGGGTTGAAGCTGGAGCCGGAACAGGTTGGACCGTATATCCTCCTCTAGCAAGCAACGTCGCCCAC
TCCAGGAGCATCAGTTGACTTAACTATTTTTTCCCTCCACTTAGCGGGTGTTTCATCCATTCTTGGGGCAATTAATTTTATCACAA
CCATCTTTAATATAAAACCTGCCGCTGCTTCAATATATCAAATACCCCTGTTCGTTTGGGCCGTTCTAGTTACAGCAGTACTACTTC
TTCTTTCTCTCCCTGTCTTAGCCGCTGGCATCACTATACTATTAACG

No.2025-166-23-26

TGAGCCGGAATAGTGGGGACGGCCCTAAGCCTTTTAATTCGTGCGGAACTTAGCCAACCCGGCGCCCTCCTAGGAGACGACCA
AATATATAATGTTATCGTTACTGCACACGCCTTCGTAATAATCTTTTTTATAGTTATGCCAATTATAATTGGAGGTTTCGGGAACTGA
CTTGTACCTTTAATAATTGGCGCCCCTGACATGGCATTCCCTCGAATAAACAACATGAGCTTCTGACTACTCCCTCCTTCCTTTCTG
CTCCTTCTCGCCTCCTCTGGGGTTGAAGCTGGAGCCGGAACAGGTTGGACCGTATATCCTCCTCTAGCAAGCAACGTCGCCCAC
TCCAGGAGCATCAGTTGACTTAACTATTTTTTCCCTCCACTTAGCGGGTGTTTCATCCATTCTTGGGGCAATTAATTTTATCACAA
CCATCTTTAATATAAAACCTGCCGCTGCTTCAATATATCAAATACCCCTGTTCGTTTGGGCCGTTCTAGTTACAGCAGTACTACTTC
TTCTTTCTCTCCCTGTCTTAGCCGCTGGCATCACTATACTATTAACG

No.2025-166-23-27

ATAGTGGGGACGGCCCTAAGCCTTTTAATTCGTGCGGAACTTAGCCAACCCGGCGCCCTCCTAGGAGACGACCAAATATATAAT
GTTATCGTTACTGCACACGCCTTCGTAATAATCTTTTTTATAGTTATGCCAATTATAATTGGAGGTTTCGGGAACTGACTTGTACCT
TTAATAATTGGCGCCCCTGACATGGCATTCCCTCGAATAAACAACATGAGCTTCTGACTACTCCCTCCTTCCTTTCTGCTCCTTCTC
GCCTCCTCTGGGGTTGAAGCTGGAGCCGGAACAGGTTGGACCGTATATCCTCCTCTAGCAAGCAACGTCGCCCACTCCAGGAG
CATCAGTTGACTTAACTATTTTTTCCCTCCACTTAGCGGGTGTTTCATCCATTCTTGGGGCAATTAATTTTATCACAACCATCTTTAA
TATAAAACCTGCCGCTGCTTCAATATATCAAATACCCCTGTTCGTTTGGGCCGTTCTAGTTACAGCAGTACTACTTCTTCTTTCTCT
CCCTGTCTTAGCCGCTGGCATCACTATACTAT

No.2025-166-23-28

TGAGCCGGAATAGTGGGGACGGCCCTAAGCCTTTTAATTCGTGCGGAACTTAGCCAACCCGGCGCCCTCCTAGGAGACGACCA
AATATATAATGTTATCGTTACTGCACACGCCTTCGTAATAATCTTTTTTATAGTTATGCCAATTATAATTGGAGGTTTCGGGAACTGA
CTTGTACCTTTAATAATTGGCGCCCCTGACATGGCATTCCCTCGAATAAACAACATGAGCTTCTGACTACTCCCTCCTTCCTTTCTG
CTCCTTCTCGCCTCCTCTGGGGTTGAAGCTGGAGCCGGAACAGGTTGGACCGTATATCCTCCTCTAGCAAGCAACGTCGCCCAC
TCCAGGAGCATCAGTTGACTTAACTATTTTTTCCCTCCACTTAGCGGGTGTTTCATCCATTCTTGGGGCAATTAATTTTATCACAA
CCATCTTTAATATAAAACCTGCCGCTGCTTCAATATATCAAATACCCCTGTTCGTTTGGGCCGTTCTAGTTACAGCAGTACTACTTC
TTCTTTCTCTCCCTGTCTTAGCCGCTGGCATCACTATACTATTAACGGATCGAA

No.2025-166-23-29

TGAGCCGGAATAGTGGGGACGGCCCTAAGCCTTTTAATTCGTGCGGAACTTAGCCAACCCGGCGCCCTCCTAGGAGACGACCA
AATATATAATGTTATCGTTACTGCACACGCCTTCGTAATAATCTTTTTTATAGTTATGCCAATTATAATTGGAGGTTTCGGGAACTGA
CTTGTACCTTTAATAATTGGCGCCCCTGACATGGCATTCCCTCGAATAAACAACATGAGCTTCTGACTACTCCCTCCTTCCTTTCTG
CTCCTTCTCGCCTCCTCTGGGGTTGAAGCTGGAGCCGGAACAGGTTGGACCGTATATCCTCCTCTAGCAAGCAACGTCGCCCAC
TCCAGGAGCATCAGTTGACTTAACTATTTTTTCCCTCCACTTAGCGGGTGTTTCATCCATTCTTGGGGCAATTAATTTTATCACAA
CCATCTTTAATATAAAACCTGCCGCTGCTTCAATATATCAAATACCCCTGTTCGTTTGGGCCGTTCTAGTTACAGCAGTACTACTTC
TTCTTTCTCTCCCTGTCTTAGCCGCTGGCATCACTATACTATTAACGGATC

No.2025-166-23-30

ATAGTGGGGACGGCCCTAAGCCTTTTAATTCGTGCGGAACTTAGCCAACCCGGCGCCCTCCTAGGAGACGACCAAATATATAAT
GTTATCGTTACTGCACACGCCTTCGTAATAATCTTTTTTATAGTTATGCCAATTATAATTGGAGGTTTCGGGAACTGACTTGTACCT
TTAATAATTGGCGCCCCTGACATGGCATTCCCTCGAATAAACAACATGAGCTTCTGACTACTCCCTCCTTCCTTTCTGCTCCTTCTC
GCCTCCTCTGGGGTTGAAGCTGGAGCCGGAACAGGTTGGACCGTATATCCTCCTCTAGCAAGCAACGTCGCCCACTCCAGGAG
CATCAGTTGACTTAACTATTTTTTCCCTCCACTTAGCGGGTGTTTCATCCATTCTTGGGGCAATTAATTTTATCACAACCATCTTTAA
TATAAAACCTGCCGCTGCTTCAATATATCAAATACCCCTGTTCGTTTGGGCCGTTCTAGTTACAGCAGTACTACTTCTTCTTTCTCT
CCCTGTCTTAGCCGCTGGCATCACTATACTATTAACG

No.2025-166-23-31

TGAGCCGGAATAGTGGGGACGGCCCTAAGCCTTTTAATTCGTGCGGAACTTAGCCAACCCGGCGCCCTCCTAGGAGACGACCA
AATATATAATGTTATCGTTACTGCACACGCCTTCGTAATAATCTTTTTTATAGTTATGCCAATTATAATTGGAGGTTTCGGGAACTGA
CTTGTACCTTTAATAATTGGCGCCCCTGACATGGCATTCCCTCGAATAAACAACATGAGCTTCTGACTACTCCCTCCTTCCTTTCTG
CTCCTTCTCGCCTCCTCTGGGGTTGAAGCTGGAGCCGGAACAGGTTGGACCGTATATCCTCCTCTAGCAAGCAACGTCGCCCAC
TCCAGGAGCATCAGTTGACTTAACTATTTTTTCCCTCCACTTAGCGGGTGTTTCATCCATTCTTGGGGCAATTAATTTTATCACAA
CCATCTTTAATATAAAACCTGCCGCTGCTTCAATATATCAAATACCCCTGTTCGTTTGGGCCGTTCTAGTTACAGCAGTACTACTTC
TTCTTTCTCTCCCTGTCTTAGCCGCTGGCATCACTATACTATTAACG

No.2025-166-23-32

TGAGCCGGAATAGTGGGGACGGCCCTAAGCCTTTTAATTCGTGCGGAACTTAGCCAACCCGGCGCCCTCCTAGGAGACGACCA
AATATATAATGTTATCGTTACTGCACACGCCTTCGTAATAATCTTTTTTATAGTTATGCCAATTATAATTGGAGGTTTCGGGAACTGA
CTTGTACCTTTAATAATTGGCGCCCCTGACATGGCATTCCCTCGAATAAACAACATGAGCTTCTGACTACTCCCTCCTTCCTTTCTG
CTCCTTCTCGCCTCCTCTGGGGTTGAAGCTGGAGCCGGAACAGGTTGGACCGTATATCCTCCTCTAGCAAGCAACGTCGCCCAC
TCCAGGAGCATCAGTTGACTTAACTATTTTTTCCCTCCACTTAGCGGGTGTTTCATCCATTCTTGGGGCAATTAATTTTATCACAA
CCATCTTTAATATAAAACCTGCCGCTGCTTCAATATATCAAATACCCCTGTTCGTTTGGGCCGTTCTAGTTACAGCAGTACTACTTC
TTCTTTCTCTCCCTGTCTTAGCCGCTGGCATCACTATACTATTAACGGATCGA

No.2025-166-23-33

TGAGCCGGAATAGTGGGGACGGCCCTAAGCCTTTTAATTCGTGCGGAACTTAGCCAACCCGGCGCCCTCCTAGGAGACGACCA
AATATATAATGTTATCGTTACTGCACACGCCTTCGTAATAATCTTTTTTATAGTTATGCCAATTATAATTGGAGGTTTCGGGAACTGA
CTTGTACCTTTAATAATTGGCGCCCCTGACATGGCATTCCCTCGAATAAACAACATGAGCTTCTGACTACTCCCTCCTTCCTTTCTG
CTCCTTCTCGCCTCCTCTGGGGTTGAAGCTGGAGCCGGAACAGGTTGGACCGTATATCCTCCTTTAGCAAGCAACGTCGCCCAC
TCCAGGAGCATCAGTTGACTTAACTATTTTTTCCCTCCACTTAGCGGGTGTTTCATCCATTCTTGGGGCAATTAATTTTATCACAA
CCATCTTTAATATAAAACCTGCCGCTGCTTCAATATATCAAATACCCCTGTTCGTTTGGGCCGTTCTAGTTACAGCAGTACTACTTC
TTCTTTCTCTCCCTGTCTTAGCCGCTGGCATCACTATACTATTAACG

No.2025-166-23-34

TGAGCCGGAATAGTGGGGACGGCCCTAAGCCTTTTAATTCGTGCGGAACTTAGCCAACCCGGCGCCCTCCTAGGAGACGACCA
AATATATAATGTTATCGTTACTGCACACGCCTTCGTAATAATCTTTTTTATAGTTATGCCAATTATAATTGGAGGTTTCGGGAACTGA
CTTGTACCTTTAATAATTGGCGCCCCTGACATGGCATTCCCTCGAATAAACAACATGAGCTTCTGACTACTCCCTCCTTCCTTTCTG
CTCCTTCTCGCCTCCTCTGGGGTTGAAGCTGGAGCCGGAACAGGTTGGACCGTATATCCTCCTCTAGCAAGCAACGTCGCCCAC
TCCAGGAGCATCAGTTGACTTAACTATTTTTTCCCTCCACTTAGCGGGTGTTTCATCCATTCTTGGGGCAATTAATTTTATCACAA
CCATCTTTAATATAAAACCTGCCGCTGCTTCAATATATCAAATACCCCTGTTCGTTTGGGCCGTTCTAGTTACAGCAGTACTACTTC
TTCTTTCTCTCCCTGTCTTAGCCGCTGGCATCACTATACTAT

No.2025-166-23-35

TGAGCCGGAATAGTGGGGACGGCCCTAAGCCTTTTAATTCGTGCGGAACTTAGCCAACCCGGCGCCCTCCTAGGAGACGACCA
AATATATAATGTTATCGTTACTGCACACGCCTTCGTAATAATCTTTTTTATAGTTATGCCAATTATAATTGGAGGTTTCGGGAACTGA
CTTGTACCTTTAATAATTGGCGCCCCTGACATGGCATTCCCTCGAATAAACAACATGAGCTTCTGACTACTCCCTCCTTCCTTTCTG
CTCCTTCTCGCCTCCTCTGGGGTTGAAGCTGGAGCCGGAACAGGTTGGACCGTATATCCTCCTCTAGCAAGCAACGTCGCCCAC
TCCAGGAGCATCAGTTGACTTAACTATTTTTTCCCTCCACTTAGCGGGTGTTTCATCCATTCTTGGGGCAATTAATTTTATCACAA
CCATCTTTAATATAAAACCTGCCGCTGCTTCAATATATCAAATACCCCTGTTCGTTTGGGCCGTTCTAGTTACAGCAGTACTACTTC
TTCTTTCTCTCCCTGTCTTAGCCGCTGGCATCACTATACTAT

No.2025-166-23-36

TGAGCCGGAATAGTGGGGACGGCCCTAAGCCTTTTAATTCGTGCGGAACTTAGCCAACCCGGCGCCCTCCTAGGAGACGACCA
AATATATAATGTTATCGTTACTGCACACGCCTTCGTAATAATCTTTTTTATAGTTATGCCAATTATAATTGGAGGTTTCGGGAACTGA
CTTGTACCTTTAATAATTGGCGCCCCTGACATGGCATTCCCTCGAATAAACAACATGAGCTTCTGACTACTCCCTCCTTCCTTTCTG
CTCCTTCTCGCCTCCTCTGGGGTTGAAGCTGGAGCCGGAACAGGTTGGACCGTATATCCTCCTCTAGCAAGCAACGTCGCCCAC
TCCAGGAGCATCAGTTGACTTAACTATTTTTTCCCTCCACTTAGCGGGTGTTTCATCCATTCTTGGGGCAATTAATTTTATCACAA
CCATCTTTAATATAAAACCTGCCGCTGCTTCAATATATCAAATACCCCTGTTCGTTTGGGCCGTTCTAGTTACAGCAGTACTACTTC
TTCTTTCTCTCCCTGTCTTAGCCGCTGGCATCACTATACTA-TAACGGATC

No.2025-166-23-37

TGAGCCGGAATAGTGGGGACGGCCCTAAGCCTTTTAATTCGTGCGGAACTTAGCCAACCCGGCGCCCTCCTAGGAGACGACCA
AATATATAATGTTATCGTTACTGCACACGCCTTCGTAATAATCTTTTTTATAGTTATGCCAATTATAATTGGAGGTTTCGGGAACTGA
CTTGTACCTTTAATAATTGGCGCCCCTGACATGGCATTCCCTCGAATAAACAACATGAGCTTCTGACTACTCCCTCCTTCCTTTCTG
CTCCTTCTCGCCTCCTCTGGGGTTGAAGCTGGAGCCGGAACAGGTTGGACCGTATATCCTCCTCTAGCAAGCAACGTCGCCCAC
TCCAGGAGCATCAGTTGACTTAACTATTTTTTCCCTCCACTTAGCGGGTGTTTCATCCATTCTTGGGGCAATTAATTTTATCACAA
CCATCTTTAATATAAAACCTGCCGCTGCTTCAATATATCAAATACCCCTGTTCGTTTGGGCCGTTCTAGTTACAGCAGTACTACTTC
TTCTTTCTCTCCCTGTCTTAGCCGCTGGCATCACTATACTAT

No.2025-166-23-38

ATAGTGGGGACGGCCCTAAGCCTTTTAATTCGTGCGGAACTTAGCCAACCCGGCGCCCTCCTAGGAGACGACCAAATATATAAT
GTTATCGTTACTGCACACGCCTTCGTAATAATCTTTTTTATAGTTATGCCAATTATAATTGGAGGTTTCGGGAACTGACTTGTACCT
TTAATAATTGGCGCCCCTGACATGGCATTCCCTCGAATAAACAACATGAGCTTCTGACTACTCCCTCCTTCCTTTCTGCTCCTTCTC
GCCTCCTCTGGGGTTGAAGCTGGAGCCGGAACAGGTTGGACCGTATATCCTCCTCTAGCAAGCAACGTCGCCCACTCCAGGAG
CATCAGTTGACTTAACTATTTTTTCCCTCCACTTAGCGGGTGTTTCATCCATTCTTGGGGCAATTAATTTTATCACAACCATCTTTAA
TATAAAACCTGCCGCTGCTTCAATATATCAAATACCCCTGTTCGTTTGGGCCGTTCTAGTTACAGCAGTACTACTTCTTCTTTCTCT
CCCTGTCTTAGCCGCTGGCATCACTATACTAT

No.2025-166-23-39

TGAGCCGGAATAGTGGGGACGGCCCTAAGCCTTTTAATTCGTGCGGAACTTAGCCAACCCGGCGCCCTCCTAGGAGACGACCA
AATATATAATGTTATCGTTACTGCACACGCCTTCGTAATAATCTTTTTTATAGTTATGCCAATTATAATTGGAGGTTTCGGGAACTGA
CTTGTACCTTTAATAATTGGCGCCCCTGACATGGCATTCCCTCGAATAAACAACATGAGCTTCTGACTACTCCCTCCTTCCTTTCTG
CTCCTTCTCGCCTCCTCTGGGGTTGAAGCTGGAGCCGGAACAGGTTGGACCGTATATCCTCCTCTAGCAAGCAACGTCGCCCAC
TCCAGGAGCATCAGTTGACTTAACTATTTTTTCCCTCCACTTAGCGGGTGTTTCATCCATTCTTGGGGCAATTAATTTTATCACAA
CCATCTTTAATATAAAACCTGCCGCTGCTTCAATATATCAAATACCCCTGTTCGTTTGGGCCGTTCTAGTTACAGCAGTACTACTTC
TTCTTTCTCTCCCTGTCTTAGCCGCTGGCATCACTATACTAT

No.2025-166-23-40

TGAGCCGGAATAGTGGGGACGGCCCTAAGCCTTTTAATTCGTGCGGAACTTAGCCAACCCGGCGCCCTCCTAGGAGACGACCA
AATATATAATGTTATCGTTACTGCACACGCCTTCGTAATAATCTTTTTTATAGTTATGCCAATTATAATTGGAGGTTTCGGGAACTGA
CTTGTACCTTTAATAATTGGCGCCCCTGACATGGCATTCCCTCGAATAAACAACATGAGCTTCTGACTACTCCCTCCTTCCTTTCTG
CTCCTTCTCGCCTCCTCTGGGGTTGAAGCTGGAGCCGGAACAGGTTGGACCGTATATCCTCCTCTAGCAAGCAACGTCGCCCAC
TCCAGGAGCATCAGTTGACTTAACTATTTTTTCCCTCCACTTAGCGGGTGTTTCATCCATTCTTGGGGCAATTAATTTTATCACAA
CCATCTTTAATATAAAACCTGCCGCTGCTTCAATATATCAAATACCCCTGTTCGTTTGGGCCGTTCTAGTTACAGCAGTACTACTTC
TTCTTTCTCTCCCTGTCTTAGCCGCTGGCATCACTATACTATTAACG

No.2025-166-23-41

TGAGCCGGAATAGTGGGGACGGCCCTAAGCCTTTTAATTCGTGCGGAACTTAGCCAACCCGGCGCCCTCCTAGGAGACGACCA
AATATATAATGTTATCGTTACTGCACACGCCTTCGTAATAATCTTTTTTATAGTTATGCCAATTATAATTGGAGGTTTCGGGAACTGA
CTTGTACCTTTAATAATTGGCGCCCCTGACATGGCATTCCCTCGAATAAACAACATGAGCTTCTGACTACTCCCTCCTTCCTTTCTG
CTCCTTCTCGCCTCCTCTGGGGTTGAAGCTGGAGCCGGAACAGGTTGGACCGTATATCCTCCTTTAGCAAGCAACGTCGCCCAC
TCCAGGAGCATCAGTTGACTTAACTATTTTTTCCCTCCACTTAGCGGGTGTTTCATCCATTCTTGGGGCAATTAATTTTATCACAA
CCATCTTTAATATAAAACCTGCCGCTGCTTCAATATATCAAATACCCCTGTTCGTTTGGGCCGTTCTAGTTACAGCAGTACTACTTC
TTCTTTCTCTCCCTGTCTTAGCCGCTGGCATCACTATACTATTAACGGATC

No.2025-166-23-42

TGAGCCGGAATAGTGGGGACGGCCCTAAGCCTTTTAATTCGTGCGGAACTTAGCCAACCCGGCGCCCTCCTAGGAGACGACCA
AATATATAATGTTATCGTTACTGCACACGCCTTCGTAATAATCTTTTTTATAGTTATGCCAATTATAATTGGAGGTTTCGGGAACTGA
CTTGTACCTTTAATAATTGGCGCCCCTGACATGGCATTCCCTCGAATAAACAACATGAGCTTCTGACTACTCCCTCCTTCCTTTCTG
CTCCTTCTCGCCTCCTCTGGGGTTGAAGCTGGAGCCGGAACAGGTTGGACCGTATATCCTCCTCTAGCAAGCAACGTCGCCCAC
TCCAGGAGCATCAGTTGACTTAACTATTTTTTCCCTCCACTTAGCGGGTGTTTCATCCATTCTTGGGGCAATTAATTTTATCACAA
CCATCTTTAATATAAAACCTGCCGCTGCTTCAATATATCAAATACCCCTGTTCGTTTGGGCCGTTCTAGTTACAGCAGTACTACTTC
TTCTTTCTCTCCCTGTCTTAGCCGCTGGCATCACTATACTATTAACGGATCGAA

No.2025-166-23-43

TGAGCCGGAATAGTGGGGACGGCCCTAAGCCTTTTAATTCGTGCGGAACTTAGCCAACCCGGCGCCCTCCTAGGAGACGACCA
AATATATAATGTTATCGTTACTGCACACGCCTTCGTAATAATCTTTTTTATAGTTATGCCAATTATAATTGGAGGTTTCGGGAACTGA
CTTGTACCTTTAATAATTGGCGCCCCTGACATGGCATTCCCTCGAATAAACAACATGAGCTTCTGACTACTCCCTCCTTCCTTTCTG
CTCCTTCTCGCCTCCTCTGGGGTTGAAGCTGGAGCCGGAACAGGTTGGACCGTATATCCTCCTCTAGCAAGCAACGTCGCCCAC
TCCAGGAGCATCAGTTGACTTAACTATTTTTTCCCTCCACTTAGCGGGTGTTTCATCCATTCTTGGGGCAATTAATTTTATCACAA
CCATCTTTAATATAAAACCTGCCGCTGCTTCAATATATCAAATACCCCTGTTCGTTTGGGCCGTTCTAGTTACAGCAGTACTACTTC
TTCTTTCTCTCCCTGTCTTAGCCGCTGGCATCACTATACTATTAACGGATCGAA

No.2025-166-23-44

TGAGCCGGAATAGTGGGGACGGCCCTAAGCCTTTTAATTCGTGCGGAACTTAGCCAACCCGGCGCCCTCCTAGGAGACGACCA
AATATATAATGTTATCGTTACTGCACACGCCTTCGTAATAATCTTTTTTATAGTTATGCCAATTATAATTGGAGGTTTCGGGAACTGA
CTTGTACCTTTAATAATTGGCGCCCCTGACATGGCATTCCCTCGAATAAACAACATGAGCTTCTGACTACTCCCTCCTTCCTTTCTG
CTCCTTCTCGCCTCCTCTGGGGTTGAAGCTGGAGCCGGAACAGGTTGGACCGTATATCCTCCTCTAGCAAGCAACGTCGCCCAC
TCCAGGAGCATCAGTTGACTTAACTATTTTTTCCCTCCACTTAGCGGGTGTTTCATCCATTCTTGGGGCAATTAATTTTATCACAA
CCATCTTTAATATAAAACCTGCCGCTGCTTCAATATATCAAATACCCCTGTTCGTTTGGGCCGTTCTAGTTACAGCAGTACTACTTC
TTCTTTCTCTCCCTGTCTTAGCCGCTGGCATCACTATACTAT

No.2025-166-23-45

TGAGCCGGAATAGTGGGGACGGCCCTAAGCCTTTTAATTCGTGCGGAACTTAGCCAACCCGGCGCCCTCCTAGGAGACGACCA
AATATATAATGTTATCGTTACTGCACACGCCTTCGTAATAATCTTTTTTATAGTTATGCCAATTATAATTGGAGGTTTCGGGAACTGA
CTTGTACCTTTAATAATTGGCGCCCCTGACATGGCATTCCCTCGAATAAACAACATGAGCTTCTGACTACTCCCTCCTTCCTTTCTG
CTCCTTCTCGCCTCCTCTGGGGTTGAAGCTGGAGCCGGAACAGGTTGGACCGTATATCCTCCTCTAGCAAGCAACGTCGCCCAC
TCCAGGAGCATCAGTTGACTTAACTATTTTTTCCCTCCACTTAGCGGGTGTTTCATCCATTCTTGGGGCAATTAATTTTATCACAA
CCATCTTTAATATAAAACCTGCCGCTGCTTCAATATATCAAATACCCCTGTTCGTTTGGGCCGTTCTAGTTACAGCAGTACTACTTC
TTCTTTCTCTCCCTGTCTTAGCCGCTGGCATCACTATACTATTAACGGATC

No.2025-166-23-46

TGAGCCGGAATAGTGGGGACGGCCCTAAGCCTTTTAATTCGTGCGGAACTTAGCCAACCCGGCGCCCTCCTAGGAGACGACCA
AATATATAATGTTATCGTTACTGCACACGCCTTCGTAATAATCTTTTTTATAGTTATGCCAATTATAATTGGAGGTTTCGGGAACTGA
CTTGTACCTTTAATAATTGGCGCCCCTGACATGGCATTCCCTCGAATAAACAACATGAGCTTCTGACTACTCCCTCCTTCCTTTCTG
CTCCTTCTCGCCTCCTCTGGGGTTGAAGCTGGAGCCGGAACAGGTTGGACCGTATATCCTCCTCTAGCAAGCAACGTCGCCCAC
TCCAGGAGCATCAGTTGACTTAACTATTTTTTCCCTCCACTTAGCGGGTGTTTCATCCATTCTTGGGGCAATTAATTTTATCACAA
CCATCTTTAATATAAAACCTGCCGCTGCTTCAATATATCAAATACCCCTGTTCGTTTGGGCCGTTCTAGTTACAGCAGTACTACTTC
TTCTTTCTCTCCCTGTCT-TAGCCGCTGGCATCACTATACTATTAACGGATC

No.2025-166-23-47

TGAGCCGGAATAGTGGGGACGGCCCTAAGCCTTTTAATTCGTGCGGAACTTAGCCAACCCGGCGCCCTCCTAGGAGACGACCA
AATATATAATGTTATCGTTACTGCACACGCCTTCGTAATAATCTTTTTTATAGTTATGCCAATTATAATTGGAGGTTTCGGGAACTGA
CTTGTACCTTTAATAATTGGCGCCCCTGACATGGCATTCCCTCGAATAAACAACATGAGCTTCTGACTACTCCCTCCTTCCTTTCTG
CTCCTTCTCGCCTCCTCTGGGGTTGAAGCTGGAGCCGGAACAGGTTGGACCGTATATCCTCCTCTAGCAAGCAACGTCGCCCAC
TCCAGGAGCATCAGTTGACTTAACTATTTTTTCCCTCCACTTAGCGGGTGTTTCATCCATTCTTGGGGCAATTAATTTTATCACAA
CCATCTTTAATATAAAACCTGCCGCTGCTTCAATATATCAAATACCCCTGTTCGTTTGGGCCGTTCTAGTTACAGCAGTACTACTTC
TTCTTTCTCTCCCTGTCTTAGCCGCTGGCATCACTATACTATTAACG

No.2025-166-23-48

TGAGCCGGAATAGTGGGGACGGCCCTAAGCCTTTTAATTCGTGCGGAACTTAGCCAACCCGGCGCCCTCCTAGGAGACGACCA
AATATATAATGTTATCGTTACTGCACACGCCTTCGTAATAATCTTTTTTATAGTTATGCCAATTATAATTGGAGGTTTCGGGAACTGA
CTTGTACCTTTAATAATTGGCGCCCCTGACATGGCATTCCCTCGAATAAACAACATGAGCTTCTGACTACTCCCTCCTTCCTTTCTG
CTCCTTCTCGCCTCCTCTGGGGTTGAAGCTGGAGCCGGAACAGGTTGGACCGTATATCCTCCTCTAGCAAGCAACGTCGCCCAC
TCCAGGAGCATCAGTTGACTTAACTATTTTTTCCCTCCACTTAGCGGGTGTTTCATCCATTCTTGGGGCAATTAATTTTATCACAA
CCATCTTTAATATAAAACCTGCCGCTGCTTCAATATATCAAATACCCCTGTTCGTTTGGGCCGTTCTAGTTACAGCAGTACTACTTC
TTCTTTCTCTCCCTGTCTTAGCCGCTGGCATCACTATACTATTAACGGATCGAAACCTAAATACTGCCTTCTTTGACCCTGCAGGCG
GCGGAGACCCGATCCTCTACC

No.2025-166-23-49

TGAGCCGGAATAGTGGGGACGGCCCTAAGCCTTTTAATTCGTGCGGAACTTAGCCAACCCGGCGCCCTCCTAGGAGACGACCA
AATATATAATGTTATCGTTACTGCACACGCCTTCGTAATAATCTTTTTTATAGTTATGCCAATTATAATTGGAGGTTTCGGGAACTGA
CTTGTACCTTTAATAATTGGCGCCCCTGACATGGCATTCCCTCGAATAAACAACATGAGCTTCTGACTACTCCCTCCTTCCTTTCTG
CTCCTTCTCGCCTCCTCTGGGGTTGAAGCTGGAGCCGGAACAGGTTGGACCGTATATCCTCCTCTAGCAAGCAACGTCGCCCAC
TCCAGGAGCATCAGTTGACTTAACTATTTTTTCCCTCCACTTAGCGGGTGTTTCATCCATTCTTGGGGCAATTAATTTTATCACAA
CCATCTTTAATATAAAACCTGCCGCTGCTTCAATATATCAAATACCCCTGTTCGTTTGGGCCGTTCTAGTTACAGCAGTACTACTTC
TTCTTTCTCTCCCTGTCTTAGCCGCTGGCATCACTATACTATTAACGGATCGAAACCTAAATACTGCCTTCTTTGACCCTGCAGGCG
GCGGAGACCCGA

No.2025-166-23-50

TGAGCCGGAATAGTGGGGACGGCCCTAAGCCTTTTAATTCGTGCGGAACTTAGCCAACCCGGCGCCCTCCTAGGAGACGACCA
AATATATAATGTTATCGTTACTGCACACGCCTTCGTAATAATCTTTTTTATAGTTATGCCAATTATAATTGGAGGTTTCGGGAACTGA
CTTGTACCTTTAATAATTGGCGCCCCTGACATGGCATTCCCTCGAATAAACAACATGAGCTTCTGACTACTCCCTCCTTCCTTTCTG
CTCCTTCTCGCCTCCTCTGGGGTTGAAGCTGGAGCCGGAACAGGTTGGACCGTATATCCTCCTCTAGCAAGCAACGTCGCCCAC
TCCAGGAGCATCAGTTGACTTAACTATTTTTTCCCTCCACTTAGCGGGTGTTTCATCCATTCTTGGGGCAATTAATTTTATCACAA
CCATCTTTAATATAAAACCTGCCGCTGCTTCAATATATCAAATACCCCTGTTCGTTTGGGCCGTTCTAGTTACAGCAGTACTACTTC
TTCTTTCTCTCCCTGTCTTAGCCGCTGGCATCACTATACTATTAACG

No.2025-166-23-51

TGAGCCGGAATAGTGGGGACGGCCCTAAGCCTTTTAATTCGTGCGGAACTTAGCCAACCCGGCGCCCTCCTAGGAGACGACCA
AATATATAATGTTATCGTTACTGCACACGCCTTCGTAATAATCTTTTTTATAGTTATGCCAATTATAATTGGAGGTTTCGGGAACTGA
CTTGTACCTTTAATAATTGGCGCCCCTGACATGGCATTCCCTCGAATAAACAACATGAGCTTCTGACTACTCCCTCCTTCCTTTCTG
CTCCTTCTCGCCTCCTCTGGGGTTGAAGCTGGAGCCGGAACAGGTTGGACCGTATATCCTCCTCTAGCAAGCAACGTCGCCCAC
TCCAGGAGCATCAGTTGACTTAACTATTTTTTCCCTCCACTTAGCGGGTGTTTCATCCATTCTTGGGGCAATTAATTTTATCACAA
CCATCTTTAATATAAAACCTGCCGCTGCTTCAATATATCAAATACCCCTGTTCGTTTGGGCCGTTCTAGTTACAGCAGTACTACTTC
TTCTTTCTCTCCCTGTCTTAGCCGCTGGCATCACTATACTATTAACGGATC

No.2025-166-23-52

TGAGCCGGAATAGTGGGGACGGCCCTAAGCCTTTTAATTCGTGCGGAACTTAGCCAACCCGGCGCCCTCCTAGGAGACGACCA
AATATATAATGTTATCGTTACTGCACACGCCTTCGTAATAATCTTTTTTATAGTTATGCCAATTATAATTGGAGGTTTCGGGAACTGA
CTTGTACCTTTAATAATTGGCGCCCCTGACATGGCATTCCCTCGAATAAACAACATGAGCTTCTGACTACTCCCTCCTTCCTTTCTG
CTCCTTCTCGCCTCCTCTGGGGTTGAAGCTGGAGCCGGAACAGGTTGGACCGTATATCCTCCTCTAGCAAGCAACGTCGCCCAC
TCCAGGAGCATCAGTTGACTTAACTATTTTTTCCCTCCACTTAGCGGGTGTTTCATCCATTCTTGGGGCAATTAATTTTATCACAA
CCATCTTTAATATAAAACCTGCCGCTGCTTCAATATATCAAATACCCCTGTTCGTTTGGGCCGTTCTAGTTACAGCAGTACTACTTC
TTCTTTCTCTCCCTGTCTTAGCCGCTGGCATCACTATACTATTAACGGATC

No.2025-166-23-53

TGAGCCGGAATAGTGGGGACGGCCCTAAGCCTTTTAATTCGTGCGGAACTTAGCCAACCCGGCGCCCTCCTAGGAGACGACCA
AATATATAATGTTATCGTTACTGCACACGCCTTCGTAATAATCTTTTTTATAGTTATGCCAATTATAATTGGAGGTTTCGGGAACTGA
CTTGTACCTTTAATAATTGGCGCCCCTGACATGGCATTCCCTCGAATAAACAACATGAGCTTCTGACTACTCCCTCCTTCCTTTCTG
CTCCTTCTCGCCTCCTCTGGGGTTGAAGCTGGAGCCGGAACAGGTTGGACCGTATATCCTCCTCTAGCAAGCAACGTCGCCCAC
TCCAGGAGCATCAGTTGACTTAACTATTTTTTCCCTCCACTTAGCGGGTGTTTCATCCATTCTTGGGGCAATTAATTTTATCACAA
CCATCTTTAATATAAAACCTGCCGCTGCTTCAATATATCAAATACCCCTGTTCGTTTGGGCCGTTCTAGTTACAGCAGTACTACTTC
TTCTTTCTCTCCCTGTCTTAGCCGCTGGCATCACTATACTAT

No.2025-166-23-54

TGAGCCGGAATAGTGGGGACGGCCCTAAGCCTTTTAATTCGTGCGGAACTTAGCCAACCCGGCGCCCTCCTAGGAGACGACCA
AATATATAATGTTATCGTTACTGCACACGCCTTCGTAATAATCTTTTTTATAGTTATGCCAATTATAATTGGAGGTTTCGGGAACTGA
CTTGTACCTTTAATAATTGGCGCCCCTGACATGGCATTCCCTCGAATAAACAACATGAGCTTCTGACTACTCCCTCCTTCCTTTCTG
CTCCTTCTCGCCTCCTCTGGGGTTGAAGCTGGAGCCGGAACAGGTTGGACCGTATATCCTCCTCTAGCAAGCAACGTCGCCCAC
TCCAGGAGCATCAGTTGACTTAACTATTTTTTCCCTCCACTTAGCGGGTGTTTCATCCATTCTTGGGGCAATTAATTTTATCACAA
CCATCTTTAATATAAAACCTGCCGCTGCTTCAATATATCAAATACCCCTGTTCGTTTGGGCCGTTCTAGTTACAGCAGTACTACTTC
TTCTTTCTCTCCCTGTCTTAGCCGCTGGCATCACTATACTATTAACGGATC

Fish maw: COI gene - Large-sized cylindrical samples (n=24)

No.2025-166-24-1

ATAGTAGGCACGTCTCTAAGCCTCCTAATCCGAGCAGAACTGAGTCAGCCCGGCTCACTCCTCGGAGATGACCAGATTTTTAAC
GTAATTGTTACGGCACATGCCTTCGTTATAATTTTCTTTATAGTAATACCCGTTATGATCGGAGGGTTCGGAAACTGACTCGTTCCC
CTAATGATCGGAGCCCCCGACATGGCCTTCCCCCGAATGAACAATATGAGTTTCTGACTCCTTCCCCCTTCTTTCCTACTACTTCTT
GTCTCTTCGGCGGTAGAGGCAGGGGCCGGAACAGGGTGAACAGTCTACCCGCCACTCGCCGGAAATCTCGCACACGCAGGTG
CTTCTGTCGACTTAGCTATTTTCTCCCTTCATCTCGCAGGTGTATCCTCAATTCTGGGGGCCATTAATTTTATTACCACGATTATTAA
CATGAAACCCCCTGCGATTTCTCAGTACCAGACACCTTTGTTCGTATGGGCCGTCTTAATTACAGCGGTTCTCCTACTACTCTCAC
TTCCTGTCCTGGCTGCTGGCATTACAATGCTTCTAACAGATCGCAACCTTAACACAACCTTCTTCGACCCAGGGGGGCGGAGGA
GATCCGATTCTTTATCAGCACCTATTCTGATTCT

No.2025-166-24-2

TAGTAGGCACGTCTCTAAGCCTCCTAATCCGAGCAGAACTGAGTCAGCCCGGCTCACTCCTCGGAGATGACCAGATTTTTAACG
TAATTGTTACGGCACATGCCTTCGTTATAATTTTCTTTATAGTAATACCCGTTATGATCGGAGGGTTCGGAAACTGACTCGTTCCCC
TAATGATCGGAGCCCCCGACATGGCCTTCCCCCGAATGAACAATATGAGTTTCTGACTCCTTCCCCCTTCTTTCCTACTACTTCTT
GTCTCTTCGGCGGTAGAGGCAGGGGCCGGAACAGGGTGAACAGTCTACCCGCCACTCGCCGGAAATCTCGCACACGCAGGTG
CTTCTGTCGACTTAGCTATTTTCTCCCTTCATCTCGCAGGTGTATCCTCAATTCTGGGGGCCATTAATTTTATTACCACGATTATTAA
CATGAAACCCCCTGCGATTTCTCAGTACCAGACACCTTTGTTCGTATGGGCCGTCTTAATTACAGCGGTTCTCCTACTACTCTCAC
TTCCTGTCCTGGCTGCTGGCATTACAATGCTTCTAACAGA

No.2025-166-24-3

CCTCCTAATCCGAGCAGAACTAAGTCAGCCAGGCTCACTCCTCGGAGATGACCAGATTTTTAACGTAATTGTTACGGCACATGCC
TTTGTCATGATTTTCTTTATAGTAATACCCGTCATGATTGGAGGGTTCGGAAACTGACTTGTACCCCTAATGATCGGAGCCCCCGA
CATGGCCTTTCCCCGGATGAACAACATAAGCTTCTGGCTTCTCCCCCCTTCTTTCCTGCTACTCCTCACTTCTTCCGGAGTAGAGG
CAGGTGCCGGGACAGGGTGAACAGTCTACCCCCCGCTCGCTGGTAACCTCGCACACGCAGGTGCTTCTGTCGACCTGGCTATC
TTTTCCCTCCATCTCGCAGGTGTATCCTCGATTCTAGGGGCTATTAACTTTATTACAACGATTATTAATATGAAGCCCCCAGCAATT
TCTCAGTACCAGACACCTTTATTCGTATGGGCCGTCTTAATTACTGCTGTCCTCCTACTACTCTCACTCCCTGTCCTAGCTGCCGGC
ATTACAATG

No.2025-166-24-4

TAGTAGGCACGTCTCTAAGCCTCCTAATCCGAGCAGAACTGAGTCAGCCCGGCTCACTCCTCGGAGATGACCAGATTTTTAACG
TAATTGTTACGGCACATGCCTTCGTTATAATTTTCTTTATAGTAATACCCGTTATGATCGGAGGGTTCGGAAACTGACTCGTTCCCC
TAATGATCGGAGCCCCCGACATGGCCTTCCCCCGAATGAACAATATGAGTTTCTGACTCCTTCCCCCTTCTTTCCTACTACTTCTT
GTCTCTTCGGCGGTAGAGGCAGGGGCCGGAACAGGGTGAACAGTCTACCCGCCACTCGCCGGAAATCTCGCACACGCAGGTG
CTTCTGTCGACTTAGCTATTTTCTCCCTTCATCTCGCAGGTGTATCCTCAATTCTGGGGGCCATTAATTTTATTACCACGATTATTAA
CATGAAACCCCCTGCGATTTCTCAGTACCAGACACCTTTGTTCGTATGGGCCGTCTTAATTACAGCGGTTCTCCTACTACTCTCAC
TTCCTGTCCTGGCTGCTGGCATTACAATGCTTCTAACAGA

No.2025-166-24-5

ATAGTAGGCACAGCCTTAAGCCTCCTAATCCGAGCAGAACTAAGTCAGCCAGGCTCACTCCTCGGAGATGACCAGATTTTTAAC
GTAATTGTTACGGCACATGCCTTTGTCATGATTTTCTTTATAGTAATACCCGTCATGATTGGAGGGTTCGGAAACTGACTTGTACC
CCTAATGATCGGAGCCCCCGACATGGCCTTTCCCCGGATGAACAACATAAGCTTCTGGCTTCTCCCCCCTTCTTTCCTGCTACTCC
TCACTTCTTCCGGAGTAGAGGCAGGTGCCGGGACAGGGTGAACAGTCTACCCCCCGCTCGCTGGTAACCTCGCACACGCAGGT
GCTTCTGTCGACCTGGCTATCTTTTCCCTCCATCTCGCAGGTGTATCCTCGATTCTAGGGGCTATTAACTTTATTACAACGATTATTA
ATATGAAGCCCCCAGCAATTTCTCAGTACCAGACACCTTTATTCGTATGGGCCGTCTTAATTACTGCTGTCCTCCTACTACTCTCAC
TCCCTGTCCTAGCTGCCGGCATTACAATGCTTCTAACAGACC

No.2025-166-24-6

GCCTCCTAATCCGAGCAGAACTAAGTCAGCCAGGCTCACTCCTCGGAGATGACCAGATTTTTAACGTAATTGTTACGGCACATG
CCTTTGTCATGATTTTCTTTATAGTAATACCCGTCATGATTGGAGGGTTCGGAAACTGACTTGTACCCCTAATGATCGGAGCCCCC
GACATGGCCTTTCCCCGGATGAACAACATAAGCTTCTGGCTTCTCCCCCCTTCTTTCCTGCTACTCCTCACTTCTTCCGGAGTAGA
GGCAGGTGCCGGGACAGGGTGAACAGTCTACCCCCCGCTCGCTGGTAATCTCGCACACGCAGGTGCTTCTGTCGACCTGGCTA
TCTTTTCCCTCCATCTCGCAGGTGTATCCTCGATTCTAGGGGCTATTAACTTTATTACAACGATTATTAATATGAAGCCCCCAGCAA
TTTCTCAGTACCAGACGCCTTTATTCGTATGGGCCGTCTTAATTACTGCTGTCCTCCTACTACTCTCACTCCCTGTCCTAGCTGCCG
GCA

No.2025-166-24-7

ATAGTAGGCACAGCCTTAAGCCTCCTAATCCGAGCAGAACTAAGTCAGCCAGGCTCACTCCTCGGAGATGACCAGATTTTTAAC
GTAATTGTTACGGCACATGCCTTTGTCATGATTTTCTTTATAGTAATACCCGTCATGATTGGAGGGTTCGGAAACTGACTTGTACC
CCTAATGATCGGAGCCCCCGACATGGCCTTTCCCCGGATGAACAACATAAGCTTCTGGCTTCTCCCCCCTTCTTTCCTGCTACTCC
TCACTTCTTCCGGAGTAGAGGCAGGTGCCGGGACAGGGTGAACAGTCTACCCCCCGCTCGCTGGTAACCTCGCACACGCAGGT
GCTTCTGTCGACCTGGCTATCTTTTCCCTCCATCTCGCAGGTGTATCCTCGATTCTAGGGGCTATTAACTTTATTACAACGATTATTA
ATATGAAGCCCCCAGCAATTTCTCAGTACCAGACACCTTTATTCGTATGGGCCGTCTTAATTACTGCTGTCCTCCTACTACTCTCAC
TCCCTGTCCTAGCTGCCGGCATTACAATGCTTCTAACAGACCGCAACCTCAACACAACCTTC

No.2025-166-24-8

TAGTAGGCACGTCTCTAAGCCTCCTAATCCGAGCAGAACTGAGTCAGCCCGGCTCACTCCTCGGAGATGACCAGATTTTTAACG
TAATTGTTACGGCACATGCCTTCGTTATAATTTTCTTTATAGTAATACCCGTTATGATCGGAGGGTTCGGAAACTGACTCGTTCCCC

TAATGATCGGAGCCCCCGACATGGCCTTCCCCCGAATGAACAATATGAGTTTCTGACTCCTTCCCCCTTCTTTCCTACTACTTCTT
GTCTCTTCGGCGGTAGAGGCAGGGGCCGGAACAGGGTGAACAGTCTACCCGCCACTCGCCGGAAATCTCGCACACGCAGGTG
CTTCTGTCGACTTAGCTATTTTCTCCCTTCATCTCGCAGGTGTATCCTCAATTCTGGGGGCCATTAATTTTATTACCACGATTATTAA
CATGAAACCCCCTGCGATTTCTCAGTACCAGACACCTTTGTTCGTATGGGCCGTCTTAATTACAGCGGTTCTCCTACTACTCTCAC
TTCCTGTCCTGGCTGCTGGCATTACAATGCTTCT

No.2025-166-24-9

ATAGTAGGCACAGCCTTAAGCCTCCTAATCCGAGCAGAACTAAGTCAGCCAGGCTCACTCCTCGGAGATGACCAGATTTTTAAC
GTAATTGTTACGGCACATGCCTTTGTCATGATTTTCTTTATAGTAATACCCGTCATGATTGGAGGGTTCGGAAACTGACTTGTACC
CCTAATGATCGGAGCCCCCGACATGGCCTTTCCCCGGATGAACAACATAAGCTTCTGGCTTCTCCCCCCTTCTTTCCTGCTACTCC
TCACTTCTTCCGGAGTAGAGGCAGGTGCCGGGACAGGGTGAACAGTCTACCCCCCGCTCGCTGGTAACCTCGCACACGCAGGT
GCTTCTGTCGACCTGGCTATCTTTTCCCTCCATCTCGCAGGTGTATCCTCGATTCTAGGGGCTATTAACTTTATTACAACGATTATTA
ATATGAAGCCCCCAGCAATTTCTCAGTACCAGACACCTTTATTCGTATGGGCCGTCTTAATTACTGCTGTCCTCCTACTACTCTCAC
TCCCTGTCCTAGCTGCCGGCATTACAATGCTTCTAACAGACCGCAACCTCAACACAACC

No.2025-166-24-10

TAGTAGGCACGTCTCTAAGCCTCCTAATCCGAGCAGAACTGAGTCAGCCCGGCTCACTCCTCGGAGATGACCAGATTTTTAACG
TAATTGTTACGGCACATGCCTTCGTTATAATTTTCTTTATAGTAATACCCGTTATGATCGGAGGGTTCGGAAACTGACTCGTTCCCC
TAATGATCGGAGCCCCCGACATGGCCTTCCCCCGAATGAACAATATGAGTTTCTGACTCCTTCCCCCTTCTTTCCTACTACTTCTT
GTCTCTTCGGCGGTAGAGGCAGGGGCCGGAACAGGGTGAACAGTCTACCCGCCACTCGCCGGAAATCTCGCACACGCAGGTG
CTTCTGTCGACTTAGCTATTTTCTCCCTTCATCTCGCAGGTGTATCCTCAATTCTGGGGGCCATTAATTTTATTACCACGATTATTAA
CATGAAACCCCCTGCGATTTCTCAGTACCAGACACCTTTGTTCGTATGGGCCGTCTTAATTACAGCGGTTCTCCTACTACTCTCAC
TTCCTGTCCTGGCTGCTGGCATTACAATGCTTCTAACAG

No.2025-166-24-11

CACATGCCTTCGTTATAATTTTCTTTATAGTAATACCCGTTATGATCGGAGGGTTCGGAAACTGACTCGTTCCCCTAATGATCGGA
GCCCCCGACATGGCCTTCCCCCGAATGAACAATATGAGTTTCTGACTCCTTCCCCCTTCTTTCCTACTACTTCTTGTCTCTTCGGC
GGTAGAGGCAGGGGCCGGAACAGGGTGAACAGTCTACCCGCCACTCGCCGGAAATCTCGCACACGCAGGTGCTTCTGTCGAC
TTAGCTATTTTCTCCCTTCATCTCGCAGGTGTATCCTCAATTCTGGGGGCCATTAATTTTATTACCACGATTATTAACATGAAACCCC
CTGCGATTTCTCAGTACCAGACACCTTTGTTCGTATGGGCCGTCTTAATTACAGCGGTTCTCCTACTACTCTCACTTCCTGTCCTG
GCTGCTGGCATTACAATGCTTCTAACAGATCGCAACCTTAACACAACCTTCTTCGACCCAGGGGGCGGAGGAGATCCGATTCTT
TATCAGCACCTATTCTGATTCTTTGACCCTGCA

No.2025-166-24-12

ATAGTAGGCACAGCCTTAAGCCTCCTAATCCGAGCAGAACTAAGTCAGCCAGGCTCACTCCTCGGAGATGACCAGATTTTTAAC
GTAATTGTTACGGCACATGCCTTTGTCATGATTTTCTTTATAGTAATACCCGTCATGATTGGAGGGTTCGGAAACTGACTTGTACC
CCTAATGATCGGAGCCCCCGACATGGCCTTTCCCCGGATGAACAACATAAGCTTCTGGCTTCTCCCCCCTTCTTTCCTGCTACTCC
TCACTTCTTCCGGAGTAGAGGCAGGTGCCGGGACAGGGTGAACAGTCTACCCCCCGCTCGCTGGTAATCTCGCACACGCAGGT
GCTTCTGTCGACCTGGCTATCTTTTCCCTCCATCTCGCAGGTGTATCCTCGATTCTAGGGGCTATTAACTTTATTACAACGATTATTA
ATATGAAGCCCCCAGCAATTTCTCAGTACCAGACGCCTTTATTCGTATGGGCCGTCTTAATTACTGCTGTCCTCCTACTACTCTCAC
TCCCTGTCCTAGCTGCCGGCATTACAATGC

No.2025-166-24-13

AATAGTAGGCACAGCCTTAAGCCTCCTAATCCGAGCAGAACTAAGTCAGCCAGGCTCACTCCTCGGAGATGACCAGATTTTTAA
CGTAATTGTTACGGCACATGCCTTTGTCATGATTTTCTTTATAGTAATACCCGTCATGATTGGAGGGTTCGGAAACTGACTTGTAC
CCCTAATGATCGGAGCCCCCGACATGGCCTTTCCCCGGATGAACAACATAAGCTTCTGGCTTCTCCCCCCTTCTTTCCTGCTACTC
CTCACTTCTTCCGGAGTAGAGGCAGGTGCCGGGACAGGGTGAACAGTCTACCCCCCGCTCGCTGGTAACCTCGCACACGCAG
GTGCTTCTGTCGACCTGGCTATCTTTTCCCTCCATCTCGCAGGTGTATCCTCGATTCTAGGGGCTATTAACTTTATTACAACGATTA
TTAATATGAAGCCCCCAGCAATTTCTCAGTACCAGACACCTTTATTCGTATGGGCCGTCTTAATTACTGCTGTCCTCCTACTACTCT
CACTCCCTGTCCTAGCTGCCGGCATTACAATGCTTCTAACAG

No.2025-166-24-14

AATAGTAGGCACAGCCTTAAGCCTCCTAATCCGAGCAGAACTAAGTCAGCCAGGCTCACTCCTCGGAGATGACCAGATTTTTAA
CGTAATTGTTACGGCACATGCCTTTGTCATGATTTTCTTTATAGTAATACCCGTCATGATTGGAGGGTTCGGAAACTGACTTGTAC
CCCTAATGATCGGAGCCCCCGACATGGCCTTTCCCCGGATGAACAACATAAGCTTCTGGCTTCTCCCCCCTTCTTTCCTGCTACTC
CTCACTTCTTCCGGAGTAGAGGCAGGTGCCGGGACAGGGTGAACAGTCTACCCCCCGCTCGCTGGTAACCTCGCACACGCAG
GTGCTTCTGTCGACCTGGCTATCTTTTCCCTCCATCTCGCAGGTGTATCCTCGATTCTAGGGGCTATTAACTTTATTACAACGATTA
TTAATATGAAGCCCCCAGCAATTTCTCAGTACCAGACACCTTTATTCGTATGGGCCGTCTTAATTACTGCTGTCCTCCTACTACTCT
CACTCCCTGTCCTAGCTGCCGGCATTACAATGCTTCTAACAG

No.2025-166-24-15

GAATAGTAGGCACAGCCTTAAGCCTCCTAATCCGAGCAGAACTAAGTCAGCCAGGCTCACTCCTCGGAGATGACCAGATTTTTA
ACGTAATTGTTACGGCACATGCCTTTGTCATGATTTTCTTTATAGTAATACCCGTCATGATTGGAGGGTTCGGAAACTGACTTGTA
CCCCTAATGATCGGAGCCCCCGACATGGCCTTTCCCCGGATGAACAACATAAGCTTCTGGCTTCTCCCCCCTTCTTTCCTGCTACT
CCTCACTTCTTCCGGAGTAGAGGCAGGTGCCGGGACAGGGTGAACAGTCTACCCCCCGCTCGCTGGTAATCTCGCACACGCAG
GTGCTTCTGTCGACCTGGCTATCTTTTCCCTCCATCTCGCAGGTGTGTCCTCGATTCTAGGGGCTATTAACTTTATTACAACGATTA
TTAATATGAAGCCCCCAGCAATTTCTCAGTACCAGACACCTTTATTCGTATGGGCCGTCTTAATTACTGCTGTCCTCCTACTACTCT
CACTCCCTGTCCTAGCTGCCGGCATTACAATGCTTCTAACAG

No.2025-166-24-16

AATAGTAGGCACAGCCTTAAGCCTCCTAATCCGAGCAGAACTAAGTCAGCCAGGCTCACTCCTCGGAGATGACCAGATTTTTAA

CGTAATTGTTACGGCACATGCCTTTGTCATGATTTTCTTTATAGTAATACCCGTCATGATTGGAGGGTTCGGAAACTGACTTGTAC
CCCTAATGATCGGAGCCCCCGACATGGCCTTTCCCCGGATGAACAACATAAGCTTCTGGCTTCTCCCCCCTTCTTTCCTGCTACTC
CTCACTTCTTCCGGAGTAGAGGCAGGTGCCGGGACAGGGTGAACAGTCTACCCCCCGCTCGCTGGTAACCTCGCACACGCAG
GTGCTTCTGTCGACCTGGCTATCTTTTCCCTCCATCTCGCAGGTGTATCCTCGATTCTAGGGGCTATTAACTTTATTACAACGATTA
TTAATATGAAGCCCCCAGCAATTTCTCAGTACCAGACACCTTTATTCGTATGGGCCGTCTTAATTACTGCTGTCCTCCTACTACTCT
CACTCCCTGTCCTAGCTGCCGGCATTACAATGCTTCTAACAG

No.2025-166-24-17

AATAGTAGGCACAGCCTTAAGCCTCCTAATCCGAGCAGAACTAAGTCAGCCAGGCTCACTCCTCGGAGATGACCAGATTTTTAA
CGTAATTGTTACGGCACATGCCTTTGTCATGATTTTCTTTATAGTAATACCCGTCATGATTGGAGGGTTCGGAAACTGACTTGTAC
CCCTAATGATCGGAGCCCCCGACATGGCCTTTCCCCGGATGAACAACATAAGCTTCTGGCTTCTCCCCCCTTCTTTCCTGCTACTC
CTCACTTCTTCCGGAGTAGAGGCAGGTGCCGGGACAGGGTGAACAGTCTACCCCCCGCTCGCTGGTAACCTCGCACACGCAG
GTGCTTCTGTCGACCTGGCTATCTTTTCCCTCCATCTCGCAGGTGTATCCTCGATTCTAGGGGCTATTAACTTTATTACAACGATTA
TTAATATGAAGCCCCCAGCAATTTCTCAGTACCAGACACCTTTATTCGTATGGGCCGTCTTAATTACTGCTGTCCTCCTACTACTCT
CACTCCCTGTCCTAGCTGCCGGCATTACAATGCTTCTAACAG

No.2025-166-24-18

TAGTAGGCACAGCCTTAAGCCTCCTAATCCGAGCAGAACTAAGTCAGCCAGGCTCACTCCTCGGAGATGACCAGATTTTTAACG
TAATTGTTACGGCACATGCCTTTGTCATGATTTTCTTTATAGTAATACCCGTCATGATTGGAGGGTTCGGAAACTGACTTGTACCC
CTAATGATCGGAGCCCCCGACATGGCCTTTCCCCGGATGAACAACATAAGCTTCTGGCTTCTCCCCCCTTCTTTCCTGCTACTCCT
CACTTCTTCCGGAGTAGAGGCAGGTGCCGGGACAGGGTGAACAGTCTACCCCCCGCTCGCTGGTAATCTCGCACACGCAGGT
GCTTCTGTCGACCTGGCTATCTTTTCCCTCCATCTCGCAGGTGTATCCTCGATTCTAGGGGCTATTAACTTTATTACAACGATTATTA
ATATGAAGCCCCCAGCAATTTCTCAGTACCAGACACCTTTATTCGTATGGGCCGTCTTAATTACTGCTGTCCTCCTACTACTCTCAC
TCCCTGTCCTAGCTGCCGGCATTACAATGCTTCTAACAGA

No.2025-166-24-19

ACAGCCTTAAGCCTCCTAATCCGAGCAGAACTAAGTCAGCCAGGCTCACTCCTCGGAGATGACCAGATTTTTAACGTAATTGTTA
CGGCACATGCCTTTGTCATGATTTTCTTTATAGTAATACCCGTCATGATTGGAGGGTTCGGAAACTGACTTGTACCCCTAATGATC
GGAGCCCCCGACATGGCCTTTCCCCGGATGAACAACATAAGCTTCTGGCTTCTCCCCCCTTCTTTCCTGCTACTCCTCACTTCTTC
CGGAGTAGAGGCAGGTGCCGGGACAGGGTGAACAGTCTACCCCCCGCTCGCTGGTAACCTCGCACACGCAGGTGCTTCTGTC
GACCTGGCTATCTTTTCCCTCCATCTCGCAGGTGTATCCTCGATTCTAGGGGCTATTAACTTTATTACAACGATTATTAATATGAAG
CCCCCAGCAATTTCTCAGTACCAGACACCTTTATTCGTATGGGCCGTCTTAATTACTGCTGTCCTCCTACTACTCTCACTCCCTGTC
CTAGCTGCCGGCATTACAATGCTTCTAACAGACCGCAACCTCAACACAACCTTCT

No.2025-166-24-20

GGTGCATGGGCCGGAATAGTAGGCACAGCCTTAAGCCTCCTAATCCGAGCAGAACTAAGTCAGCCAGGCTCACTCCTCGGAGA
TGACCAGATTTTTAACGTAATTGTTACGGCACATGCCTTTGTCATGATTTTCTTTATAGTAATACCCGTCATGATTGGAGGGTTCG
GAAACTGACTTGTACCCCTAATGATCGGAGCCCCCGACATGGCCTTTCCCCGGATGAACAACATAAGCTTCTGGCTTCTCCCCCC
TTCTTTCCTGCTACTCCTCACTTCTTCCGGAGTAGAGGCAGGTGCCGGGACAGGGTGAACAGTCTACCCCCCGCTCGCTGGTAA
CCTCGCACACGCAGGTGCTTCTGTCGACCTGGCTATCTTTTCCCTCCATCTCGCAGGTGTATCCTCGATTCTAGGGGCTATTAACT
TTATTACAACGATTATTAATATGAAGCCCCCAGCAATTTCTCAGTACCAGACACCTTTATTCGTATGGGCCGTCTTAATTACTGCTG
TCCTCCTACTACTCTCACT-CCCTGTCCTAGCTGCCGGCA

No.2025-166-24-21

CCGGAATAGTAGGCACAGCCTTAAGCCTCCTAATCCGAGCAGAACTAAGTCAGCCAGGCTCACTCCTCGGAGATGACCAGATTT
TTAACGTAATTGTTACGGCACATGCCTTTGTCATGATTTTCTTTATAGTAATACCCGTCATGATTGGAGGGTTCGGAAACTGACTT
GTACCCCTAATGATCGGAGCCCCCGACATGGCCTTTCCCCGGATGAACAACATAAGCTTCTGGCTTCTCCCCCCTTCTTTCCTGCT
ACTCCTCACTTCTTCCGGAGTAGAGGCAGGTGCCGGGACAGGGTGAACAGTCTACCCCCCGGTCGCTGGTAACCTCGCACACG
CAGGTGCTTCTGTCGACCTGGCTATCTTTTCCCTCCATCTCGCAGGTGTATCCTCGATTCTAGGGGCTATTAACTTTATTACAACGA
TTATTAATATGAAGCCCCCAGCAATTTCTCAGTACCAGACACCTTTATTCGTATGGGCCGTCTTAATTACTGCTGTCCTCCTACTACT
CTCACTCCCTGTCCTAGCTGCCGGCATTACAA

No.2025-166-24-22

GCCTTAAGCCTCCTAATCCGAGCAGAACTAAGTCAGCCAGGCTCACTCCTCGGAGATGACCAGATTTTTAACGTAATTGTTACG
GCACATGCCTTTGTCATGATTTTCTTTATAGTAATACCCGTCATGATTGGAGGGTTCGGAAACTGACTTGTACCCCTAATGATCGG
AGCCCCCGACATGGCCTTTCCCCGGATGAACAACATAAGCTTCTGGCTTCTCCCCCCTTCTTTCCTGCTACTCCTCACTTCTTCCG
GAGTAGAGGCAGGTGCCGGGACAGGGTGAACAGTCTACCCCCCGCTCGCTGGTAACCTCGCACACGCAGGTGCTTCTGTCGA
CCTGGCTATCTTTTCCCTCCATCTCGCAGGTGTATCCTCGATTCTAGGGGCTATTAACTTTATTACAACGATTATTAATATGAAGCCC
CCAGCAATTTCTCAGTACCAGACACCTTTATTCGTATGGGCCGTCTTAATTACTGCTGTCCTCCTACTACTCTCACTCCCTGTCCTA
GCTGCCGGCATTACAATGCTTCTAACAGACCGCAACCTCAACACAACCTTCTTT

No.2025-166-24-23

AATAGTAGGCACAGCCTTAAGCCTCCTAATCCGAGCAGAACTAAGTCAGCCAGGCTCACTCCTCGGAGATGACCAGATTTTTAA
CGTAATTGTTACGGCACATGCCTTTGTCATGATTTTCTTTATAGTAATACCCGTCATGATTGGAGGGTTCGGAAACTGACTTGTAC
CCCTAATGATCGGAGCCCCCGACATGGCCTTTCCCCGGATGAACAACATAAGCTTCTGGCTTCTCCCCCCTTCTTTCCTGCTACTC
CTCACTTCTTCCGGAGTAGAGGCAGGTGCCGGGACAGGGTGAACAGTCTACCCCCCGCTCGCTGGTAACCTCGCACACGCAG
GTGCTTCTGTCGACCTGGCTATCTTTTCCCTCCATCTCGCAGGTGTATCCTCGATTCTAGGGGCTATTAACTTTATTACAACGATTA
TTAATATGAAGCCCCCAGCAATTTCTCAGTACCAGACACCTTTATTCGTATGGGCCGTCTTAATTACTGCTGTCCTCCTACTACTCT
CACTCCCTGTCCTAGCTGCCGGCATTACAATGCTTCTAACA

No.2025-166-24-24

ATAGTAGGCACAGCCTTAAGCCTCCTAATCCGAGCAGAACTAAGTCAGCCAGGCTCACTCCTCGGAGATGACCATATTTTTAACG
TAATTGTTACGGCACATGCCTTTGTCATGATTTTCTTTATAGTAATACCCGTCATGATTGGAGGGTTCGGAAACTGACTTGTACCC
CTAATGATCGGAGCCCCCGACATGGCCTTTCCCCGGATGAACAACATAAGCTTCTGGCTTCTCCCCCCTTCTTTCCTGCTACTCCT
CACTTCTTCCGGAGTAGAGGCAGGTGCCGGGACAGGGTGAACAGTCTACCCCCCGCTCGCTGGTAATCTCGCACACGCAGGT
GCTTCTGTCGACCTGGCTATCTTTTCCCTCCATCTCGCAGGTGTATCCTCGATTCTAGGGGCTATTAACTTTATTACAACGATTATTA
ATATGAAGCCCCCAGCAATTTCTCAGTACCAGACGCCTTTATTCGTATGGGCCGTCTTAATTACTGCTGTCCTCCTACTACTCTCAC
TCCCTGTCCTAGCTGCCGGCATTACAATGCTTCTAACAGACCGCAACCTCAACACAACCTTCTTT

Fish maw: COI gene - Small-sized cylindrical samples (n=57)

No.2025-166-25-1

ATAGTAGGCACAGCCTTAAGCCTCCTAATCCGAGCAGAACTAAGTCAGCCAGGCTCACTCCTCGGAGATGACCAGATTTTTAAC
GTAATTGTTACGGCACATGCCTTTGTCATGATTTTCTTTATAGTAATACCCGTCATGATTGGAGGGTTCGGAAACTGACTTGTACC
CCTAATGATCGGAGCCCCCGACATGGCCTTTCCCCGGATGAACAACATAAGCTTCTGGCTTCTCCCCCCTTCTTTCCTGCTACTCC
TCACTTCTTCCGGAGTAGAGGCAGGTGCCGGGACAGGGTGAACAGTCTACCCCCCGCTCGCTGGTAACCTCGCACACGCAGGT
GCTTCTGTCGACCTGGCTATCTTTTCCCTCCATCTCGCAGGTGTATCCTCGATTCTAGGGGCTATTAACTTTATTACAACGATTATTA
ATATGAAGCCCCCAGCAATTTCTCAGTACCAGACACCTTTATTCGTATGGGCCGTCTTAATTACTGCTGTCCTCCTACTACTCTCAC
TCCCTGTCCTAGCTGCCGGCATTACAA

No.2025-166-25-2

ATAGTAGGCACAGCCTTAAGCCTCCTAATCCGAGCAGAACTAAGTCAGCCAGGCTCACTCCTCGGAGATGACCAGATTTTTAAC
GTAATTGTTACGGCACATGCCTTTGTCATGATTTTCTTTATAGTAATACCCGTCATGATTGGAGGGTTCGGAAACTGACTTGTACC
CCTAATGATCGGAGCCCCCGACATGGCCTTTCCCCGGATGAACAACATAAGCTTCTGGCTTCTCCCCCCTTCTTTCCTGCTACTCC
TCACTTCTTCCGGAGTAGAGGCAGGTGCCGGGACAGGGTGAACAGTCTACCCCCCGCTCGCTGGTAACCTCGCACACGCAGGT
GCTTCTGTCGACCTGGCTATCTTTTCCCTCCATCTCGCAGGTGTATCCTCGATTCTAGGGGCTATTAACTTTATTACAACGATTATTA
ATATGAAGCCCCCAGCAATTTCTCAGTACCAGACACCTTTATTCGTATGGGCCGTCTTAATTACTGCTGTCCTCCTACTACTCTCAC
TCCCTGTCCTAGCTGCCGGCAT

No.2025-166-25-3

GTAGGCACAGCCTTAAGCCTCCTAATCCGAGCAGAACTAAGTCAGCCAGGCTCACTCCTCGGAGATGACCAGATTTTTAACGTA
ATTGTTACGGCACATGCCTTTGTCATGATTTTCTTTATAGTAATACCCGTCATGATTGGAGGGTTCGGAAACTGACTTGTACCCCT
AATGATCGGAGCCCCCGACATGGCCTTTCCCCGGATGAACAACATAAGCTTCTGGCTTCTCCCCCCTTCTTTCCTGCTACTCCTCA
CTTCTTCCGGAGTAGAGGCAGGTGCCGGGACAGGGTGAACAGTCTACCCCCCGCTCGCTGGTAATCTCGCACACGCAGGTGCT
TCTGTCGACCTGGCTATCTTTTCCCTCCATCTCGCAGGTGTATCCTCGATTCTAGGGGCTATTAACTTTATTACAACGATTATTAATA
TGAAGCCCCCAGCAATTTCTCAGTACCAGACACCTTTATTCGTATGGGCCGTCTTAATTACTGCTGTCCTCCTACTACTCTCA

No.2025-166-25-4

TCCTCGGAGATGACCAGATTTTTAACGTAATTGTTACGGCACATGCCTTCGTTATAATTTTCTTTATAGTAATACCCGTTATGATCG
GAGGGTTCGGAAACTGACTCGTTCCCCTAATGATCGGAGCCCCCGACATGGCCTTCCCCCGAATGAACAATATGAGTTTCTGAC
TCCTTCCCCCTTCTTTCCTACTACTTCTTGTCTCTTCGGCGGTAGAGGCAGGGGCCGGAACAGGGTGAACAGTCTACCCGCCAC
TCGCCGGAAATCTCGCACACGCAGGTGCTTCTGTCGACTTAGCTATTTTCTCCCTTCATCTCGCAGGTGTATCCTCAATTCTGGGG
GCCATTAATTTTATTACCACGATTATTAACATGAAACCCCCTGCGATTTCTCAGTACCAGACACCTTTGTTCGTATGGGCCGTCTTA
ATTACAGCGGTTCTCCTACTACTCTCACTTCCTGTCCTGGCTGCTGGCATTACAATGCTTCTAACAGATCGCAACCTTAACACAAC
CTTCTTCGACCCAGGGGGCGGAGGAGATCCGATTCTTTATCAGCACCTATTCTGATTCTTTGACCCTTGCAGG

No.2025-166-25-5

AGCCTTAAGCCTCCTAATCCGAGCAGAACTAAGTCAGCCAGGCTCACTCCTCGGAGATGACCAGATTTTTAACGTAATTGTTACG
GCACATGCCTTTGTCATGATTTTCTTTATAGTAATACCCGTCATGATTGGAGGGTTCGGAAACTGACTTGTACCCCTAATGATCGG
AGCCCCCGACATGGCCTTTCCCCGGATGAACAACATAAGCTTCTGGCTTCTCCCCCCTTCTTTCCTGCTACTCCTCACTTCTTCCG
GAGTAGAGGCAGGTGCCGGGACAGGGTGAACAGTCTACCCCCCGCTCGCTGGTAATCTCGCACACGCAGGTGCTTCTGTCGA
CCTGGCTATCTTTTCCCTCCATCTCGCAGGTGTATCCTCGATTCTAGGGGCTATTAACTTTATTACAACGATTATTAATATGAAGCCC
CCAGCAATTTCTCAGTACCAGACACCTTTATTCGTATGGGCCGTCTTAATTACTGCTGTCCTCCTACTACTCTCACTCCCTGTCCTA
GCTGCCGGCATTACAATGCT

No.2025-166-25-6

AATAGTAGGCACGTCTCTAAGCCTCCTAATCCGAGCAGAACTGAGTCAGCCCGGCTCACTCCTCGGAGATGACCAGATTTTTAA
CGTAATTGTTACGGCACATGCCTTCGTTATAATTTTCTTTATAGTAATACCCGTTATGATCGGAGGGTTCGGAAACTGACTCGTTCC
CCTAATGATCGGAGCCCCCGACATGGCCTTCCCCCGAATGAACAATATGAGTTTCTGACTCCTTCCCCCTTCTTTCCTACTACTTCT
TGTCTCTTCGGCGGTAGAGGCAGGGGCCGGAACAGGGTGAACAGTCTACCCGCCACTCGCCGGAAATCTCGCACACGCAGGT
GCTTCTGTCGACTTAGCTATTTTCTCCCTTCATCTCGCAGGTGTATCCTCAATTCTGGGGGCCATTAATTTTATTACCACGATTATTA
ACATGAAACCCCCTGCGATTTCTCAGTACCAGACACCTTTGTTCGTATGGGCCGTCTTAATTACAGCGGTTCTCCTACTACTCTCA
CTTCCTGTCCTGGCTGCTGGCATTACAATGCTTCTAACAG

No.2025-166-25-7

AATAGTAGGCACAGCCTTAAGCCTCCTAATCCGAGCAGAACTAAGTCAGCCAGGCTCACTCCTCGGAGATGACCAGATTTTTAA
CGTAATTGTTACGGCACATGCCTTTGTCATGATTTTCTTTATAGTAATACCCGTCATGATTGGAGGGTTCGGAAACTGACTTGTAC
CCCTAATGATCGGAGCCCCCGACATGGCCTTTCCCCGGATGAACAACATAAGCTTCTGGCTTCTCCCCCCTTCTTTCCTGCTACTC
CTCACTTCTTCCGGAGTAGAGGCAGGTGCCGGGACAGGGTGAACAGTCTACCCCCCGCTCGCTGGTAACCTCGCACACGCAG
GTGCTTCTGTCGACCTGGCTATCTTTTCCCTCCATCTCGCAGGTGTATCCTCGATTCTAGGGGCTATTAACTTTATTACAACGATTA
TTAATATGAAGCCCCCAGCAATTTCTCAGTACCAGACACCTTTATTCGTATGGGCCGTCTTAATTACTGCTGTCCTCCTACTACTCT
CACTCCCTGTCCTAGCTGCCGGCATTACAATGCTTCTA

No.2025-166-25-8

AATAGTAGGCACGTCTCTAAGCCTCCTAATCCGAGCAGAACTGAGTCAGCCCGGCTCACTCCTCGGAGATGACCAGATTTTTAA
CGTAATTGTTACGGCACATGCCTTCGTTATAATTTTCTTTATAGTAATACCCGTTATGATCGGAGGGTTCGGAAACTGACTCGTTCC
CCTAATGATCGGAGCCCCCGACATGGCCTTCCCCCGAATGAACAATATGAGTTTCTGACTCCTTCCCCCTTCTTTCCTACTACTTCT
TGTCTCTTCGGCGGTAGAGGCAGGGGCCGGAACAGGGTGAACAGTCTACCCGCCACTCGCCGGAAATCTCGCACACGCAGGT

GCTTCTGTCGACTTAGCTATTTTCTCCCTTCATCTCGCAGGTGTATCCTCAATTCTGGGGGCCATTAATTTTATTACCACGATTATTA
ACATGAAACCCCCTGCGATTTCTCAGTACCAGACACCTTTGTTCGTATGGGCCGTCTTAATTACAGCGGTTCTCCTACTACTCTCA
CTTCCTGTCCTGGCTGCTGGCATTACAATGCTTCT

No.2025-166-25-9

TAGTAGGCACGTCTCTAAGCCTCCTAATCCGAGCAGAACTGAGTCAGCCCGGCTCACTCCTCGGAGATGACCAGATTTTTAACG
TAATTGTTACGGCACATGCCTTCGTTATAATTTTCTTTATAGTAATACCCGTTATGATCGGAGGGTTCGGAAACTGACTCGTTCCCC
TAATGATCGGAGCCCCCGACATGGCCTTCCCCCGAATGAACAATATGAGTTTCTGACTCCTTCCCCCTTCTTTCCTACTACTTCTT
GTCTCTTCGGCGGTAGAGGCAGGGGCCGGAACAGGGTGAACAGTCTACCCGCCACTCGCCGGAAATCTCGCACACGCAGGTG
CTTCTGTCGACTTAGCTATTTTCTCCCTTCATCTCGCAGGTGTATCCTCAATTCTGGGGGCCATTAATTTTATTACCACGATTATTAA
CATGAAACCCCCTGCGATTTCTCAGTACCAGACACCTTTGTTCGTATGGGCCGTCTTAATTACAGCGGTTCTCCTACTACTCTCAC
TTCCTGTCCTGGCTGCTGGCATTACAATGCTTCT

No.2025-166-25-10

ATAGTAGGCACGTCTCTAAGCCTCCTAATCCGAGCAGAACTGAGTCAGCCCGGCTCACTCCTCGGAGATGACCAGATTTTTAAC
GTAATTGTTACGGCACATGCCTTCGTTATAATTTTCTTTATAGTAATACCCGTTATGATCGGAGGGTTCGGAAACTGACTCGTTCCC
CTAATGATCGGAGCCCCCGACATGGCCTTCCCCCGAATGAACAATATGAGTTTCTGACTCCTTCCCCCTTCTTTCCTACTACTTCTT
GTCTCTTCGGCGGTAGAGGCAGGGGCCGGAACAGGGTGAACAGTCTACCCGCCACTCGCCGGAAATCTCGCACACGCAGGTG
CTTCTGTCGACTTAGCTATTTTCTCCCTTCATCTCGCAGGTGTATCCTCAATTCTGGGGGCCATTAATTTTATTACCACGATTATTAA
CATGAAACCCCCTGCGATTTCTCAGTACCAGACACCTTTGTTCGTATGGGCCGTCTTAATTACAGCGGTTCTCCTACTACTCTCAC
TTCCTGTCCTGGCTGCTGGCATTACAATGCTTCTAACAGATCGCAACCTTAACACAACCTTCTTCGACCCAGGGGGCGGAGGAG
ATCCGAT

No.2025-166-25-11

GAATAGTAGGCACGTCTCTAAGCCTCCTAATCCGAGCAGAACTGAGTCAGCCCGGCTCACTCCTCGGAGATGACCAGATTTTTA
ACGTAATTGTTACGGCACATGCCTTCGTTATAATTTTCTTTATAGTAATACCCGTTATGATCGGAGGGTTCGGAAACTGACTCGTTC
CCCTAATGATCGGAGCCCCCGACATGGCCTTCCCCCGAATGAACAATATGAGTTTCTGACTCCTTCCCCCTTCTTTCCTACTACTT
CTTGTCTCTTCGGCGGTAGAGGCAGGGGCCGGAACAGGGTGAACAGTCTACCCGCCACTCGCCGGAAATCTCGCACACGCAG
GTGCTTCTGTCGACTTAGCTATTTTCTCCCTTCATCTCGCAGGTGTATCCTCAATTCTGGGGGCCATTAATTTTATTACCACGATTAT
TAACATGAAACCCCCTGCGATTTCTCAGTACCAGACACCTTTGTTCGTATGGGCCGTCTTAATTACAGCGGTTCTCCTACTACTCT
CACTTCCTGTCCTGGCTGCTGGCATTACAATGCTTCT

No.2025-166-25-12

TCGGTGCATGAGCCGGAATAGTAGGCACGTCTCTAAGCCTCCTAATCCGAGCAGAACTGAGTCAGCCCGGCTCACTCCTCGGA
GATGACCAGATTTTTAACGTAATTGTTACGGCACATGCCTTCGTTATAATTTTCTTTATAGTAATACCCGTTATGATCGGAGGGTTC
GGAAACTGACTCGTTCCCCTAATGATCGGAGCCCCCGACATGGCCTTCCCCCGAATGAACAATATGAGTTTCTGACTCCTTCCCC
CTTCTTTCCTACTACTTCTTGTCTCTTCGGCGGTAGAGGCAGGGGCCGGAACAGGGTGAACAGTCTACCCGCCACTCGCCGGA
AATCTCGCACACGCAGGTGCTTCTGTCGACTTAGCTATTTTCTCCCTTCATCTCGCAGGTGTATCCTCAATTCTGGGGGCCATTAA
TTTTATTACCACGATTATTAACATGAAACCCCCTGCGATTTCTCAGTACCAGACACCTTTGTTCGTATGGGCCGTCTTAATTACAGC
GGTTCTCCTACTACTCTCACTTCCTGTCCTGGCTGCTGGCATTACAATGCTTCTAACAGATCGCAACCTTAACACAACCTTCTTCG
ACCCGGCGGGCGGAGGAGATCCGATTCTTTATCAGCACCTATTCTGATTCTTTGA

No.2025-166-25-13

ATAGTAGGCACAGCCTTAAGCCTCCTAATCCGAGCAGAACTAAGTCAGCCAGGCTCACTCCTCGGAGATGACCAGATTTTTAAC
GTAATTGTTACGGCACATGCCTTTGTCATGATTTTCTTTATAGTAATACCCGTCATGATTGGAGGGTTCGGAAACTGACTTGTACC
CCTAATGATCGGAGCCCCCGACATGGCCTTTCCCCGGATGAACAACATAAGCTTCTGGCTTCTCCCCCCTTCTTTCCTGCTACTCC
TCACTTCTTCCGGAGTAGAGGCAGGTGCCGGGACAGGGTGAACAGTCTACCCCCCGCTCGCTGGTAACCTCGCACACGCAGGT
GCTTCTGTCGACCTGGCTATCTTTTCCCTCCATCTCGCAGGTGTATCCTCGATTCTAGGGGCTATTAACTTTATTACAACGATTATTA
ATATGAAGCCCCCAGCAATTTCTCAGTACCAGACACCTTTATTCGTATGGGCCGTCTTAATTACTGCTGTCCTCCTACTACTCTCAC
TCCCTGTCCTAGCTGCCGGCATTACAATGCTTCTAACAGACC

No.2025-166-25-14

GGTGCATGAGCCGGAATAGTAGGCACGTCTCTAAGCCTCCTAATCCGAGCAGAACTGAGTCAGCCCGGCTCACTCCTCGGAGA
TGACCAGATTTTTAACGTAATTGTTACGGCACATGCCTTCGTTATAATTTTCTTTATAGTAATACCCGTTATGATCGGAGGGTTCGG
AAACTGACTCGTTCCCCTAATGATCGGAGCCCCCGACATGGCCTTCCCCCGAATGAACAATATGAGTTTCTGACTCCTTCCCCCTT
CTTTCCTACTACTTCTTGTCTCTTCGGCGGTAGAGGCAGGGGCCGGAACAGGGTGAACAGTCTACCCGCCACTCGCCGGAAAT
CTCGCACACGCAGGTGCTTCTGTCGACTTAGCTATTTTCTCCCTTCATCTCGCAGGTGTATCCTCAATTCTGGGGGCCATTAATTT
TATTACCACGATTATTAACATGAAACCCCCTGCGATTTCTCAGTACCAGACACCTTTGTTCGTATGGGCCGTCTTAATTACAGCGG
TTCTCCTACTACTCTCACTTCCTGTCCTGGCTGCTGGCATTACAATGCTTCTAACAGATCGCAACCTTAACACAACCTTCTTCGACC
CAGGGGGCGGAGGAGATCCGAT

No.2025-166-25-15

TAGTAGGCACGTCTCTAAGCCTCCTAATCCGAGCAGAACTGAGTCAGCCCGGCTCACTCCTCGGAGATGACCAGATTTTTAACG
TAATTGTTACGGCACATGCCTTCGTTATAATTTTCTTTATAGTAATACCCGTTATGATCGGAGGGTTCGGAAACTGACTCGTTCCCC
TAATGATCGGAGCCCCCGACATGGCCTTCCCCCGAATGAACAATATGAGTTTCTGACTCCTTCCCCCTTCTTTCCTACTACTTCTT
GTCTCTTCGGCGGTAGAGGCAGGGGCCGGAACAGGGTGAACAGTCTACCCGCCACTCGCCGGAAATCTCGCACACGCAGGTG
CTTCTGTCGACTTAGCTATTTTCTCCCTTCATCTCGCAGGTGTATCCTCAATTCTGGGGGCCATTAATTTTATTACCACGATTATTAA
CATGAAACCCCCTGCGATTTCTCAGTACCAGACACCTTTGTTCGTATGGGCCGTCTTAATTACAGCGGTTCTCCTACTACTCTCAC
TTCCTGTCCTGGCTGCTGGCATTACAATGCTTCT

No.2025-166-25-16

ATAGTAGGCACAGCCTTAAGCCTCCTAATCCGAGCAGAACTAAGTCAGCCAGGCTCACTCCTCGGAGATGACCAGATTTTTAAC
GTAATTGTTACGGCACATGCCTTTGTCATGATTTTCTTTATAGTAATACCCGTCATGATTGGAGGGTTCGGAAACTGACTTGTACC
CCTAATGATCGGAGCCCCCGACATGGCCTTTCCCCGGATGAACAACATAAGCTTCTGGCTTCTCCCCCCTTCTTTCCTGCTACTCC
TCACTTCTTCCGGAGTAGAGGCAGGTGCCGGGACAGGGTGAACAGTCTACCCCCCGCTCGCTGGTAATCTCGCACACGCAGGT
GCTTCTGTCGACCTGGCTATCTTTTCCCTCCATCTCGCAGGTGTATCCTCGATTCTAGGGGCTATTAACTTTATTACAACGATTATTA
ATATGAAGCCCCCAGCAATTTCTCAGTACCAGACGCCTTTATTCGTATGGGCCGTCTTAATTACTGCTGTCCTCCTACTACTCTCAC
TCCCTGTCCTAGCTGCCGGCATTACAATGCTTCTAACAG

No.2025-166-25-17

AATAGTAGGCACGTCTCTAAGCCTCCTAATCCGAGCAGAACTGAGTCAGCCCGGCTCACTCCTCGGAGATGACCAGATTTTTAA
CGTAATTGTTACGGCACATGCCTTCGTTATAATTTTCTTTATAGTAATACCCGTTATGATCGGAGGGTTCGGAAACTGACTCGTTCC
CCTAATGATCGGAGCCCCCGACATGGCCTTCCCCCGAATGAACAATATGAGTTTCTGACTCCTTCCCCCTTCTTTCCTGCTACTTC
TTGTCTCTTCGGCGGTAGAGGCAGGGGCCGGAACAGGGTGAACAGTCTACCCGCCACTCGCCGGAAATCTCGCACACGCAGG
TGCTTCTGTCGACTTAGCTATTTTCTCCCTTCATCTCGCAGGTGTATCCTCAATTCTGGGGGCCATTAATTTTATTACCACGATTATT
AACATGAAACCCCCTGCGATTTCTCAGTACCAGACACCTTTGTTCGTATGGGCCGTCTTAATTACAGCGGTTCTCCTACTACTCTC
ACTTCCTGTCCTGGCTGCTGGCATTACAATGCTTCTAACA

No.2025-166-25-18

AATAGTAGGCACAGCCTTAAGCCTCCTAATCCGAGCAGAACTAAGTCAGCCAGGCTCACTCCTCGGAGATGACCAGATTTTTAA
CGTAATTGTTACGGCACATGCCTTTGTCATGATTTTCTTTATAGTAATACCCGTCATGATTGGAGGGTTCGGAAACTGACTTGTAC
CCCTAATGATCGGAGCCCCCGACATGGCCTTTCCCCGGATGAACAACATAAGCTTCTGGCTTCTCCCCCCTTCTTTCCTGCTACTC
CTCACTTCTTCCGGAGTAGAGGCAGGTGCCGGGACAGGGTGAACAGTCTACCCCCCGCTCGCTGGTAACCTCGCACACGCAG
GTGCTTCTGTCGACCTGGCTATCTTTTCCCTCCATCTCGCAGGTGTATCCTCGATTCTAGGGGCTATTAACTTTATTACAACGATTA
TTAATATGAAGCCCCCAGCAATTTCTCAGTACCAGACACCTTTATTCGTATGGGCCGTCTTAATTACTGCTGTCCTCCTACTAC

No.2025-166-25-19

AATAGTAGGCACGTCTCTAAGCCTCCTAATCCGAGCAGAACTGAGTCAGCCCGGCTCACTCCTCGGAGATGACCAGATTTTTAA
CGTAATTGTTACGGCACATGCCTTCGTTATAATTTTCTTTATAGTAATACCCGTTATGATCGGAGGGTTCGGAAACTGACTCGTTCC
CCTAATGATCGGAGCCCCCGACATGGCCTTCCCCCGAATGAACAATATGAGTTTCTGACTCCTTCCCCCTTCTTTCCTACTACTTCT
TGTCTCTTCGGCGGTAGAGGCAGGGGCCGGAACAGGGTGAACAGTCTACCCGCCACTCGCCGGAAATCTCGCACACGCAGGT
GCTTCTGTCGACTTAGCTATTTTCTCCCTTCATCTCGCAGGTGTATCCTCAATTCTGGGGGCCATTAATTTTATTACCACGATTATTA
ACATGAAACCCCCTGCGATTTCTCAGTACCAGACACCTTTGTTCGTATGGGCCGTCTTAATTACAGCGGTTCTCCTACTACTCTCA
CTTCCTGTCCTGGCTGCTGGCATTACAATGCTTCTAA

No.2025-166-25-20

AATAGTAGGCACAGCCTTAAGCCTCCTAATCCGAGCAGAACTAAGTCAGCCAGGCTCACTCCTCGGAGATGACCAGATTTTTAA
CGTAATTGTTACGGCACATGCCTTTGTCATGATTTTCTTTATAGTAATACCCGTCATGATTGGAGGGTTCGGAAACTGACTTGTAC
CCCTAATGATCGGAGCCCCCGACATGGCCTTTCCCCGGATGAACAACATAAGCTTCTGGCTTCTCCCCCCTTCTTTCCTGCTACTC
CTCACTTCTTCCGGAGTAGAGGCAGGTGCCGGGACAGGGTGAACAGTCTACCCCCCGCTCGCTGGTAACCTCGCACACGCAG
GTGCTTCTGTCGACCTGGCTATCTTTTCCCTCCATCTCGCAGGTGTATCCTCGATTCTAGGGGCTATTAACTTTATTACAACGATTA
TTAATATGAAGCCCCCAGCAATTTCTCAGTACCAGACACCTTTATTCGTATGGGCCGTCTTAATTACTGCTGTCCTCCTACTACTCT
CACTCCCTGTCCTAGCTGCCGGCATTACAATGCTTCTAACA

No.2025-166-25-21

CGGTGCATGAGCCGGAATAGTAGGCACGTCTCTAAGCCTCCTAATCCGAGCAGAACTGAGTCAGCCCGGCTCACTCCTCGGAG
ATGACCAGATTTTTAACGTAATTGTTACGGCACATGCCTTCGTTATAATTTTCTTTATAGTAATACCCGTTATGATCGGAGGGTTCG
GAAACTGACTCGTTCCCCTAATGATCGGAGCCCCCGACATGGCCTTCCCCCGAATGAACAATATGAGTTTCTGACTCCTTCCCCC
TTCTTTCCTACTACTTCTTGTCTCTTCGGCGGTAGAGGCAGGGGCCGGAACAGGGTGAACAGTCTACCCGCCACTCGCCGGAA
ATCTCGCACACGCAGGTGCTTCTGTCGACTTAGCTATTTTCTCCCTTCATCTCGCAGGTGTATCCTCAATTCTGGGGGCCATTAAT
TTTATTACCACGATTATTAACATGAAACCCCCTGCGATTTCTCAGTACCAGACACCTTTGTTCGTATGGGCCGTCTTAATTACAGCG
GTTCTCCTACTACTCTCACTTCCTGTCCTGGCTGCTGGCATTACAATGCTTCTAACAGATCGCAACCTTAACACAACCTTCTTCGA
CCCAGGGGGCGGAGGAGATCCGAT

No.2025-166-25-22

AATAGTAGGCACGTCTCTAAGCCTCCTAATCCGAGCAGAACTGAGTCAGCCCGGCTCACTCCTCGGAGATGACCAGATTTTTAA
CGTAATTGTTACGGCACATGCCTTCGTTATAATTTTCTTTATAGTAATACCCGTTATGATCGGAGGGTTCGGAAACTGACTCGTTCC
CCTAATGATCGGAGCCCCCGACATGGCCTTCCCCCGAATGAACAATATGAGTTTCTGACTCCTTCCCCCTTCTTTCCTACTACTTCT
TGTCTCTTCGGCGGTAGAGGCAGGGGCCGGAACAGGGTGAACAGTCTACCCGCCACTCGCCGGAAATCTCGCACACGCAGGT
GCTTCTGTCGACTTAGCTATTTTCTCCCTTCATCTCGCAGGTGTATCCTCAATTCTGGGGGCCATTAATTTTATTACCACGATTATTA
ACATGAAACCCCCTGCGATTTCTCAGTACCAGACACCTTTGTTCGTATGGGCCGTCTTAATTACAGCGGTTCTCCTACTACTCTCA
CTTCCTGTCCTGGCTGCTGGCATTACAATGCTTCTAA

No.2025-166-25-23

GGAATAGTAGGCACAGCCTTAAGCCTCCTAATCCGAGCAGAACTAAGTCAGCCAGGCTCACTCCTCGGAGATGACCAGATTTTT
AACGTAATTGTTACGGCACATGCCTTTGTCATGATTTTCTTTATAGTAATACCCGTCATGATTGGAGGGTTCGGAAACTGACTTGT
ACCCCTAATGATCGGAGCCCCCGACATGGCCTTTCCCCGGATGAACAACATAAGCTTCTGGCTTCTCCCCCCTTCTTTCCTGCTAC
TCCTCACTTCTTCCGGAGTAGAGGCAGGTGCCGGGACAGGGTGAACAGTCTACCCCCCGCTCGCTGGTAACCTCGCACACGCA
GGTGCTTCTGTCGACCTGGCTATCTTTTCCCTCCATCTCGCAGGTGTATCCTCGATTCTAGGGGCTATTAACTTTATTACAACGATT
ATTAATATGAAGCCCCCAGCAATTTCTCAGTACCAGACACCTTTATTCGTATGGGCCGTCTTAATTACTGCTGTCCTCCTACTACTC
TCACT

No.2025-166-25-24

TAGTAGGCACAGCCTTAAGCCTCCTAATCCGAGCAGAACTAAGTCAGCCAGGCTCACTCCTCGGAGATGACCAGATTTTTAACG
TAATTGTTACGGCACATGCCTTTGTCATGATTTTCTTTATAGTAATACCCGTCATGATTGGAGGGTTCGGAAACTGACTTGTACCC
CTAATGATCGGAGCCCCCGACATGGCCTTTCCCCGGATGAACAACATAAGCTTCTGGCTTCTCCCCCCTTCTTTCCTGCTACTCCT
CACTTCTTCCGGAGTAGAGGCAGGTGCCGGGACAGGGTGAACAGTCTACCCCCCGCTCGCTGGTAACCTCGCACACGCAGGT
GCTTCTGTCGACCTGGCTATCTTTTCCCTCCATCTCGCAGGTGTATCCTCGATTCTAGGGGCTATTAACTTTATTACAACGATTATTA
ATATGAAGCCCCCAGCAATTTCTCAGTACCAGACACCTTTATTCGTATGGGCCGTCTTAATTACTGCTGTCCTCCTACTACTCTCAC
TCCCTGTCCTAGCTGCCGG

No.2025-166-25-25

GGAATAGTAGGCACGTCTCTAAGCCTCCTAATCCGAGCAGAACTGAGTCAGCCCGGCTCACTCCTCGGAGATGACCAGATTTTT
AACGTAATTGTTACGGCACATGCCTTCGTTATAATTTTCTTTATAGTAATACCCGTTATGATCGGAGGGTTCGGAAACTGACTCGT
TCCCCTAATGATCGGAGCCCCCGACATGGCCTTCCCCCGAATGAACAATATGAGTTTCTGACTCCTTCCCCCTTCTTTCCTACTAC
TTCTTGTCTCTTCGGCGGTAGAGGCAGGGGCCGGAACAGGGTGAACAGTCTACCCGCCACTCGCCGGAAATCTCGCACACGC
AGGTGCTTCTGTCGACTTAGCTATTTTCTCCCTTCATCTCGCAGGTGTATCCTCAATTCTGGGGGCCATTAATTTTATTACCACGAT
TATTAACATGAAACCCCCTGCGATTTCTCAGTACCAGACACCTTTGTTCGTATGGGCCGTCTTAATTACAGCGGTTCTCCTACTACT
CTCACTTCCTGTCCTGGCTGCTGGCATTACAATGCTTCTAACA

No.2025-166-25-26

GGAATAGTAGGCACGTCTCTAAGCCTCCTAATCCGAGCAGAACTGAGTCAGCCCGGCTCACTCCTCGGAGATGACCAGATTTTT
AACGTAATTGTTACGGCACATGCCTTCGTTATAATTTTCTTTATAGTAATACCCGTTATGATCGGAGGGTTCGGAAACTGACTCGT
TCCCCTAATGATCGGAGCCCCCGACATGGCCTTCCCCCGAATGAACAATATGAGTTTCTGACTCCTTCCCCCTTCTTTCCTACTAC
TTCTTGTCTCTTCGGCGGTAGAGGCAGGGGCCGGAACAGGGTGAACAGTCTACCCGCCACTCGCCGGAAATCTCGCACACGC
AGGTGCTTCTGTCGACTTAGCTATTTTCTCCCTTCATCTCGCAGGTGTATCCTCAATTCTGGGGGCCATTAATTTTATTACCACGAT
TATTAACATGAAACCCCCTGCGATTTCTCAGTACCAGACACCTTTGTTCGTATGGGCCGTCTTAATTACAGCGGTTCTCCTACTACT
CTCACTTCCTGTCCTGGCTGCTGGCATTACAATGCTTCTAACAG

No.2025-166-25-27

TTCGGTGCATGAGCCGGAATAGTAGGCACGTCTCTAAGCCTCCTAATCCGAGCAGAACTGAGTCAGCCCGGCTCACTCCTCGGA
GATGACCAGATTTTTAACGTAATTGTTACGGCACATGCCTTCGTTATAATTTTCTTTATAGTAATACCCGTTATGATCGGAGGGTTC
GGAAACTGACTCGTTCCCCTAATGATCGGAGCCCCCGACATGGCCTTCCCCCGAATGAACAATATGAGTTTCTGACTCCTTCCCC
CTTCTTTCCTACTACTTCTTGTCTCTTCGGCGGTAGAGGCAGGGGCCGGAACAGGGTGAACAGTCTACCCGCCACTCGCCGGA
AATCTCGCACACGCAGGTGCTTCTGTCGACTTAGCTATTTTCTCCCTTCATCTCGCAGGTGTATCCTCAATTCTGGGGGCCATTAA
TTTTATTACCACGATTATTAACATGAAACCCCCTGCGATTTCTCAGTACCAGACACCTTTGTTCGTATGGGCCGTCTTAATTACAGC
GGTTCTCCTACTACTCTCACTTCCTGTCCTGGCTGCTGGCATTACAATGCTTCTAACAGATCGCAACCTTAACACAACCTTCTTCG
ACCCAGGGGGCGGAGGAGATCCGAT

No.2025-166-25-29

TAGTAGGCACGTCTCTAAGCCTCCTAATCCGAGCAGAACTGAGTCAGCCCGGCTCACTCCTCGGAGATGACCAGATTTTTAACG
TAATTGTTACGGCACATGCCTTCGTTATAATTTTCTTTATAGTAATACCCGTTATGATCGGAGGGTTCGGAAACTGACTCGTTCCCC
TAATGATCGGAGCCCCCGACATGGCCTTCCCCCGAATGAACAATATGAGTTTCTGACTCCTTCCCCCTTCTTTCCTACTACTTCTT
GTCTCTTCGGCGGTAGAGGCAGGGGCCGGAACAGGGTGAACAGTCTACCCGCCACTCGCCGGAAATCTCGCACACGCAGGTG
CTTCTGTCGACTTAGCTATTTTCTCCCTTCATCTCGCAGGTGTATCCTCAATTCTGGGGGCCATTAATTTTATTACCACGATTATTAA
CATGAAACCCCCTGCGATTTCTCAGTACCAGACACCTTTGTTCGTATGGGCCGTCTTAATTACAGCGGTTCTCCTACTACTCTCAC
TTCCTGTCCTGGCTGCTGGCATTACAATGCTTCTAACA

No.2025-166-25-30

TTCGGTGCATGAGCCGGAATAGTAGGCACGTCTCTAAGCCTCCTAATCCGAGCAGAACTGAGTCAGCCCGGCTCACTCCTCGGA
GATGACCAGATTTTTAACGTAATTGTTACGGCACATGCCTTCGTTATAATTTTCTTTATAGTAATACCCGTTATGATCGGAGGGTTC
GGAAACTGACTCGTTCCCCTAATGATCGGAGCCCCCGACATGGCCTTCCCCCGAATGAACAATATGAGTTTCTGACTCCTTCCCC
CTTCTTTCCTACTACTTCTTGTCTCTTCGGCGGTAGAGGCAGGGGCCGGAACAGGGTGAACAGTCTACCCGCCACTCGCCGGA
AATCTCGCACACGCAGGTGCTTCTGTCGACTTAGCTATTTTCTCCCTTCATCTCGCAGGTGTATCCTCAATTCTGGGGGCCATTAA
TTTTATTACCACGATTATTAACATGAAACCCCCTGCGATTTCTCAGTACCAGACACCTTTGTTCGTATGGGCCGTCTTAATTACAGC
GGTTCTCCTACTACTCTCACTTCCTGTCCTGGCTGCTGGCATTACAATGCTTCTAACAGATCGCAACCTTAACACAACCTTCTTCG
ACCCAGGGGGCGGAGGAGATCCGAT

No.2025-166-25-31

TAGTAGGCACAGCCTTAAGCCTCCTAATCCGAGCAGAACTAAGTCAGCCAGGCTCACTCCTCGGAGATGACCAGATTTTTAACG
TAATTGTTACGGCACATGCCTTTGTCATGATTTTCTTTATAGTAATACCCGTCATGATTGGAGGGTTCGGAAACTGACTTGTACCC
CTAATGATCGGAGCCCCCGACATGGCCTTTCCCCGGATGAACAACATAAGCTTCTGGCTTCTCCCCCCTTCTTTCCTGCTACTCCT
CACTTCTTCCGGAGTAGAGGCAGGTGCCGGGACAGGGTGAACAGTCTACCCCCCGCTCGCTGGTAATCTCGCACACGCAGGT
GCTTCTGTCGACCTGGCTATCTTTTCCCTCCATCTCGCAGGTGTATCCTCGATTCTAGGGGCTATTAACTTTATTACAACGATTATTA
ATATGAAGCCCCCAGCAATTTCTCAGTACCAGACGCCTTTATTCGTATGGGCCGTCTTAATTACTGCTGTCCTCCTACTACTCTCAC
TCCCTGTCCTAGCTGCCGGCATTACAATGCTTCTAAC

No.2025-166-25-32

TTCGGTGCATGAGCCGGAATAGTAGGCACGTCTCTAAGCCTCCTAATCCGAGCAGAACTGAGTCAGCCCGGCTCACTCCTCGGA
GATGACCAGATTTTTAACGTAATTGTTACGGCACATGCCTTCGTTATAATTTTCTTTATAGTAATACCCGTTATGATCGGAGGGTTC
GGAAACTGACTCGTTCCCCTAATGATCGGAGCCCCCGACATGGCCTTCCCCCGAATGAACAATATGAGTTTCTGACTCCTTCCCC
CTTCTTTCCTACTACTTCTTGTCTCTTCGGCGGTAGAGGCAGGGGCCGGAACAGGGTGAACAGTCTACCCGCCACTCGCCGGA
AATCTCGCACACGCAGGTGCTTCTGTCGACTTAGCTATTTTCTCCCTTCATCTCGCAGGTGTATCCTCAATTCTGGGGGCCATTAA
TTTTATTACCACGATTATTAACATGAAACCCCCTGCGATTTCTCAGTACCAGACACCTTTGTTCGTATGGGCCGTCTTAATTACAGC
GGTTCTCCTACTACTCTCACTTCCTGTCCTGGCTGCTGGCATTACAATGCTTCTAACAGATCGCAACCTTAACACAACCTTCTTCG

ACCCAGGGGGCGGAGGAGATCCGAT

No.2025-166-25-34

TAGTAGGCACAGCCTTAAGCCTCCTAATCCGAGCAGAACTAAGTCAGCCAGGCTCACTCCTCGGAGATGACCAGATTTTTAACG
TAATTGTTACGGCACATGCCTTTGTCATGATTTTCTTTATAGTAATACCCGTCATGATTGGAGGGTTCGGAAACTGACTTGTACCC
CTAATGATCGGAGCCCCCGACATGGCCTTTCCCCGGATGAACAACATAAGCTTCTGGCTTCTCCCCCCTTCTTTCCTGCTACTCCT
CACTTCTTCCGGAGTAGAGGCAGGTGCCGGGACAGGGTGAACAGTCTACCCCCCGCTCGCTGGTAACCTCGCACACGCAGGT
GCTTCTGTCGACCTGGCTATCTTTTCCCTCCATCTCGCAGGTGTATCCTCGATTCTAGGGGCTATTAACTTTATTACAACGATTATTA
ATATGAAGCCCCCAGCAATTTCTCAGTACCAGACACCTTTATTCGTATGGGCCGTCTTAATTACTGCTGTCCTCCTACTACTCTCAC
TCCCTGTCCTAGCTGCCGGCATTACAATGCTTCTAA

No.2025-166-25-35

AATAGTAGGCACAGCCTTAAGCCTCCTAATCCGAGCAGAACTAAGTCAGCCAGGCTCACTCCTCGGAGATGACCAGATTTTTAA
CGTAATTGTTACGGCACATGCCTTTGTCATGATTTTCTTTATAGTAATACCCGTCATGATTGGAGGGTTCGGAAACTGACTTGTAC
CCCTAATGATCGGAGCCCCCGACATGGCCTTTCCCCGGATGAACAACATAAGCTTCTGGCTTCTCCCCCCTTCTTTCCTGCTACTC
CTCACTTCTTCCGGAGTAGAGGCAGGTGCCGGGACAGGGTGAACAGTCTACCCCCCGCTCGCTGGTAACCTCGCACACGCAG
GTGCTTCTGTCGACCTGGCTATCTTTTCCCTCCATCTCGCAGGTGTATCCTCGATTCTAGGGGCTATTAACTTTATTACAACGATTA
TTAATATGAAGCCCCCAGCAATTTCTCAGTACCAGACACCTTTATTCGTATGGGCCGTCTTAATTACTGCTGTCCTCCTACTACTCT
CACTCCCTGTCCTAGCTGCCGGCATTACAATGCTTCTAA

No.2025-166-25-36

TAGTAGGCACAGCCTTAAGCCTCCTAATCCGAGCAGAACTAAGTCAGCCAGGCTCACTCCTCGGAGATGACCAGATTTTTAACG
TAATTGTTACGGCACATGCCTTTGTCATGATTTTCTTTATAGTAATACCCGTCATGATTGGAGGGTTCGGAAACTGACTTGTACCC
CTAATGATCGGAGCCCCCGACATGGCCTTTCCCCGGATGAACAACATAAGCTTCTGGCTTCTCCCCCCTTCTTTCCTGCTACTCCT
CACTTCTTCCGGAGTAGAGGCAGGTGCCGGGACAGGGTGAACAGTCTACCCCCCGCTCGCTGGTAATCTCGCACACGCAGGT
GCTTCTGTCGACCTGGCTATCTTTTCCCTCCATCTCGCAGGTGTATCCTCGATTCTAGGGGCTATTAACTTTATTACAACGATTATTA
ATATGAAGCCCCCAGCAATTTCTCAGTACCAGACGCCTTTATTCGTATGGGCCGTCTTAATTACTGCTGTCCTCCTACTACTCTCAC
TCCCTGTCCTAGCTGCCGGCATTACAATGCTTCTAACA

No.2025-166-25-37

AGGCACAGCCTTAAGCCTCCTAATCCGAGCAGAACTAAGTCAGCCAGGCTCACTCCTCGGAGATGACCAGATTTTTAACGTAAT
TGTTACGGCACATGCCTTTGTCATGATTTTCTTTATAGTAATACCCGTCATGATTGGAGGGTTCGGAAACTGACTTGTACCCCTAA
TGATCGGAGCCCCCGACATGGCCTTTCCCCGGATGAACAACATAAGCTTCTGGCTTCTCCCCCCTTCTTTCCTGCTACTCCTCACT
TCTTCCGGAGTAGAGGCAGGTGCCGGGACAGGGTGAACAGTCTACCCCCCGCTCGCTGGTAACCTCGCACACGCAGGTGCTT
CTGTCGACCTGGCTATCTTTTCCCTCCATCTCGCAGGTGTATCCTCGATTCTAGGGGCTATTAACTTTATTACAACGATTATTAATAT
GAAGCCCCCAGCAATTTCTCAGTACCAGACACCTTTATTCGTATGGGCCGTCTTAATTACTGCTGTCCTCCTACTACTCTCACTCCC
TGTCCTAGCTGCCGGCATTACAATGCTTCTAACAG

No.2025-166-25-38

GCCAGGCTCACTCCTCGGAGATGACCAGATTTTTAACGTAATTGTTACGGCACATGCCTTTGTCATGATTTTCTTTATAGTAATAC
CCGTCATGATTGGAGGGTTCGGAAACTGACTTGTACCCCTAATGATCGGAGCCCCCGACATGGCCTTTCCCCGGATGAACAACA
TAAGCTTCTGGCTTCTCCCCCCTTCTTTCCTGCTACTCCTCACTTCTTCCGGAGTAGAGGCAGGTGCCGGGACAGGGTGAACAG
TCTACCCCCCGCTCGCTGGTAACCTCGCACACGCAGGTGCTTCTGTCGACCTGGCTATCTTTTCCCTCCATCTCGCAGGTGTATCC
TCGATTCTAGGGGCTATTAACTTTATTACAACGATTATTAATATGAAGCCCCCAGCAATTTCTCAGTACCAGACACCTTTATTCGTA
TGGGCCGTCTTAATTACTGCTGTCCTCCTACTACTCTCACTCCCTGTCCTAGCTGCCGGCATTACAATGCTTCTAAC

No.2025-166-25-39

TAGTAGGCACAGCCTTAAGCCTCCTAATCCGAGCAGAACTAAGTCAGCCAGGCTCACTCCTCGGAGATGACCAGATTTTTAACG
TAATTGTTACGGCACATGCCTTTGTCATGATTTTCTTTATAGTAATACCCGTCATGATTGGAGGGTTCGGAAACTGACTTGTACCC
CTAATGATCGGAGCCCCCGACATGGCCTTTCCCCGGATGAACAACATAAGCTTCTGGCTTCTCCCCCCTTCTTTCCTGCTACTCCT
CACTTCTTCCGGAGTAGAGGCAGGTGCCGGGACAGGGTGAACAGTCTACCCCCCGCTCGCTGGTAACCTCGCACACGCAGGT
GCTTCTGTCGACCTGGCTATCTTTTCCCTCCATCTCGCAGGTGTATCCTCGATTCTAGGGGCTATTAACTTTATTACAACGATTATTA
ATATGAAGCCCCCAGCAATTTCTCAGTACCAGACACCTTTATTCGTATGGGCCGTCTTAATTACTGCTGTCCTCCTACTACTCTCAC
TCCCTGTCCTAGCTGCCGGCATTACAATGCTTCTAACA

No.2025-166-25-40

GCCAGGCTCACTCCTCGGAGATGACCAGATTTTTAACGTAATTGTTACGGCACATGCCTTTGTCATGATTTTCTTTATAGTAATAC
CCGTCATGATTGGAGGGTTCGGAAACTGACTTGTACCCCTAATGATCGGAGCCCCCGACATGGCCTTTCCCCGGATGAACAACA
TAAGCTTCTGGCTTCTCCCCCCTTCTTTCCTGCTACTCCTCACTTCTTCCGGAGTAGAGGCAGGTGCCGGGACAGGGTGAACAG
TCTACCCCCCGCTCGCTGGTAATCTCGCACACGCAGGTGCTTCTGTCGACCTGGCTATCTTTTCCCTCCATCTCGCAGGTGTATCC
TCGATTCTAGGGGCTATTAACTTTATTACAACGATTATTAATATGAAGCCCCCAGCAATTTCTCAGTACCAGACGCCTTTATTCGTA
TGGGCCGTCTTAATTACTGCTGTCCTCCTACTACTCTCACTCCCTGTCCTAGCTGCCGGCATTACAATGCTTCTAACA

No.2025-166-25-41

GCCGGAATAGTAGGCACAGCCTTAAGCCTCCTAATCCGAGCAGAACTAAGTCAGCCAGGCTCACTCCTCGGAGATGACCAGAT
TTTTAACGTAATTGTTACGGCACATGCCTTTGTCATGATTTTCTTTATAGTAATACCCGTCATGATTGGAGGGTTCGGAAACTGAC
TTGTACCCCTAATGATCGGAGCCCCCGACATGGCCTTTCCCCGGATGAACAACATAAGCTTCTGGCTTCTCCCCCCTTCTTTCCTG
CTACTCCTCACTTCTTCCGGAGTAGAGGCAGGTGCCGGGACAGGGTGAACAGTCTACCCCCCGCTCGCTGGTAATCTCGCACAC
GCAGGTGCTTCTGTCGACCTGGCTATCTTTTCCCTCCATCTCGCAGGTGTATCCTCGATTCTAGGGGCTATTAACTTTATTACAAC
GATTATTAATATGAAGCCCCCAGCAATTTCTCAGTACCAGACGCCTTTATTCGTATGGGCCGTCTTAATTACTGCTGTCCTCCTACT
ACTCTCACTCCCTGTCCTAGCTGCCGGCATTACAATGCT

No.2025-166-25-42

CATGAGCCGGAATAGTAGGCACGTCTCTAAGCCTCCTAATCCGAGCAGAACTGAGTCAGCCCGGCTCACTCCTCGGAGATGACC
AGATTTTTAACGTAATTGTTACGGCACATGCCTTCGTTATAATTTTCTTTATAGTAATACCCGTTATGATCGGAGGGTTCGGAAACT
GACTCGTTCCCCTAATGATCGGAGCCCCCGACATGGCCTTCCCCCGAATGAACAATATGAGTTTCTGACTCCTTCCCCCTTCTTTC
CTACTACTTCTTGTCTCTTCGGCGGTAGAGGCAGGGGCCGGAACAGGGTGAACAGTCTACCCGCCACTCGCCGGAAATCTCGC
ACACGCAGGTGCTTCTGTCGACTTAGCTATTTTCTCCCTTCATCTCGCAGGTGTATCCTCAATTCTGGGGGCCATTAATTTTATTAC
CACGATTATTAACATGAAACCCCCCGCGATTTCTCAGTACCAGACACCTTTGTTCGTATGGGCCGTCTTAATTACAGCGGTTCTCC
TACTACTCTCACTTCCTGTCCTGGCTGCTGGCATTACAATGCTTCTAACAGATCGCAACCTTAACACAACCTTCTTCGA

No.2025-166-25-43

CGGTGCATGAGCCGGAATAGTAGGCACGTCTCTAAGCCTCCTAATCCGAGCAGAACTGAGTCAGCCCGGCTCACTCCTCGGAG
ATGACCAGATTTTTAACGTAATTGTTACGGCACATGCCTTCGTTATAATTTTCTTTATAGTAATACCCGTTATGATCGGAGGGTTCG
GAAACTGACTCGTTCCCCTAATGATCGGAGCCCCCGACATGGCCTTCCCCCGAATGAACAATATGAGTTTCTGACTCCTTCCCCC
TTCTTTCCTACTACTTCTTGTCTCTTCGGCGGTAGAGGCAGGGGCCGGAACAGGGTGAACAGTCTACCCGCCACTCGCCGGAA
ATCTCGCACACGCAGGTGCTTCTGTCGACTTAGCTATTTTCTCCCTTCATCTCGCAGGTGTATCCTCAATTCTGGGGGCCATTAAT
TTTATTACCACGATTATTAACATGAAACCCCCTGCGATTTCTCAGTACCAGACACCTTTGTTCGTATGGGCCGTCTTAATTACAGCG
GTTCTCCTACTACTCTCACTTCCTGTCCTGGCTGCTGGCATTACAATGCTTCTAACAGATCGCAACCTTAACACAACCTTCTTCGA
CCCAGGGGGCGGAGGAGATCCGAT

No.2025-166-25-44

TAGTAGGCACAGCCTTAAGCCTCCTAATCCGAGCAGAACTAAGTCAGCCAGGCTCACTCCTCGGAGATGACCAGATTTTTAACG
TAATTGTTACGGCACATGCCTTTGTCATGATTTTCTTTATAGTAATACCCGTCATGATTGGAGGGTTCGGAAACTGACTTGTACCC
CTAATGATCGGAGCCCCCGACATGGCCTTTCCCCGGATGAACAACATAAGCTTCTGGCTTCTCCCCCCTTCTTTCCTGCTACTCCT
CACTTCTTCCGGAGTAGAGGCAGGTGCCGGGACAGGGTGAACAGTCTACCCCCCGCTCGCTGGTAATCTCGCACACGCAGGT
GCTTCTGTCGACCTGGCTATCTTTTCCCTCCATCTCGCAGGTGTATCCTCGATTCTAGGGGCTATTAACTTTATTACAACGATTATTA
ATATGAAGCCCCCAGCAATTTCTCAGTACCAGACGCCTTTATTCGTATGGGCCGTCTTAATTACTGCTGTCCTCCTACTACTCTCAC
TCCCTGTCCTAGCTGCCGGCATTACAATGCTTCTAACA

No.2025-166-25-45

AATAGTAGGCACGTCTCTAAGCCTCCTAATCCGAGCAGAACTGAGTCAGCCCGGCTCACTCCTCGGAGATGACCAGATTTTTAA
CGTAATTGTTACGGCACATGCCTTCGTTATAATTTTCTTTATAGTAATACCCGTTATGATCGGAGGGTTCGGAAACTGACTCGTTCC
CCTAATGATCGGAGCCCCCGACATGGCCTTCCCCCGAATGAACAATATGAGTTTCTGACTCCTTCCCCCTTCTTTCCTACTACTTCT
TGTCTCTTCGGCGGTAGAGGCAGGGGCCGGAACAGGGTGAACAGTCTACCCGCCACTCGCCGGAAATCTCGCACACGCAGGT
GCTTCTGTCGACTTAGCTATTTTCTCCCTTCATCTCGCAGGTGTATCCTCAATTCTGGGGGCCATTAATTTTATTACCACGATTATTA
ACATGAAACCCCCTGCGATTTCTCAGTACCAGACACCTTTGTTCGTATGGGCCGTCTTAATTACAGCGGTTCTCCTACTACTCTCA
CTTCCTGTCCTGGCTGCTGGCATTACAATGCTTCTAACA

No.2025-166-25-46

TGGGCCGGAATAGTAGGCACAGCCTTAAGCCTCCTAATCCGAGCAGAACTAAGTCAGCCAGGCTCACTCCTCGGAGATGACCA
GATTTTTAACGTAATTGTTACGGCACATGCCTTTGTCATGATTTTCTTTATAGTAATACCCGTCATGATTGGAGGGTTCGGAAACT
GACTTGTACCCCTAATGATCGGAGCCCCCGACATGGCCTTTCCCCGGATGAACAACATAAGCTTCTGGCTTCTCCCCCCTTCTTT
CCTGCTACTCCTCACTTCTTCCGGAGTAGAGGCAGGTGCCGGGACAGGGTGAACAGTCTACCCCCCGCTCGCTGGTAACCTCG
CACACGCAGGTGCTTCTGTCGACCTGGCTATCTTTTCCCTCCATCTCGCAGGTGTATCCTCGATTCTAGGGGCTATTAACTTTATTA
CAACGATTATTAATATGAAGCCCCCAGCAATTTCTCAGTACCAGACACCTTTATTCGTATGGGCCGTCTTAATTACTGCTGTCCTCC
TACTACTCTCACTCCCTGTCCTAGCTGCCGGCATTACAATGCTTCTAACAGACCGCA

No.2025-166-25-47

AATAGTAGGCACAGCCTTAAGCCTCCTAATCCGAGCAGAACTAAGTCAGCCAGGCTCACTCCTCGGAGATGACCAGATTTTTAA
CGTAATTGTTACGGCACATGCCTTTGTCATGATTTTCTTTATAGTAATACCCGTCATGATTGGAGGGTTCGGAAACTGACTTGTAC
CCCTAATGATCGGAGCCCCCGACATGGCCTTTCCCCGGATGAACAACATAAGCTTCTGGCTTCTCCCCCCTTCTTTCCTGCTACTC
CTCACTTCTTCCGGAGTAGAGGCAGGTGCCGGGACAGGGTGAACAGTCTACCCCCCGCTCGCTGGTAACCTCGCACACGCAG
GTGCTTCTGTCGACCTGGCTATCTTTTCCCTCCATCTCGCAGGTGTATCCTCGATTCTAGGGGCTATTAACTTTATTACAACGATTA
TTAATATGAAGCCCCCAGCAATTTCTCAGTACCAGACACCTTTATTCGTATGGGCCGTCTTAATTACTGCTGTCCTCCTACTACTCT
CACTCCCTGTCCTAGCTGCCGGCATTACAATGCTTCTAAC

No.2025-166-25-48

AGTAGGCACAGCCTTAAGCCTCCTAATCCGAGCAGAACTAAGTCAGCCAGGCTCACTCCTCGGAGATGACCAGATTTTTAACGT
AATTGTTACGGCACATGCCTTTGTCATGATTTTCTTTATAGTAATACCCGTCATGATTGGAGGGTTCGGAAACTGACTTGTACCCC
TAATGATCGGAGCCCCCGACATGGCCTTTCCCCGGATGAACAACATAAGCTTCTGGCTTCTCCCCCCTTCTTTCCTGCTACTCCTC
ACTTCTTCCGGAGTAGAGGCAGGTGCCGGGACAGGGTGAACAGTCTACCCCCCGCTCGCTGGTAATCTCGCACACGCAGGTG
CTTCTGTCGACCTGGCTATCTTTTCCCTCCATCTCGCAGGTGTGTCCTCGATTCTAGGGGCTATTAACTTTATTACAACGATTATTA
ATATGAAGCCCCCAGCAATTTCTCAGTACCAGACACCTTTATTCGTATGGGCCGTCTTAATTACTGCTGTCCTCCTACTACTCTCAC
TCCCTGTCCTAGCTGCCGGCATTACAATGCTTCTAA

No.2025-166-25-49

TGGGCCGGAATAGTAGGCACAGCCTTAAGCCTCCTAATCCGAGCAGAACTAAGTCAGCCAGGCTCACTCCTCGGAGATGACCA
GATTTTTAACGTAATTGTTACGGCACATGCCTTTGTCATGATTTTCTTTATAGTAATACCCGTCATGATTGGAGGGTTCGGAAACT
GACTTGTACCCCTAATGATCGGAGCCCCCGACATGGCCTTTCCCCGGATGAACAACATAAGCTTCTGGCTTCTCCCCCCTTCTTT
CCTGCTACTCCTCACTTCTTCCGGAGTAGAGGCAGGTGCCGGGACAGGGTGAACAGTCTACCCCCCGCTCGCTGGTAACCTCG
CACACGCAGGTGCTTCTGTCGACCTGGCTATCTTTTCCCTCCATCTCGCAGGTGTATCCTCGATTCTAGGGGCTATTAACTTTATTA
CAACGATTATTAATATGAAGCCCCCAGCAATTTCTCAGTACCAGACACCTTTATTCGTATGGGCCGTCTTAATTACTGCTGTCCTCC

TACTACTCTCACTCCCTGTCCTAGCTGCCGGCATTACAATGCTTCTAACAGACCGCAACCTCAACACAACCTTCT

No.2025-166-25-50

AATAGTAGGCACAGCCTTAAGCCTCCTAATCCGAGCAGAACTAAGTCAGCCAGGCTCACTCCTCGGAGATGACCAGATTTTTAA
CGTAATTGTTACGGCACATGCCTTTGTCATGATTTTCTTTATAGTAATACCCGTCATGATTGGAGGGTTCGGAAACTGACTTGTAC
CCCTAATGATCGGAGCCCCCGACATGGCCTTTCCCCGGATGAACAACATAAGCTTCTGGCTTCTCCCCCCTTCTTTCCTGCTACTC
CTCACTTCTTCCGGAGTAGAGGCAGGTGCCGGGACAGGGTGAACAGTCTACCCCCCGCTCGCTGGTAATCTCGCACACGCAGG
TGCTTCTGTCGACCTGGCTATCTTTTCCCTCCATCTCGCAGGTGTGTCCTCGATTCTAGGGGCTATTAACTTTATTACAACGATTAT
TAATATGAAGCCCCCAGCAATTTCTCAGTACCAGACACCTTTATTCGTATGGGCCGTCTTAATTACTGCTGTCCTCCTACTACTCTC
ACTCCCTGTCCTAGCTGCCGGCATTACAATGCTTCTAACA

No.2025-166-25-51

GAATAGTAGGCACAGCCTTAAGCCTCCTAATCCGAGCAGAACTAAGTCAGCCAGGCTCACTCCTCGGAGATGACCAGATTTTTA
ACGTAATTGTTACGGCACATGCCTTTGTCATGATTTTCTTTATAGTAATACCCGTCATGATTGGAGGGTTCGGAAACTGACTTGTA
CCCCTAATGATCGGAGCCCCCGACATGGCCTTTCCCCGGATGAACAACATAAGCTTCTGGCTTCTCCCCCCTTCTTTCCTGCTGCT
CCTCACTTCTTCCGGAGTAGAGGCAGGTGCCGGGACAGGGTGAACAGTCTACCCCCCGCTCGCTGGTAACCTCGCACACGCAG
GTGCTTCTGTCGACCTGGCTATCTTTTCCCTCCATCTCGCAGGTGTATCCTCGATTCTAGGGGCTATTAACTTTATTACAACGATTA
TTAATATGAAGCCCCCAGCAATTTCTCAGTACCAGACACCTTTATTCGTATGGGCCGTCTTAATTACTGCTGTCCTCCTACTACTCT
CACTCCCTGTCCTAGCTGCCGGCATTACAATGCTTCTAAC

No.2025-166-25-52

TGAGCCGGAATAGTGGGGACGGCCCTAAGCCTTTTAATTCGTGCGGAACTTAGCCAACCCGGCGCCCTCCTAGGAGACGACCA
AATATATAATGTTATCGTTACTGCACACGCCTTCGTAATAATCTTTTTTATAGTTATGCCAATTATAATTGGAGGTTTCGGGAACTGA
CTTGTACCTTTAATAATTGGCGCCCCTGACATGGCATTCCCTCGAATAAACAACATGAGCTTCTGACTACTCCCTCCTTCCTTTCTG
CTCCTTCTCGCCTCCTCTGGGGTTGAAGCTGGAGCCGGAACAGGTTGGACCGTATATCCTCCTCTAGCAAGCAACGTCGCCCAC
TCCAGGAGCATCAGTTGACTTAACTATTTTTTCCCTCCACTTAGCGGGTGTTTCATCCATTCTTGGGGCAATTAATTTTATCACAA
CCATCTTTAATATAAAACCTGCCGCTGCTTCAATATATCAAATACCCCTGTTCGTTTGGGCCGTTCTAGTTACAGCAGTACTACTTC
TTCTTTCTCTCCCTGTCTTAGCCGCTGGCATCACTATACTAT

No.2025-166-25-53

AATAGTAGGCACAGCCTTAAGCCTCCTAATCCGAGCAGAACTAAGTCAGCCAGGCTCACTCCTCGGAGATGACCAGATTTTTAA
CGTAATTGTTACGGCACATGCCTTTGTCATGATTTTCTTTATAGTAATACCCGTCATGATTGGAGGGTTCGGAAACTGACTTGTAC
CCCTAATGATCGGAGCCCCCGACATGGCCTTTCCCCGGATGAACAACATAAGCTTCTGGCTTCTCCCCCCTTCTTTCCTGCTACTC
CTCACTTCTTCCGGAGTAGAGGCAGGTGCCGGGACAGGGTGAACAGTCTACCCCCCGCTCGCTGGTAATCTCGCACACGCAGG
TGCTTCTGTCGACCTGGCTATCTTTTCCCTCCATCTCGCAGGTGTATCCTCGATTCTAGGGGCTATTAACTTTATTACAACGATTATT
AATATGAAGCCCCCAGCAATTTCTCAGTACCAGACACCTTTATTCGTATGGGCCGTCTTAATTACTGCTGTCCTCCTACTACTCTCA
CTCCCTGTCCTAGCTGCCGGCATTACAATGCTTCT

No.2025-166-25-54

TGAGCCGGAATAGTGGGGACGGCCCTAAGCCTTTTAATTCGTGCGGAACTTAGCCAACCCGGCGCCCTCCTAGGAGACGACCA
AATATATAATGTTATCGTTACTGCACACGCCTTCGTAATAATCTTTTTTATAGTTATGCCAATTATAATTGGAGGTTTCGGGAACTGA
CTTGTACCTTTAATAATTGGCGCCCCTGACATGGCATTCCCTCGAATAAACAACATGAGCTTCTGACTACTCCCTCCTTCCTTTCTG
CTCCTTCTCGCCTCCTCTGGGGTTGAAGCTGGAGCCGGAACAGGTTGGACCGTATATCCTCCTCTAGCAAGCAACGTCGCCCAC
TCCAGGAGCATCAGTTGACTTAACTATTTTTTCCCTCCACTTAGCGGGTGTTTCATCCATTCTTGGGGCAATTAATTTTATCACAA
CCATCTTTAATATAAAACCTGCCGCTGCTTCAATATATCAAATACCCCTGTTCGTTTGGGCCGTTCTAGTTACAGCAGTACTACTTC
TTCTTTACTCTCCACTGTCTTAGCACGCTGGCGATCACTATACTAT

No.2025-166-25-55

ATAGTGGGGACGGCCCTAAGCCTTTTAATTCGTGCGGAACTTAGCCAACCCGGCGCCCTCCTAGGAGACGACCAAATATATAAT
GTTATCGTTACTGCACACGCCTTCGTAATAATCTTTTTTATAGTTATGCCAATTATAATTGGAGGTTTCGGGAACTGACTTGTACCT
TTAATAATTGGCGCCCCTGACATGGCATTCCCTCGAATAAACAACATGAGCTTCTGACTACTCCCTCCTTCCTTTCTGCTCCTTCTC
GCCTCCTCTGGGGTTGAAGCTGGAGCCGGAACAGGTTGGACCGTATATCCTCCTCTAGCAAGCAACGTCGCCCACTCCAGGAG
CATCAGTTGACTTAACTATTTTTTCCCTCCACTTAGCGGGTGTTTCATCCATTCTTGGGGCAATTAATTTTATCACAACCATCTTTAA
TATAAAACCTGCCGCTGCTTCAATATATCAAATACCCCTGTTCGTTTGGGCCGTTCTAGTTACAGCAGTACTACTTCTTCTTTCTCT
CCCTGTCTTAGCCGCTGGCATCACTATACTAT

No.2025-166-25-56

AATAGTAGGCACGTCTCTAAGCCTCCTAATCCGAGCAGAACTGAGTCAGCCCGGCTCACTCCTCGGAGATGACCAGATTTTTAA
CGTAATTGTTACGGCACATGCCTTCGTTATAATTTTCTTTATAGTAATACCCGTTATGATCGGAGGGTTCGGAAACTGACTCGTTCC
CCTAATGATCGGAGCCCCCGACATGGCCTTCCCCCGAATGAACAATATGAGTTTCTGACTCCTTCCCCCTTCTTTCCTACTACTTCT
TGTCTCTTCGGCGGTAGAGGCAGGGGCCGGAACAGGGTGAACAGTCTACCCGCCACTCGCCGGAAATCTCGCACACGCAGGT
GCTTCTGTCGACTTAGCTATTTTCTCCCTTCATCTCGCAGGTGTATCCTCAATTCTGGGGGCCATTAATTTTATTACCACGATTATTA
ACATGAAACCCCCTGCGATTTCTCAGTACCAGACACCTTTGTTCGTATGGGCCGTCTTAATTACAGCGGTTCTCCTACTACTCTCA
CTTTCCTGTCCTGGCTGCTGGCATTACAATGCTTCTAAC

No.2025-166-25-57

AATAGTAGGCACGTCTCTAAGCCTCCTAATCCGAGCAGAACTGAGTCAGCCCGGCTCACTCCTCGGAGATGACCAGATTTTTAA
CGTAATTGTTACGGCACATGCCTTCGTTATAATTTTCTTTATAGTAATACCCGTTATGATCGGAGGGTTCGGAAACTGACTCGTTCC
CCTAATGATCGGAGCCCCCGACATGGCCTTCCCCCGAATGAACAATATGAGTTTCTGACTCCTTCCCCCTTCTTTCCTACTACTTCT
TGTCTCTTCGGCGGTAGAGGCAGGGGCCGGAACAGGGTGAACAGTCTACCCGCCACTCGCCGGAAATCTCGCACACGCAGGT
GCTTCTGTCGACTTAGCTATTTTCTCCCTTCATCTCGCAGGTGTATCCTCAATTCTGGGGGCCATTAATTTTATTACCACGATTATTA

ACATGAAACCCCCTGCGATTTCTCAGTACCAGACACCTTTGTTCGTATGGGCCGTCTTAATTACAGCGGTTCTCCTACTACTCTCA
CTTCCTGTCCTGGCTGCTGGCATTACAATGCTTCTAAC

No.2025-166-25-58

ATAGTGGGGACGGCCCTAAGCCTTTTAATTCGTGCGGAACTTAGCCAACCCGGCGCCCTCCTAGGAGACGACCAAATATATAAT
GTTATCGTTACTGCACACGCCTTCGTAATAATCTTTTTTATAGTTATGCCAATTATAATTGGAGGTTTCGGGAACTGACTTGTACCT
TTAATAATTGGCGCCCCTGACATGGCATTCCCTCGAATAAACAACATGAGCTTCTGACTACTCCCTCCTTCCTTTCTGCTCCTTCTC
GCCTCCTCTGGGGTTGAAGCTGGAGCCGGAACAGGTTGGACCGTATATCCTCCTCTAGCAAGCAACGTCGCCCACTCCAGGAG
CATCAGTTGACTTAACTATTTTTTCCCTCCACTTAGCGGGTGTTTCATCCATTCTTGGGGCAATTAATTTTATCACAACCATCTTTAA
TATAAAACCTGCCGCTGCTTCAATATATCAAATACCCCTGTTCGTTTGGGCCGTTCTAGTTACAGCAGTACTACTTCTTCTTTCTCT
CCCTGTCTTAGCCGCTGGCATCACTATACTAT

No.2025-166-25-59

ATAGTGGGGACGGCCCTAAGCCTTTTAATTCGTGCGGAACTTAGCCAACCCGGCGCCCTCCTAGGAGACGACCAAATATATAAT
GTTATCGTTACTGCACACGCCTTCGTAATAATCTTTTTTATAGTTATGCCAATTATAATTGGAGGTTTCGGGAACTGACTTGTACCT
TTAATAATTGGCGCCCCTGACATGGCATTCCCTCGAATAAACAACATGAGCTTCTGACTACTCCCTCCTTCCTTTCTGCTCCTTCTC
GCCTCCTCTGGGGTTGAAGCTGGAGCCGGAACAGGTTGGACCGTATATCCTCCTCTAGCAAGCAACGTCGCCCACTCCAGGAG
CATCAGTTGACTTAACTATTTTTTCCCTCCACTTAGCGGGTGTTTCATCCATTCTTGGGGCAATTAATTTTATCACAACCATCTTTAA
TATAAAACCTGCCGCTGCTTCAATATATCAAATACCCCTGTTCGTTTGGGCCGTTCTAGTTACAGCAGTATTACTTCTTCTTTCTCTC
CCTGTCTTAGCCGCTGGCATCACTATACTAT

Fish maw: 16S rRNA gene – Supplementary samples (n=30)

No.2025-166-24-1

AGAGGTCCTGCCTGCCCTGTGACCATGAGTTCAACGGCCGCGGTATTTTGACCGTGCAAAGGTAGCGCAATCACTTGTCTTTTA
AATGAAGACCCGTATGAATGGCAAGACGAGGGCTTAGCTGTCTCCTTTTTCAAGTCAATGAAATTGATCTTCCCGTGCAGAAGC
GGGAATCCCCCCATAAGACGAGAAGACCCTATGGAGCTTTAGACACGAAGACAGGCCACGTCAAACCCCCTAACTAAAGAATT
GAACTAAATGGACCCTGCCCTAATGTCTTTGGTTGGGGCGACCACGGGGAAAGACAAAACCCCCGCGTGGAATGGAAGCACC
CCTTGCTTCTGCAACCAAGAGCTCCCGCTCTAATAAACAGAACTTCTGACCAACAAGATCCGGCAAAGCCGATCAACGAACCGA
GTTACCCTAGGGATAACAGCGCAATCCTCTTTTAGAGCCCATATCGACAAGAGGGTTTACGACCTCGATGTTGGATCAGGACATC
CTAATGGTGCAACCGCTATTAAGGGTTCGTT

No.2025-166-24-2

AGAGGTCCTGCCTGCCCTGTGACCATGAGTTCAACGGCCGCGGTATTTTGACCGTGCAAAGGTAGCGCAATCACTTGTCTTTTA
AATGAAGACCCGTATGAATGGCAAGACGAGGGCTTAGCTGTCTCCTTTTTCAAGTCAATGAAATTGATCTTCCCGTGCAGAAGC
GGGAATCCCCCCATAAGACGAGAAGACCCTATGGAGCTTTAGACACGAAGACAGGCCACGTCAAACCCCCTAACTAAAGAATT
GAACTAAATGGACCCTGCCCTAATGTCTTTGGTTGGGGCGACCACGGGGAAAGACAAAACCCCCGCGTGGAATGGAAGCACC
CCTTGCTTCTGCAACCAAGAGCTCCCGCTCTAATAAACAGAACTTCTGACCAACAAGATCCGGCAAAGCCGATCAACGAACCGA
GTTACCCTAGGGATAACAGCGCAATCCTCTTTTAGAGCCCATATCGACAAGAGGGTTTACGACCTCGATGTTGGATCAGGACATC
CTAATGGTGCAACCGCTATTAAGGGTTCGTT

No.2025-166-24-4

AGAGGTCCTGCCTGCCCTGTGACCATGAGTTCAACGGCCGCGGTATTTTGACCGTGCAAAGGTAGCGCAATCACTTGTCTTTTA
AATGAAGACCCGTATGAATGGCAAGACGAGGGCTTAGCTGTCTCCTTTTTCAAGTCAATGAAATTGATCTTCCCGTGCAGAAGC
GGGAATCCCCCCATAAGACGAGAAGACCCTATGGAGCTTTAGACACGAAGACAGGCCACGTCAAACCCCCTAACTAAAGAATT
GAACTAAATGGACCCTGCCCTAATGTCTTTGGTTGGGGCGACCACGGGGAAAGACAAAACCCCCGCGTGGAATGGAAGCACC
CCTTGCTTCTGCAACCAAGAGCTCCCGCTCTAATAAACAGAACTTCTGACCAACAAGATCCGGCAAAGCCGATCAACGAACCGA
GTTACCCTAGGGATAACAGCGCAATCCTCTTTTAGAGCCCATATCGACAAGAGGGTTTACGACCTCGATGTTGGATCAGGACATC
CTAATGGTGCAACCGCTATTAAGGGTTCGTT

No.2025-166-24-8

AGAGGTCCTGCCTGCCCTGTGACCATGAGTTCAACGGCCGCGGTATTTTGACCGTGCAAAGGTAGCGCAATCACTTGTCTTTTA
AATGAAGACCCGTATGAATGGCAAGACGAGGGCTTAGCTGTCTCCTTTTTCAAGTCAATGAAATTGATCTTCCCGTGCAGAAGC
GGGAATCCCCCCATAAGACGAGAAGACCCTATGGAGCTTTAGACACGAAGACAGGCCACGTCAAACCCCCTAACTAAAGAATT
GAACTAAATGGACCCTGCCCTAATGTCTTTGGTTGGGGCGACCACGGGGAAAGACAAAACCCCCGCGTGGAATGGAAGCACC
CCTTGCTTCTGCAACCAAGAGCTCCCGCTCTAATAAACAGAACTTCTGACCAACAAGATCCGGCAAAGCCGATCAACGAACCGA
GTTACCCTAGGGATAACAGCGCAATCCTCTTTTAGAGCCCATATCGACAAGAGGGTTTACGACCTCGATGTTGGATCAGGACATC
CTAATGGTGC

No.2025-166-24-10

AGAGGTCCTGCCTGCCCTGTGACCATGAGTTCAACGGCCGCGGTATTTTGACCGTGCAAAGGTAGCGCAATCACTTGTCTTTTA
AATGAAGACCCGTATGAATGGCAAGACGAGGGCTTAGCTGTCTCCTTTTTCAAGTCAATGAAATTGATCTTCCCGTGCAGAAGC
GGGAATCCCCCCATAAGACGAGAAGACCCTATGGAGCTTTAGACACGAAGACAGGCCACGTCAAACCCCCTAACTAAAGAATT
GAACTAAATGGACCCTGCCCTAATGTCTTTGGTTGGGGCGACCACGGGGAAAGACAAAACCCCCGCGTGGAATGGAAGCACC
CCTTGCTTCTGCAACCAAGAGCTCCCGCTCTAATAAACAGAACTTCTGACCAACAAGATCCGGCAAAGCCGATCAACGAACCGA
GTTACCCTAGGGATAACAGCGCAATCCTCTTTTAGAGCCCATATCGACAAGAGGGTTTACGACCTCGATGTTGGATCAGGACATC
CTAATGGTGC

No.2025-166-24-11

AGAGGTCCTGCCTGCCCTGTGACCATGAGTTCAACGGCCGCGGTATTTTGACCGTGCAAAGGTAGCGCAATCACTTGTCTTTTA
AATGAAGACCCGTATGAATGGCAAGACGAGGGCTTAGCTGTCTCCTTTTTCAAGTCAATGAAATTGATCTTCCCGTGCAGAAGC
GGGAATCCCCCCATAAGACGAGAAGACCCTATGGAGCTTTAGACACGAAGACAGGCCACGTCAAACCCCCTAACTAAAGAATT
GAACTAAATGGACCCTGCCCTAATGTCTTTGGTTGGGGCGACCACGGGGAAAGACAAAACCCCCGCGTGGAATGGAAGCACC
CCTTGCTTCTGCAACCAAGAGCTCCCGCTCTAATAAACAGAACTTCTGACCAACAAGATCCGGCAAAGCCGATCAACGAACCGA
GTTACCCTAGGGATAACAGCGCAATCCTCTTTTAGAGCCCATATCGACAAGAGGGTTTACGACCTCGATGTTGGATCAGGACATC
CTAATGGT

No.2025-166-25-4

AGAGGTCCTGCCTGCCCTGTGACCATGAGTTCAACGGCCGCGGTATTTTGACCGTGCAAAGGTAGCGCAATCACTTGTCTTTTA
AATGAAGACCCGTATGAATGGCAAGACGAGGGCTTAGCTGTCTCCTTTTTCAAGTCAATGAAATTGATCTTCCCGTGCAGAAGC
GGGAATCCCCCCATAAGACGAGAAGACCCTATGGAGCTTTAGACACGAAGACAGGCCACGTCAAACCCCCTAACTAAAGAATT
GAACTAAATGGACCCTGCCCTAATGTCTTTGGTTGGGGCGACCACGGGGAAAGACAAAACCCCCGCGTGGAATGGAAGCACC
CCTTGCTTCTGCAACCAAGAGCTCCCGCTCTAATAAACAGAACTTCTGACCAACAAGATCCGGCAAAGCCGATCAACGAACCGA
GTTACCCTAGGGATAACAGCGCAATCCTCTTTTAGAGCCCATATCGACAAGAGGGTTTACGACCTCGATGTTGGATCAGGACATC
CTAATGGTGCAACCGCTATTAAGGGTTCGTT

No.2025-166-25-6

GCCTGCCCTGTGACCATGAGTTCAACGGCCGCGGTATTTTGACCGTGCAAAGGTAGCGCAATCACTTGTCTTTTAAATGAAGAC
CCGTATGAATGGCAAGACGAGGGCTTAGCTGTCTCCTTTTTCAAGTCAATGAAATTGATCTTCCCGTGCAGAAGCGGGAATCCC
CCCATAAGACGAGAAGACCCTATGGAGCTTTAGACACGAAGACAGGCCACGTCAAACCCCCTAACTAAAGAATTGAACTAAAT

GGACCCTGCCCTAATGTCTTTGGTTGGGGCGACCACGGGGAAAGACAAAACCCCCGCGTGGAATGGAAGCACCCCTTGCTTC
TGCAACCAAGAGCTCCCGCTCTAATAAACAGAACTTCTGACCAACAAGATCCGGCAAAGCCGATCAACGAACCGAGTTACCCTA
GGGATAACAGCGCAATCCTCTTTTAGAGCCCATATCGACAAGAGGGTTTACGACCTCGATGTTGGATCAGGACATCCTAATGGT
GCAACCGCTATTAAGGGTTCGTTTGTTCAACGATTAAAGTCCTACGTGA

No.2025-166-25-8

AGAGGTCCTGCCTGCCCTGTGACCATGAGTTCAACGGCCGCGGTATTTTGACCGTGCAAAGGTAGCGCAATCACTTGTCTTTTA
AATGAAGACCCGTATGAATGGCAAGACGAGGGCTTAGCTGTCTCCTTTTTCAAGTCAATGAAATTGATCTTCCCGTGCAGAAGC
GGGAATCCCCCCATAAGACGAGAAGACCCTATGGAGCTTTAGACACGAAGACAGGCCACGTCAAACCCCCTAACTAAAGAATT
GAACTAAATGGACCCTGCCCTAATGTCTTTGGTTGGGGCGACCACGGGGAAAGACAAAACCCCCGCGTGGAATGGAAGCACC
CCTTGCTTCTGCAACCAAGAGCTCCCGCTCTAATAAACAGAACTTCTGACCAACAAGATCCGGCAAAGCCGATCAACGAACCGA
GTTACCCTAGGGATAACAGCGCAATCCTCTTTTAGAGCCCATATCGACAAGAGGGTTTACGACCTCGATGTTGGATCAGGACATC
CTAATGGTGCAACCGCTATTAAGGGTTCGTT

No.2025-166-25-9

AGAGGTCCTGCCTGCCCTGTGACCATGAGTTCAACGGCCGCGGTATTTTGACCGTGCAAAGGTAGCGCAATCACTTGTCTTTTA
AATGAAGACCCGTATGAATGGCAAGACGAGGGCTTAGCTGTCTCCTTTTTCAAGTCAATGAAATTGATCTTCCCGTGCAGAAGC
GGGAATCCCCCCATAAGACGAGAAGACCCTATGGAGCTTTAGACACGAAGACAGGCCACGTCAAACCCCCTAACTAAAGAATT
GAACTAAATGGACCCTGCCCTAATGTCTTTGGTTGGGGCGACCACGGGGAAAGACAAAACCCCCGCGTGGAATGGAAGCACC
CCTTGCTTCTGCAACCAAGAGCTCCCGCTCTAATAAACAGAACTTCTGACCAACAAGATCCGGCAAAGCCGATCAACGAACCGA
GTTACCCTAGGGATAACAGCGCAATCCTCTTTTAGAGCCCATATCGACAAGAGGGTTTACGACCTCGATGTTGGATCAGGACATC
CTAATGGTGCAACCGCTATTAAGGGTTCGTTT

No.2025-166-25-10

AGAGGTCCTGCCTGCCCTGTGACCATGAGTTCAACGGCCGCGGTATTTTGACCGTGCAAAGGTAGCGCAATCACTTGTCTTTTA
AATGAAGACCCGTATGAATGGCAAGACGAGGGCTTAGCTGTCTCCTTTTTCAAGTCAATGAAATTGATCTTCCCGTGCAGAAGC
GGGAATCCCCCCATAAGACGAGAAGACCCTATGGAGCTTTAGACACGAAGACAGGCCACGTCAAACCCCCTAACTAAAGAATT
GAACTAAATGGACCCTGCCCTAATGTCTTTGGTTGGGGCGACCACGGGGAAAGACAAAACCCCCGCGTGGAATGGAAGCACC
CCTTGCTTCTGCAACCAAGAGCTCCCGCTCTAATAAACAGAACTTCTGACCAACAAGATCCGGCAAAGCCGATCAACGAACCGA
GTTACCCTAGGGATAACAGCGCAATCCTCTTTTAGAGCCCATATCGACAAGAGGGTTTACGACCTCGATGTTGGATCAGGACATC
CTAATGGTGCAACCGCTATTAAGGGTTCGTT

No.2025-166-25-11

GAGGTCCTGCCTGCCCTGTGACCATGAGTTCAACGGCCGCGGTATTTTGACCGTGCAAAGGTAGCGCAATCACTTGTCTTTTAA
ATGAAGACCCGTATGAATGGCAAGACGAGGGCTTAGCTGTCTCCTTTTTCAAGTCAATGAAATTGATCTTCCCGTGCAGAAGCG
GGAATCCCCCCATAAGACGAGAAGACCCTATGGAGCTTTAGACACGAAGACAGGCCACGTCAAACCCCCTAACTAAAGAATTG
AACTAAATGGACCCTGCCCTAATGTCTTTGGTTGGGGCGACCACGGGGAAAGACAAAACCCCCGCGTGGAATGGAAGCACCC
CTTGCTTCTGCAACCAAGAGCTCCCGCTCTAATAAACAGAACTTCTGACCAACAAGATCCGGCAAAGCCGATCAACGAACCGA
GTTACCCTAGGGATAACAGCGCAATCCTCTTTTAGAGCCCATATCGACAAGAGGGTTTACGACCTCGATGTTGGATCAGGACATC
CTAATGGTGCAACCGCTATTAAGGGTTCGTT

No.2025-166-25-12

AGAGGTCCTGCCTGCCCTGTGACCATGAGTTCAACGGCCGCGGTATTTTGACCGTGCAAAGGTAGCGCAATCACTTGTCTTTTA
AATGAAGACCCGTATGAATGGCAAGACGAGGGCTTAGCTGTCTCCTTTTTCAAGTCAATGAAATTGATCTTCCCGTGCAGAAGC
GGGAATCCCCCCATAAGACGAGAAGACCCTATGGAGCTTTAGACACGAAGACAGGCCACGTCAAACCCCCTAACTAAAGAATT
GAACTAAATGGACCCTGCCCTAATGTCTTTGGTTGGGGCGACCACGGGGAAAGACAAAACCCCCGCGTGGAATGGAAGCACC
CCTTGCTTCTGCAACCAAGAGCTCCCGCTCTAATAAACAGAACTTCTGACCAACAAGATCCGGCAAAGCCGATCAACGAACCGA
GTTACCCTAGGGATAACAGCGCAATCCTCTTTTAGAGCCCATATCGACAAGAGGGTTTACGACCTCGATGTTGGATCAGGACATC
CTAATGGTGCAACCGCTATTAAGGGTTCGTT

No.2025-166-25-14

ATAAGAGGTCCTGCCTGCCCTGTGACCATGAGTTCAACGGCCGCGGTATTTTGACCGTGCAAAGGTAGCGCAATCACTTGTCTT
TTAAATGAAGACCCGTATGAATGGCAAGACGAGGGCTTAGCTGTCTCCTTTTTCAAGTCAATGAAATTGATCTTCCCGTGCAGA
AGCGGGAATCCCCCCATAAGACGAGAAGACCCTATGGAGCTTTAGACACGAAGACAGGCCACGTCAAACCCCCTAACTAAAGA
ATTGAACTAAATGGACCCTGCCCTAATGTCTTTGGTTGGGGCGACCACGGGGAAAGACAAAACCCCCGCGTGGAATGGAAGC
ACCCCTTGCTTCTGCAACCAAGAGCTCCCGCTCTAATAAACAGAACTTCTGACCAACAAGATCCGGCAAAGCCGATCAACGAAC
CGAGTTACCCTAGGGATAACAGCGCAATCCTCTTTTAGAGCCCATATCGACAAGAGGGTTTACGACCTCGATGTTGGATCAGGA
CATCCTAATGGTGCAACCGCTATTAAGGGTTCGTT

No.2025-166-25-15

ATAAGAGGTCCTGCCTGCCCTGTGACCATGAGTTCAACGGCCGCGGTATTTTGACCGTGCAAAGGTAGCGCAATCACTTGTCTT
TTAAATGAAGACCCGTATGAATGGCAAGACGAGGGCTTAGCTGTCTCCTTTTTCAAGTCAATGAAATTGATCTTCCCGTGCAGA
AGCGGGAATCCCCCCATAAGACGAGAAGACCCTATGGAGCTTTAGACACGAAGACAGGCCACGTCAAACCCCCTAACTAAAGA
ATTGAACTAAATGGACCCTGCCCTAATGTCTTTGGTTGGGGCGACCACGGGGAAAGACAAAACCCCCGCGTGGAATGGAAGC
ACCCCTTGCTTCTGCAACCAAGAGCTCCCGCTCTAATAAACAGAACTTCTGACCAACAAGATCCGGCAAAGCCGATCAACGAAC
CGAGTTACCCTAGGGATAACAGCGCAATCCTCTTTTAGAGCCCATATCGACAAGAGGGTTTACGACCTCGATGTTGGATCAGGA
CATCCTAATGGTGCAACCGCTATTAAGGGTTCGTT

No.2025-166-25-17

AGAGGTCCTGCCTGCCCTGTGACCATGAGTTCAACGGCCGCGGTATTTTGACCGTGCAAAGGTAGCGCAATCACTTGTCTTTTA
AATGAAGACCCGTATGAATGGCAAGACGAGGGCTTAGCTGTCTCCTTTTTCAAGTCAATGAAATTGATCTTCCCGTGCAGAAGC

GGGAATCCCCCCATAAGACGAGAAGACCCTATGGAGCTTTAGACACGAAGACAGGCCACGTCAAACCCCCTAACTAAAGAATT
GAACTAAATGGACCCTGCCCTAATGTCTTTGGTTGGGGCGACCACGGGGAAAGACAAAACCCCCGCGTGGAATGGAAGCACC
CCTTGCTTCTGCAACCAAGAGCTCCCGCTCTAATAAACAGAACTTCTGACCAACAAGATCCGGCAAAGCCGATCAACGAACCGA
GTTACCCTAGGGATAACAGCGCAATCCTCTTTTAGAGCCCATATCGACAAGAGGGTTTACGACCTCGATGTTGGATCAGGACATC
CTAATGGTGCAACCGCTATTAAGGGTTCGTT

No.2025-166-25-19

AGAGGTCCTGCCTGCCCTGTGACCATGAGTTCAACGGCCGCGGTATTTTGACCGTGCAAAGGTAGCGCAATCACTTGTCTTTTA
AATGAAGACCCGTATGAATGGCAAGACGAGGGCTTAGCTGTCTCCTTTTTCAAGTCAATGAAATTGATCTTCCCGTGCAGAAGC
GGGAATCCCCCCATAAGACGAGAAGACCCTATGGAGCTTTAGACACGAAGACAGGCCACGTCAAACCCCCTAACTAAAGAATT
GAACTAAATGGACCCTGCCCTAATGTCTTTGGTTGGGGCGACCACGGGGAAAGACAAAACCCCCGCGTGGAATGGAAGCACC
CCTTGCTTCTGCAACCAAGAGCTCCCGCTCTAATAAACAGAACTTCTGACCAACAAGATCCGGCAAAGCCGATCAACGAACCGA
GTTACCCTAGGGATAACAGCGCAATCCTCTTTTAGAGCCCATATCGACAAGAGGGTTTACGACCTCGATGTTGGATCAGGACATC
CTAATGGTGCAACCGCTATTAAGGGTTCGTT

No.2025-166-25-21

GCCCTGTGACCATGAGTTCAACGGCCGCGGTATTTTGACCGTGCAAAGGTAGCGCAATCACTTGTCTTTTAAATGAAGACCCGT
ATGAATGGCAAGACGAGGGCTTAGCTGTCTCCTTTTTCAAGTCAATGAAATTGATCTTCCCGTGCAGAAGCGGGAATCCCCCCA
TAAGACGAGAAGACCCTATGGAGCTTTAGACACGAAGACAGGCCACGTCAAACCCCCTAACTAAAGAATTGAACTAAATGGAC
CCTGCCCTAATGTCTTTGGTTGGGGCGACCACGGGGAAAGACAAAACCCCCGCGTGGAATGGAAGCACCCCTTGCTTCTGCAA
CCAAGAGCTCCCGCTCTAATAAACAGAACTTCTGACCAACAAGATCCGGCAAAGCCGATCAACGAACCGAGTTACCCTAGGGAT
AACAGCGCAATCCTCTTTTAGAGCCCATATCGACAAGAGGGTTTACGACCTCGATGTTGGATCAGGACATCCTAATGGTGCAAC
CGCTATTAA

No.2025-166-25-22

AGAGGTCCTGCCTGCCCTGTGACCATGAGTTCAACGGCCGCGGTATTTTGACCGTGCAAAGGTAGCGCAATCACTTGTCTTTTA
AATGAAGACCCGTATGAATGGCAAGACGAGGGCTTAGCTGTCTCCTTTTTCAAGTCAATGAAATTGATCTTCCCGTGCAGAAGC
GGGAATCCCCCCATAAGACGAGAAGACCCTATGGAGCTTTAGACACGAAGACAGGCCACGTCAAACCCCCTAACTAAAGAATT
GAACTAAATGGACCCTGCCCTAATGTCTTTGGTTGGGGCGACCACGGGGAAAGACAAAACCCCCGCGTGGAATGGAAGCACC
CCTTGCTTCTGCAACCAAGAGCTCCCGCTCTAATAAACAGAACTTCTGACCAACAAGATCCGGCAAAGCCGATCAACGAACCGA
GTTACCCTAGGGATAACAGCGCAATCCTCTTTTAGAGCCCATATCGACAAGAGGGTTTACGACCTCGATGTTGGATCAGGACATC
CTAATGGTGCAACCGCTATTAAGGGT

No.2025-166-25-25

AGAGGTCCTGCCTGCCCTGTGACCATGAGTTCAACGGCCGCGGTATTTTGACCGTGCAAAGGTAGCGCAATCACTTGTCTTTTA
AATGAAGACCCGTATGAATGGCAAGACGAGGGCTTAGCTGTCTCCTTTTTCAAGTCAATGAAATTGATCTTCCCGTGCAGAAGC
GGGAATCCCCCCATAAGACGAGAAGACCCTATGGAGCTTTAGACACGAAGACAGGCCACGTCAAACCCCCTAACTAAAGAATT
GAACTAAATGGACCCTGCCCTAATGTCTTTGGTTGGGGCGACCACGGGGAAAGACAAAACCCCCGCGTGGAATGGAAGCACC
CCTTGCTTCTGCAACCAAGAGCTCCCGCTCTAATAAACAGAACTTCTGACCAACAAGATCCGGCAAAGCCGATCAACGAACCGA
GTTACCCTAGGGATAACAGCGCAATCCTCTTTTAGAGCCCATATCGACAAGAGGGTTTACGACCTCGATGTTGGATCAGGACATC
CTAATGGTGCAACCGCTATTAAGGGTTCGTT

No.2025-166-25-26

AGAGGTCCTGCCTGCCCTGTGACCATGAGTTCAACGGCCGCGGTATTTTGACCGTGCAAAGGTAGCGCAATCACTTGTCTTTTA
AATGAAGACCCGTATGAATGGCAAGACGAGGGCTTAGCTGTCTCCTTTTTCAAGTCAATGAAATTGATCTTCCCGTGCAGAAGC
GGGAATCCCCCCATAAGACGAGAAGACCCTATGGAGCTTTAGACACGAAGACAGGCCACGTCAAACCCCCTAACTAAAGAATT
GAACTAAATGGACCCTGCCCTAATGTCTTTGGTTGGGGCGACCACGGGGAAAGACAAAACCCCCGCGTGGAATGGAAGCACC
CCTTGCTTCTGCAACCAAGAGCTCCCGCTCTAATAAACAGAACTTCTGACCAACAAGATCCGGCAAAGCCGATCAACGAACCGA
GTTACCCTAGGGATAACAGCGCAATCCTCTTTTAGAGCCCATATCGACAAGAGGGTTTACGACCTCGATGTTGGATCAGGACATC
CTAATGGTGCAACCGCTATTAAGGGT

No.2025-166-25-27

GCCCTGTGACCATGAGTTCAACGGCCGCGGTATTTTGACCGTGCAAAGGTAGCGCAATCACTTGTCTTTTAAATGAAGACCCGT
ATGAATGGCAAGACGAGGGCTTAGCTGTCTCCTTTTTCAAGTCAATGAAATTGATCTTCCCGTGCAGAAGCGGGAATCCCCCCA
TAAGACGAGAAGACCCTATGGAGCTTTAGACACGAAGACAGGCCACGTCAAACCCCCTAACTAAAGAATTGAACTAAATGGAC
CCTGCCCTAATGTCTTTGGTTGGGGCGACCACGGGGAAAGACAAAACCCCCGCGTGGAATGGAAGCACCCCTTGCTTCTGCAA
CCAAGAGCTCCCGCTCTAATAAACAGAACTTCTGACCAACAAGATCCGGCAAAGCCGATCAACGAACCGAGTTACCCTAGGGAT
AACAGCGCAATCCTCTTTTAGAGCCCATATCGACAAGAGGGTTTACGACCTCGATGTTGGATCAGGACATCCTAATGGTGCAAC
CGCTATTAA

No.2025-166-25-29

AGAGGTCCTGCCTGCCCTGTGACCATGAGTTCAACGGCCGCGGTATTTTGACCGTGCAAAGGTAGCGCAATCACTTGTCTTTTA
AATGAAGACCCGTATGAATGGCAAGACGAGGGCTTAGCTGTCTCCTTTTTCAAGTCAATGAAATTGATCTTCCCGTGCAGAAGC
GGGAATCCCCCCATAAGACGAGAAGACCCTATGGAGCTTTAGACACGAAGACAGGCCACGTCAAACCCCCTAACTAAAGAATT
GAACTAAATGGACCCTGCCCTAATGTCTTTGGTTGGGGCGACCACGGGGAAAGACAAAACCCCCGCGTGGAATGGAAGCACC
CCTTGCTTCTGCAACCAAGAGCTCCCGCTCTAATAAACAGAACTTCTGACCAACAAGATCCGGCAAAGCCGATCAACGAACCGA
GTTACCCTAGGGATAACAGCGCAATCCTCTTTTAGAGCCCATATCGACAAGAGGGTTTACGACCTCGATGTTGGATCAGGACATC
CTAATGGTGCAACCGCTATTAAGGGTTCGTT

No.2025-166-25-30

GCCCTGTGACCATGAGTTCAACGGCCGCGGTATTTTGACCGTGCAAAGGTAGCGCAATCACTTGTCTTTTAAATGAAGACCCGT

ATGAATGGCAAGACGAGGGCTTAGCTGTCTCCTTTTTCAAGTCAATGAAATTGATCTTCCCGTGCAGAAGCGGGAATCCCCCCA
TAAGACGAGAAGACCCTATGGAGCTTTAGACACGAAGACAGGCCACGTCAAACCCCCTAACTAAAGAATTGAACTAAATGGAC
CCTGCCCTAATGTCTTTGGTTGGGGCGACCACGGGGAAAGACAAAACCCCCGCGTGGAATGGAAGCACCCCTTGCTTCTGCAA
CCAAGAGCTCCCGCTCTAATAAACAGAACTTCTGACCAACAAGATCCGGCAAAGCCGATCAACGAACCGAGTTACCCTAGGGAT
AACAGCGCAATCCTCTTTTAGAGCCCATATCGACAAGAGGGTTTACGACCTCGATGTTGGATCAGGACATCCTAATGGTGCAAC
CGCTATTAA

No.2025-166-25-32

GCCCTGTGACCATGAGTTCAACGGCCGCGGTATTTTGACCGTGCAAAGGTAGCGCAATCACTTGTCTTTTAAATGAAGACCCGT
ATGAATGGCAAGACGAGGGCTTAGCTGTCTCCTTTTTCAAGTCAATGAAATTGATCTTCCCGTGCAGAAGCGGGAATCCCCCCA
TAAGACGAGAAGACCCTATGGAGCTTTAGACACGAAGACAGGCCACGTCAAACCCCCTAACTAAAGAATTGAACTAAATGGAC
CCTGCCCTAATGTCTTTGGTTGGGGCGACCACGGGGAAAGACAAAACCCCCGCGTGGAATGGAAGCACCCCTTGCTTCTGCAA
CCAAGAGCTCCCGCTCTAATAAACAGAACTTCTGACCAACAAGATCCGGCAAAGCCGATCAACGAACCGAGTTACCCTAGGGAT
AACAGCGCAATCCTCTTTTAGAGCCCATATCGACAAGAGGGTTTACGACCTCGATGTTGGATCAGGACATCCTAATGGTGCAAC
CGCTATTAA

No.2025-166-25-42

AGAGGTCCTGCCTGCCCTGTGACCATGAGTTCAACGGCCGCGGTATTTTGACCGTGCAAAGGTAGCGCAATCACTTGTCTTTTA
AATGAAGACCCGTATGAATGGCAAGACGAGGGCTTAGCTGTCTCCTTTTTCAAGTCAATGAAATTGATCTTCCCGTGCAGAAGC
GGGAATCCCCCCATAAGACGAGAAGACCCTATGGAGCTTTAGACACGAAGACAGGCCACGTCAAACCCCCTAACTAAAGAATT
GAACTAAATGGACCCTGCCCTAATGTCTTTGGTTGGGGCGACCACGGGGAAAGACAAAACCCCCGCGTGGAATGGAAGCACC
CCTTGCTTCTGCAACCAAGAGCTCCCGCTCTAATAAACAGAACTTCTGACCAACAAGATCCGGCAAAGCCGATCAACGAACCGA
GTTACCCTAGGGATAACAGCGCAATCCTCTTTTAGAGCCCATATCGACAAGAGGGTTTACGACCTCGATGTTGGATCAGGACATC
CTAATGGTGCAACCGCTATTAAGGGT

No.2025-166-25-43

AGAGGTCCTGCCTGCCCTGTGACCATGAGTTCAACGGCCGCGGTATTTTGACCGTGCAAAGGTAGCGCAATCACTTGTCTTTTA
AATGAAGACCCGTATGAATGGCAAGACGAGGGCTTAGCTGTCTCCTTTTTCAAGTCAATGAAATTGATCTTCCCGTGCAGAAGC
GGGAATCCCCCCATAAGACGAGAAGACCCTATGGAGCTTTAGACACGAAGACAGGCCACGTCAAACCCCCTAACTAAAGAATT
GAACTAAATGGACCCTGCCCTAATGTCTTTGGTTGGGGCGACCACGGGGAAAGACAAAACCCCCGCGTGGAATGGAAGCACC
CCTTGCTTCTGCAACCAAGAGCTCCCGCTCTAATAAACAGAACTTCTGACCAACAAGATCCGGCAAAGCCGATCAACGAACCGA
GTTACCCTAGGGATAACAGCGCAATCCTCTTTTAGAGCCCATATCGACAAGAGGGTTTACGACCTCGATGTTGGATCAGGACATC
CTAATGGTGC

No.2025-166-25-45

AGAGGTCCTGCCTGCCCTGTGACCATGAGTTCAACGGCCGCGGTATTTTGACCGTGCAAAGGTAGCGCAATCACTTGTCTTTTA
AATGAAGACCCGTATGAATGGCAAGACGAGGGCTTAGCTGTCTCCTTTTTCAAGTCAATGAAATTGATCTTCCCGTGCAGAAGC
GGGAATCCCCCCATAAGACGAGAAGACCCTATGGAGCTTTAGACACGAAGACAGGCCACGTCAAACCCCCTAACTAAAGAATT
GAACTAAATGGACCCTGCCCTAATGTCTTTGGTTGGGGCGACCACGGGGAAAGACAAAACCCCCGCGTGGAATGGAAGCACC
CCTTGCTTCTGCAACCAAGAGCTCCCGCTCTAATAAACAGAACTTCTGACCAACAAGATCCGGCAAAGCCGATCAACGAACCGA
GTTACCCTAGGGATAACAGCGCAATCCTCTTTTAGAGCCCATATCGACAAGAGGGTTTACGACCTCGATGTTGGATCAGGACATC
CTAATGGTGCAACCGCTATTAAGGGT

No.2025-166-25-56

AGAGGTCCTGCCTGCCCTGTGACCATGAGTTCAACGGCCGCGGTATTTTGACCGTGCAAAGGTAGCGCAATCACTTGTCTTTTA
AATGAAGACCCGTATGAATGGCAAGACGAGGGCTTAGCTGTCTCCTTTTTCAAGTCAATGAAATTGATCTTCCCGTGCAGAAGC
GGGAATCCCCCCATAAGACGAGAAGACCCTATGGAGCTTTAGACACGAAGACAGGCCACGTCAAACCCCCTAACTAAAGAATT
GAACTAAATGGACCCTGCCCTAATGTCTTTGGTTGGGGCGACCACGGGGAAAGACAAAACCCCCGCGTGGAATGGAAGCACC
CCTTGCTTCTGCAACCAAGAGCTCCCGCTCTAATAAACAGAACTTCTGACCAACAAGATCCGGCAAAGCCGATCAACGAACCGA
GTTACCCTAGGGATAACAGCGCAATCCTCTTTTAGAGCCCATATCGACAAGAGGGTTTACGACCTCGATGTTGGATCAGGACATC
CTAATGGTGCAACCGCTATTAAGGGT

No.2025-166-25-57

AGAGGTCCTGCCTGCCCTGTGACCATGAGTTCAACGGCCGCGGTATTTTGACCGTGCAAAGGTAGCGCAATCACTTGTCTTTTA
AATGAAGACCCGTATGAATGGCAAGACGAGGGCTTAGCTGTCTCCTTTTTCAAGTCAATGAAATTGATCTTCCCGTGCAGAAGC
GGGAATCCCCCCATAAGACGAGAAGACCCTATGGAGCTTTAGACACGAAGACAGGCCACGTCAAACCCCCTAACTAAAGAATT
GAACTAAATGGACCCTGCCCTAATGTCTTTGGTTGGGGCGACCACGGGGAAAGACAAAACCCCCGCGTGGAATGGAAGCACC
CCTTGCTTCTGCAACCAAGAGCTCCCGCTCTAATAAACAGAACTTCTGACCAACAAGATCCGGCAAAGCCGATCAACGAACCGA
GTTACCCTAGGGATAACAGCGCAATCCTCTTTTAGAGCCCATATCGACAAGAGGGTTTACGACCTCGATGTTGGATCAGGACATC
CTAATGGTGCAACCGCTATTAAGGGT

Shark fin: COI gene - Small-sized samples (n=64)

No.2025-166-26-1

TGAGCAGGAATAGTGGGAACAGCCCTCAGCCTTCTAATTCGAGCCGAGTTAGGCCAGCCCGGATCACTCCTAGGGGATGATCA
GGTCTATAATGTTATCGTAACCGCCCATGCATTTGTAATAATCTTCTTCATGGTTATACCCGTAATAATTGGGGGATTTGGAAACTG
ATTAGTACCCTTAATAATTGGTGCACCAGACATGGCCTTCCCGCGAATAAATAACATAAGCTTTTGACTCCTTCCCCCTTCTTTTCT
CTTACTCCTAGCTTCAGCTGGGGTTGAAGCTGGAGCTGGCACTGGTTGAACAGTTTATCCCCCCTTAGCTGGCAACTTAGCACA
TGCTGGGGCATCTGTTGACTTGGCCATTTTCTCGCTTCATTTAGCAGGTATCTCATCAATTTTAGCTTCAATTAACTTTATTACAAC
TATCATTAATATAAAACCACCAGCCATCTCTCAATATCAAACACCACTATTTGTATGATCAATCCTAGTAACAACCATCCTCCTCCTC
TTATCCCTCCCAGTACTCGCAGCCGGCATCACAATAT

No.2025-166-26-2

ATGAGCAGGAATAGTGGGAACAGCCCTCAGCCTTCTAATTCGAGCCGAGTTAGGCCAGCCCGGATCACTCCTAGGGGATGATC
AGGTCTATAATGTTATCGTAACCGCCCATGCATTTGTAATAATCTTCTTCATGGTTATACCCGTAATAATTGGGGGATTTGGAAACT
GATTAGTACCCTTAATAATTGGTGCACCAGACATGGCCTTCCCGCGAATAAATAACATAAGCTTTTGACTCCTTCCCCCTTCTTTTC
TCTTACTCCTAGCTTCAGCTGGGGTTGAAGCTGGAGCTGGCACTGGTTGAACAGTTTATCCCCCCTTAGCTGGCAACTTAGCAC
ATGCTGGGGCATCTGTTGACTTGGCCATTTTCTCGCTTCATTTAGCAGGTATCTCATCAATTTTAGCTTCAATTAACTTTATTACAA
CTATCATTAATATAAAACCACCAGCCATCTCTCAATATCAAACACCATTATTTGTATGATCAATCCTAGTAACAACCATCCTCCTCCTC
TTATCCCTCCCAGTACTCGCAGCCGGCATCACAATAT

No.2025-166-26-3

TGAGCAGGAATAGTGGGAACAGCCCTCAGCCTTCTAATTCGAGCCGAGTTAGGCCAGCCCGGATCACTCCTAGGGGATGATCA
GGTCTATAATGTTATCGTAACCGCCCATGCATTTGTAATAATCTTCTTCATGGTTATACCCGTAATAATTGGGGGATTTGGAAACTG
ATTAGTACCCTTAATAATTGGTGCACCAGACATGGCCTTCCCGCGAATAAATAACATAAGCTTTTGACTCCTTCCCCCTTCTTTTCT
CTTACTCCTAGCTTCAGCTGGGGTTGAAGCTGGAGCTGGCACTGGTTGAACAGTTTATCCCCCCTTAGCTGGCAACTTAGCACA
TGCTGGGGCATCTGTTGACTTGGCCATTTTCTCGCTTCATTTAGCAGGTATCTCATCAATTTTAGCTTCAATTAACTTTATTACAAC
TATCATTAATATAAAACCACCAGCCATCTCTCAATATCAAACACCATTATTTGTATGATCAATCCTAGTAACAACCATCCTCCTCCTCT
TATCCCTCCCAGTACTCGCAGCCGGCATCACAATAT

No.2025-166-26-4

GGGACAGCCCATCAGCCTTCTAATTCGAGCCGAGTTAGGCCAGCCCGGATCACTCCTAGGGGATGATCAGGTCTATAATGTTATC
GTAACCGCCCATGCATTTGTAATAATCTTCTTCATGGTTATACCCGTAATAATTGGGGGATTTGGAAACTGATTAGTACCCTTAATA
ATTGGTGCACCAGACATGGCCTTCCCGCGAATAAATAACATAAGCTTTTGACTCCTTCCCCCTTCTTTTCTCTTACTCCTAGCTTCA
GCTGGGGTTGAAGCTGGAGCTGGCACTGGTTGAACAGTTTATCCCCCCTTAGCTGGCAACTTAGCACATGCTGGGGCATCTGT
TGACTTAGCCATTTTCTCGCTTCATTTAGCAGGTATCTCATCAATTTTAGCTTCAATTAACTTTATTACAACTATCATTAATATAAAAC
CACCAGCCATCTCTCAATATCAAACACCATTATTTGTATGATCAATCCTAGTAACAACCATCCTCCTCCTCTTATCCCTCCCAGTACT
CGCAGCCGGCATCACAATATTATTAACTGATCGAAACCTAAACACAACATT

No.2025-166-26-5

TGAGCAGGATAAGTGGGAACAGCCCTCAGCCTTCTAATTCGAGCCGAGTTAGGCCAGCCCGGATCACTCCTAGGGGATGATCA
GGTCTATAATGTTATCGTAACCGCCCATGCATTTGTAATAATCTTCTTCATGGTTATACCCGTAATAATTGGGGGATTTGGAAACTG
ATTAGTACCCTTAATAATTGGTGCACCAGACATGGCCTTCCCGCGAATAAATAACATAAGCTTTTGACTCCTTCCCCCTTCTTTTCT
CTTACTCCTAGCTTCAGCTGGGGTTGAAGCTGGAGCTGGCACTGGTTGAACAGTTTATCCCCCCTTAGCTGGCAACTTAGCACA
TGCTGGGGCATCTGTTGACTTGGCCATTTTCTCGCTTCATTTAGCAGGTATCTCATCAATTTTAGCTTCAATTAACTTTATTACAAC
TATCATTAATATAAAACCACCAGCCATCTCTCAATATCAAACACCATTATTTGTATGATCAATCCTAGTAACAACCATCCTCCTCCTCT
TATCCCTCCCAGTACTCGCAGCCGGCATCACAATAT

No.2025-166-26-6

ATGAGCAGGAATAGTGGGGACGGCCCTAAGCCTCTTAATTCGAGCCGAATTAGGACAGCCAGGATCACTTCTAGGAGATGATC
AAATCTATAATGTTATTGTAACCGCCCATGCATTCGTAATAATCTTCTTTATAGTTATACCCGTAATAATTGGCGGATTTGGAAACTG
ACTAGTGCCATTAATAATTGGTGCACCAGACATAGCTTTTCCACGAATAAATAATATAAGCTTTTGACTCCTTCCCCCCTCTTTTCT
TTTACTTCTAGCTTCAGCTGGAGTTGAAGCCGGAGCCGGTACTGGTTGAACAGTTTATCCTCCATTAGCTGGCAATTTAGCACAT
GCTGGGGCATCCGTTGACTTAGCTATTTTCTCTCTCCATTTAGCAGGTATTTCATCAATTTTAGCCTCAATCAACTTTATTACAACTA
TTATTAATATAAAACCCCCTGCAATCTCCCAATATCAAACACCACTATTTGTGTGATCAATTCTAGTAACAACTATCCTCCTTCTATTA
TCCCTCCCAGTACTTGCAGCCGGCATTACAATACTACT

No.2025-166-26-7

ATGAGCAGGAATAGTGGGAACAGCCCTCAGCCTTCTAATTCGAGCCGAGTTAGGCCAGCCCGGATCACTCCTAGGGGATGATC
AGGTCTATAATGTTATCGTAACCGCCCATGCATTTGTAATAATCTTCTTCATGGTTATACCCGTAATAATTGGGGGATTTGGAAACT
GATTAGTACCCTTAATAATTGGTGCACCAGACATGGCCTTCCCGCGAATAAATAACATAAGCTTTTGACTCCTTCCCCCTTCTTTTC
TCTTACTCCTAGCTTCAGCTGGGGTTGAAGCTGGAGCTGGCACTGGTTGAACAGTTTATCCCCCCTTAGCTGGCAACTTAGCAC
ATGCTGGGGCATCTGTTGACTTGGCCATTTTCTCGCTTCATTTAGCAGGTATCTCATCAATTTTAGCTTCAATTAACTTTATTACAA
CTATCATTAATATAAAACCACCAGCCATCTCTCAATATCAAACACCATTATTTGTATGATCAATCCTAGTAACAACCATCCTCCTCCTC
TTATCCCTCCCAGTACTCGCAGCCGGCATCACAATAT

No.2025-166-26-8

TGAGCAGGAATAGTGGGAACAGCCCTCAGCCTTCTAATTCGAGCCGAGTTAGGCCAGCCCGGATCACTCCTAGGGGATGATCA
GGTCTATAATGTTATCGTAACCGCCCATGCATTTGTAATAATCTTCTTCATGGTTATACCCGTAATAATTGGGGGATTTGGAAACTG
ATTAGTACCCTTAATAATTGGTGCACCAGACATGGCCTTCCCGCGAATAAATAACATAAGCTTTTGACTCCTTCCCCCTTCTTTTCT

CTTACTCCTAGCTTCAGCTGGGGTTGAAGCTGGAGCTGGCACTGGTTGAACAGTTTATCCCCCCTTAGCTGGCAACTTAGCACA
TGCTGGGGCATCTGTTGACTTGGCCATTTTCTCGCTTCATTTAGCAGGTATCTCATCAATTTTAGCTTCAATTAACTTTATTACAAC
TATCATTAATATAAAACCACCAGCCATCTCTCAATATCAAACACCATTATTTGTATGATCAATCCTAGTAACAACCATCCTCCTCCTCT
TATCCCTCCCAGTACTCGCAGCCGGCATCACAATATTATTAACT

No.2025-166-26-9

TGAGCAGGAATAGTGGGAACAGCCCTCAGCCTTCTAATTCGAGCCGAGTTAGGCCAGCCCGGATCACTCCTAGGGGATGATCA
GGTCTATAATGTTATCGTAACCGCCCATGCATTTGTAATAATCTTCTTCATGGTTATACCCGTAATAATTGGGGGATTTGGAAACTG
ATTAGTACCCTTAATAATTGGTGCACCAGACATGGCCTTCCCGCGAATAAATAACATAAGCTTTTGACTCCTTCCCCCTTCTTTTCT
CTTACTCCTAGCTTCAGCTGGGGTTGAAGCTGGAGCTGGCACTGGTTGAACAGTTTATCCCCCCTTAGCTGGCAACTTAGCACA
TGCTGGGGCATCTGTTGACTTGGCCATTTTCTCGCTTCATTTAGCAGGTATCTCATCAATTTTAGCTTCAATTAACTTTATTACAAC
TATCATTAATATAAAACCACCAGCCATCTCTCAATATCAAACACCATTATTTGTATGATCAATCCTAGTAACAACCATCCTCCTCCTCT
TATCCCTCCCAGTACTCGCAGCCGGCATCACAATATTAT

No.2025-166-26-10

ATGAGCAGGAATAGTGGGACCAGCCCTCAGCCTTCTAATTCGAGCCGAGTTAGGCCAGCCCGGATCACTCCTAGGGGATGATC
AGGTCTATAATGTTATCGTAACCGCCCATGCATTTGTAATAATCTTCTTCATGGTTATACCCGTAATAATTGGGGGATTTGGAAACT
GATTAGTACCCTTAATAATTGGTGCACCAGACATGGCCTTCCCGCGAATAAATAACATAAGCTTTTGACTCCTTCCCCCTTCTTTTC
TCTTACTCCTAGCTTCAGCTGGGGTTGAAGCTGGAGCTGGCACTGGTTGAACAGTTTATCCCCCCTTAGCTGGCAACTTAGCAC
ATGCTGGGGCATCTGTTGACTTGGCCATTTTCTCGCTTCATTTAGCAGGTATCTCATCAATTTTAGCTTCAATTAACTTTATTACAA
CTATCATTAATATAAAACCACCAGCCATCTCTCAATATCAAACACCATTATTTGTATGATCAATCCTAGTAACAACCATCCTCCTCCTC
TTATCCCTCCCAGTACTCGCAGCCGGCATCACAATA

No.2025-166-26-11

ACAGCCCTCAGCCTTCTAATTCGAGCCGAGTTAGGCCAGCCCGGATCACTCCTAGGGGATGATCAGGTCTATAATGTTATCGTAA
CCGCCCATGCATTTGTAATAATCTTCTTCATGGTTATACCCGTAATAATTGGGGGATTTGGAAACTGATTAGTACCCTTAATAATTG
GTGCACCAGACATGGCCTTCCCGCGAATAAATAACATAAGCTTTTGACTCCTTCCCCCTTCTTTTCTCTTACTCCTAGCTTCAGCT
GGGGTTGAAGCTGGAGCTGGCACTGGTTGAACAGTTTATCCCCCCTTAGCTGGCAACTTAGCACATGCTGGGGCATCTGTTGA
CTTGGCCATTTTCTCGCTTCATTTAGCAGGTATCTCATCAATTTTAGCTTCAATTAACTTTATTACAACTATCATTAATATAAAACCAC
CAGCCATCTCTCAATATCAAACACCATTATTTGTATGATCAATCCTAGTAACAACCATCCTCCTCCTCTTATCCCTCCCAGTACTCGC
AGCCGGCATCACAATATTATTAACTGATCGAAACCTAAACACAACATTC

No.2025-166-26-12

ATGAGCAGGAATAGTGGGAACAGCCCTAAGCCTCTTAATTCGAGCCGAATTAGGACAGCCAGGATCACTTCTAGGAGATGATC
AAATCTATAATGTTATTGTAACCGCCCATGCATTCGTAATAATCTTCTTTATAGTTATACCCGTGATAATTGGCGGATTTGGAAACTG
ACTAGTGCCATTAATAATTGGTGCACCAGACATAGCTTTTCCACGAATAAATAATATAAGCTTTTGACTCCTTCCCCCCTCTTTTCT
TTTACTTCTAGCTTCAGCTGGAGTTGAAGCCGGAGCCGGTACTGGTTGAACAGTTTATCCTCCATTAGCTGGCAATTTAGCACAT
GCTGGAGCATCCGTTGACTTAGCTATTTTCTCTCTCCATTTAGCAGGTATTTCATCAATTTTAGCCTCAATCAACTTTATTACAACTA
TTATTAATATAAAACCCCCTGCAATCTCCCAATATCAAACACCATTATTTGTGTGATCAATTCTAGTAACAACTATTCTCCTTCTATTA
TCCCTCCCAGTACTTGCAGCCGGCATTACAATACTACT

No.2025-166-26-13

ATGAGCAGGAATAGTGGGGACAGCCCTAAGCCTCTTAATTCGAGCCGAATTAGGACAGCCAGGATCACTTCTAGGAGATGATC
AAATCTATAATGTTATTGTAACCGCCCATGCATTCGTAATAATCTTCTTTATAGTTATACCCGTAATAATTGGCGGATTTGGAAACTG
ACTAGTGCCATTAATAATTGGTGCACCAGACATAGCTTTTCCACGAATAAATAATATAAGCTTTTGACTCCTTCCCCCCTCTTTTCT
TTTACTTCTAGCTTCAGCTGGAGTTGAAGCCGGAGCCGGTACTGGTTGAACAGTTTATCCTCCATTAGCTGGCAATTTAGCACAT
GCTGGGGCATCCGTTGACTTAGCTATTTTCTCTCTCCATTTAGCAGGTATTTCATCAATTTTAGCCTCAATCAACTTTATTACAACTA
TTATTAATATAAAACCCCCTGCAATCTCCCAATATCAAACACCACTATTTGTGTGATCAATTCTAGTAACAACTATCCTCCTTCTATTA
TCCCTCCCAGTACTTGCAGCCGGCATTACAATACTACT

No.2025-166-26-14

ATGAGCAGGAATAGTGGGGACGGCCCTAAGCCTCTTAATTCGAGCCGAATTAGGACAGCCAGGATCACTTCTAGGAGATGATC
AAATCTATAATGTTATTGTAACCGCCCATGCATTCGTAATAATCTTCTTTATAGTTATACCCGTAATAATTGGCGGATTTGGAAACTG
ACTAGTGCCATTAATAATTGGTGCACCAGACATAGCTTTTCCACGAATAAATAATATAAGCTTTTGACTCCTTCCCCCCTCTTTTCT
TTTACTTCTAGCTTCAGCTGGAGTTGAAGCCGGAGCCGGTACTGGTTGAACAGTTTATCCTCCATTAGCTGGCAATTTAGCACAT
GCTGGGGCATCCGTTGACTTAGCTATTTTCTCTCTCCATTTAGCAGGTATTTCATCAATTTTAGCCTCAATCAACTTTATTACAACTA
TTATTAATATAAAACCCCCTGCAATCTCCCAATATCAAACACCACTATTTGTGTGATCAATTCTAGTAACAACTATCCTCCTTCTATTA
TCCCTCCCAGTACTTGCAGCCGGCATTACAATACTACT

No.2025-166-26-15

CCCTAAGCCTCTTAATTCGAGCCGAATTAGGACAGCCAGGATCACTTCTAGGAGATGATCAAATCTATAATGTTATTGTAACCGCC
CATGCATTCGTAATAATCTTCTTTATAGTTATACCCGTAATAATTGGCGGATTTGGAAACTGACTAGTGCCATTAATAATTGGTGCA
CCAGACATAGCTTTTCCACGAATAAATAATATAAGCTTTTGACTCCTTCCCCCCTCTTTTCTTTTACTTCTAGCTTCAGCTGGAGTT
GAAGCCGGAGCCGGTACTGGTTGAACAGTTTATCCTCCATTAGCTGGCAATTTAGCACATGCTGGGGCATCCGTTGACTTAGCT
ATTTTCTCTCTCCATTTAGCAGGTATTTCATCAATTTTAGCCTCAATCAACTTTATTACAACTATTATTAATATAAAACCCCCTGCAAT
CTCCCAATATCAAACACCACTATTTGTGTGATCAATTCTAGTAACAACTATCCTCCTTCTATTATCCCTCCCAGTACTTGCAGCCGGC
ATTACAATACTACT

No.2025-166-26-16

TGAGCAGGAATAGTGGGGACAGCCCTAAGCCTCTTAATTCGAGCCGAATTAGGACAGCCAGGATCACTTCTAGGAGATGATCA
AATCTATAATGTTATTGTAACCGCCCATGCATTCGTAATAATCTTCTTTATAGTTATACCCGTAATAATTGGCGGATTTGGAAACTGA

CTAGTGCCATTAATAATTGGTGCACCAGACATAGCTTTTCCACGAATAAATAATATAAGCTTTTGACTCCTTCCCCCCTCTTTTCTT
TTACTTCTAGCTTCAGCTGGAGTTGAAGCCGGAGCCGGTACTGGTTGAACAGTTTATCCTCCATTAGCTGGCAATTTAGCACATG
CTGGGGCATCCGTTGACTTAGCTATTTTCTCTCTCCATTTAGCAGGTATTTCATCAATTTTAGCCTCAATCAACTTTATTACAACTAT
TATTAATATAAAACCCCCTGCAATCTCCCAATATCAAACACCACTATTTGTGTGATCAATTCTAGTAACAACTATCCTCCTTCTATTAT
CCCTCCCAGTACTTGCAGCCGGCATTACAATACTACT

No.2025-166-26-17

TGAGCAGGAATAGTGGGGACGGCCCTAAGCCTCTTAATTCGAGCCGAATTAGGACAGCCAGGATCACTTCTAGGAGATGATCA
AATCTATAATGTTATTGTAACCGCCCATGCATTCGTAATAATCTTCTTTATAGTTATACCCGTAATAATTGGCGGATTTGGAAACTGA
CTAGTGCCATTAATAATTGGTGCACCAGACATAGCTTTTCCACGAATAAATAATATAAGCTTTTGACTCCTTCCCCCCTCTTTTCTT
TTACTTCTAGCTTCAGCTGGAGTTGAAGCCGGAGCCGGTACTGGTTGAACAGTTTATCCTCCATTAGCTGGCAATTTAGCACATG
CTGGGGCATCCGTTGACTTAGCTATTTTCTCTCTCCATTTAGCAGGTATTTCATCAATTTTAGCCTCAATCAACTTTATTACAACTAT
TATTAATATAAAACCCCCTGCAATCTCCCAATATCAAACACCACTATTTGTGTGATCAATTCTAGTAACAACTATCCTCCTTCTATTAT
CCCTCCCAGTACTTGCAGCCGGCATTACAATACTACT

No.2025-166-26-18

GGATCACTCCTAGGGGATGATCAGGTCTATAATGTTATCGTAACCGCCCATGCATTTGTAATAATCTTCTTCATGGTTATACCCGTA
ATAATTGGAGGATTTGGAAACTGATTAGTACCCTTAATAATTGGTGCACCAGACATGGCCTTCCCGCGAATAAATAACATAAGCT
TTTGACTCCTTCCCCCTTCTTTTCTCTTACTCCTAGCTTCAGCTGGGGTTGAAGCTGGAGCTGGCACTGGTTGAACAGTTTATCC
CCCCTTAGCTGGCAACTTAGCACATGCTGGGGCATCTGTTGACTTGGCCATTTTCTCGCTTCATTTAGCAGGTATCTCATCAATTT
TAGCTTCAATTAACTTTATTACAACTATCATTAATATAAAACCACCAGCCATCTCTCAATATCAAACACCATTATTTGTATGATCAATC
CTAGTAACAACCATCCTCCTCCTCTTATCCCTCCCAGTACTCGCAGCCGGCATCACAATATTATTAACTGATCGAAACCTAAACACA
ACATTC

No.2025-166-26-19

TGAGCAGGAATAGTGGGGACGGCCCTAAGCCTCTTAATTCGAGCCGAATTAGGACAGCCAGGATCACTTCTAGGAGATGATCA
AATCTATAATGTTATTGTAACCGCCCATGCATTCGTAATAATCTTCTTTATAGTTATACCCGTAATAATTGGCGGATTTGGAAACTGA
CTAGTGCCATTAATAATTGGTGCACCAGACATAGCTTTTCCACGAATAAATAATATAAGCTTTTGACTCCTTCCCCCCTCTTTTCTT
TTACTTCTAGCTTCAGCTGGAGTTGAAGCCGGAGCCGGTACTGGTTGAACAGTTTATCCTCCATTAGCTGGCAATTTAGCACATG
CTGGGGCATCCGTTGACTTAGCTATTTTCTCTCTCCATTTAGCAGGTATTTCATCAATTTTAGCCTCAATCAACTTTATTACAACTAT
TATTAATATAAAACCCCCTGCAATCTCCCAATATCAAACACCACTATTTGTGTGATCAATTCTAGTAACAACTATCCTCCTTCTATTAT
CCCTCCCAGTACTTGCAGCCGGCATTACAATACTACT

No.2025-166-26-20

AGCAGGAATAGTGGGGACGGCCCTAAGCCTCTTAATTCGAGCCGAATTAGGACAGCCAGGATCACTTCTAGGAGATGATCAAA
TCTATAATGTTATTGTAACCGCCCATGCATTCGTAATAATCTTCTTTATAGTTATACCCGTAATAATTGGCGGATTTGGAAACTGACT
AGTGCCATTAATAATTGGTGCACCAGACATAGCTTTTCCACGAATAAATAATATAAGCTTTTGACTCCTTCCCCCCTCTTTTCTTTT
ACTTCTAGCTTCAGCTGGAGTTGAAGCCGGAGCCGGTACTGGTTGAACAGTTTATCCTCCATTAGCTGGCAATTTAGCACATGCT
GGGGCATCCGTTGACTTAGCTATTTTCTCTCTCCATTTAGCAGGTATTTCATCAATTTTAGCCTCAATCAACTTTATTACAACTATTA
TTAATATAAAACCCCCTGCAATCTCCCAATATCAAACACCACTATTTGTGTGATCAATTCTAGTAACAACTATCCTCCTTCTATTATCC
CTCCCAGTACTTGCAGCCGGCATTACAATACTACT

No.2025-166-26-21

TGAGCAGGAATAGTGGGGACAGCCCTAAGCCTCTTAATTCGAGCCGAATTAGGACAGCCAGGATCACTTCTAGGAGATGATCA
AATCTATAATGTTATTGTAACCGCCCATGCATTCGTAATAATCTTCTTTATAGTTATACCCGTAATAATTGGCGGATTTGGAAACTGA
CTAGTGCCATTAATAATTGGTGCACCAGACATAGCTTTTCCACGAATAAATAATATAAGCTTTTGACTCCTTCCCCCCTCTTTTCTT
TTACTTCTAGCTTCAGCTGGAGTTGAAGCCGGAGCCGGTACTGGTTGAACAGTTTATCCTCCATTAGCTGGCAATTTAGCACATG
CTGGGGCATCCGTTGACTTAGCTATTTTCTCTCTCCATTTAGCAGGTATTTCATCAATTTTAGCCTCAATCAACTTTATTACAACTAT
TATTAATATAAAACCCCCTGCAATCTCCCAATATCAAACACCACTATTTGTGTGATCAATTCTAGTAACAACTATCCTCCTTCTATTAT
CCCTCCCAGTACTTGCAGCCGGCATTACAATACTACT

No.2025-166-26-22

TGAGCAGGAATAGTGGGGACGGCCCTAAGCCTCTTAATTCGAGCCGAATTAGGACAGCCAGGATCACTTCTAGGAGATGATCA
AATCTATAATGTTATTGTAACCGCCCATGCATTCGTAATAATCTTCTTTATAGTTATACCCGTAATAATTGGCGGATTTGGAAACTGA
CTAGTGCCATTAATAATTGGTGCACCAGACATAGCTTTTCCACGAATAAATAATATAAGCTTTTGACTCCTTCCCCCCTCTTTTCTT
TTACTTCTAGCTTCAGCTGGAGTTGAAGCCGGAGCCGGTACTGGTTGAACAGTTTATCCTCCATTAGCTGGCAATTTAGCACATG
CTGGGGCATCCGTTGACTTAGCTATTTTCTCTCTCCATTTAGCAGGTATTTCATCAATTTTAGCCTCAATCAACTTTATTACAACTAT
TATTAATATAAAACCCCCTGCAATCTCCCAATATCAAACACCACTATTTGTGTGATCAATTCTAGTAACAACTATCCTCCTTCTATTAT
CCCTCCCAGTACTTGCAGCCGGCATTACAATACTACT

No.2025-166-26-23

TGAGCAGGTATAGTTGGAACAGCCCTAAGTCTTCTAATTCGAGCTGAGCTTGGACAACCTGGATCACTTTTAGGGGATGATCAG
ATTTATAATGTAATCGTAACCGCCCACGCTTTTGTAATAATCTTTTTTATGGTTATGCCAATCATAATTGGTGGTTTCGGAAATTGAC
TAGTTCCTTTAATAATTGGTGCACCAGATATAGCCTTCCCACGAATAAATAACATAAGTTTCTGACTTCTTCCACCATCATTTCTTCT
TCTCCTCGCCTCTGCTGGAGTAGAAGCTGGAGCAGGTACTGGTTGAACAGTTTATCCTCCATTAGCTAGTAACCTAGCACATGCT
GGACCATCTGTTGATTTAGCTATTTTCTCTCTTCACTTAGCCGGTGTGTCATCTATTCTAGCTTCAATTAATTTTATTACAACTATTAT
CAATATAAAACCACCAGCCATTTCCCAATATCAAACACCATTATTTGTTTGATCTATTCTTGTAACCACTATTCTTCTCCTCCTATCAC
TTCCAGTTCTTGCAGCAGGGATTACAATATTACTTACAGATCG

No.2025-166-26-24

TGAGCAGGAATAGTGGGGACGGCCCTAAGCCTCTTAATTCGAGCCGAATTAGGACAGCCAGGATCACTTCTAGGAGATGATCA

AATCTATAATGTTATTGTAACCGCCCATGCATTCGTAATAATCTTCTTTATAGTTATACCCGTAATAATTGGCGGATTTGGAAACTGA
CTAGTGCCATTAATAATTGGTGCACCAGACATAGCTTTTCCACGAATAAATAATATAAGCTTTTGACTCCTTCCCCCCTCTTTTCTT
TTACTTCTAGCTTCAGCTGGAGTTGAAGCCGGAGCCGGTACTGGTTGAACAGTTTATCCTCCATTAGCTGGCAATTTAGCACATG
CTGGGGCATCCGTTGACTTAGCTATTTTCTCTCTCCATTTAGCAGGTATTTCATCAATTTTAGCCTCAATCAACTTTATTACAACTAT
TATTAATATAAAACCCCCTGCAATCTCCCAATATCAAACACCACTATTTGTGTGATCAATTCTAGTAACAACTATCCTCCTTCTATTAT
CCCTCCCAGTACTTGCAGCCGGCATTACAATACTACT

No.2025-166-26-25

TGAGCAGGAATAGTGGGAACAGCCCTCAGCCTTCTAATTCGAGCCGAGTTAGGCCAGCCCGGATCACTCCTAGGGGATGATCA
GGTCTATAATGTTATCGTAACCGCCCATGCATTTGTAATAATCTTCTTCATGGTTATACCCGTAATAATTGGAGGATTTGGAAACTG
ATTAGTACCCTTAATAATTGGTGCACCAGACATGGCCTTCCCGCGAATAAATAACATAAGCTTTTGACTCCTTCCCCCTTCTTTTCT
CTTACTCCTAGCTTCAGCTGGGGTTGAAGCTGGAGCTGGCACTGGTTGAACAGTTTATCCCCCCTTAGCTGGCAACTTAGCACA
TGCTGGGGCATCTGTTGACTTGGCCATTTTCTCGCTTCATTTAGCAGGTATCTCATCAATTTTAGCTTCAATTAACTTTATTACAAC
TATCATTAATATAAAACCACCAGCCATCTCTCAATATCAAACACCATTATTTGTATGATCAATCCTAGTAACAACCATCCTCCTCCTCT
TATCCCTCCCAGTACTCGCAGCCGGCATCACAATATTAT

No.2025-166-26-26

TGAGCAGGAATAGTGGGGACGGCCCTAAGCCTCTTAATTCGAGCCGAATTAGGACAGCCAGGATCACTTCTAGGAGATGATCA
AATCTATAATGTTATTGTAACCGCCCATGCATTCGTAATAATCTTCTTTATAGTTATACCCGTAATAATTGGCGGATTTGGAAACTGA
CTAGTGCCATTAATAATTGGTGCACCAGACATAGCTTTTCCACGAATAAATAATATAAGCTTTTGACTCCTTCCCCCCTCTTTTCTT
TTACTTCTAGCTTCAGCTGGAGTTGAAGCCGGAGCCGGTACTGGTTGAACAGTTTATCCTCCATTAGCTGGCAATTTAGCACATG
CTGGGGCATCCGTTGACTTAGCTATTTTCTCTCTCCATTTAGCAGGTATTTCATCAATTTTAGCCTCAATCAACTTTATTACAACTAT
TATTAATATAAAACCCCCTGCAATCTCCCAATATCAAACACCACTATTTGTGTGATCAATTCTAGTAACAACTATCCTCCTTCTATTAT
CCCTCCCAGTACTTGCAGCCGGCATTACAATACTACT

No.2025-166-26-27

TGAGCAGGAATAGTGGGGACGGCCCTAAGCCTCTTAATTCGAGCCGAATTAGGACAGCCAGGATCACTTCTAGGAGATGATCA
AATCTATAATGTTATTGTAACCGCCCATGCATTCGTAATAATCTTCTTTATAGTTATACCCGTAATAATTGGCGGATTTGGAAACTGA
CTAGTGCCATTAATAATTGGTGCACCAGACATAGCTTTTCCACGAATAAATAATATAAGCTTTTGACTCCTTCCCCCCTCTTTTCTT
TTACTTCTAGCTTCAGCTGGAGTTGAAGCCGGAGCCGGTACTGGTTGAACAGTTTATCCTCCATTAGCTGGCAATTTAGCACATG
CTGGGGCATCCGTTGACTTAGCTATTTTCTCTCTCCATTTAGCAGGTATTTCATCAATTTTAGCCTCAATCAACTTTATTACAACTAT
TATTAATATAAAACCCCCTGCAATCTCCCAATATCAAACACCACTATTTGTGTGATCAATTCTAGTAACAACTATCCTCCTTCTATTAT
CCCTCCCAGTACTTGCAGCCGGCATTACAATACTACT

No.2025-166-26-28

ATAGTGGGAACAGCCCTCAGCCTTCTAATTCGAGCCGAGTTAGGCCAGCCCGGATCACTCCTAGGGGATGATCAGGTCTATAAT
GTTATCGTAACCGCCCATGCATTTGTAATAATCTTCTTCATGGTTATACCCGTAATAATTGGGGGATTTGGAAACTGATTAGTACCC
TTAATAATTGGTGCACCAGACATGGCCTTCCCGCGAATAAATAACATAAGCTTTTGACTCCTTCCCCCTTCTTTTCTCTTACTCCTA
GCTTCAGCTGGGGTTGAAGCTGGAGCTGGCACTGGTTGAACAGTTTATCCCCCCTTAGCTGGCAACTTAGCACATGCTGGGGC
ATCTGTTGACTTGGCCATTTTCTCGCTTCATTTAGCAGGTATCTCATCAATTTTAGCTTCAATTAACTTTATTACAACTATCATTAATA
TAAAACCACCAGCCATCTCTCAATATCAAACACCATTATTTGTATGATCAATCCTAGTAACAACCATCCTCCTCCTCTTATCCCTCCC
AGTACTCGCAGCCGGCATCACAATATTAT

No.2025-166-26-29

TGAGCAGGAATAGTGGGGACGGCCCTAAGCCTCTTAATTCGAGCCGAATTAGGACAGCCAGGATCACTTCTAGGAGATGATCA
AATCTATAATGTTATTGTAACCGCCCATGCATTCGTAATAATCTTCTTTATAGTTATACCCGTAATAATTGGCGGATTTGGAAACTGA
CTAGTGCCATTAATAATTGGTGCACCAGACATAGCTTTTCCACGAATAAATAATATAAGCTTTTGACTCCTTCCCCCCTCTTTTCTT
TTACTTCTAGCTTCAGCTGGAGTTGAAGCCGGAGCCGGTACTGGTTGAACAGTTTATCCTCCATTAGCTGGCAATTTAGCACATG
CTGGGGCATCCGTTGACTTAGCTATTTTCTCTCTCCATTTAGCAGGTATTTCATCAATTTTAGCCTCAATCAACTTTATTACAACTAT
TATTAATATAAAACCCCCTGCAATCTCCCAATATCAAACACCACTATTTGTGTGATCAATTCTAGTAACAACTATCCTCCTTCTATTAT
CCCTCCCAGTACTTGCAGCCGGCATTACAATACTACT

No.2025-166-26-30

GAGCAGGATTAGTGGGACCAGCCCTAAGCCTCTTAATTCGAGCCGAATTAGGACAGCCAGGATCACTTCTAGGAGATGATCAA
ATCTATAATGTTATTGTAACCGCCCATGCATTCGTAATAATCTTCTTTATAGTTATACCCGTGATAATTGGCGGATTTGGAAACTGAC
TAGTGCCATTAATAATTGGTGCACCAGACATAGCTTTTCCACGAATAAATAATATAAGCTTTTGACTCCTTCCCCCCTCTTTTCTTTT
ACTTCTAGCTTCAGCTGGAGTTGAAGCCGGAGCCGGTACTGGTTGAACAGTTTATCCTCCATTAGCTGGCAATTTAGCACATGCT
GGAGCATCCGTTGACTTAGCTATTTTCTCTCTCCATTTAGCAGGTATTTCATCAATTTTAGCCTCAATCAACTTTATTACAACTATTA
TTAATATAAAACCCCCTGCAATCTCCCAATATCAAACACCATTATTTGTGTGATCAATTCTAGTAACAACTATTCTCCTTCTATTATCC
CTCCCAGTACTTGCAGCCGGCATTACAATACTACT

No.2025-166-26-31

TGAGCAGGAATAGTGGGGACGGCCCTAAGCCTCTTAATTCGAGCCGAATTAGGACAGCCAGGATCACTTCTAGGAGATGATCA
AATCTATAATGTTATTGTAACCGCCCATGCATTCGTAATAATCTTCTTTATAGTTATACCCGTAATAATTGGCGGATTTGGAAACTGA
CTAGTGCCATTAATAATTGGTGCACCAGACATAGCTTTTCCACGAATAAATAATATAAGCTTTTGACTCCTTCCCCCCTCTTTTCTT
TTACTTCTAGCTTCAGCTGGAGTTGAAGCCGGAGCCGGTACTGGTTGAACAGTTTATCCTCCATTAGCTGGCAATTTAGCACATG
CTGGGGCATCCGTTGACTTAGCTATTTTCTCTCTCCATTTAGCAGGTATTTCATCAATTTTAGCCTCAATCAACTTTATTACAACTAT
TATTAATATAAAACCCCCTGCAATCTCCCAATATCAAACACCACTATTTGTGTGATCAATTCTAGTAACAACTATCCTCCTTCTATTAT
CCCTCCCAGTACTTGCAGCCGGCATTACAATACTACT

No.2025-166-26-32

TGAGCAGGAATAGTGGGGACGGCCCTAAGCCTCTTAATTCGAGCCGAATTAGGACAGCCAGGATCACTTCTAGGAGATGATCA
AATCTATAATGTTATTGTAACCGCCCATGCATTCGTAATAATCTTCTTTATAGTTATACCCGTAATAATTGGCGGATTTGGAAACTGA
CTAGTGCCATTAATAATTGGTGCACCAGACATAGCTTTTCCACGAATAAATAATATAAGCTTTTGACTCCTTCCCCCCTCTTTTCTT
TTACTTCTAGCTTCAGCTGGAGTTGAAGCCGGAGCCGGTACTGGTTGAACAGTTTATCCTCCATTAGCTGGCAATTTAGCACATG
CTGGGGCATCCGTTGACTTAGCTATTTTCTCTCTCCATTTAGCAGGTATTTCATCAATTTTAGCCTCAATCAACTTTATTACAACTAT
TATTAATATAAAACCCCCTGCAATCTCCCAATATCAAACACCACTATTTGTGTGATCAATTCTAGTAACAACTATCCTCCTTCTATTAT
CCCTCCCAGTACTTGCAGCCGGCATTACAATACTACT

No.2025-166-26-33

TGAGCAGGAATAGTGGGGACAGCCCTAAGCCTCTTAATTCGAGCCGAATTAGGACAGCCAGGATCACTTCTAGGAGATGATCA
AATCTATAATGTTATTGTAACCGCCCATGCATTCGTAATAATCTTCTTTATAGTTATACCCGTAATAATTGGCGGATTTGGAAACTGA
CTAGTGCCATTAATAATTGGTGCACCAGACATAGCTTTTCCACGAATAAATAATATAAGCTTTTGACTCCTTCCCCCCTCTTTTCTT
TTACTTCTAGCTTCAGCTGGAGTTGAAGCCGGAGCCGGTACTGGTTGAACAGTTTATCCTCCATTAGCTGGCAATTTAGCACATG
CTGGGGCATCCGTTGACTTAGCTATTTTCTCTCTCCATTTAGCAGGTATTTCATCAATTTTAGCCTCAATCAACTTTATTACAACTAT
TATTAATATAAAACCCCCTGCAATCTCCCAATATCAAACACCACTATTTGTGTGATCAATTCTAGTAACAACTATCCTCCTTCTATTAT
CCCTCCCAGTACTTGCAGCCGGCATTACAATACTACT

No.2025-166-26-34

GCCCTCAGCCTTCTAATTCGAGCCGAGTTAGGCCAGCCCGGATCACTCCTAGGGGATGATCAGGTCTATAATGTTATCGTAACCG
CCCATGCATTTGTAATAATCTTCTTCATGGTTATACCCGTAATAATTGGGGGATTTGGAAACTGATTAGTACCCTTAATAATTGGTG
CACCAGACATGGCCTTCCCGCGAATAAATAACATAAGCTTTTGACTCCTTCCCCCTTCTTTTCTCTTACTCCTAGCTTCAGCTGGG
GTTGAAGCTGGAGCTGGCACTGGTTGAACAGTTTATCCCCCCTTAGCTGGCAACTTAGCACATGCTGGGGCATCTGTTGACTTG
GCCATTTTCTCGCTTCATTTAGCAGGTATCTCATCAATTTTAGCTTCAATTAACTTTATTACAACTATCATTAATATAAAACCACCAG
CCATCTCTCAATATCAAACACCATTATTTGTATGATCAATCCTAGTAACAACCATCCTCCTCCTCTTATCCCTCCCAGTACTCGCAGC
CGGCATCACAATATTATTAACTGATCGAAACCTAAACACAACATTCT

No.2025-166-26-35

GCCCTCAGCCTTCTAATTCGAGCCGAGTTAGGCCAGCCCGGATCACTCCTAGGGGATGATCAGGTCTATAATGTTATCGTAACCG
CCCATGCATTTGTAATAATCTTCTTCATGGTTATACCCGTAATAATTGGGGGATTTGGAAACTGATTAGTACCCTTAATAATTGGTG
CACCAGACATGGCCTTCCCGCGAATAAATAACATAAGCTTTTGACTCCTTCCCCCTTCTTTTCTCTTACTCCTAGCTTCAGCTGGG
GTTGAAGCTGGAGCTGGCACTGGTTGAACAGTTTATCCCCCCTTAGCTGGCAACTTAGCACATGCTGGGGCATCTGTTGACTTG
GCCATTTTCTCGCTTCATTTAGCAGGTATCTCATCAATTTTAGCTTCAATTAACTTTATTACAACTATCATTAATATAAAACCACCAG
CCATCTCTCAATATCAAACACCATTATTTGTATGATCAATCCTAGTAACAACCATCCTCCTCCTCTTATCCCTCCCAGTACTCGCAGC
CGGCATCACAATAT

No.2025-166-26-36

CCTAAGTCTTCTAATTCGAGCTGAGCTTGGACAACCTGGATCACTTTTAGGGGATGATCAGATTTATAATGTAATCGTAACCGCCC
ACGCTTTTGTAATAATCTTTTTTATGGTTATGCCAATCATAATTGGTGGTTTCGGAAATTGACTAGTTCCTTTAATAATTGGTGCAC
CAGATATAGCCTTCCCACGAATAAATAACATAAGTTTCTGACTTCTTCCACCATCATTTCTTCTTCTCCTCGCCTCTGCTGGAGTAG
AAGCTGGAGCAGGTACTGGTTGAACAGTTTATCCTCCATTAGCTAGTAACCTAGCACATGCTGGACCATCTGTTGATTTAGCTAT
TTTCTCTCTTCACTTAGCCGGTGTGTCATCTATTCTAGCTTCAATTAATTTTATTACAACTATTATCAATATAAAACCACCAGCCATTT
CCCAATATCAAACACCATTATTTGTTTGATCTATTCTTGTAACCACTATTCTTCTCCTCCTATCACTTCCAGTTCTTGCAGCAGGGAT
TACAATATTACTTACAGATCG

No.2025-166-26-37

TGCATGAGCAGGTATAGTTGGAACAGCCCTAAGTCTTCTAATTCGAGCTGAGCTTGGACAACCTGGATCACTTTTAGGGGATGA
TCAGATTTATAATGTAATCGTAACCGCCCACGCTTTTGTAATAATCTTTTTTATGGTTATGCCAATCATAATTGGTGGTTTCGGAAAT
TGACTAGTTCCTTTAATAATTGGTGCACCAGATATAGCCTTCCCACGAATAAATAACATAAGTTTCTGACTTCTTCCACCATCATTT
CTTCTTCTCCTCGCCTCTGCTGGAGTAGAAGCTGGAGCAGGTACTGGTTGAACAGTTTATCCTCCATTAGCTAGTAACCTAGCAC
ATGCTGGACCATCTGTTGATTTAGCTATTTTCTCTCTTCACTTAGCCGGTGTGTCATCTATTCTAGCTTCAATTAATTTTATTACAACT
ATTATCAATATAAAACCACCAGCCATTTCCCAATATCAAACACCATTATTTGTTTGATCTATTCTTGTAACCACTATTCTTCTCCTCCT
ATCACTTCCAGTTCTTGCAGCAGGGATTACAATATTACTTACAGATCG

No.2025-166-26-38

TATATTTAATCTTTGGTGCATGAGCAGGAATAGTGGGGACGGCCCTAAGCCTCTTAATTCGAGCCGAATTAGGACAGCCAGGAT
CACTTCTAGGAGATGATCAAATCTATAATGTTATTGTAACCGCCCATGCATTCGTAATAATCTTCTTTATAGTTATACCCGTAATAATT
GGCGGATTTGGAAACTGACTAGTGCCATTAATAATTGGTGCACCAGACATAGCTTTTCCACGAATAAATAATATAAGCTTTTGACT
CCTTCCCCCCTCTTTTCTTTTACTTCTAGCTTCAGCTGGAGTTGAAGCCGGAGCCGGTACTGGTTGAACAGTTTATCCTCCATTAG
CTGGCAATTTAGCACATGCTGGGGCATCCGTTGACTTAGCTATTTTCTCTCTCCATTTAGCAGGTATTTCATCAATTTTAGCCTCAA
TCAACTTTATTACAACTATTATTAATATAAAACCCCCTGCAATCTCCCAATATCAAACACCACTATTTGTGTGATCAATTCTAGTAAC
AACTATCCTCCTTCTATTATCCCTCCCAGTACTTGCAGCCGGCATTACAATACTACT

No.2025-166-26-39

TGAGCAGGTATAGTTGGAACAGCCCTAAGTCTTCTAATTCGAGCTGAGCTTGGACAACCTGGATCACTTTTAGGGGATGATCAG
ATTTATAATGTAATCGTAACCGCCCACGCTTTTGTAATAATCTTTTTTATGGTTATGCCAATCATAATTGGTGGTTTCGGAAATTGAC
TAGTTCCTTTAATAATTGGTGCACCAGATATAGCCTTCCCACGAATAAATAACATAAGTTTCTGACTTCTTCCACCATCATTTCTTCT
TCTCCTCGCCTCTGCTGGAGTAGAAGCTGGAGCAGGTACTGGTTGAACAGTTTATCCTCCATTAGCTAGTAACCTAGCACATGCT
GGACCATCTGTTGATTTAGCTATTTTCTCTCTTCACTTAGCCGGTGTGTCATCTATTCTAGCTTCAATTAATTTTATTACAACTATTAT
CAATATAAAACCACCAGCCATTTCCCAATATCAAACACCATTATTTGTTTGATCTATTCTTGTAACCACTATTCTTCTCCTCCTATCAC
TTCCAGTTCTTGCAGCAGGGATTACAATATTACTTACAGATCG

No.2025-166-26-40

CAGCCCTAAGTCTTCTAATTCGAGCTGAGCTTGGACAACCTGGATCACTTTTAGGAGATGATCAGATTTATAATGTAATCGTAACC
GCCCACGCTTTTGTAATAATCTTTTTTATGGTTATGCCAATCATAATTGGTGGTTTCGGAAATTGACTAGTTCCTTTAATAATTGGT
GCACCAGATATAGCCTTCCCACGAATAAATAACATAAGTTTCTGACTTCTTCCACCATCATTTCTTCTTCTCCTCGCCTCTGCTGGA
GTAGAAGCTGGAGCAGGTACTGGTTGAACAGTTTATCCTCCATTAGCTAGTAACCTAGCACATGCTGGACCATCTGTTGATTTAG
CTATTTTCTCTCTTCACTTAGCCGGTGTGTCATCTATTCTAGCTTCAATTAATTTTATTACAACTATTATCAATATAAAACCACCAGCC
ATTTCCCAATATCAAACACCATTATTTGTTTGATCTATTCTTGTAACCACTATTCTTCTCCTCCTATCACTTCCAGTTCTTGCAGCAG
GGATTACAATATTACTTACAGATCG

No.2025-166-26-41

TGAGCAGGTATAGTTGGAACAGCCCTAAGTCTTCTAATTCGAGCTGAGCTTGGACAACCTGGATCACTTTTAGGGGATGATCAG
ATTTATAATGTAATCGTAACCGCCCACGCTTTTGTAATAATCTTTTTTATGGTTATGCCAATCATAATTGGTGGTTTCGGAAATTGAC
TAGTTCCTTTAATAATTGGTGCACCAGATATAGCCTTCCCACGAATAAATAACATAAGTTTCTGACTTCTTCCACCATCATTTCTTCT
TCTCCTCGCCTCTGCTGGAGTAGAAGCTGGAGCAGGTACTGGTTGAACAGTTTATCCTCCATTAGCTAGTAACCTAGCACATGCT
GGACCATCTGTTGATTTAGCTATTTTCTCTCTTCACTTAGCCGGTGTGTCATCTATTCTAGCTTCAATTAATTTTATTACAACTATTAT
CAATATAAAACCACCAGCCATTTCCCAATATCAAACACCATTATTTGTTTGATCTATTCTTGTAACCACTATTCTTCTCCTCCTATCAC
TTCCAGTTCTTGCAGCAGGGATTACAATATTACTTACAGATCG

No.2025-166-26-42

AGCCCTAAGTCTTCTAATTCGAGCTGAGCTTGGACAACCTGGATCACTTTTAGGGGATGATCAGATTTATAATGTAATCGTAACC
GCCCACGCTTTTGTAATAATCTTTTTTATGGTTATGCCAATCATAATTGGTGGTTTCGGAAATTGACTAGTTCCTTTAATAATTGGT
GCACCAGATATAGCCTTCCCACGAATAAATAACATAAGTTTCTGACTTCTTCCACCATCATTTCTTCTTCTCCTCGCCTCTGCTGGA
GTAGAAGCTGGAGCAGGTACTGGTTGAACAGTTTATCCTCCATTAGCTAGTAACCTAGCACATGCTGGACCATCTGTTGATTTAG
CTATTTTCTCTCTTCACTTAGCCGGTGTGTCATCTATTCTAGCTTCAATTAATTTTATTACAACTATTATCAATATAAAACCACCAGCC
ATTTCCCAATATCAAACACCATTATTTGTTTGATCTATTCTTGTAACCACTATTCTTCTCCTCCTATCACTTCCAGTTCTTGCAGCAG
GGATTACAATATTACTTACAGATCG

No.2025-166-26-43

CAGCCCTAAGTCTTCTAATTCGAGCTGAGCTTGGACAACCTGGATCACTTTTAGGGGATGATCAGATTTATAATGTAATCGTAACC
GCCCACGCTTTTGTAATAATCTTTTTTATGGTTATGCCAATCATAATTGGTGGTTTCGGAAATTGACTAGTTCCTTTAATAATTGGT
GCACCAGATATAGCCTTCCCACGAATAAATAACATAAGTTTCTGACTTCTTCCACCATCATTTCTTCTTCTCCTCGCCTCTGCTGGA
GTAGAAGCTGGAGCAGGTACTGGTTGAACAGTTTATCCTCCATTAGCTAGTAACCTAGCACATGCTGGACCATCTGTTGATTTAG
CTATTTTCTCTCTTCACTTAGCCGGTGTGTCATCTATTCTAGCTTCAATTAATTTTATTACAACTATTATCAATATAAAACCACCAGCC
ATTTCCCAATATCAAACACCATTATTTGTTTGATCTATTCTTGTAACCACTATTCTTCTCCTCCTATCACTTCCAGTTCTTGCAGCAG
GGATTACAATATTACTTACAGATCG

No.2025-166-26-44

TGCATGAGCAGGTATAGTTGGAACAGCCCTAAGTCTTCTAATTCGAGCTGAGCTTGGACAACCTGGATCACTTTTAGGGGATGA
TCAGATTTATAATGTAATCGTAACCGCCCACGCTTTTGTAATAATCTTTTTTATGGTTATGCCAATCATAATTGGTGGTTTCGGAAAT
TGACTAGTTCCTTTAATAATTGGTGCACCAGATATAGCCTTCCCACGAATAAATAACATAAGTTTCTGACTTCTTCCACCATCATTT
CTTCTTCTCCTCGCCTCTGCTGGAGTAGAAGCTGGAGCAGGTACTGGTTGAACAGTTTATCCTCCATTAGCTAGTAACCTAGCAC
ATGCTGGACCATCTGTTGATTTAGCTATTTTCTCTCTTCACTTAGCCGGTGTGTCATCTATTCTAGCTTCAATTAATTTTATTACAACT
ATTATCAATATAAAACCACCAGCCATTTCCCAATATCAAACACCATTATTTGTTTGATCTATTCTTGTAACCACTATTCTTCTCCTCCT
ATCACTTCCAGTTCTTGCAGCAGGGATTACAATATTACTTACAGATCG

No.2025-166-26-45

CAGCCCTCAGCCTTCTAATTCGAGCCGAGTTAGGCCAGCCCGGATCACTCCTAGGGGATGATCAGGTCTATAATGTTATCGTAAC
CGCCCATGCATTTGTAATAATCTTCTTCATGGTTATACCCGTAATAATTGGGGGATTTGGAAACTGATTAGTACCCTTAATAATTGG
TGCACCAGACATGGCCTTCCCGCGAATAAATAACATAAGCTTTTGACTCCTTCCCCCTTCTTTTCTCTTACTCCTAGCTTCAGCTG
GGGTTGAAGCTGGAGCTGGCACTGGTTGAACAGTTTATCCCCCCTTAGCTGGCAACTTAGCACATGCTGGGGCATCTGTTGAC
TTGGCCATTTTCTCGCTTCATTTAGCAGGTATCTCATCAATTTTAGCTTCAATTAACTTTATTACAACTATCATTAATATAAAACCACC
AGCCATCTCTCAATATCAAACACCATTATTTGTATGATCAATCCTAGTAACAACCATCCTCCTCCTCTTATCCCTCCCAGTACTCGCA
GCCGGCATCACAATATTAT

No.2025-166-26-46

TGAGCAGGAATAGTGGGGACGGCCCTAAGCCTCTTAATTCGAGCCGAATTAGGACAGCCAGGATCACTTCTAGGAGATGATCA
AATCTATAATGTTATTGTAACCGCCCATGCATTCGTAATAATCTTCTTTATAGTTATACCCGTAATAATTGGCGGATTTGGAAACTGA
CTAGTGCCATTAATAATTGGTGCACCAGACATAGCTTTTCCACGAATAAATAATATAAGCTTTTGACTCCTTCCCCCCTCTTTTCTT
TTACTTCTAGCTTCAGCTGGAGTTGAAGCCGGAGCCGGTACTGGTTGAACAGTTTATCCTCCATTAGCTGGCAATTTAGCACATG
CTGGGGCATCCGTTGACTTAGCTATTTTCTCTCTCCATTTAGCAGGTATTTCATCAATTTTAGCCTCAATCAACTTTATTACAACTAT
TATTAATATAAAACCCCCTGCAATCTCCCAATATCAAACACCACTATTTGTGTGATCAATTCTAGTAACAACTATCCTCCTTCTATTAT
CCCTCCCAGTACTTGCAGCCGGCATTACAATACTACT

No.2025-166-26-47

GCCCTAAGCCTTTTAATTCGTGCCGAACTGGGTCAGCCTGGTTCCCTCCTAGGGGATGATCAGATTTATAATGTTATTGTAACCGC
CCATGCATTTGTAATAATTTTCTTTATGGTTATGCCCGTAATAATTGGAGGCTTTGGAAATTGACTAGTGCCTTTAATGATCGGAGC
ACCAGACATAGCCTTCCCCCGAATAAATAACATAAGTTTCTGGCTCCTACCCCCTTCTTTCCTTTTACTCTTAGCCTCAGCCGGAG
TTGAGTCAGGAGCCGGCACTGGTTGAACAGTCTACCCTCCCCTAGCTGGCAACTTAGCACACGCCGGAGCATCTGTTGATCTAG
CCATTTTCTCCCTCCACCTGGCTGGTATCTCGTCCATCCTAGCTTCCATTAACTTCATTACAACCATCATCAACATAAAACCCCCAG
CAATCTCCCAATACCAAACACCCCTGTTTGTCTGGTCCATTCTAGTGACAACCATCCTCCTTCTTTTAGCACTCCCAGTGCTCGCC
GCTGGCATTACAATACTACTTACGGACCGAAACCTAAACACAACATTCTTT

No.2025-166-26-48

TGAGCAGGTATAGTTGGAACAGCCCTAAGTCTTCTAATTCGAGCTGAGCTTGGACAACCTGGATCACTTTTAGGGGATGATCAG
ATTTATAATGTAATCGTAACCGCCCACGCTTTTGTAATAATCTTTTTTATGGTTATGCCAATCATAATTGGTGGTTTCGGAAATTGAC
TAGTTCCTTTAATAATTGGTGCACCAGATATAGCCTTCCCACGAATAAATAACATAAGTTTCTGACTTCTTCCACCATCATTTCTTCT
TCTCCTCGCCTCTGCTGGAGTAGAAGCTGGAGCAGGTACTGGTTGAACAGTTTATCCTCCATTAGCTAGTAACCTAGCACATGCT
GGACCATCTGTTGATTTAGCTATTTTCTCTCTTCACTTAGCCGGTGTGTCATCTATTCTAGCTTCAATTAATTTTATTACAACTATTAT
CAATATAAAACCACCAGCCATTTCCCAATATCAAACACCATTATTTGTTTGATCTATTCTTGTAACCACTATTCTTCTCCTCCTATCAC
TTCCAGTTCTTGCAGCAGGGATTACAATATTACTTACAGATCG

No.2025-166-26-49

TGAGCAGGTATAGTTGGAACAGCCCTAAGTCTTCTAATTCGAGCTGAGCTTGGACAACCTGGATCACTTTTAGGGGATGATCAG
ATTTATAATGTAATCGTAACCGCCCACGCTTTTGTAATAATCTTTTTTATGGTTATGCCAATCATAATTGGTGGTTTCGGAAATTGAC
TAGTTCCTTTAATAATTGGTGCACCAGATATAGCCTTCCCACGAATAAATAACATAAGTTTCTGACTTCTTCCACCATCATTTCTTCT
TCTCCTCGCCTCTGCTGGAGTAGAAGCTGGAGCAGGTACTGGTTGAACAGTTTATCCTCCATTAGCTAGTAACCTAGCACATGCT
GGACCATCTGTTGATTTAGCTATTTTCTCTCTTCACTTAGCCGGTGTGTCATCTATTCTAGCTTCAATTAATTTTATTACAACTATTAT
CAATATAAAACCACCAGCCATTTCCCAATATCAAACACCATTATTTGTTTGATCTATTCTTGTAACCACTATTCTTCTCCTCCTATCAC
TTCCAGTTCTTGCAGCAGGGATTACAATATTACTTACAGATCG

No.2025-166-26-50

ATAGTGGGGACGGCCCTAAGCCTCTTAATTCGAGCCGAATTAGGACAGCCAGGATCACTTCTAGGAGATGATCAAATCTATAATG
TTATTGTAACCGCCCATGCATTCGTAATAATCTTCTTTATAGTTATACCCGTAATAATTGGCGGATTTGGAAACTGACTAGTGCCATT
AATAATTGGTGCACCAGACATAGCTTTTCCACGAATAAATAATATAAGCTTTTGACTCCTTCCCCCCTCTTTTCTTTTACTTCTAGCT
TCAGCTGGAGTTGAAGCCGGAGCCGGTACTGGTTGAACAGTTTATCCTCCATTAGCTGGCAATTTAGCACATGCTGGGGCATCC
GTTGACTTAGCTATTTTCTCTCTCCATTTAGCAGGTATTTCATCAATTTTAGCCTCAATCAACTTTATTACAACTATTATTAATATAAA
ACCCCCTGCAATCTCCCAATATCAAACACCACTATTTGTGTGATCAATTCTAGTAACAACTATCCTCCTTCTATTATCCCTCCCAGTA
CTTGCAGCCGGCATTACAATACTACT

No.2025-166-26-51

CCCTAAGCCTTTTAATTCGTGCCGAACTGGGTCAGCCTGGTTCCCTCCTAGGGGATGATCAGATTTATAATGTTATTGTAACCGCC
CATGCATTTGTAATAATTTTCTTTATGGTTATGCCCGTAATAATTGGAGGCTTTGGAAATTGACTAGTGCCTTTAATGATCGGAGCA
CCAGACATAGCCTTCCCCCGAATAAATAACATAAGTTTCTGGCTCCTACCCCCTTCTTTCCTTTTACTCTTAGCCTCAGCCGGAGTT
GAGTCAGGAGCCGGCACTGGTTGAACAGTCTACCCTCCCCTAGCTGGCAACTTAGCACACGCCGGAGCATCTGTTGATCTAGC
CATTTTCTCCCTCCACCTGGCTGGTATCTCGTCCATCCTAGCTTCCATTAACTTCATTACAACCATCATCAACATAAAACCCCCAGC
AATCTCCCAATACCAAACACCCCTGTTTGTCTGGTCCATTCTAGTGACAACCATCCTCCTTCTTTTAGCACTCCCAGTGCTCGCCG
CTGGCATTACAATACTACTTACGGACCGGAACCTAAACACAACATTC

No.2025-166-26-52

GAGCAGGTATAGTTGGACAGCCCTAAGTCTTCTAATTCGAGCTGAGCTTGGACAACCTGGATCACTTTTAGGGGATGATCAGAT
TTATAATGTAATCGTAACCGCCCACGCTTTTGTAATAATCTTTTTTATGGTTATGCCAATCATAATTGGTGGTTTCGGAAATTGACTA
GTTCCTTTAATAATTGGTGCACCAGATATAGCCTTCCCACGAATAAATAACATAAGTTTCTGACTTCTTCCACCATCATTTCTTCTTC
TCCTCGCCTCTGCTGGAGTAGAAGCTGGAGCAGGTACTGGTTGAACAGTTTATCCTCCATTAGCTAGTAACCTAGCACATGCTG
GACCATCTGTTGATTTAGCTATTTTCTCTCTTCACTTAGCCGGTGTGTCATCTATTCTAGCTTCAATTAATTTTATTACAACTATTATC
AATATAAAACCACCAGCCATTTCCCAATATCAAACACCATTATTTGTTTGATCTATTCTTGTAACCACTATTCTTCTCCTCCTATCACT
TCCAGTTCTTGCAGCAGGGATTACAATATTACTTACAGATCG

No.2025-166-26-53

TGAGCAGGTATAGTTGGACAGCCCTAAGTCTTCTAATTCGAGCTGAGCTTGGACAACCTGGATCACTTTTAGGGGATGATCAGA
TTTATAATGTAATCGTAACCGCCCACGCTTTTGTAATAATCTTTTTTATGGTTATGCCAATCATAATTGGTGGTTTCGGAAATTGACT
AGTTCCTTTAATAATTGGTGCACCAGATATAGCCTTCCCACGAATAAATAACATAAGTTTCTGACTTCTTCCACCATCATTTCTTCTT
CTCCTCGCCTCTGCTGGAGTAGAAGCTGGAGCAGGTACTGGTTGAACAGTTTATCCTCCATTAGCTAGTAACCTAGCACATGCTG
GACCATCTGTTGATTTAGCTATTTTCTCTCTTCACTTAGCCGGTGTGTCATCTATTCTAGCTTCAATTAATTTTATTACAACTATTATC
AATATAAAACCACCAGCCATTTCCCAATATCAAACACCATTATTTGTTTGATCTATTCTTGTAACCACTATTCTTCTCCTCCTATCACT
TCCAGTTCTTGCAGCAGGGATTACAATATTACTTACAGATCGTAACCTTAATACTACATTCTTTGA

No.2025-166-26-54

TGAGCAGGTATAGTTGGAACAGCCCTAAGTCTTCTAATTCGAGCTGAGCTTGGACAACCTGGATCACTTTTAGGGGATGATCAG
ATTTATAATGTAATCGTAACCGCCCACGCTTTTGTAATAATCTTTTTTATGGTTATGCCAATCATAATTGGTGGTTTCGGAAATTGAC
TAGTTCCTTTAATAATTGGTGCACCAGATATAGCCTTCCCACGAATAAATAACATAAGTTTCTGACTTCTTCCACCATCATTTCTTCT
TCTCCTCGCCTCTGCTGGAGTAGAAGCTGGAGCAGGTACTGGTTGAACAGTTTATCCTCCATTAGCTAGTAACCTAGCACATGCT
GGACCATCTGTTGATTTAGCTATTTTCTCTCTTCACTTAGCCGGTGTGTCATCTATTCTAGCTTCAATTAATTTTATTACAACTATTAT
CAATATAAAACCACCAGCCATTTCCCAATATCAAACACCATTATTTGTTTGATCTATTCTTGTAACCACTATTCTTCTCCTCCTATCAC
TTCCAGTTCTTGCAGCAGGGATTACAATAT

No.2025-166-26-55

TGAGCAGGTATAGTTGGAACAGCCCTAAGTCTTCTAATTCGAGCTGAGCTTGGACAACCTGGATCACTTTTAGGGGATGATCAG
ATTTATAATGTAATCGTAACCGCCCACGCTTTTGTAATAATCTTTTTTATGGTTATGCCAATCATAATTGGTGGTTTCGGAAATTGAC
TAGTTCCTTTAATAATTGGTGCACCAGATATAGCCTTCCCACGAATAAATAACATAAGTTTCTGACTTCTTCCACCATCATTTCTTCT
TCTCCTCGCCTCTGCTGGAGTAGAAGCTGGAGCAGGTACTGGTTGAACAGTTTATCCTCCATTAGCTAGTAACCTAGCACATGCT
GGACCATCTGTTGATTTAGCTATTTTCTCTCTTCACTTAGCCGGTGTGTCATCTATTCTAGCTTCAATTAATTTTATTACAACTATTAT
CAATATAAAACCACCAGCCATTTCCCAATATCAAACACCATTATTTGTTTGATCTATTCTTGTAACCACTATTCTTCTCCTCCTATCAC
TTCCAGTTCTTGCAGCAGGGATTACAATATTACTTACAGATCG

No.2025-166-26-56

TGAGCAGGTATAGTTGGAACAGCCCTAAGTCTTCTAATTCGAGCTGAGCTTGGACAACCTGGATCACTTTTAGGGGATGATCAG
ATTTATAATGTAATCGTAACCGCCCACGCTTTTGTAATAATCTTTTTTATGGTTATGCCAATCATAATTGGTGGTTTTGGAAATTGAC
TAGTTCCTTTAATAATTGGTGCACCAGATATAGCCTTCCCACGAATAAATAACATAAGTTTCTGACTTCTTCCACCATCATTTCTTCT
TCTCCTCGCCTCTGCTGGAGTAGAAGCTGGAGCAGGTACTGGTTGAACAGTTTATCCTCCATTAGCTAGTAACCTAGCACATGCT
GGACCATCTGTTGATTTAGCTATTTTCTCTCTTCACTTAGCCGGTGTGTCATCTATTCTAGCTTCAATTAATTTTATTACAACTATTAT
CAATATAAAACCACCAGCCATTTCCCAATATCAAACACCATTATTTGTTTGATCTATTCTTGTAACCACTATTCTTCTCCTCCTATCAC
TTCCAGTTCTTGCAGCAGGGATTACAATATTACTTACAGATCG

No.2025-166-26-57

TGAGCAGGTATAGTTGGAACAGCCCTAAGTCTTCTAATTCGAGCTGAGCTTGGACAACCTGGATCACTTTTAGGGGATGATCAG
ATTTATAATGTAATCGTAACCGCCCACGCTTTTGTAATAATCTTTTTTATGGTTATGCCAATCATAATTGGTGGTTTCGGAAATTGAC
TAGTTCCTTTAATAATTGGTGCACCAGATATAGCCTTCCCACGAATAAATAACATAAGTTTCTGACTTCTTCCACCATCATTTCTTCT
TCTCCTCGCCTCTGCTGGAGTAGAAGCTGGAGCAGGTACTGGTTGAACAGTTTATCCTCCATTAGCTAGTAACCTAGCACATGCT
GGACCATCTGTTGATTTAGCTATTTTCTCTCTTCACTTAGCCGGTGTGTCATCTATTCTAGCTTCAATTAATTTTATTACAACTATTAT
CAATATAAAACCACCAGCCATTTCCCAATATCAAACACCATTATTTGTTTGATCTATTCTTGTAACCACTATTCTTCTCCTCCTATCAC
TTCCAGTTCTTGCAGCAGGGATTACAATATTACTTACAGATC

No.2025-166-26-58

TGAGCAGGTATAGTTGGAACAGCCCTAAGTCTTCTAATTCGAGCTGAGCTTGGACAACCTGGATCACTTTTAGGGGATGATCAG
ATTTATAATGTAATCGTAACCGCCCACGCTTTTGTAATAATCTTTTTTATGGTTATGCCAATCATAATTGGTGGTTTCGGAAATTGAC
TAGTTCCTTTAATAATTGGTGCACCAGATATAGCCTTCCCACGAATAAATAACATAAGTTTCTGACTTCTTCCACCATCATTTCTTCT
TCTCCTCGCCTCTGCTGGAGTAGAAGCTGGAGCAGGTACTGGTTGAACAGTTTATCCTCCATTAGCTAGTAACCTAGCACATGCT
GGACCATCTGTTGATTTAGCTATTTTCTCTCTTCACTTAGCCGGTGTGTCATCTATTCTAGCTTCAATTAATTTTATTACAACTATTAT
CAATATAAAACCACCAGCCATTTCCCAATATCAAACACCATTATTTGTTTGATCTATTCTTGTAACCACTATTCTTCTCCTCCTATCAC
TTCCAGTTCTTGCAGCAGGGATTACAATATTACTTACAGATCG

No.2025-166-26-59

TGAGCAGGTATAGTTGGAACAGCCCTAAGTCTTCTAATTCGAGCTGAGCTTGGACAACCTGGATCACTTTTAGGGGATGATCAG
ATTTATAATGTAATCGTAACCGCCCACGCTTTTGTAATAATCTTTTTTATGGTTATGCCAATCATAATTGGTGGTTTCGGAAATTGAC
TAGTTCCTTTAATAATTGGTGCACCAGATATAGCCTTCCCACGAATAAATAACATAAGTTTCTGACTTCTTCCACCATCATTTCTTCT
TCTCCTCGCCTCTGCTGGAGTAGAAGCTGGAGCAGGTACTGGTTGAACAGTTTATCCTCCATTAGCTAGTAACCTAGCACATGCT
GGACCATCTGTTGATTTAGCTATTTTCTCTCTTCACTTAGCCGGTGTGTCATCTATTCTAGCTTCAATTAATTTTATTACAACTATTAT
CAATATAAAACCACCAGCCATTTCCCAATATCAAACACCATTATTTGTTTGATCTATTCTTGTAACCACTATTCTTCTCCTCCTATCAC
TTCCAGTTCTTGCAGCAGGGATTACAATATTACTTACAGATCG

No.2025-166-26-60

CAGCCCTAAGCCTTTTAATTCGTGCCGAACTGGGTCAGCCTGGTTCCCTCCTAGGGGATGATCAGATTTATAATGTTATTGTAACC
GCCCATGCATTTGTAATAATTTTCTTTATGGTTATGCCCGTAATAATTGGAGGCTTTGGAAATTGACTAGTGCCTTTAATGATCGGA
GCACCAGACATAGCCTTCCCCCGAATAAATAACATAAGTTTCTGGCTCCTACCCCCTTCTTTCCTTTTACTCTTAGCCTCAGCCGG
AGTTGAGTCAGGAGCCGGCACTGGTTGAACAGTCTACCCTCCCCTAGCTGGCAACTTAGCACACGCCGGAGCATCTGTTGATCT
AGCCATTTTCTCCCTCCACCTGGCTGGTATCTCGTCCATCCTAGCTTCCATTAACTTCATTACAACCATCATCAACATAAAACCCCC
AGCAATCTCCCAATACCAAACACCCCTGTTTGTCTGGTCCATTCTAGTGACAACCATCCTCCTTCTTTTAGCACTCCCAGTGCTCG
CCGCTGGCATTACAATACTACTTACGGACCGAAACCTAAACACAACATTCTTT

No.2025-166-26-61

ACAGCCCTCAGCCTTCTAATTCGAGCCGAGTTAGGCCAGCCCGGATCACTCCTAGGGGATGATCAGGTCTATAATGTTATCGTAA
CCGCCCATGCATTTGTAATAATCTTCTTCATGGTTATACCCGTAATAATTGGGGGATTTGGAAACTGATTAGTACCCTTAATAATTG
GTGCACCAGACATGGCCTTCCCGCGAATAAATAACATAAGCTTTTGACTCCTTCCCCCTTCTTTTCTCTTACTCCTAGCTTCAGCT
GGGGTTGAAGCTGGAGCTGGCACTGGTTGAACAGTTTATCCCCCCTTAGCTGGCAACTTAGCACATGCTGGGGCATCTGTTGA
CTTGGCCATTTTCTCGCTTCATTTAGCAGGTATCTCATCAATTTTAGCTTCAATTAACTTTATTACAACTATCATTAATATAAAACCAC
CAGCCATCTCTCAATATCAAACACCATTATTTGTATGATCAATCCTAGTAACAACCATCCTCCTCCTCTTATCCCTCCCAGTACTCGC
AGCCGGCATCACAATATTATTAACTGATCGAAACCTAAACACAACATTCT

No.2025-166-26-62

TGAGCAGGTATAGTTGGAACAGCCCTAAGTCTTCTAATTCGAGCTGAGCTTGGACAACCTGGATCACTTTTAGGGGATGATCAG
ATTTATAATGTAATCGTAACCGCCCACGCTTTTGTAATAATCTTTTTTATGGTTATGCCAATCATAATTGGTGGTTTCGGAAATTGAC
TAGTTCCTTTAATAATTGGTGCACCAGATATAGCCTTCCCACGAATAAATAACATAAGTTTCTGACTTCTTCCACCATCATTTCTTCT
TCTCCTCGCCTCTGCTGGAGTAGAAGCTGGAGCAGGTACTGGTTGAACAGTTTATCCTCCATTAGCTAGTAACCTAGCACATGCT
GGACCATCTGTTGATTTAGCTATTTTCTCTCTTCACTTAGCCGGTGTGTCATCTATTCTAGCTTCAATTAATTTTATTACAACTATTAT
CAATATAAAACCACCAGCCATTTCCCAATATCAAACACCATTATTTGTTTGATCTATTCTTGTAACCACTATTCTTCTCCTCCTATCAC
TTCCAGTTCTTGCAGCAGGGATTACAATATTACTTACAGATC

No.2025-166-26-63

TGAGCAGGAATAGTGGGGACGGCCCTAAGCCTCTTAATTCGAGCCGAATTAGGACAGCCAGGATCACTTCTAGGAGATGATCA
AATCTATAATGTTATTGTAACCGCCCATGCATTCGTAATAATCTTCTTTATAGTTATACCCGTAATAATTGGCGGATTTGGAAACTGA
CTAGTGCCATTAATAATTGGTGCACCAGACATAGCTTTTCCACGAATAAATAATATAAGCTTTTGACTCCTTCCCCCCTCTTTTCTT
TTACTTCTAGCTTCAGCTGGAGTTGAAGCCGGAGCCGGTACTGGTTGAACAGTTTATCCTCCATTAGCTGGCAATTTAGCACATG
CTGGGGCATCCGTTGACTTAGCTATTTTCTCTCTCCATTTAGCAGGTATTTCATCAATTTTAGCCTCAATCAACTTTATTACAACTAT
TATTAATATAAAACCCCCTGCAATCTCCCAATATCAAACACCACTATTTGTGTGATCAATTCTAGTAACAACTATCCTCCTTCTATTAT
CCCTCCCAGTACTTGCAGCCGGCATTACAATACTACT

No.2025-166-26-64

TGAGCAGGAATAGTGGGAACAGCCCTCAGCCTTCTAATTCGAGCCGAGTTAGGCCAGCCCGGATCACTCCTAGGGGATGATCA
GGTCTATAATGTTATCGTAACCGCCCATGCATTTGTAATAATCTTCTTCATGGTTATACCCGTAATAATTGGGGGATTTGGAAACTG
ATTAGTACCCTTAATAATTGGTGCACCAGACATGGCCTTCCCGCGAATAAATAACATAAGCTTTTGACTCCTTCCCCCTTCTTTTCT
CTTACTCCTAGCTTCAGCTGGGGTTGAAGCTGGAGCTGGCACTGGTTGAACAGTTTATCCCCCCTTAGCTGGCAACTTAGCACA
TGCTGGGGCATCTGTTGACTTGGCCATTTTCTCGCTTCATTTAGCAGGTATCTCATCAATTTTAGCTTCAATTAACTTTATTACAAC
TATCATTAATATAAAACCACCAGCCATCTCTCAATATCAAACACCATTATTTGTATGATCAATCCTAGTAACAACCATCCTCCTCCT-C
TTATCCCTCCCAGT-ACTCGCAGCCGGCATCACAATATTA

Shark fin: 16S rRNA gene - Small-sized samples (n=64)

No.2025-166-26-1

AGAGGTCCCGCCTGCCCTGTGACAATGTTTTAACGGCCGCGGTATTTTGACCGTGCAAAGGTAGCGTAATCACTTGTCTTTTAAA
TGAAGACCCGTATGAAAGGCATCACGAGAGTTCAACTGTCTCTATTTTCTAATCAATGAAATTGATCTACTCGTGCAGAAGCGAG
TATAACTACATTAGACGAGAAGACCCTATGGAGCTTCAAACACATGAATTAAATATGTAAACTAACTACTCCCCGGACATAAATAA
AATAATATTTTTAATTTAACTGTTTTTGGTTGGGGTGACCAAGGGGAAAAATAAATCCCCCTTATCGATTGAGTACTCAAGTACTT
AAAAATCAGAATTACAATTCTGATTAATAAAATATTTATCGAAAAATGACCCAGGATTTCCTGATCAATGAACCAAGTTACCCTAG
GGATAACAGCGCAATCCTTTCTTAGAGTCCCTATCGCCGAAAGGGTTTACGACCTCGATGTTGGATCAGGACATCCTAATGATGC
AACCGTTATTAAGGGTTCGTT

No.2025-166-26-2

CCGCCTGCCCTGTGACAATGTTTTAACGGCCGCGGTATTTTGACCGTGCAAAGGTAGCGTAATCACTTGTCTTTTAAATGAAGAC
CCGTATGAAAGGCATCACGAGAGTTCAACTGTCTCTATTTTCTAATCAATGAAATTGATCTACTCGTGCAGAAGCGAGTATAACTA
CATTAGACGAGAAGACCCTATGGAGCTTCAAACACATGAATTAAATATGTAAACTAACTACTCCCCGGACATAAATAAAATAATAT
TTTTAATTTAACTGTTTTTGGTTGGGGTGACCAAGGGGAAAAATAAATCCCCCTTATCGATTGAGTACTCAAGTACTTAAAAATC
AGAATTACAATTCTGATTAATAAAATATTTATCGAAAAATGACCCAGGATTTCCTGATCAATGAACCAAGTTACCCTAGGGATAAC
AGCGCAATCCTTTCTTAGAGTCCCTATCGCCGAAAGGGTTTACGACCTCGATGTTGGATCAGGACATCCTAATGATGCAACCGTT
ATTAAGGGTTCGTT

No.2025-166-26-3

CCGCCTGCCCTGTGACAATGTTTTAACGGCCGCGGTATTTTGACCGTGCAAAGGTAGCGTAATCACTTGTCTTTTAAATGAAGAC
CCGTATGAAAGGCATCACGAGAGTTCAACTGTCTCTATTTTCTAATCAATGAAATTGATCTACTCGTGCAGAAGCGAGTATAACTA
CATTAGACGAGAAGACCCTATGGAGCTTCAAACACATGAATTAAATATGTAAACTAACTACTCCCCGGACATAAATAAAATAATAT
TTTTAATTTAACTGTTTTTGGTTGGGGTGACCAAGGGGAAAAATAAATCCCCCTTATCGATTGAGTACTCAAGTACTTAAAAATC
AGAATTACAATTCTGATTAATAAAATATTTATCGAAAAATGACCCAGGATTTCCTGATCAATGAACCAAGTTACCCTAGGGATAAC
AGCGCAATCCTTTCTTAGAGTCCCTATCGCCGAAAGGGTTTACGACCTCGATGTTGGATCAGGACATCCTAATGATGCAACCGTT
ATTAAGGGT

No.2025-166-26-4

CCGCCTGCCCTGTGACAATGTTTTAACGGCCGCGGTATTTTGACCGTGCAAAGGTAGCGTAATCACTTGTCTTTTAAATGAAGAC
CCGTATGAAAGGCATCACGAGAGTTCAACTGTCTCTATTTTCTAATCAATGAAATTGATCTACTCGTGCAGAAGCGAGTATAACTA
CATTAGACGAGAAGACCCTATGGAGCTTCAAACACATGAATTAAATATGTAAACTAACTACTCCCCGGACATAAATAAAATAATAT
TTTTAATTTAACTGTTTTTGGTTGGGGTGACCAAGGGGAAAAATAAATCCCCCTTATCGATTGAGTACTCAAGTACTTAAAAATC
AGAATTACAATTCTGATTAATAAAATATTTATCGAAAAATGACCCAGGATTTCCTGATCAATGAACCAAGTTACCCTAGGGATAAC
AGCGCAATCCTTTCTTAGAGTCCCTATCGCCGAAAGGGTTTACGACCTCGATGTTGGATCAGGACATCCTAATGATGCAACCGTT
ATTAAGGGT

No.2025-166-26-5

AGAGGTCCCGCCTGCCCTGTGACAATGTTTTAACGGCCGCGGTATTTTGACCGTGCAAAGGTAGCGTAATCACTTGTCTTTTAAA
TGAAGACCCGTATGAAAGGCATCACGAGAGTTCAACTGTCTCTATTTTCTAATCAATGAAATTGATCTACTCGTGCAGAAGCGAG
TATAACTACATTAGACGAGAAGACCCTATGGAGCTTCAAACACATGAATTAAATATGTAAACTAACTACTCCCCGGACATAAATAA
AATAATATTTTTAATTTAACTGTTTTTGGTTGGGGTGACCAAGGGGAAAAATAAATCCCCCTTATCGATTGAGTACTCAAGTACTT
AAAAATCAGAATTACAATTCTGATTAATAAAATATTTATCGAAAAATGACCCAGGATTTCCTGATCAATGAACCAAGTTACCCTAG
GGATAACAGCGCAATCCTTTCTTAGAGTCCCTATCGCCGAAAGGGTTTACGACCTCGATGTTGGATCAGGACATCCTAATGATGC
AACCGTTATTAAGGGTTCGTTTGTTCAACGATTAAT

No.2025-166-26-6

TGCCCTGTGACAATGTTTTAACGGCCGCGGTATTTTGACCGTGCAAAGGTAGCGTAATCACTTGTCTTTTAAATGAAGACCCGTA
TGAAAGGCATCACGAGAGTTTAACTGTCTCTATTTTCTAATCAATGAAATTGATCTACTCGTGCAGAAGCGAGTATAATCACATCA
GACGAGAAGACCCTATGGAGCTTCAAACACATAAATTAACTACATAAATTAATTATTCCACGGATATAAATAAAAATACAGTACCT
TTAATTTAACTGTTTTTGGTTGGGGTGACCAAGGGGAAAAACAAATCCCCCTTATCGACTGAGTACTCAAGTACTTAAAAATTAG
ATTTACAATTCTAATTAATAAAATATTTATCGAACAATGACCCAGGATTTCCTGATCAATGAACCAAGTTACCCTAGGGATAACAGC
GCAATCCTTTCTCAGAGTCCCTATCGCCGAAAGGGTTTACGACCTCGATGTTGGATCAGGACATCCTAATGATGCAACCGTTATT
AAGGGTTCGTTTGTTCAACGATTAATAGTCCTAC

No.2025-166-26-7

TGCCCTGTGACAATGTTTTAACGGCCGCGGTATTTTGACCGTGCAAAGGTAGCGTAATCACTTGTCTTTTAAATGAAGACCCGTA
TGAAAGGCATCACGAGAGTTCAACTGTCTCTATTTTCTAATCAATGAAATTGATCTACTCGTGCAGAAGCGAGTATAACTACATTA
GACGAGAAGACCCTATGGAGCTTCAAACACATGAATTAAATATGTAAACTAACTACTCCCCGGACATAAATAAAATAATATTTTTA
ATTTAACTGTTTTTGGTTGGGGTGACCAAGGGGAAAAATAAATCCCCCTTATCGATTGAGTACTCAAGTACTTAAAAATCAGAAT
TACAATTCTGATTAATAAAATATTTATCGAAAAATGACCCAGGATTTCCTGATCAATGAACCAAGTTACCCTAGGGATAACAGCGC
AATCCTTTCTTAGAGTCCCTATCGCCGAAAGGGTTTACGACCTCGATGTTGGATCAGGACATCCTAATGATGCAACCGTTATTAA
GGGTTCGTTTGTTCAACGATTAATAGTCCTACGTGAT

No.2025-166-26-8

AGAGGTCCCGCCTGCCCTGTGACAATGTTTTAACGGCCGCGGTATTTTGACCGTGCAAAGGTAGCGTAATCACTTGTCTTTTAAA
TGAAGACCCGTATGAAAGGCATCACGAGAGTTCAACTGTCTCTATTTTCTAATCAATGAAATTGATCTACTCGTGCAGAAGCGAG
TATAACTACATTAGACGAGAAGACCCTATGGAGCTTCAAACACATGAATTAAATATGTAAACTAACTACTCCCCGGACATAAATAA

AATAATATTTTTAATTTAACTGTTTTTGGTTGGGGTGACCAAGGGGAAAAATAAATCCCCCTTATCGATTGAGTACTCAAGTACTT
AAAAATCAGAATTACAATTCTGATTAATAAAATATTTATCGAAAAATGACCCAGGATTTCCTGATCAATGAACCAAGTTACCCTAG
GGATAACAGCGCAATCCTTTCTTAGAGTCCCTATCGCCGAAAGGGTTTACGACCTCGATGTTGGATCAGGACATCCTAATGATGC
AACCGTTATTAAGGGTTCGTTTGTTCAACGATTAAT

No.2025-166-26-9

CCGCCTGCCCTGTGACAATGTTTTAACGGCCGCGGTATTTTGACCGTGCAAAGGTAGCGTAATCACTTGTCTTTTAAATGAAGAC
CCGTATGAAAGGCATCACGAGAGTTCAACTGTCTCTATTTTCTAATCAATGAAATTGATCTACTCGTGCAGAAGCGAGTATAACTA
CATTAGACGAGAAGACCCTATGGAGCTTCAAACACATGAATTAAATATGTAAACTAACTACTCCCCGGACATAAATAAAATAATAT
TTTTAATTTAACTGTTTTTGGTTGGGGTGACCAAGGGGAAAAATAAATCCCCCTTATCGATTGAGTACTCAAGTACTTAAAAATC
AGAATTACAATTCTGATTAATAAAATATTTATCGAAAAATGACCCAGGATTTCCTGATCAATGAACCAAGTTACCCTAGGGATAAC
AGCGCAATCCTTTCTTAGAGTCCCTATCGCCGAAAGGGTTTACGACCTCGATGTTGGATCAGGACATCCTAATGATGCAACCGTT
ATTAAGGGT

No.2025-166-26-10

CCGCCTGCCCTGTGACAATGTTTTAACGGCCGCGGTATTTTGACCGTGCAAAGGTAGCGTAATCACTTGTCTTTTAAATGAAGAC
CCGTATGAAAGGCATCACGAGAGTTCAACTGTCTCTATTTTCTAATCAATGAAATTGATCTACTCGTGCAGAAGCGAGTATAACTA
CATTAGACGAGAAGACCCTATGGAGCTTCAAACACATGAATTAAATATGTAAACTAACTACTCCCCGGACATAAATAAAATAATAT
TTTTAATTTAACTGTTTTTGGTTGGGGTGACCAAGGGGAAAAATAAATCCCCCTTATCGATTGAGTACTCAAGTACTTAAAAATC
AGAATTACAATTCTGATTAATAAAATATTTATCGAAAAATGACCCAGGATTTCCTGATCAATGAACCAAGTTACCCTAGGGATAAC
AGCGCAATCCTTTCTTAGAGTCCCTATCGCCGAAAGGGTTTACGACCTCGATGTTGGATCAGGACATCCTAATGATGCAACCGTT
ATTAAGGGTTC

No.2025-166-26-11

CCGCCTGCCCTGTGACAATGTTTTAACGGCCGCGGTATTTTGACCGTGCAAAGGTAGCGTAATCACTTGTCTTTTAAATGAAGAC
CCGTATGAAAGGCATCACGAGAGTTCAACTGTCTCTATTTTCTAATCAATGAAATTGATCTACTCGTGCAGAAGCGAGTATAACTA
CATTAGACGAGAAGACCCTATGGAGCTTCAAACACATGAATTAAATATGTAAACTAACTACTCCCCGGACATAAATAAAATAATAT
TTTTAATTTAACTGTTTTTGGTTGGGGTGACCAAGGGGAAAAATAAATCCCCCTTATCGATTGAGTACTCAAGTACTTAAAAATC
AGAATTACAATTCTGATTAATAAAATATTTATCGAAAAATGACCCAGGATTTCCTGATCAATGAACCAAGTTACCCTAGGGATAAC
AGCGCAATCCTTTCTTAGAGTCCCTATCGCCGAAAGGGTTTACGACCTCGATGTTGGATCAGGACATCCTAATGATGCAACCGTT
ATTAAGGGTTCGTTTGTTCAACGATT

No.2025-166-26-12

CCCTGTGACAATGTTTTAACGGCCGCGGTATTTTGACCGTGCAAAGGTAGCGTAATCACTTGTCTTTTAAATGAAGACCCGTATG
AAAGGCATCACGAGAGTTTAACTGTCTCTATTTTCTAATCAATGAAATTGATCTACTCGTGCAGAAGCGAGTATAATCACATCAGA
CGAGAAGACCCTATGGAGCTTCAAACACATAAATTAACTACATAAATTAATTATTCCACGGATATAAATAAAAATATAGTACCTTTA
ATTTAACTGTTTTTGGTTGGGGTGACCAAGGGGAAAAACAAATCCCCCTTATCGACTGAGTACTCAAGTACTTAGAAATTAGATT
TACAATTCTAATTAATAAAATATTTATCGAACAATGACCCAGGATTTCCTGATCAATGAACCAAGTTACCCTAGGGATAACAGCGC
AATCCTTTCTCAGAGTCCCTATCGCCGAAAGGGTTTACGACCTCGATGTTGGATCAGGACATCCTAATGATGCAACCGTTATTAA
GGGTTCGTTTGTTCAACGATTAATAGTCCTACGT

No.2025-166-26-13

CCGCCTGCCCTGTGACAATGTTTTAACGGCCGCGGTATTTTGACCGTGCAAAGGTAGCGTAATCACTTGTCTTTTAAATGAAGAC
CCGTATGAAAGGCATCACGAGAGTTTAACTGTCTCTATTTTCTAATCAATGAAATTGATCTACTCGTGCAGAAGCGAGTATAATCA
CATCAGACGAGAAGACCCTATGGAGCTTCAAACACATAAATTAACTACATAAATTAATTATTCCACGGATATAAATAAAAATACAG
TACCTTTAATTTAACTGTTTTTGGTTGGGGTGACCAAGGGGAAAAACAAATCCCCCTTATCGACTGAGTACTCAAGTACTTAAAA
ATTAGATTTACAATTCTAATTAATAAAATATTTATCGAACAATGACCCAGGATTTCCTGATCAATGAACCAAGTTACCCTAGGGATA
ACAGCGCAATCCTTTCTCAGAGTCCCTATCGCCGAAAGGGTTTACGACCTCGATGTTGGATCAGGACATCCTAATGATGCAACC
GTTATTAAGGGT

No.2025-166-26-14

CCCTGTGACAATGTTTTAACGGCCGCGGTATTTTGACCGTGCAAAGGTAGCGTAATCACTTGTCTTTTAAATGAAGACCCGTATG
AAAGGCATCACGAGAGTTTAACTGTCTCTATTTTCTAATCAATGAAATTGATCTACTCGTGCAGAAGCGAGTATAATCACATCAGA
CGAGAAGACCCTATGGAGCTTCAAACACATAAATTAACTACATAAATTAATTATTCCACGGATATAAATAAAAATACAGTACCTTTA
ATTTAACTGTTTTTGGTTGGGGTGACCAAGGGGAAAAACAAATCCCCCTTATCGACTGAGTACTCAAGTACTTAAAAATTAGATT
TACAATTCTAATTAATAAAATATTTATCGAACAATGACCCAGGATTTCCTGATCAATGAACCAAGTTACCCTAGGGATAACAGCGC
AATCCTTTCTCAGAGTCCCTATCGCCGAAAGGGTTTACGACCTCGATGTTGGATCAGGACATCCTAATGATGCAACCGTTATTAA
GGGTTCGTT

No.2025-166-26-15

TGTGACAATGTTTTAACGGCCGCGGTATTTTGACCGTGCAAAGGTAGCGTAATCACTTGTCTTTTAAATGAAGACCCGTATGAAA
GGCATCACGAGAGTTTAACTGTCTCTATTTTCTAATCAATGAAATTGATCTACTCGTGCAGAAGCGAGTATAACCACATCAGACG
AGAAGACCCTATGGAGCTTCAAACACATAAATTAACTACATAAATTAATTATTCCACGGATATAAATAAAAATACAGTACCTTTAAT
TTAACTGTTTTTGGTTGGGGTGACCAAGGGGAAAAACAAATCCCCCTTATCGACTGAGTACTCAAGTACTTAAAAATTAGATTTA
CAATTCTAATTAATAAAATATTTATCGAACAATGACCCAGGATTTCCTGATCAATGAACCAAGTTACCCTAGGGATAACAGCGCAA
TCCTTTCTCAGAGTCCCTATCGCCGAAAGGGTTTACGACCTCGATGTTGGATCAGGACATCCTAATGATGCAACCGTTATTAAGG
GTTCGTTTGTTCAACGATTAATAGTCCTACGT

No.2025-166-26-16

CCGCCTGCCCTGTGACAATGTTTTAACGGCCGCGGTATTTTGACCGTGCAAAGGTAGCGTAATCACTTGTCTTTTAAATGAAGAC
CCGTATGAAAGGCATCACGAGAGTTTAACTGTCTCTATTTTCTAATCAATGAAATTGATCTACTCGTGCAGAAGCGAGTATAATCA

CATCAGACGAGAAGACCCTATGGAGCTTCAAACACATAAATTAACTACATAAATTAATTATTCCACGGATATAAATAAAAATACAG
TACCTTTAATTTAACTGTTTTTGGTTGGGGTGACCAAGGGGAAAAACAAATCCCCCTTATCGACTGAGTACTCAAGTACTTAAAA
ATTAGATTTACAATTCTAATTAATAAAATATTTATCGAACAATGACCCAGGATTTCCTGATCAATGAACCAAGTTACCCTAGGGATA
ACAGCGCAATCCTTTCTCAGAGTCCCTATCGCCGAAAGGGTTTACGACCTCGATGTTGGATCAGGACATCCTAATGATGCAACC
GTTATTAAGGGTTCGTT

No.2025-166-26-17

AGAGGTCCCGCCTGCCCTGTGACAATGTTTTAACGGCCGCGGTATTTTGACCGTGCAAAGGTAGCGTAATCACTTGTCTTTTAAA
TGAAGACCCGTATGAAAGGCATCACGAGAGTTTAACTGTCTCTATTTTCTAATCAATGAAATTGATCTACTCGTGCAGAAGCGAG
TATAATCACATCAGACGAGAAGACCCTATGGAGCTTCAAACACATAAATTAACTACATAAATTAATTATTCCACGGATATAAATAAA
AATACAGTACCTTTAATTTAACTGTTTTTGGTTGGGGTGACCAAGGGGAAAAACAAATCCCCCTTATCGACTGAGTACTCAAGTA
CTTAAAAATTAGATTTACAATTCTAATTAATAAAATATTTATCGAACAATGACCCAGGATTTCCTGATCAATGAACCAAGTTACCCT
AGGGATAACAGCGCAATCCTTTCTCAGAGTCCCTATCGCCGAAAGGGTTTACGACCTCGATGTTGGATCAGGACATCCTAATGAT
GCAACCGTTATTAAGGGTTCGTT

No.2025-166-26-18

AGAGGTCCCGCCTGCCCTGTGACAATGTTTTAACGGCCGCGGTATTTTGACCGTGCAAAGGTAGCGTAATCACTTGTCTTTTAAA
TGAAGACCCGTATGAAAGGCATCACGAGAGTTCAACTGTCTCTATTTTCTAATCAATGAAATTGATCTACTCGTGCGGAAGCGAG
TATAACTACATTAGACGAGAAGACCCTATGGAGCTTCAAACACATGAATTAAATATGTAAACTAACTACTCCCCGGACATAAATAA
AATAATATTTTTAATTTAACTGTTTTTGGTTGGGGTGACCAAGGGGAAAAATAAATCCCCCTTATCGATTGAGTACTCAAGTACTT
AAAAATCAGAATTACAATTCTGATTAATAAAATATTTATCGAAAAATGACCCAGGATTTCCTGATCAATGAACCAAGTTACCCTAG
GGATAACAGCGCAATCCTTTCTTAGAGTCCCTATCGCCGAAAGGGTTTACGACCTCGATGTTGGATCAGGACATCCTAATGATGC
AACCGTTATTAAGGGTTCGTTTGTTCAACGAT

No.2025-166-26-19

AGAGGTCCCGCCTGCCCTGTGACAATGTTTTAACGGCCGCGGTATTTTGACCGTGCAAAGGTAGCGTAATCACTTGTCTTTTAAA
TGAAGACCCGTATGAAAGGCATCACGAGAGTTTAACTGTCTCTATTTTCTAATCAATGAAATTGATCTACTCGTGCAGAAGCGAG
TATAATCACATCAGACGAGAAGACCCTATGGAGCTTCAAACACATAAATTAACTACATAAATTAATTATTCCACGGATATAAATAAA
AATACAGTACCTTTAATTTAACTGTTTTTGGTTGGGGTGACCAAGGGGAAAAACAAATCCCCCTTATCGACTGAGTACTCAAGTA
CTTAAAAATTAGATTTACAATTCTAATTAATAAAATATTTATCGAACAATGACCCAGGATTTCCTGATCAATGAACCAAGTTACCCT
AGGGATAACAGCGCAATCCTTTCTCAGAGTCCCTATCGCCGAAAGGGTTTACGACCTCGATGTTGGATCAGGACATCCTAATGAT
GCAACCGTTATTAAGGGTTCGTT

No.2025-166-26-20

CCGCCTGCCCTGTGACAATGTTTTAACGGCCGCGGTATTTTGACCGTGCAAAGGTAGCGTAATCACTTGTCTTTTAAATGAAGAC
CCGTATGAAAGGCATCACGAGAGTTTAACTGTCTCTATTTTCTAATCAATGAAATTGATCTACTCGTGCAGAAGCGAGTATAATCA
CATCAGACGAGAAGACCCTATGGAGCTTCAAACACATAAATTAACTACATAAATTAATTATTCCACGGATATAAATAAAAATACAG
TACCTTTAATTTAACTGTTTTTGGTTGGGGTGACCAAGGGGAAAAACAAATCCCCCTTATCGACTGAGTACTCAAGTACTTAAAA
ATTAGATTTACAATTCTAATTAATAAAATATTTATCGAACAATGACCCAGGATTTCCTGATCAATGAACCAAGTTACCCTAGGGATA
ACAGCGCAATCCTTTCTCAGAGTCCCTATCGCCGAAAGGGTTTACGACCTCGATGTTGGATCAGGACATCCTAATGATGCAACC
GTTATTAAGGGT

No.2025-166-26-21

AGAGGTCCCGCCTGCCCTGTGACAATGTTTTAACGGCCGCGGTATTTTGACCGTGCAAAGGTAGCGTAATCACTTGTCTTTTAAA
TGAAGACCCGTATGAAAGGCATCACGAGAGTTTAACTGTCTCTATTTTCTAATCAATGAAATTGATCTACTCGTGCAGAAGCGAG
TATAATCACATCAGACGAGAAGACCCTATGGAGCTTCAAACACATAAATTAACTACATAAATTAATTATTCCACGGATATAAATAAA
AATACAGTACCTTTAATTTAACTGTTTTTGGTTGGGGTGACCAAGGGGAAAAACAAATCCCCCTTATCGACTGAGTACTCAAGTA
CTTAAAAATTAGATTTACAATTCTAATTAATAAAATATTTATCGAACAATGACCCAGGATTTCCTGATCAATGAACCAAGTTACCCT
AGGGATAACAGCGCAATCCTTTCTCAGAGTCCCTATCGCCGAAAGGGTTTACGACCTCGATGTTGGATCAGGACATCCTAATGAT
GCAACCGTTATTAAGGGTTCGTT

No.2025-166-26-22

AGAGGTCCCGCCTGCCCTGTGACAATGTTTTAACGGCCGCGGTATTTTGACCGTGCAAAGGTAGCGTAATCACTTGTCTTTTAAA
TGAAGACCCGTATGAAAGGCATCACGAGAGTTTAACTGTCTCTATTTTCTAATCAATGAAATTGATCTACTCGTGCAGAAGCGAG
TATAATCACATCAGACGAGAAGACCCTATGGAGCTTCAAACACATAAATTAACTACATAAATTAATTATTCCACGGATATAAATAAA
AATACAGTACCTTTAATTTAACTGTTTTTGGTTGGGGTGACCAAGGGGAAAAACAAATCCCCCTTATCGACTGAGTACTCAAGTA
CTTAAAAATTAGATTTACAATTCTAATTAATAAAATATTTATCGAACAATGACCCAGGATTTCCTGATCAATGAACCAAGTTACCCT
AGGGATAACAGCGCAATCCTTTCTCAGAGTCCCTATCGCCGAAAGGGTTTACGACCTCGATGTTGGATCAGGACATCCTAATGAT
GCAACCGTTATTAAGGGT

No.2025-166-26-23

CCGCCTGCCCTGTGACAACGTTCAACGGCCGCGGTATTTTGACCGTGCAAAGGTAGCGTAATCACTTGTCTTTTAAATGAAGAC
CTGTATGAAAGGCATCACGAGAGTTTAACTGTCTCTATTTTCTAATCAATGAAATTGATCTATTCGTGCAGAAGCGAATATAATAA
CATTAGACGAGAAGACCCTATGGAGCTTCAAACACTTAAATTAATTATGTAATCCTCCACCTCCCAGGGAATAAACAAAATATACA
ATACTTCTAATTTAACTGTTTTTGGTTGGGGTGACCAAGGGGAAAAACAAATCCCCCTCATCGATTGAGTACTAAGTACTTAAAA
ATTAGAATGACAATTCTAATTAATAAAACATTTATCGAAAAATGACCCAGGATTTCCTGATCAATGAACCAAGTTACCCTAGGGAT
AACAGCGCAATCCTTTCTCAGAGTCCCTATCGAAGAAAGGGTTTACGACCTCGATGTTGGATCAGGACATCCTAATGGTGCAAC
CGCTATTAAGGGTTCGTT

No.2025-166-26-24

CCGCCTGCCCTGTGACAATGTTTTAACGGCCGCGGTATTTTGACCGTGCAAAGGTAGCGTAATCACTTGTCTTTTAAATGAAGAC

CCGTATGAAAGGCATCACGAGAGTTTAACTGTCTCTATTTTCTAATCAATGAAATTGATCTACTCGTGCAGAAGCGAGTATAATCA
CATCAGACGAGAAGACCCTATGGAGCTTCAAACACATAAATTAACTACATAAATTAATTATTCCACGGATATAAATAAAAATACAG
TACCTTTAATTTAACTGTTTTTGGTTGGGGTGACCAAGGGGAAAAACAAATCCCCCTTATCGACTGAGTACTCAAGTACTTAAAA
ATTAGATTTACAATTCTAATTAATAAAATATTTATCGAACAATGACCCAGGATTTCCTGATCAATGAACCAAGTTACCCTAGGGATA
ACAGCGCAATCCTTTCTCAGAGTCCCTATCGCCGAAAGGGTTTACGACCTCGATGTTGGATCAGGACATCCTAATGATGCAACC
GTTATTAAGGGTTCGTT

No.2025-166-26-25

AGAGGTCCCGCCTGCCCTGTGACAATGTTTTAACGGCCGCGGTATTTTGACCGTGCAAAGGTAGCGTAATCACTTGTCTTTTAAA
TGAAGACCCGTATGAAAGGCATCACGAGAGTTCAACTGTCTCTATTTTCTAATCAATGAAATTGATCTACTCGTGCGGAAGCGAG
TATAACTACATTAGACGAGAAGACCCTATGGAGCTTCAAACACATGAATTAAATATGTAAACTAACTACTCCCCGGACATAAATAA
AATAATATTTTTAATTTAACTGTTTTTGGTTGGGGTGACCAAGGGGAAAAATAAATCCCCCTTATCGATTGAGTACTCAAGTACTT
AAAAATCAGAATTACAATTCTGATTAATAAAATATTTATCGAAAAATGACCCAGGATTTCCTGATCAATGAACCAAGTTACCCTAG
GGATAACAGCGCAATCCTTTCTTAGAGTCCCTATCGCCGAAAGGGTTTACGACCTCGATGTTGGATCAGGACATCCTAATGATGC
AACCGTTATTAAGGGTTCGTT

No.2025-166-26-26

CCGCCTGCCCTGTGACAATGTTTTAACGGCCGCGGTATTTTGACCGTGCAAAGGTAGCGTAATCACTTGTCTTTTAAATGAAGAC
CCGTATGAAAGGCATCACGAGAGTTTAACTGTCTCTATTTTCTAATCAATGAAATTGATCTACTCGTGCAGAAGCGAGTATAATCA
CATCAGACGAGAAGACCCTATGGAGCTTCAAACACATAAATTAACTACATAAATTAATTATTCCACGGATATAAATAAAAATACAG
TACCTTTAATTTAACTGTTTTTGGTTGGGGTGACCAAGGGGAAAAACAAATCCCCCTTATCGACTGAGTACTCAAGTACTTAAAA
ATTAGATTTACAATTCTAATTAATAAAATATTTATCGAACAATGACCCAGGATTTCCTGATCAATGAACCAAGTTACCCTAGGGATA
ACAGCGCAATCCTTTCTCAGAGTCCCTATCGCCGAAAGGGTTTACGACCTCGATGTTGGATCAGGACATCCTAATGATGCAACC
GTTATTAAGGGTTCGTT

No.2025-166-26-27

CCGCCTGCCCTGTGACAATGTTTTAACGGCCGCGGTATTTTGACCGTGCAAAGGTAGCGTAATCACTTGTCTTTTAAATGAAGAC
CCGTATGAAAGGCATCACGAGAGTTTAACTGTCTCTATTTTCTAATCAATGAAATTGATCTACTCGTGCAGAAGCGAGTATAATCA
CATCAGACGAGAAGACCCTATGGAGCTTCAAACACATAAATTAACTACATAAATTAATTATTCCACGGATATAAATAAAAATACAG
TACCTTTAATTTAACTGTTTTTGGTTGGGGTGACCAAGGGGAAAAACAAATCCCCCTTATCGACTGAGTACTCAAGTACTTAAAA
ATTAGATTTACAATTCTAATTAATAAAATATTTATCGAACAATGACCCAGGATTTCCTGATCAATGAACCAAGTTACCCTAGGGATA
ACAGCGCAATCCTTTCTCAGAGTCCCTATCGCCGAAAGGGTTTACGACCTCGATGTTGGATCAGGACATCCTAATGATGCAACC
GTTATTAAGGGTTCGTTTGTTCAACGAT

No.2025-166-26-28

AGAGGTCCCGCCTGCCCTGTGACAATGTTTTAACGGCCGCGGTATTTTGACCGTGCAAAGGTAGCGTAATCACTTGTCTTTTAAA
TGAAGACCCGTATGAAAGGCATCACGAGAGTTCAACTGTCTCTATTTTCTAATCAATGAAATTGATCTACTCGTGCAGAAGCGAG
TATAACTACATTAGACGAGAAGACCCTATGGAGCTTCAAACACATGAATTAAATATGTAAACTAACTACTCCCCGGACATAAATAA
AATAATATTTTTAATTTAACTGTTTTTGGTTGGGGTGACCAAGGGGAAAAATAAATCCCCCTTATCGATTGAGTACTCAAGTACTT
AAAAATCAGAATTACAATTCTGATTAATAAAATATTTATCGAAAAATGACCCAGGATTTCCTGATCAATGAACCAAGTTACCCTAG
GGATAACAGCGCAATCCTTTCTTAGAGTCCCTATCGCCGAAAGGGTTTACGACCTCGATGTTGGATCAGGACATCCTAATGATGC
AACCGTTATTAAGGGTTCGTT

No.2025-166-26-29

CCGCCTGCCCTGTGACAATGTTTTAACGGCCGCGGTATTTTGACCGTGCAAAGGTAGCGTAATCACTTGTCTTTTAAATGAAGAC
CCGTATGAAAGGCATCACGAGAGTTTAACTGTCTCTATTTTCTAATCAATGAAATTGATCTACTCGTGCAGAAGCGAGTATAATCA
CATCAGACGAGAAGACCCTATGGAGCTTCAAACACATAAATTAACTACATAAATTAATTATTCCACGGATATAAATAAAAATACAG
TACCTTTAATTTAACTGTTTTTGGTTGGGGTGACCAAGGGGAAAAACAAATCCCCCTTATCGACTGAGTACTCAAGTACTTAAAA
ATTAGATTTACAATTCTAATTAATAAAATATTTATCGAACAATGACCCAGGATTTCCTGATCAATGAACCAAGTTACCCTAGGGATA
ACAGCGCAATCCTTTCTCAGAGTCCCTATCGCCGAAAGGGTTTACGACCTCGATGTTGGATCAGGACATCCTAATGATGCAACC
GTTATTAAGGGT

No.2025-166-26-30

CCGCCTGCCCTGTGACAATGTTTTAACGGCCGCGGTATTTTGACCGTGCAAAGGTAGCGTAATCACTTGTCTTTTAAATGAAGAC
CCGTATGAAAGGCATCACGAGAGTTTAACTGTCTCTATTTTCTAATCAATGAAATTGATCTACTCGTGCAGAAGCGAGTATAATCA
CATCAGACGAGAAGACCCTATGGAGCTTCAAACACATAAATTAACTACATAAATTAATTATTCCACGGATATAAATAAAAATATAGT
ACCTTTAATTTAACTGTTTTTGGTTGGGGTGACCAAGGGGAAAAACAAATCCCCCTTATCGACTGAGTACTCAAGTACTTAGAAA
TTAGATTTACAATTCTAATTAATAAAATATTTATCGAACAATGACCCAGGATTTCCTGATCAATGAACCAAGTTACCCTAGGGATAA
CAGCGCAATCCTTTCTCAGAGTCCCTATCGCCGAAAGGGTTTACGACCTCGATGTTGGATCAGGACATCCTAATGATGCAACCGT
TATTAAGGGTTCGTT

No.2025-166-26-31

CCGCCTGCCCTGTGACAATGTTTTAACGGCCGCGGTATTTTGACCGTGCAAAGGTAGCGTAATCACTTGTCTTTTAAATGAAGAC
CCGTATGAAAGGCATCACGAGAGTTTAACTGTCTCTATTTTCTAATCAATGAAATTGATCTACTCGTGCAGAAGCGAGTATAATCA
CATCAGACGAGAAGACCCTATGGAGCTTCAAACACATAAATTAACTACATAAATTAATTATTCCACGGATATAAATAAAAATACAG
TACCTTTAATTTAACTGTTTTTGGTTGGGGTGACCAAGGGGAAAAACAAATCCCCCTTATCGACTGAGTACTCAAGTACTTAAAA
ATTAGATTTACAATTCTAATTAATAAAATATTTATCGAACAATGACCCAGGATTTCCTGATCAATGAACCAAGTTACCCTAGGGATA
ACAGCGCAATCCTTTCTCAGAGTCCCTATCGCCGAAAGGGTTTACGACCTCGATGTTGGATCAGGACATCCTAATGATGCAACC
GTTATTAAGGGTTCGTT

No.2025-166-26-32

CCGCCTGCCCTGTGACAATGTTTTAACGGCCGCGGTATTTTGACCGTGCAAAGGTAGCGTAATCACTTGTCTTTTAAATGAAGAC
CCGTATGAAAGGCATCACGAGAGTTTAACTGTCTCTATTTTCTAATCAATGAAATTGATCTACTCGTGCAGAAGCGAGTATAATCA
CATCAGACGAGAAGACCCTATGGAGCTTCAAACACATAAATTAACTACATAAATTAATTATTCCACGGATATAAATAAAAATACAG
TACCTTTAATTTAACTGTTTTTGGTTGGGGTGACCAAGGGGAAAAACAAATCCCCCTTATCGACTGAGTACTCAAGTACTTAAAA
ATTAGATTTACAATTCTAATTAATAAAATATTTATCGAACAATGACCCAGGATTTCCTGATCAATGAACCAAGTTACCCTAGGGATA
ACAGCGCAATCCTTTCTCAGAGTCCCTATCGCCGAAAGGGTTTACGACCTCGATGTTGGATCAGGACATCCTAATGATGCAACC
GTTATTAAGGGTTCGTT

No.2025-166-26-33

CCGCCTGCCCTGTGACAATGTTTTAACGGCCGCGGTATTTTGACCGTGCAAAGGTAGCGTAATCACTTGTCTTTTAAATGAAGAC
CCGTATGAAAGGCATCACGAGAGTTTAACTGTCTCTATTTTCTAATCAATGAAATTGATCTACTCGTGCAGAAGCGAGTATAATCA
CATCAGACGAGAAGACCCTATGGAGCTTCAAACACATAAATTAACTACATAAATTAATTATTCCACGGATATAAATAAAAATACAG
TACCTTTAATTTAACTGTTTTTGGTTGGGGTGACCAAGGGGAAAAACAAATCCCCCTTATCGACTGAGTACTCAAGTACTTAAAA
ATTAGATTTACAATTCTAATTAATAAAATATTTATCGAACAATGACCCAGGATTTCCTGATCAATGAACCAAGTTACCCTAGGGATA
ACAGCGCAATCCTTTCTCAGAGTCCCTATCGCCGAAAGGGTTTACGACCTCGATGTTGGATCAGGACATCCTAATGATGCAACC
GTTATTAAGGGTTCGTTTGTTCAACGAT

No.2025-166-26-34

AGAGGTCCCGCCTGCCCTGTGACAATGTTTTAACGGCCGCGGTATTTTGACCGTGCAAAGGTAGCGTAATCACTTGTCTTTTAAA
TGAAGACCCGTATGAAAGGCATCACGAGAGTTCAACTGTCTCTATTTTCTAATCAATGAAATTGATCTACTCGTGCAGAAGCGAG
TATAACTACATTAGACGAGAAGACCCTATGGAGCTTCAAACACATGAATTAAATATGTAAACTAACTACTCCCCGGACATAAATAA
AATAATATTTTTAATTTAACTGTTTTTGGTTGGGGTGACCAAGGGGAAAAATAAATCCCCCTTATCGATTGAGTACTCAAGTACTT
AAAAATCAGAATTACAATTCTGATTAATAAAATATTTATCGAAAAATGACCCAGGATTTCCTGATCAATGAACCAAGTTACCCTAG
GGATAACAGCGCAATCCTTTCTTAGAGTCCCTATCGCCGAAAGGGTTTACGACCTCGATGTTGGATCAGGACATCCTAATGATGC
AACCGTTATTAAGGGTTCGTT

No.2025-166-26-35

CCGCCTGCCCTGTGACAATGTTTTAACGGCCGCGGTATTTTGACCGTGCAAAGGTAGCGTAATCACTTGTCTTTTAAATGAAGAC
CCGTATGAAAGGCATCACGAGAGTTCAACTGTCTCTATTTTCTAATCAATGAAATTGATCTACTCGTGCAGAAGCGAGTATAACTA
CATTAGACGAGAAGACCCTATGGAGCTTCAAACACATGAATTAAATATGTAAACTAACTACTCCCCGGACATAAATAAAATAATAT
TTTTAATTTAACTGTTTTTGGTTGGGGTGACCAAGGGGAAAAATAAATCCCCCTTATCGATTGAGTACTCAAGTACTTAAAAATC
AGAATTACAATTCTGATTAATAAAATATTTATCGAAAAATGACCCAGGATTTCCTGATCAATGAACCAAGTTACCCTAGGGATAAC
AGCGCAATCCTTTCTTAGAGTCCCTATCGCCGAAAGGGTTTACGACCTCGATGTTGGATCAGGACATCCTAATGATGCAACCGTT
ATTAAGGGTTCGTT

No.2025-166-26-36

CATAAGAGGTCCCGCCTGCCCTGTGACAACGTTCAACGGCCGCGGTATTTTGACCGTGCAAAGGTAGCGTAATCACTTGTCTTT
TAAATGAAGACCTGTATGAAAGGCATCACGAGAGTTTAACTGTCTCTATTTTCTAATCAATGAAATTGATCTATTCGTGCAGAAGC
GAATATAATAACATTAGACGAGAAGACCCTATGGAGCTTCAAACACTTAAATTAATTATGTAATCCTCCACCTCCCAGGGAATAAA
CAAAATATACAATACTTCTAATTTAACTGTTTTTGGTTGGGGTGACCAAGGGGAAAAACAAATCCCCCTCATCGATTGAGTACTA
AGTACTTAAAAATTAGAATGACAATTCTAATTAATAAAACATTTATCGAAAAATGACCCAGGATTTCCTGATCAATGAACCAAGTT
ACCCTAGGGATAACAGCGCAATCCTTTCTCAGAGTCCCTATCGAAGAAAGGGTTTACGACCTCGATGTTGGATCAGGACATCCT
AATGGTGCAACCGCTATTAAGGGTTCGTT

No.2025-166-26-37

AGAGGTCCCGCCTGCCCTGTGACAACGTTCAACGGCCGCGGTATTTTGACCGTGCAAAGGTAGCGTAATCACTTGTCTTTTAAA
TGAAGACCTGTATGAAAGGCATCACGAGAGTTTAACTGTCTCTATTTTCTAATCAATGAAATTGATCTATTCGTGCAGAAGCGAAT
ATAATAACATTAGACGAGAAGACCCTATGGAGCTTCAAACACTTAAATTAATTATGTAATCCTCCACCTCCCAGGGAATAAACAAA
ATATACAATACTTCTAATTTAACTGTTTTTGGTTGGGGTGACCAAGGGGAAAAACAAATCCCCCTCATCGATTGAGTACTAAGTAC
TTAAAAATTAGAATGACAATTCTAATTAATAAAACATTTATCGAAAAATGACCCAGGATTTCCTGATCAATGAACCAAGTTACCCT
AGGGATAACAGCGCAATCCTTTCTCAGAGTCCCTATCGAAGAAAGGGTTTACGACCTCGATGTTGGATCAGGACATCCTAATGG
TGCAACCGCTATTAAGGGTTCGTT

No.2025-166-26-38

CCGCCTGCCCTGTGACAATGTTTTAACGGCCGCGGTATTTTGACCGTGCAAAGGTAGCGTAATCACTTGTCTTTTAAATGAAGAC
CCGTATGAAAGGCATCACGAGAGTTTAACTGTCTCTATTTTCTAATCAATGAAATTGATCTACTCGTGCAGAAGCGAGTATAATCA
CATCAGACGAGAAGACCCTATGGAGCTTCAAACACATAAATTAACTACATAAATTAATTATTCCACGGATATAAATAAAAATACAG
TACCTTTAATTTAACTGTTTTTGGTTGGGGTGACCAAGGGGAAAAACAAATCCCCCTTATCGACTGAGTACTCAAGTACTTAAAA
ATTAGATTTACAATTCTAATTAATAAAATATTTATCGAACAATGACCCAGGATTTCCTGATCAATGAACCAAGTTACCCTAGGGATA
ACAGCGCAATCCTTTCTCAGAGTCCCTATCGCCGAAAGGGTTTACGACCTCGATGTTGGATCAGGACATCCTAATGATGCAACC
GTTATTAAGGGTTCGTT

No.2025-166-26-39

CCGCCTGCCCTGTGACAACGTTCAACGGCCGCGGTATTTTGACCGTGCAAAGGTAGCGTAATCACTTGTCTTTTAAATGAAGAC
CTGTATGAAAGGCATCACGAGAGTTTAACTGTCTCTATTTTCTAATCAATGAAATTGATCTATTCGTGCAGAAGCGAATATAATAA
CATTAGACGAGAAGACCCTATGGAGCTTCAAACACTTAAATTAATTATGTAATCCTCCACCTCCCAGGGAATAAACAAAATATACA
ATACTTCTAATTTAACTGTTTTTGGTTGGGGTGACCAAGGGGAAAAACAAATCCCCCTCATCGATTGAGTACTAAGTACTTAAAA
ATTAGAATGACAATTCTAATTAATAAAACATTTATCGAAAAATGACCCAGGATTTCCTGATCAATGAACCAAGTTACCCTAGGGAT
AACAGCGCAATCCTTTCTCAGAGTCCCTATCGAAGAAAGGGTTTACGACCTCGATGTTGGATCAGGACATCCTAATGGTGCAAC
CGCTATTAAGGGTTCGTT

No.2025-166-26-40

TAAGAGGTCCCGCCTGCCCTGTGACAACGTTCAACGGCCGCGGTATTTTGACCGTGCAAAGGTAGCGTAATCACTTGTCTTTTA
AATGAAGACCTGTATGAAAGGCATCACGAGAGTTTAACTGTCTCTATTTTCTAATCAATGAAATTGATCTATTCGTGCAGAAGCG
AATATAATAACATTAGACGAGAAGACCCTATGGAGCTTCAAACACTTAAATTAATTATGTAATCCTCCACCTCCCAGGGAATAAAC
AAAATATACAATACTTCTAATTTAACTGTTTTTGGTTGGGGTGACCAAGGGGAAAAACAAATCCCCCTCATCGATTGAGTACTAA
GTACTTAAAAATTAGAATGACAATTCTAATTAATAAAACATTTATCGAAAAATGACCCAGGATTTCCTGATCAATGAACCAAGTTA
CCCTAGGGATAACAGCGCAATCCTTTCTCAGAGTCCCTATCGAAGAAAGGGTTTACGACCTCGATGTTGGATCAGGACATCCTA
ATGGTGCAACCGCTATTAAGGGTTCGTT

No.2025-166-26-41

CCGCCTGCCCTGTGACAACGTTCAACGGCCGCGGTATTTTGACCGTGCAAAGGTAGCGTAATCACTTGTCTTTTAAATGAAGAC
CTGTATGAAAGGCATCACGAGAGTTTAACTGTCTCTATTTTCTAATCAATGAAATTGATCTATTCGTGCAGAAGCGAATATAATAA
CATTAGACGAGAAGACCCTATGGAGCTTCAAACACTTAAATTAATTATGTAATCCTCCACCTCCCAGGGAATAAACAAAATATACA
ATACTTCTAATTTAACTGTTTTTGGTTGGGGTGACCAAGGGGAAAAACAAATCCCCCTCATCGATTGAGTACTAAGTACTTAAAA
ATTAGAATGACAATTCTAATTAATAAAACATTTATCGAAAAATGACCCAGGATTTCCTGATCAATGAACCAAGTTACCCTAGGGAT
AACAGCGCAATCCTTTCTCAGAGTCCCTATCGAAGAAAGGGTTTACGACCTCGATGTTGGATCAGGACATCCTAATGGTGCAAC
CGCTATTAAGGGTTCGTT

No.2025-166-26-42

CCGCCTGCCCTGTGACAACGTTCAACGGCCGCGGTATTTTGACCGTGCAAAGGTAGCGTAATCACTTGTCTTTTAAATGAAGAC
CTGTATGAAAGGCATCACGAGAGTTTAACTGTCTCTATTTTCTAATCAATGAAATTGATCTATTCGTGCAGAAGCGAATATAATAA
CATTAGACGAGAAGACCCTATGGAGCTTCAAACACTTAAATTAATTATGTAATCCTCCACCTCCCAGGGAATAAACAAAATATACA
ATACTTCTAATTTAACTGTTTTTGGTTGGGGTGACCAAGGGGAAAAACAAATCCCCCTCATCGATTGAGTACTAAGTACTTAAAA
ATTAGAATGACAATTCTAATTAATAAAACATTTATCGAAAAATGACCCAGGATTTCCTGATCAATGAACCAAGTTACCCTAGGGAT
AACAGCGCAATCCTTTCTCAGAGTCCCTATCGAAGAAAGGGTTTACGACCTCGATGTTGGATCAGGACATCCTAATGGTGCAAC
CGCTATTAAGGGTTCGTT

No.2025-166-26-43

AGAGGTCCCGCCTGCCCTGTGACAACGTTCAACGGCCGCGGTATTTTGACCGTGCAAAGGTAGCGTAATCACTTGTCTTTTAAA
TGAAGACCTGTATGAAAGGCATCACGAGAGTTTAACTGTCTCTATTTTCTAATCAATGAAATTGATCTATTCGTGCAGAAGCGAAT
ATAATAACATTAGACGAGAAGACCCTATGGAGCTTCAAACACTTAAATTAATTATGTAATCCTCCACCTCCCAGGGAATAAACAAA
ATATACAATACTTCTAATTTAACTGTTTTTGGTTGGGGTGACCAAGGGGAAAAACAAATCCCCCTCATCGATTGAGTACTAAGTAC
TTAAAAATTAGAATGACAATTCTAATTAATAAAACATTTATCGAAAAATGACCCAGGATTTCCTGATCAATGAACCAAGTTACCCT
AGGGATAACAGCGCAATCCTTTCTCAGAGTCCCTATCGAAGAAAGGGTTTACGACCTCGATGTTGGATCAGGACATCCTAATGG
TGCAACCGCTATTAAGGGTTCGTT

No.2025-166-26-44

AGAGGTCCCGCCTGCCCTGTGACAACGTTCAACGGCCGCGGTATTTTGACCGTGCAAAGGTAGCGTAATCACTTGTCTTTTAAA
TGAAGACCTGTATGAAAGGCATCACGAGAGTTTAACTGTCTCTATTTTCTAATCAATGAAATTGATCTATTCGTGCAGAAGCGAAT
ATAATAACATTAGACGAGAAGACCCTATGGAGCTTCAAACACTTAAATTAATTATGTAATCCTCCACCTCCCAGGGAATAAACAAA
ATATACAATACTTCTAATTTAACTGTTTTTGGTTGGGGTGACCAAGGGGAAAAACAAATCCCCCTCATCGATTGAGTACTAAGTAC
TTAAAAATTAGAATGACAATTCTAATTAATAAAACATTTATCGAAAAATGACCCAGGATTTCCTGATCAATGAACCAAGTTACCCT
AGGGATAACAGCGCAATCCTTTCTCAGAGTCCCTATCGAAGAAAGGGTTTACGACCTCGATGTTGGATCAGGACATCCTAATGG
TGCAACCGCTATTAAGGGTTCGTT

No.2025-166-26-45

CCGCCTGCCCTGTGACAATGTTTTAACGGCCGCGGTATTTTGACCGTGCAAAGGTAGCGTAATCACTTGTCTTTTAAATGAAGAC
CCGTATGAAAGGCATCACGAGAGTTCAACTGTCTCTATTTTCTAATCAATGAAATTGATCTACTCGTGCAGAAGCGAGTATAACTA
CATTAGACGAGAAGACCCTATGGAGCTTCAAACACATGAATTAAATATGTAAACTAACTACTCCCCGGACATAAATAAAATAATAT
TTTTAATTTAACTGTTTTTGGTTGGGGTGACCAAGGGGAAAAATAAATCCCCCTTATCGATTGAGTACTCAAGTACTTAAAAATC
AGAATTACAATTCTGATTAATAAAATATTTATCGAAAAATGACCCAGGATTTCCTGATCAATGAACCAAGTTACCCTAGGGATAAC
AGCGCAATCCTTTCTTAGAGTCCCTATCGCCGAAAGGGTTTACGACCTCGATGTTGGATCAGGACATCCTAATGATGCAACCGTT
ATTAAGGGTTCGTT

No.2025-166-26-46

CCGCCTGCCCTGTGACAATGTTTTAACGGCCGCGGTATTTTGACCGTGCAAAGGTAGCGTAATCACTTGTCTTTTAAATGAAGAC
CCGTATGAAAGGCATCACGAGAGTTTAACTGTCTCTATTTTCTAATCAATGAAATTGATCTACTCGTGCAGAAGCGAGTATAATCA
CATCAGACGAGAAGACCCTATGGAGCTTCAAACACATAAATTAACTACATAAATTAATAATTCCACGGATATAAATAAAAATACAG
TACCTTTAATTTAACTGTTTTTGGTTGGGGTGACCAAGGGGAAAAACAAATCCCCCTTATCGACTGAGTACTCAAGTACTTAAAA
ATTAGATTTACAATTCTAATTAATAAAATATTTATCGAACAATGACCCAGGATTTCCTGATCAATGAACCAAGTTACCCTAGGGATA
ACAGCGCAATCCTTTCTCAGAGTCCCTATCGCCGAAAGGGTTTACGACCTCGATGTTGGATCAGGACATCCTAATGATGCAACC
GTTATTAAGGGTTCGTT

No.2025-166-26-47

AGAGGTCCCGCCTGCCCTGTGACAATGTTTAACGGCCGCGGTATTCTGACCGTGCAAAGGTAGCGTAATCACTTGTCTTTTAAAT
GAAGACCCGTATGAAAGGCATCACGAGAGTTCAACTGTCTCTACTTTCCAATCAATGAAATTGATCCACCCGTGCAGAAGCGGG
TATAAACACATCAGACGAGAAGACCCTATGGAGCTTCAAACACATGAATTAATTATGTAGACTAACTGCTCCACGGACATAAATA
AAAATACAACACTTTTAATTTAACTGTTTTGGTTGGGGTGACCGAGGGGAAAAATCAATCCCCCTTATCGACCGAGTGTTCTCAA
GCACTTAAAAATTAGAATTACAATTCTAATTAGTAAAATATTTACCGAAAAATGACCCAGAATTTTCTGATCAATGAACCAAGTTA
CCCTAGGGATAACAGCGCAATCCTTTCCTAGAGTCCCTATCGACGAAAGGGTTTACGACCTCGATGTTGGATCAGGACATCCTAA
TGATGTAGCCGTTATTAAGGGTTCGTT

No.2025-166-26-48

CCGCCTGCCCTGTGACAACGTTCAACGGCCGCGGTATTTTGACCGTGCAAAGGTAGCGTAATCACTTGTCTTTTAAATGAAGAC
CTGTATGAAAGGCATCACGAGAGTTTAACTGTCTCTATTTTCTAATCAATGAAATTGATCTATTCGTGCAGAAGCGAATATAATAA
CATTAGACGAGAAGACCCTATGGAGCTTCAAACACTTAAATTAATTATGTAATCCTCCACCTCCCAGGGAATAAACAAAATATACA
ATACTTCTAATTTAACTGTTTTTGGTTGGGGTGACCAAGGGGAAAAACAAATCCCCCTCATCGATTGAGTACTAAGTACTTAAAA
ATTAGAATGACAATTCTAATTAATAAAACATTTATCGAAAAATGACCCAGGATTTCCTGATCAATGAACCAAGTTACCCTAGGGAT
AACAGCGCAATCCTTTCTCAGAGTCCCTATCGAAGAAAGGGTTTACGACCTCGATGTTGGATCAGGACATCCTAATGGTGCAAC
CGCTATTAAGGGTTCGTT

No.2025-166-26-49

CCGCCTGCCCTGTGACAACGTTCAACGGCCGCGGTATTTTGACCGTGCAAAGGTAGCGTAATCACTTGTCTTTTAAATGAAGAC
CTGTATGAAAGGCATCACGAGAGTTTAACTGTCTCTATTTTCTAATCAATGAAATTGATCTATTCGTGCAGAAGCGAATATAATAA
CATTAGACGAGAAGACCCTATGGAGCTTCAAACACTTAAATTAATTATGTAATCCTCCACCTCCCAGGGAATAAACAAAATATACA
ATACTTCTAATTTAACTGTTTTTGGTTGGGGTGACCAAGGGGAAAAACAAATCCCCCTCATCGATTGAGTACTAAGTACTTAAAA
ATTAGAATGACAATTCTAATTAATAAAACATTTATCGAAAAATGACCCAGGATTTCCTGATCAATGAACCAAGTTACCCTAGGGAT
AACAGCGCAATCCTTTCTCAGAGTCCCTATCGAAGAAAGGGTTTACGACCTCGATGTTGGATCAGGACATCCTAATGGTGCAAC
CGCTATTAAGGGTTCGTT

No.2025-166-26-50

CCGCCTGCCCTGTGACAATGTTTTAACGGCCGCGGTATTTTGACCGTGCAAAGGTAGCGTAATCACTTGTCTTTTAAATGAAGAC
CCGTATGAAAGGCATCACGAGAGTTTAACTGTCTCTATTTTCTAATCAATGAAATTGATCTACTCGTGCAGAAGCGAGTATAATCA
CATCAGACGAGAAGACCCTATGGAGCTTCAAACACATAAATTAACTACATAAATTAATTATTCCACGGATATAAATAAAAATACAG
TACCTTTAATTTAACTGTTTTTGGTTGGGGTGACCAAGGGGAAAAACAAATCCCCCTTATCGACTGAGTACTCAAGTACTTAAAA
ATTAGATTTACAATTCTAATTAATAAAATATTTATCGAACAATGACCCAGGATTTCCTGATCAATGAACCAAGTTACCCTAGGGATA
ACAGCGCAATCCTTTCTCAGAGTCCCTATCGCCGAAAGGGTTTACGACCTCGATGTTGGATCAGGACATCCTAATGATGCAACC
GTTATTAAGGGTTCGTT

No.2025-166-26-51

CCGCCTGCCCTGTGACAATGTTTAACGGCCGCGGTATTCTGACCGTGCAAAGGTAGCGTAATCACTTGTCTTTTAAATGAAGACC
CGTATGAAAGGCATCACGAGAGTTCAACTGTCTCTACTTTCCAATCAATGAAATTGATCCACCCGTGCAGAAGCGGGTATAAACA
CATCAGACGAGAAGACCCTATGGAGCTTCAAACACATGAATTAATTATGTAGACTAACTGCTCCACGGACATAAATAAAAATACA
ACACTTTTAATTTAACTGTTTTGGTTGGGGTGACCGAGGGGAAAAATCAATCCCCCTTATCGACCGAGTGTTCTCAAGCACTTAA
AAATTAGAATTACAATTCTAATTAGTAAAATATTTACCGAAAAATGACCCAGAATTTTCTGATCAATGAACCAAGTTACCCTAGGG
ATAACAGCGCAATCCTTTCCTAGAGTCCCTATCGACGAAAGGGTTTACGACCTCGATGTTGGATCAGGACATCCTAATGATGTAG
CCGTTATTAAGGGTTCGTT

No.2025-166-26-52

CGGCCGCGGTATTTTGACCGTGCAAAGGTAGCGTAATCACTTGTCTTTTAAATGAAGACCTGTATGAAAGGCATCACGAGAGTT
TAACTGTCTCTATTTTCTAATCAATGAAATTGATCTATTCGTGCAGAAGCGAATATAATAACATTAGACGAGAAGACCCTATGGAG
CTTCAAACACTTAAATTAATTATGTAATCCTCCACCTCCCAGGGAATAAACAAAATATACAATACTTCTAATTTAACTGTTTTTGGTT
GGGGTGACCAAGGGGAAAAACAAATCCCCCTCATCGATTGAGTACTAAGTACTTAAAAATTAGAATGACAATTCTAATTAATAAA
ACATTTATCGAAAAATGACCCAGGATTTCCTGATCAATGAACCAAGTTACCCTAGGGATAACAGCGCAATCCTTTCTCAGAGTCC
CTATCGAAGAAAGGGTTTACGACCTCGATGTTGGATCAGGACATCCTAATGGTGCAACCGCTATTAAGGGTTCGTTTGTTCAAC
GATTAACAGTCCTACGT

No.2025-166-26-53

CCGCCTGCCCTGTGACAACGTTCAACGGCCGCGGTATTTTGACCGTGCAAAGGTAGCGTAATCACTTGTCTTTTAAATGAAGAC
CTGTATGAAAGGCATCACGAGAGTTTAACTGTCTCTATTTTCTAATCAATGAAATTGATCTATTCGTGCAGAAGCGAATATAATAA
CATTAGACGAGAAGACCCTATGGAGCTTCAAACACTTAAATTAATTATGTAATCCTCCACCTCCCAGGGAATAAACAAAATATACA
ATACTTCTAATTTAACTGTTTTTGGTTGGGGTGACCAAGGGGAAAAACAAATCCCCCTCATCGATTGAGTACTAAGTACTTAAAA
ATTAGAATGACAATTCTAATTAATAAAACATTTATCGAAAAATGACCCAGGATTTCCTGATCAATGAACCAAGTTACCCTAGGGAT
AACAGCGCAATCCTTTCTCAGAGTCCCTATCGAAGAAAGGGTTTACGACCTCGATGTTGGATCAGGACATCCTAATGGTGCAAC
CGCTATTAAGGGTTCGTT

No.2025-166-26-54

CCGCCTGCCCTGTGACAACGTTCAACGGCCGCGGTATTTTGACCGTGCAAAGGTAGCGTAATCACTTGTCTTTTAAATGAAGAC
CTGTATGAAAGGCATCACGAGAGTTTAACTGTCTCTATTTTCTAATCAATGAAATTGATCTATTCGTGCAGAAGCGAATATAATAA
CATTAGACGAGAAGACCCTATGGAGCTTCAAACACTTAAATTAATTATGTAATCCTCCACCTCCCAGGGAATAAACAAAATATACA
ATACTTCTAATTTAACTGTTTTTGGTTGGGGTGACCAAGGGGAAAAACAAATCCCCCTCATCGATTGAGTACTAAGTACTTAAAA
ATTAGAATGACAATTCTAATTAATAAAACATTTATCGAAAAATGACCCAGGATTTCCTGATCAATGAACCAAGTTACCCTAGGGAT
AACAGCGCAATCCTTTCTCAGAGTCCCTATCGAAGAAAGGGTTTACGACCTCGATGTTGGATCAGGACATCCTAATGGTGCAAC
CGCTATTAAGGGTTCGT

No.2025-166-26-55

CCGCCTGCCCTGTGACAACGTTCAACGGCCGCGGTATTTTGACCGTGCAAAGGTAGCGTAATCACTTGTCTTTTAAATGAAGAC
CTGTATGAAAGGCATCACGAGAGTTTAACTGTCTCTATTTTCTAATCAATGAAATTGATCTATTCGTGCAGAAGCGAATATAATAA
CATTAGACGAGAAGACCCTATGGAGCTTCAAACACTTAAATTAATTATGTAATCCTCCACCTCCCAGGGAATAAACAAAATATACA
ATACTTCTAATTTAACTGTTTTTGGTTGGGGTGACCAAGGGGAAAAACAAATCCCCCTCATCGATTGAGTACTAAGTACTTAAAA
ATTAGAATGACAATTCTAATTAATAAAACATTTATCGAAAAATGACCCAGGATTTCCTGATCAATGAACCAAGTTACCCTAGGGAT
AACAGCGCAATCCTTTCTCAGAGTCCCTATCGAAGAAAGGGTTTACGACCTCGATGTTGGATCAGGACATCCTAATGGTGCAAC
CGCTATTAAGGGTTCGT

No.2025-166-26-56

CCGCCTGCCCTGTGACAACGTTCAACGGCCGCGGTATTTTGACCGTGCAAAGGTAGCGTAATCACTTGTCTTTTAAATGAAGAC
CTGTATGAAAGGCATCACGAGAGTTTAACTGTCTCTATTTTCTAATCAATGAAATTGATCTATTCGTGCAGAAGCGAATATAATAA
CATTAGACGAGAAGACCCTATGGAGCTTCAAACACTTAAATTAATTATGTAATCCTCCACCTCCCAGGGAATAAACAAAATATACA
ATACTTCTAATTTAACTGTTTTTGGTTGGGGTGACCAAGGGGAAAAACAAATCCCCCTCATCGATTGAGTACTAAGTACTTAAAA
ATTAGAATGACAATTCTAATTAATAAAACATTTATCGAAAAATGACCCAGGATTTCCTGATCAATGAACCAAGTTACCCTAGGGAT
AACAGCGCAATCCTTTCTCAGAGTCCCTATCGAAGAAAGGGTTTACGACCTCGATGTTGGATCAGGACATCCTAATGGTGCAAC
CGCTATTAAGGGTTCGTT

No.2025-166-26-57

CCGCCTGCCCTGTGACAACGTTCAACGGCCGCGGTATTTTGACCGTGCAAAGGTAGCGTAATCACTTGTCTTTTAAATGAAGAC
CTGTATGAAAGGCATCACGAGAGTTTAACTGTCTCTATTTTCTAATCAATGAAATTGATCTATTCGTGCAGAAGCGAATATAATAA
CATTAGACGAGAAGACCCTATGGAGCTTCAAACACTTAAATTAATTATGTAATCCTCCACCTCCCAGGGAATAAACAAAATATACA
ATACTTCTAATTTAACTGTTTTTGGTTGGGGTGACCAAGGGGAAAAACAAATCCCCCTCATCGATTGAGTACTAAGTACTTAAAA
ATTAGAATGACAATTCTAATTAATAAAACATTTATCGAAAAATGACCCAGGATTTCCTGATCAATGAACCAAGTTACCCTAGGGAT
AACAGCGCAATCCTTTCTCAGAGTCCCTATCGAAGAAAGGGTTTACGACCTCGATGTTGGATCAGGACATCCTAATGGTGTAAC
CGCTATTAAGGGTTCGTT

No.2025-166-26-58

CCCTGTGACAACGTTCAACGGCCGCGGTAATTTGACCGTGCAAAGGTAGCGTAATCACTTGTCTTTTAAATGAAGACCTGTATG
AAAGGCATCACGAGAGTTTAACTGTCTCTATTTTCTAATCAATGAAATTGATCTATTCGTGCAGAAGCGAATATAATAACATTAGA
CGAGAAGACCCTATGGAGCTTCAAACACTTAAATTAATTATGTAATCCTCCACCTCCCAGGGAATAAACAAAATATACAATACTTC
TAATTTAACTGTTTTTGGTTGGGGTGACCAAGGGGAAAAACAAATCCCCCTCATCGATTGAGTACTAAGTACTTAAAAATTAGAA
TGACAATTCTAATTAATAAAACATTTATCGAAAAATGACCCAGGATTTCCTGATCAATGAACCAAGTTACCCTAGGGATAACAGC
GCAATCCTTTCTCAGAGTCCCTATCGAAGAAAGGGTTTACGACCTCGATGTTGGATCAGGACATCCTAATGGTGCAACCGCTATT
AAGGGTTCGTT

No.2025-166-26-59

AACGTTCAACGGCCGCGGTATTTTGACCGTGCAAAGGTAGCGTAATCACTTGTCTTTTAAATGAAGACCTGTATGAAAGGCATC
ACGAGAGTTTAACTGTCTCTATTTTCTAATCAATGAAATTGATCTATTCGTGCAGAAGCGAATATAATAACATTAGACGAGAAGAC
CCTATGGAGCTTCAAACACTTAAATTAATTATGTAATCCTCCACCTCCCAGGGAATAAACAAAATATACAATACTTCTAATTTAACT
GTTTTTGGTTGGGGTGACCAAGGGGAAAAACAAATCCCCCTCATCGATTGAGTACTAAGTACTTAAAAATTAGAATGACAATTC
TAATTAATAAAACATTTATCGAAAAATGACCCAGGATTTCCTGATCAATGAACCAAGTTACCCTAGGGATAACAGCGCAATCCTTT
CTCAGAGTCCCTATCGAAGAAAGGGTTTACGACCTCGATGTTGGATCAGGACATCCTAATGGTGCAACCGCTATTAAGGGTTCG
TTTGTTCAACGATTAACAGTCCTACGTGATC

No.2025-166-26-60

CTGCCCTGTGACAATGTTTAACGGCCGCGGTATTCTGACCGTGCAAAGGTAGCGTAATCACTTGTCTTTTAAATGAAGACCCGTA
TGAAAGGCATCACGAGAGTTCAACTGTCTCTACTTTCCAATCAATGAAATTGATCCACCCGTGCAGAAGCGGGTATAAACACATC
AGACGAGAAGACCCTATGGAGCTTCAAACACATGAATTAATTATGTAGACTAACTGCTCCACGGACATAAATAAAAATACAACAC
TTTTAATTTAACTGTTTTGGTTGGGGTGACCGAGGGGAAAAATCAATCCCCCTTATCGACCGAGTGTTCTCAAGCACTTAAAAAT
TAGAATTACAATTCTAATTAGTAAAATATTTACCGAAAAATGACCCAGAATTTTCTGATCAATGAACCAAGTTACCCTAGGGATAA
CAGCGCAATCCTTTCCTAGAGTCCCTATCGACGAAAGGGTTTACGACCTCGATGTTGGATCAGGACATCCTAATGATGTAGCCGT
TATTAAGGGTTCGTT

No.2025-166-26-61

CCGCCTGCCCTGTGACAATGTTTTAACGGCCGCGGTATTTTGACCGTGCAAAGGTAGCGTAATCACTTGTCTTTTAAATGAAGAC
CCGTATGAAAGGCATCACGAGAGTTCAACTGTCTCTATTTTCTAATCAATGAAATTGATCTACTCGTGCAGAAGCGAGTATAACTA
CATTAGACGAGAAGACCCTATGGAGCTTCAAACACATGAATTAAATATGTAAACTAACTACTCCCCGGACATAAATAAAATAATAT
TTTTAATTTAACTGTTTTTGGTTGGGGTGACCAAGGGGAAAAATAAATCCCCCTTATCGATTGAGTACTCAAGTACTTAAAAATC
AGAATTACAATTCTGATTAATAAAATATTTATCGAAAAATGACCCAGGATTTCCTGATCAATGAACCAAGTTACCCTAGGGATAAC
AGCGCAATCCTTTCTTAGAGTCCCTATCGCCGAAAGGGTTTACGACCTCGATGTTGGATCAGGACATCCTAATGATGCAACCGTT
ATTAAGGGTTCGTT

No.2025-166-26-62

GTTCAACGGCCGCGGTATTTTGACCGTGCAAAGGTAGCGTAATCACTTGTCTTTTAAATGAAGACCTGTATGAAAGGCATCACG
AGAGTTTAACTGTCTCTATTTTCTAATCAATGAAATTGATCTATTCGTGCAGAAGCGAATATAATAACATTAGACGAGAAGACCCT
ATGGAGCTTCAAACACTTAAATTAATTATGTAATCCTCCACCTCCCAGGGAATAAACAAAATATACAATACTTCTAATTTAACTGTT
TTTGGTTGGGGTGACCAAGGGGAAAAACAAATCCCCCTCATCGATTGAGTACTAAGTACTTAAAAATTAGAATGACAATTCTAAT
TAATAAAACATTTATCGAAAAATGACCCAGGATTTCCTGATCAATGAACCAAGTTACCCTAGGGATAACAGCGCAATCCTTTCTCA
GAGTCCCTATCGAAGAAAGGGTTTACGACCTCGATGTTGGATCAGGACATCCTAATGGTGCAACCGCTATTAAGGGTTCGTTTG
TTCAACGATTAACAGTCCTACGTGATCT

No.2025-166-26-63

CCCTGTGACAATGTTTTAACGGCCGCGGTATTTTGACCGTGCAAAGGTAGCGTAATCACTTGTCTTTTAAATGAAGACCCGTATG
AAAGGCATCACGAGAGTTTAACTGTCTCTATTTTCTAATCAATGAAATTGATCTACTCGTGCAGAAGCGAGTATAATCACATCAGA
CGAGAAGACCCTATGGAGCTTCAAACACATAAATTAACTACATAAATTAATTATTCCACGGATATAAATAAAAATACAGTACCTTTA
ATTTAACTGTTTTTGGTTGGGGTGACCAAGGGGAAAAACAAATCCCCCTTATCGACTGAGTACTCAAGTACTTAAAAATTAGATT
TACAATTCTAATTAATAAAATATTTATCGAACAATGACCCAGGATTTCCTGATCAATGAACCAAGTTACCCTAGGGATAACAGCGC
AATCCTTTCTCAGAGTCCCTATCGCCGAAAGGGTTTACGACCTCGATGTTGGATCAGGACATCCT-AATGATGCAACCGTTATTAA
GGGTTCGTT

No.2025-166-26-64

CCGCCTGCCCTGTGACAATGTTTTAACGGCCGCGGTATTTTGACCGTGCAAAGGTAGCGTAATCACTTGTCTTTTAAATGAAGAC
CCGTATGAAAGGCATCACGAGAGTTCAACTGTCTCTATTTTCTAATCAATGAAATTGATCTACTCGTGCAGAAGCGAGTATAACTA
CATTAGACGAGAAGACCCTATGGAGCTTCAAACACATGAATTAAATATGTAAACTAACTACTCCCCGGACATAAATAAAATAATAT
TTTTAATTTAACTGTTTTTGGTTGGGGTGACCAAGGGGAAAAATAAATCCCCCTTATCGATTGAGTACTCAAGTACTTAAAAATC
AGAATTACAATTCTGATTAATAAAATATTTATCGAAAAATGACCCAGGATTTCCTGATCAATGAACCAAGTTACCCTAGGGATAAC
AGCGCAATCCTTTCTTAGAGTCCCTATCGCCGAAAGGGTTTACGACCTCGATGTTGGATCAGGACATCCTAATGATGCAACCGTT
ATTAAGGGTTCGTT

Shark fin: COI gene - Large-sized samples (n=64)

No.2025-166-27-1

TGAGCAGGAATAGTGGGACAGCCCTCAGCCTTCTAATTCGAGCCGAGTTAGGCCAGCCCGGATCACTCCTAGGGGATGATCAG
GTCTATAATGTTATCGTAACCGCCCATGCATTTGTAATAATCTTCTTCATGGTTATACCCGTAATAATTGGGGGATTTGGAAACTGA
TTAGTACCCTTAATAATTGGTGCACCAGACATGGCCTTCCCGCGAATAAATAACATAAGCTTTTGACTCCTTCCCCCTTCTTTTCTC
TTACTCCTAGCTTCAGCTGGGGTTGAAGCTGGAGCTGGCACTGGTTGAACAGTTTATCCCCCCTTAGCTGGCAACTTAGCACAT
GCTGGGGCATCTGTTGACTTGGCCATTTTCTCGCTTCATTTAGCAGGTATCTCATCAATTTTAGCTTCAATTAACTTTATTACAACT
ATCATTAATATAAAACCACCAGCCATCTCTCAATATCAAACACCATTATTTGTATGATCAATCCTAGTAACAACCATCCTCCTCCTCTT
ATCCCTCCCAGTACTCGCAGCCGGCATCACAATAT

No.2025-166-27-2

GCCCTCAGCCTTCTAATTCGAGCCGAGTTAGGCCAGCCCGGATCACTCCTAGGGGATGATCAGGTCTATAATGTTATCGTAACCG
CCCATGCATTTGTAATAATCTTCTTCATGGTTATACCCGTAATAATTGGGGGATTTGGAAACTGATTAGTACCCTTAATAATTGGTG
CACCAGACATGGCCTTCCCGCGAATAAATAACATAAGCTTTTGACTCCTTCCCCCTTCTTTTCTCTTACTCCTAGCTTCAGCTGGG
GTTGAAGCTGGAGCTGGCACTGGTTGAACAGTTTATCCCCCCTTAGCTGGCAACTTAGCACATGCTGGGGCATCTGTTGACTTG
GCCATTTTCTCGCTTCATTTAGCAGGTATCTCATCAATTTTAGCTTCAATTAACTTTATTACAACTATCATTAATATAAAACCACCAG
CCATCTCTCAATATCAAACACCATTATTTGTATGATCAATCCTAGTAACAACCATCCTCCTCCTCTTATCCCTCCCAGTACTCGCAGC
CGGCATCACAATATTATTAACTGATCGAAACCTAAACACAACATTCTTT

No.2025-166-27-3

GCCCTAAGCCTTTTAATTTCGTGCCGAACTGGGTCAGCCTGGTTCCCTCCTAGGGGATGATCAGATTTATAATGTTATTGTAACCG
CCCATGCATTTGTAATAATTTTCTTTATGGTTATGCCCGTAATAATTGGAGGCTTTGGAAATTGACTAGTGCCTTTAATGATCGGAG
CACCAGACATAGCCTTCCCCCGAATAAATAACATAAGTTTCTGGCTCCTACCCCCTTCTTTCCTTTTACTCTTAGCCTCAGCCGGA
GTTGAGTCAGGAGCCGGCACTGGTTGAACAGTCTACCCTCCCCTAGCTGGCAACTTAGCACACGCCGGAGCATCTGTTGATCTA
GCCATTTTCTCCCTCCACCTGGCTGGTATCTCGTCCATCCTAGCTTCCATTAACTTCATTACAACCATCATCAACATAAAACCCCCA
GCAATCTCCCAATACCAAACACCCCTGTTTGTCTGGTCCATTCTAGTGACAACCATCCTCCTTCTTTTAGCACTCCCAGTGCTCGC
CGCTGGCATTACAATACTACTTACGGACCGAAACCTAAACACAACATTC

No.2025-166-27-4

ATGAGCAGGAATAGTGGGACAGCCCTCAGCCTTCTAATTCGAGCCGAGTTAGGCCAGCCCGGATCACTCCTAGGGGATGATCA
GGTCTATAATGTTATCGTAACCGCCCATGCATTTGTAATAATCTTCTTCATGGTTATACCCGTAATAATTGGGGGATTTGGAAACTG
ATTAGTACCCTTAATAATTGGTGCACCAGACATGGCCTTCCCGCGAATAAATAACATAAGCTTTTGACTCCTTCCCCCTTCCTTTCT
CTTACTCCTAGCTTCAGCTGGGGTTGAAGCTGGAGCTGGCACTGGTTGAACAGTTTATCCCCCCTTAGCTGGCAACTTAGCACA
TGCTGGGGCATCTGTTGACTTGGCCATTTTCTCGCTTCATTTAGCAGGTATCTCATCAATTTTAGCTTCAATTAACTTTATTACAAC
TATCATTAATATAAAACCACCAGCCATCTCTCAATATCAAACACCATTATTTGTATGATCAATCCTAGTAACAACCATCCTCCTCCTCT
TATCCCTCCCAGTACTCGCAGCCGGCATCACAATATTAT

No.2025-166-27-5

AGCAGGTATCGTTGGAACAGCCCTAAGTCTTCTAATTCGAGCTGAGCTTGGACAACCTGGATCACTTTTAGGGGATGATCAGAT
TTATAATGTAATCGTAACCGCCCACGCTTTTGTAATAATCTTTTTTATGGTTATGCCAATCATAATTGGTGGTTTCGGAAATTGACTA
GTTCCTTTAATAATTGGTGCACCAGATATAGCCTTCCCACGAATAAATAACATAAGTTTCTGACTTCTTCCACCATCATTTCTTCTTC
TCCTCGCCTCTGCTGGAGTAGAAGCTGGAGCAGGTACTGGTTGAACAGTTTATCCTCCATTAGCTAGTAACCTAGCACATGCTG
GACCATCTGTTGATTTAGCTATTTTCTCTCTTCACTTAGCCGGTGTGTCATCTATTCTAGCTTCAATTAATTTTATTACAACTATTATC
AATATAAAACCACCAGCCATTTCCCAATATCAAACACCATTATTTGTTTGATCTATTCTTGTAACCACTATTCTTCTCCTCCTATCACT
TCCAGTTCTTGCAGCAGGGATTACAATATTACTTACAGATC

No.2025-166-27-6

TTGGAAGAGCCCTTCAGTCTTCTAATTCGAGCTGAGCTTGGACAACCTGGATCACTTTTAGGGGATGATCAGATTTATAATGTAA
TCGTAACCGCCCACGCTTTTGTAATAATCTTTTTTATGGTTATGCCAATCATAATTGGTGGTTTCGGAAATTGACTAGTTCCTTTAA
TAATTGGTGCACCAGATATAGCCTTCCCACGAATAAATAACATAAGTTTCTGACTTCTTCCACCATCATTTCTTCTTCTCCTCGCCTC
TGCTGGAGTAGAAGCTGGAGCAGGTACTGGTTGAACAGTTTATCCTCCATTAGCTAGTAACCTAGCACATGCTGGACCATCTGTT
GATTTAGCTATTTTCTCTCTTCACTTAGCCGGTGTGTCATCTATTCTAGCTTCAATTAATTTTATTACAACTATTATCAATATAAAACC
ACCAGCCATTTCCCAATATCAAACACCATTATTTGTTTGATCTATTCTTGTAACCACTATTCTTCTCCTCCTATCACTTCCAGTTCTTG
CAGCAGGGATTACAATATTACTTACAGATCG

No.2025-166-27-7

GCCCTAAGTCTTCTAATTCGAGCTGAGCTTGGACAACCTGGATCACTTTTAGGGGATGATCAGATTTATAATGTAATCGTAACCG
CCCACGCTTTTGTAATAATCTTTTTTATGGTTATGCCAATCATAATTGGTGGTTTCGGAAATTGACTAGTTCCTTTAATAATTGGTG
CACCAGATATAGCCTTCCCACGAATAAATAACATAAGTTTCTGACTTCTTCCACCATCATTTCTTCTTCTCCTCGCCTCTGCTGGAG
TAGAAGCTGGAGCAGGTACTGGTTGAACAGTTTATCCTCCATTAGCTAGTAACCTAGCACATGCTGGACCATCTGTTGATTTAGC
TATTTTCTCTCTTCACTTAGCCGGTGTGTCATCTATTCTAGCTTCAATTAATTTTATTACAACTATTATCAATATAAAACCACCAGCCA
TTTCCCAATATCAAACACCATTATTTGTTTGATCTATTCTTGTAACCACTATTCTTCTCCTCCTATCACTTCCAGTTCTTGCAGCAGG
GATTACAATATTACT

No.2025-166-27-8

TGCATGAGCAGGTATAGTTGGAACAGCCCTAAGTCTTCTAATTCGAGCTGAGCTTGGACAACCTGGATCACTTTTAGGGGATGA
TCAGATTTATAATGTAATCGTAACCGCCCACGCTTTTGTAATAATCTTTTTTATGGTTATGCCAATCATAATTGGTGGTTTCGGAAAT
TGACTAGTTCCTTTAATAATTGGTGCACCAGATATAGCCTTCCCACGAATAAATAACATAAGTTTCTGACTTCTTCCACCATCATTT

CTTCTTCTCCTCGCCTCTGCTGGAGTAGAAGCTGGAGCAGGTACTGGTTGAACAGTTTATCCTCCATTAGCTAGTAACCTAGCAC
ATGCTGGACCATCTGTTGATTTAGCTATTTTCTCTCTTCACTTAGCCGGTGTGTCATCTATTCTAGCTTCAATTAATTTTATTACAACT
ATTATCAATATAAAACCACCAGCCATTTCCCAATATCAAACACCATTATTTGTTTGATCTATTCTTGTAACCACTATTCTTCTCCTCCT
ATCACTTCCAGTTCTTGCAGCAGGGATTACAATATTACTTACAGATCGTA

No.2025-166-27-9

TGAGCAGGAATAGTGGGAACAGCCCTCAGCCTTCTAATTCGAGCCGAGTTAGGCCAGCCCGGATCACTCCTAGGGGATGATCA
GGTCTATAATGTTATCGTAACCGCCCATGCATTTGTAATAATCTTCTTCATGGTTATACCCGTAATAATTGGGGGATTTGGAAACTG
ATTAGTACCCTTAATAATTGGTGCACCAGACATGGCCTTCCCGCGAATAAATAACATAAGCTTTTGACTCCTTCCCCCTTCTTTTCT
CTTACTCCTAGCTTCAGCTGGGGTTGAAGCTGGAGCTGGCACTGGTTGAACAGTTTATCCCCCCTTAGCTGGCAACTTAGCACA
TGCTGGGGCATCTGTTGACTTGGCCATTTTCTCGCTTCATTTAGCAGGTATCTCATCAATTTTAGCTTCAATTAACTTTATTACAAC
TATCATTAATATAAAACCACCAGCCATCTCTCAATATCAAACACCATTATTTGTATGATCAATCCTAGTAACAACCATCCTCCTCCTCT
TATCCCTCCCAGTACTCGCAGCCGGCATCACAATAT

No.2025-166-27-10

ATGAGCAGGAATAGTGGGGACGGCCCTAAGCCTCTTAATTCGAGCCGAATTAGGACAGCCAGGATCACTTCTAGGAGATGATC
AAATCTATAATGTTATTGTAACCGCCCATGCATTCGTAATAATCTTCTTTATAGTTATACCCGTAATAATTGGCGGATTTGGAAACTG
ACTAGTGCCATTAATAATTGGTGCACCAGACATAGCTTTTCCACGAATAAATAATATAAGCTTTTGACTCCTTCCCCCCTCTTTTCT
TTTACTTCTAGCTTCAGCTGGAGTTGAAGCCGGAGCCGGTACTGGTTGAACAGTTTATCCTCCATTAGCTGGCAATTTAGCACAT
GCTGGGGCATCCGTTGACTTAGCTATTTTCTCTCTCCATTTAGCAGGTATTTCATCAATTTTAGCCTCAATCAACTTTATTACAACTA
TTATTAATATAAAACCCCCTGCAATCTCCCAATATCAAACACCACTATTTGTGTGATCAATTCTAGTAACAACTATCCTCCTTCTATTA
TCCCTCCCAGTACTTGCAGCCGGCATTACAATACTACTTACTGATCGA

No.2025-166-27-11

ATGAGCAGGAATAGTGGGACAGCCCTCAGCCTTCTAATTCGAGCCGAGTTAGGCCAGCCCGGATCACTCCTAGGGGATGATCA
GGTCTATAATGTTATCGTAACCGCCCATGCATTTGTAATAATCTTCTTCATGGTTATACCCGTAATAATTGGGGGATTTGGAAACTG
ATTAGTACCCTTAATAATTGGTGCACCAGACATGGCCTTCCCGCGAATAAATAACATAAGCTTTTGACTCCTTCCCCCTTCTTTTCT
CTTACTCCTAGCTTCAGCTGGGGTTGAAGCTGGAGCTGGCACTGGTTGAACAGTTTATCCCCCCTTAGCTGGCAACTTAGCACA
TGCTGGGGCATCTGTTGACTTGGCCATTTTCTCGCTTCATTTAGCAGGTATCTCATCAATTTTAGCTTCAATTAACTTTATTACAAC
TATCATTAATATAAAACCACCAGCCATCTCTCAATATCAAACACCATTATTTGTATGATCAATCCTAGTAACAACCATCCTCCTCCTCT
TATCCCTCCCAGTACTCGCAGCCGGCATCACAATAT

No.2025-166-27-12

GTGCATGAGCAGGAATAGTGGGGACAGCCCTAAGCCTCTTAATTCGAGCCGAATTAGGACAGCCAGGATCACTTCTAGGAGAT
GATCAAATCTATAATGTTATTGTAACCGCCCATGCATTCGTAATAATCTTCTTTATAGTTATACCCGTAATAATTGGCGGATTTGGAA
ACTGACTAGTGCCATTAATAATTGGTGCACCAGACATAGCTTTTCCACGAATAAATAATATAAGCTTTTGACTCCTTCCCCCCTCTT
TTCTTTTACTTCTAGCTTCAGCTGGAGTTGAAGCCGGAGCCGGTACTGGTTGAACAGTTTATCCTCCATTAGCTGGCAATTTAGC
ACATGCTGGGGCATCCGTTGACTTAGCTATTTTCTCTCTCCATTTAGCAGGTATTTCATCAATTTTAGCCTCAATCAACTTTATTACA
ACTATTATTAATATAAAACCCCCTGCAATCTCCCAATATCAAACACCACTATTTGTGTGATCAATTCTAGTAACAACTATCCTCCTTCT
ATTATCCCTCCCAGTACTTGCAGCCGGCATTACAATACTACTTACT

No.2025-166-27-13

TGAGCAGGAATAGTGGGGACGGCCCTAAGCCTCTTAATTCGAGCCGAATTAGGACAGCCAGGATCACTTCTAGGAGATGATCA
AATCTATAATGTTATTGTAACCGCCCATGCATTCGTAATAATCTTCTTTATAGTTATACCCGTAATAATTGGCGGATTTGGAAACTGA
CTAGTGCCATTAATAATTGGTGCACCAGACATAGCTTTTCCACGAATAAATAATATAAGCTTTTGACTCCTTCCCCCCTCTTTTCTT
TTACTTCTAGCTTCAGCTGGAGTTGAAGCCGGAGCCGGTACTGGTTGAACAGTTTATCCTCCATTAGCTGGCAATTTAGCACATG
CTGGGGCATCCGTTGACTTAGCTATTTTCTCTCTCCATTTAGCAGGTATTTCATCAATTTTAGCCTCAATCAACTTTATTACAACTAT
TATTAATATAAAACCCCCTGCAATCTCCCAATATCAAACACCACTATTTGTGTGATCAATTCTAGTAACAACTATCCTCCTTCTATTAT
CCCTCCCAGTACTTGCAGCCGGCATTACAATACTACT

No.2025-166-27-14

TAAGCCTCTTAATTCGAGCCGAATTAGGACAGCCAGGATCACTTCTAGGAGATGATCAAATCTATAATGTTATTGTAACCGCCCAT
GCATTCGTAATAATCTTCTTTATAGTTATACCCGTAATAATTGGCGGATTTGGAAACTGACTAGTGCCATTAATAATTGGTGCACCA
GACATAGCTTTTCCACGAATAAATAATATAAGCTTTTGACTCCTTCCCCCCTCTTTTCTTTTACTTCTAGCTTCAGCTGGAGTTGAA
GCCGGAGCCGGTACTGGTTGAACAGTTTATCCTCCATTAGCTGGCAATTTAGCACATGCTGGGGCATCCGTTGACTTAGCTATTT
TCTCTCTCCATTTAGCAGGTATTTCATCAATTTTAGCCTCAATCAACTTTATTACAACTATTATTAATATAAAACCCCCTGCAATCTCC
CAATATCAAACACCACTATTTGTGTGATCAATTCTAGTAACAACTATCCTCCTTCTATTATCCCTCCCAGTACTTGCAGCCGGCATTA
CAATACTACTTACTGATCGAAACCTAAACACAACA

No.2025-166-27-15

ATGAGCAGGTATCGTTGGAACAGCCCTAAGTCTTCTAATTCGAGCTGAGCTTGGACAACCTGGATCACTTTTAGGGGATGATCA
GATTTATAATGTAATCGTAACCGCCCACGCTTTTGTAATAATCTTTTTTATGGTTATGCCAATCATAATTGGTGGTTTCGGAAATTG
ACTAGTTCCTTTAATAATTGGTGCACCAGATATAGCCTTCCCACGAATAAATAACATAAGTTTCTGACTTCTTCCACCATCATTTCTT
CTTCTCCTCGCCTCTGCTGGAGTAGAAGCTGGAGCAGGTACTGGTTGAACAGTTTATCCTCCATTAGCTAGTAACCTAGCACATG
CTGGACCATCTGTTGATTTAGCTATTTTCTCTCTTCACTTAGCCGGTGTGTCATCTATTCTAGCTTCAATTAATTTTATTACAACTATT
ATCAATATAAAACCACCAGCCATTTCCCAATATCAAACACCATTATTTGTTTGATCTATTCTTGTAACCACTATTCTTCTCCTCCTATC
ACTTCCAGTTCTTGCAGCAGGGATTACAATATTACTTACAGATCGT

No.2025-166-27-16

GGTGCATGAGCAGGAATAGTGGGGACGGCCCTAAGCCTCTTAATTCGAGCCGAATTAGGACAGCCAGGATCACTTCTAGGAGA
TGATCAAATCTATAATGTTATTGTAACCGCCCATGCATTCGTAATAATCTTCTTTATAGTTATACCCGTAATAATTGGCGGATTTGGA

AACTGACTAGTGCCATTAATAATTGGTGCACCAGACATAGCTTTTCCACGAATAAATAATATAAGCTTTTGACTCCTTCCCCCCTCT
TTTCTTTTACTTCTAGCTTCAGCTGGAGTTGAAGCCGGAGCCGGTACTGGTTGAACAGTTTATCCTCCATTAGCTGGCAATTTAG
CACATGCTGGGGCATCCGTTGACTTAGCTATTTTCTCTCTCCATTTAGCAGGTATTTCATCAATTTTAGCCTCAATCAACTTTATTAC
AACTATTATTAATATAAAACCCCCTGCAATCTCCCAATATCAAACACCACTATTTGTGTGATCAATTCTAGTAACAACTATCCTCCTT
CTATTATCCCTCCCAGTACTTGCAGCCGGCATTACAATACTACT

No.2025-166-27-17

TGAGCAGGAATAGTGGGAACAGCCCTCAGCCTTCTAATTCGAGCCGAGTTAGGCCAGCCCGGATCACTCCTAGGGGATGATCA
GGTCTATAATGTTATCGTAACCGCCCATGCATTTGTAATAATCTTCTTCATGGTTATACCCGTAATAATTGGGGGATTTGGAAACTG
ATTAGTACCCTTAATAATTGGTGCACCAGACATGGCCTTCCCGCGAATAAATAACATAAGCTTTTGACTCCTTCCCCCTTCTTTTCT
CTTACTCCTAGCTTCAGCTGGGGTTGAAGCTGGAGCTGGCACTGGTTGAACAGTTTATCCCCCCTTAGCTGGCAACTTAGCACA
TGCTGGGGCATCTGTTGACTTGGCCATTTTCTCGCTTCATTTAGCAGGTATCTCATCAATTTTAGCTTCAATTAACTTTATTACAAC
TATCATTAATATAAAACCACCAGCCATCTCTCAATATCAAACACCATTATTTGTATGATCAATCCTAGTAACAACCATCCTCCTCCTCT
TATCCCTCCCAGTACTCGCAGCCGGCATCACAATATTAT

No.2025-166-27-18

TGAGCAGGTATAGTTGGAACAGCCCTAAGTCTTCTAATTCGAGCTGAGCTTGGACAACCTGGATCACTTTTAGGGGATGATCAG
ATTTATAATGTAATCGTAACCGCCCACGCTTTTGTAATAATCTTTTTTATGGTTATGCCAATCATAATTGGTGGTTTCGGAAATTGAC
TAGTTCCTTTAATAATTGGTGCACCAGATATAGCCTTCCCACGAATAAATAACATAAGTTTCTGACTTCTTCCACCATCATTTCTTCT
TCTCCTCGCCTCTGCTGGAGTAGAAGCTGGAGCAGGTACTGGTTGAACAGTTTATCCTCCATTAGCTAGTAACCTAGCACATGCT
GGACCATCTGTTGATTTAGCTATTTTCTCTCTTCACTTAGCCGGTGTGTCATCTATTCTAGCTTCAATTAATTTTATTACAACTATTAT
CAATATAAAACCACCAGCCATTTCCCAATATCAAACACCATTATTTGTTTGATCTATTCTTGTAACCACTATTCTTCTCCTCCTATCAC
TTCCAGTTCTTGCAGCAGGGATTACAATATTACTTACAGATCG

No.2025-166-27-19

TGAGCAGGAATAGTGGGGACGGCCCTAAGCCTCTTAATTCGAGCCGAATTAGGACAGCCAGGATCACTTCTAGGAGATGATCA
AATCTATAATGTTATTGTAACCGCCCATGCATTCGTAATAATCTTCTTTATAGTTATACCCGTAATAATTGGCGGATTTGGAAACTGA
CTAGTGCCATTAATAATTGGTGCACCAGACATAGCTTTTCCACGAATAAATAATATAAGCTTTTGACTCCTTCCCCCCTCTTTTCTT
TTACTTCTAGCTTCAGCTGGAGTTGAAGCCGGAGCCGGTACTGGTTGAACAGTTTATCCTCCATTAGCTGGCAATTTAGCACATG
CTGGGGCATCCGTTGACTTAGCTATTTTCTCTCTCCATTTAGCAGGTATTTCATCAATTTTAGCCTCAATCAACTTTATTACAACTAT
TATTAATATAAAACCCCCTGCAATCTCCCAATATCAAACACCACTATTTGTGTGATCAATTCTAGTAACAACTATCCTCCTTCTATTAT
CCCTCCCAGTACTTGCAGCCGGCATTACAATACTACT

No.2025-166-27-20

TGAGCAGGAATAGTGGGGACGGCCCTAAGCCTCTTAATTCGAGCCGAATTAGGACAGCCAGGATCACTTCTAGGAGATGATCA
AATCTATAATGTTATTGTAACCGCCCATGCATTCGTAATAATCTTCTTTATAGTTATACCCGTAATAATTGGCGGATTTGGAAACTGA
CTAGTGCCATTAATAATTGGTGCACCAGACATAGCTTTTCCACGAATAAATAATATAAGCTTTTGACTCCTTCCCCCCTCTTTTCTT
TTACTTCTAGCTTCAGCTGGAGTTGAAGCCGGAGCCGGTACTGGTTGAACAGTTTATCCTCCATTAGCTGGCAATTTAGCACATG
CTGGGGCATCCGTTGACTTAGCTATTTTCTCTCTCCATTTAGCAGGTATTTCATCAATTTTAGCCTCAATCAACTTTATTACAACTAT
TATTAATATAAAACCCCCTGCAATCTCCCAATATCAAACACCACTATTTGTGTGATCAATTCTAGTAACAACTATCCTCCTTCTATTAT
CCCTCCCAGTACTTGCAGCCGGCATTACAATACTACT

No.2025-166-27-21

TGAGCAGGAATAGTGGGAACAGCCCTCAGCCTTCTAATTCGAGCCGAGTTAGGCCAGCCCGGATCACTCCTAGGGGATGATCA
GGTCTATAATGTTATCGTAACCGCCCATGCATTTGTAATAATCTTCTTCATGGTTATACCCGTAATAATTGGGGGATTTGGAAACTG
ATTAGTACCCTTAATAATTGGTGCACCAGACATGGCCTTCCCGCGAATAAATAACATAAGCTTTTGACTCCTTCCCCCTTCTTTTCT
CTTACTCCTAGCTTCAGCTGGGGTTGAAGCTGGAGCTGGCACTGGTTGAACAGTTTATCCCCCCTTAGCTGGCAACTTAGCACA
TGCTGGGGCATCTGTTGACTTGGCCATTTTCTCGCTTCATTTAGCAGGTATCTCATCAATTTTAGCTTCAATTAACTTTATTACAAC
TATCATTAATATAAAACCACCAGCCATCTCTCAATATCAAACACCATTATTTGTATGATCAATCCTAGTAACAACCATCCTCCTCCTCT
TATCCCTCCCAGTACTCGCAGCCGGCATCACAATATTAT

No.2025-166-27-22

TGAGCAGGAATAGTGGGGACGGCCCTAAGCCTCTTAATTCGAGCCGAATTAGGACAGCCAGGATCACTTCTAGGAGATGATCA
AATCTATAATGTTATTGTAACCGCCCATGCATTCGTAATAATCTTCTTTATAGTTATACCCGTAATAATTGGCGGATTTGGAAACTGA
CTAGTGCCATTAATAATTGGTGCACCAGACATAGCTTTTCCACGAATAAATAATATAAGCTTTTGACTCCTTCCCCCCTCTTTTCTT
TTACTTCTAGCTTCAGCTGGAGTTGAAGCCGGAGCCGGTACTGGTTGAACAGTTTATCCTCCATTAGCTGGCAATTTAGCACATG
CTGGGGCATCCGTTGACTTAGCTATTTTCTCTCTCCATTTAGCAGGTATTTCATCAATTTTAGCCTCAATCAACTTTATTACAACTAT
TATTAATATAAAACCCCCTGCAATCTCCCAATATCAAACACCACTATTTGTGTGATCAATTCTAGTAACAACTATCCTCCTTCTATTAT
CCCTCCCAGTACTTGCAGCCGGCATTACAATACTACT

No.2025-166-27-23

TGAGCAGGAATAGTGGGGACGGCCCTAAGCCTCTTAATTCGAGCCGAATTAGGACAGCCAGGATCACTTCTAGGAGATGATCA
AATCTATAATGTTATTGTAACCGCCCATGCATTCGTAATAATCTTCTTTATAGTTATACCCGTAATAATTGGCGGATTTGGAAACTGA
CTAGTGCCATTAATAATTGGTGCACCAGACATAGCTTTTCCACGAATAAATAATATAAGCTTTTGACTCCTTCCCCCCTCTTTTCTT
TTACTTCTAGCTTCAGCTGGAGTTGAAGCCGGAGCCGGTACTGGTTGAACAGTTTATCCTCCATTAGCTGGCAATTTAGCACATG
CTGGGGCATCCGTTGACTTAGCTATTTTCTCTCTCCATTTAGCAGGTATTTCATCAATTTTAGCCTCAATCAACTTTATTACAACTAT
TATTAATATAAAACCCCCTGCAATCTCCCAATATCAAACACCACTATTTGTGTGATCAATTCTAGTAACAACTATCCTCCTTCTATTAT
CCCTCCCAGTACTTGCAGCCGGCATTACAATACTACT

No.2025-166-27-24

TGAGCAGGAATAGTGGGAACAGCCCTCAGCCTTCTAATTCGAGCCGAGTTAGGCCAGCCCGGATCACTCCTAGGGGATGATCA

GGTCTATAATGTTATCGTAACCGCCCATGCATTTGTAATAATCTTCTTCATGGTTATACCCGTAATAATTGGGGGATTTGGAAACTG
ATTAGTACCCTTAATAATTGGTGCACCAGACATGGCCTTCCCGCGAATAAATAACATAAGCTTTTGACTCCTTCCCCCTTCTTTTCT
CTTACTCCTAGCTTCAGCTGGGGTTGAAGCTGGAGCTGGCACTGGTTGAACAGTTTATCCCCCCTTAGCTGGCAACTTAGCACA
TGCTGGGGCATCTGTTGACTTGGCCATTTTCTCGCTTCATTTAGCAGGTATCTCATCAATTTTAGCTTCAATTAACTTTATTACAAC
TATCATTAATATAAAACCACCAGCCATCTCTCAATATCAAACACCATTATTTGTATGATCAATCCTAGTAACAACCATCCTCCTCCTCT
TATCCCTCCCAGTACTCGCAGCCGGCATCACAATATTAT

No.2025-166-27-25

TGAGCAGGTATAGTTGGAACAGCCCTAAGTCTTCTAATTCGAGCTGAGCTTGGACAACCTGGATCACTTTTAGGGGATGATCAG
ATTTATAATGTAATCGTAACCGCCCACGCTTTTGTAATAATCTTTTTTATGGTTATGCCAATCATAATTGGTGGTTTCGGAAATTGAC
TAGTTCCTTTAATAATTGGTGCACCAGATATAGCCTTCCCACGAATAAATAACATAAGTTTCTGACTTCTTCCACCATCATTTCTTCT
TCTCCTCGCCTCTGCTGGAGTAGAAGCTGGAGCAGGTACTGGTTGAACAGTTTATCCTCCATTAGCTAGTAACCTAGCACATGCT
GGACCATCTGTTGATTTAGCTATTTTCTCTCTTCACTTAGCCGGTGTGTCATCTATTCTAGCTTCAATTAATTTTATTACAACTATTAT
CAATATAAAACCACCAGCCATTTCCCAATATCAAACACCATTATTTGTTTGATCTATTCTTGTAACCACTATTCTTCTCCTCCTATCAC
TTCCAGTTCTTGCAGCAGGGATTACAATATTACTTACAGATCG

No.2025-166-27-26

TGAGCAGGTATAGTTGGAACAGCCCTAAGTCTTCTAATTCGAGCTGAGCTTGGACAACCTGGATCACTTTTAGGGGATGATCAG
ATTTATAATGTAATCGTAACCGCCCACGCTTTTGTAATAATCTTTTTTATGGTTATGCCAATCATAATTGGTGGTTTCGGAAATTGAC
TAGTTCCTTTAATAATTGGTGCACCAGATATAGCCTTCCCACGAATAAATAACATAAGTTTCTGACTCCTTCCACCATCATTTCTTCT
TCTCCTCGCCTCTGCTGGAGTAGAAGCTGGAGCAGGTACTGGTTGAACAGTTTATCCTCCATTAGCTAGTAACCTAGCACATGCT
GGACCATCTGTTGATTTAGCTATTTTCTCTCTTCACTTAGCCGGTGTGTCATCTATTCTAGCTTCAATTAATTTTATTACAACTATTAT
CAATATAAAACCACCAGCCATTTCCCAATATCAAACACCATTATTTGTTTGATCTATTCTTGTAACCACTATTCTTCTCCTCCTATCAC
TTCCAGTTCTTGCAGCAGGGATTACAATATTACTTACAGATC

No.2025-166-27-27

GCCCTCAGCCTTCTAATTCGAGCCGAGTTAGGCCAGCCCGGATCACTCCTAGGGGATGATCAGGTCTATAATGTTATCGTAACCG
CCCATGCATTTGTAATAATCTTCTTCATGGTTATACCCGTAATAATTGGGGGATTTGGAAACTGATTAGTACCCTTAATAATTGGTG
CACCAGACATGGCCTTCCCGCGAATAAATAACATAAGCTTTTGACTCCTTCCCCCTTCTTTTCTCTTACTCCTAGCTTCAGCTGGG
GTTGAAGCTGGAGCTGGCACTGGTTGAACAGTTTATCCCCCCTTAGCTGGCAACTTAGCACATGCTGGGGCATCTGTTGACTTG
GCCATTTTCTCGCTTCATTTAGCAGGTATCTCATCAATTTTAGCTTCAATTAACTTTATTACAACTATCATTAATATAAAACCACCAG
CCATCTCTCAATATCAAACACCATTATTTGTATGATCAATCCTAGTAACAACCATCCTCCTCCTCTTATCCCTCCCAGTACTCGCAGC
CGGCATCACAA

No.2025-166-27-28

GCCGAGTTAGGCCAGCCCGGATCACTCCTAGGGGATGATCAGGTCTATAATGTTATCGTAACCGCCCATGCATTTGTAATAATCTT
CTTCATGGTTATACCCGTAATAATTGGGGGATTTGGAAACTGATTAGTACCCTTAATAATTGGTGCACCAGACATGGCCTTCCCGC
GAATAAATAACATAAGCTTTTGACTCCTTCCCCCTTCTTTTCTCTTACTCCTAGCTTCAGCTGGGGTTGAAGCTGGAGCTGGCACT
GGTTGAACAGTTTATCCCCCCTTAGCTGGCAACTTAGCACATGCTGGGGCATCTGTTGACTTGGCCATTTTCTCGCTTCATTTAG
CAGGTATCTCATCAATTTTAGCTTCAATTAACTTTATTACAACTATCATTAATATAAAACCACCAGCCATCTCTCAATATCAAACACC
ATTATTTGTATGATCAATCCTAGTAACAACCATCCTCCTCCTCTTATCCCTCCCAGTACTCGCAGCCGGCATCACAATATTATTAACT
GATCGAAACCTAAACACA

No.2025-166-27-29

TGAGCAGGAATAGTGGGGACAGCCCTAAGCCTCTTAATTCGAGCCGAATTAGGACAGCCAGGATCACTTCTAGGAGATGATCA
AATCTATAATGTTATTGTAACCGCCCATGCATTCGTAATAATCTTCTTTATAGTTATACCCGTAATAATTGGCGGATTTGGAAACTGA
CTAGTGCCATTAATAATTGGTGCACCAGACATAGCTTTTCCACGAATAAATAATATAAGCTTTTGACTCCTTCCCCCCTCTTTTCTT
TTACTTCTAGCTTCAGCTGGAGTTGAAGCCGGAGCCGGTACTGGTTGAACAGTTTATCCTCCATTAGCTGGCAATTTAGCACATG
CTGGGGCATCCGTTGACTTAGCTATTTTCTCTCTCCATTTAGCAGGTATTTCATCAATTTTAGCCTCAATCAACTTTATTACAACTAT
TATTAATATAAAACCCCCTGCAATCTCCCAATATCAAACACCACTATTTGTGTGATCAATTCTAGTAACAACTATCCTCCTTCTATTAT
CCCTCCCAGTACTTGCAGCCGGCATTACAATACTACT

No.2025-166-27-30

TGAGCAGGTATAGTTGGAACAGCCCTAAGTCTTCTAATTCGAGCTGAGCTTGGACAACCTGGATCACTTTTAGGGGATGATCAG
ATTTATAATGTAATCGTAACCGCCCACGCTTTTGTAATAATCTTTTTTATGGTTATGCCAATCATAATTGGTGGTTTCGGAAATTGAC
TAGTTCCTTTAATAATTGGTGCACCAGATATAGCCTTCCCACGAATAAATAACATAAGTTTCTGACTTCTTCCACCATCATTTCTTCT
TCTCCTCGCCTCTGCTGGAGTAGAAGCTGGAGCAGGTACTGGTTGAACAGTTTATCCTCCATTAGCTAGTAACCTAGCACATGCT
GGACCATCTGTTGATTTAGCTATTTTCTCTCTTCACTTAGCCGGTGTGTCATCTATTCTAGCTTCAATTAATTTTATTACAACTATTAT
CAATATAAAACCACCAGCCATTTCCCAATATCAAACACCATTATTTGTTTGATCTATTCTTGTAACCACTATTCTTCTCCTCCTATCAC
TTCCAGTTCTTGCAGCAGGGATTACAATAT

No.2025-166-27-31

TGAGCAGGTATAGTTGGAACAGCCCTAAGTCTTCTAATTCGAGCTGAGCTTGGACAACCTGGATCACTTTTAGGGGATGATCAG
ATTTATAATGTAATCGTAACCGCCCACGCTTTTGTAATAATCTTTTTTATGGTTATGCCAATCATAATTGGTGGTTTCGGAAATTGAC
TAGTTCCTTTAATAATTGGTGCACCAGATATAGCCTTCCCACGAATAAATAACATAAGTTTCTGACTTCTTCCACCATCATTTCTTCT
TCTCCTCGCCTCTGCTGGAGTAGAAGCTGGAGCAGGTACTGGTTGAACAGTTTATCCTCCATTAGCTAGTAACCTAGCACATGCT
GGACCATCTGTTGATTTAGCTATTTTCTCTCTTCACTTAGCCGGTGTGTCATCTATTCTAGCTTCAATTAATTTTATTACAACTATTAT
CAATATAAAACCACCAGCCATTTCCCAATATCAAACACCATTATTTGTTTGATCTATTCTTGTAACCACTATTCTTCTCCTCCTATCAC
TTCCAGTTCTTGCAGCAGGGATTACAATATTACTTACAGATCG

No.2025-166-27-32

ATGAGCAGGTATAGTTGGAACAGCCCTAAGTCTTCTAATTCGAGCTGAGCTTGGACAACCTGGATCACTTTTAGGGGATGATCA
GATTTATAATGTAATCGTAACCGCCCACGCTTTTGTAATAATCTTTTTTATGGTTATGCCAATCATAATTGGTGGTTTCGGAAATTG
ACTAGTTCCTTTAATAATTGGTGCACCAGATATAGCCTTCCCACGAATAAATAACATAAGTTTCTGACTTCTTCCACCATCATTTCTT
CTTCTCCTCGCCTCTGCTGGAGTAGAAGCTGGAGCAGGTACTGGTTGAACAGTTTATCCTCCATTAGCTAGTAACCTAGCACATG
CTGGACCATCTGTTGATTTAGCTATTTTCTCTCTTCACTTAGCCGGTGTGTCATCTATTCTAGCTTCAATTAATTTTATTACAACTATT
ATCAATATAAAACCACCAGCCATTTCCCAATATCAAACACCATTATTTGTTTGATCTATTCTTGTAACCACTATTCTTCTCCTCCTATC
ACTTCCAGTTCTTGCAGCAGGGATTACAATATTACTTACAGATCG

No.2025-166-27-33

TGAGCAGGTATAGTTGGAACAGCCCTAAGTCTTCTAATTCGAGCTGAGCTTGGACAACCTGGATCACTTTTAGGGGATGATCAG
ATTTATAATGTAATCGTAACCGCCCACGCTTTTGTAATAATCTTTTTTATGGTTATGCCAATCATAATTGGTGGTTTCGGAAATTGAC
TAGTTCCTTTAATAATTGGTGCACCAGATATAGCCTTCCCACGAATAAATAACATAAGTTTCTGACTTCTTCCACCATCATTTCTTCT
TCTCCTCGCCTCTGCTGGAGTAGAAGCTGGAGCAGGTACTGGTTGAACAGTTTATCCTCCATTAGCTAGTAACCTAGCACATGCT
GGACCATCTGTTGATTTAGCTATTTTCTCTCTTCACTTAGCCGGTGTGTCATCTATTCTAGCTTCAATTAATTTTATTACAACTATTAT
CAATATAAAACCACCAGCCATTTCCCAATATCAAACACCATTATTTGTTTGATCTATTCTTGTAACCACTATTCTTCTCCTCCTATCAC
TTCCAGTTCTTGCAGCAGGGATTACAATATTACTTACAGATCGTA

No.2025-166-27-34

TGAGCAGGAATAGTGGGGACAGCCCTAAGCCTCTTAATTCGAGCCGAATTAGGACAGCCAGGATCACTTCTAGGAGATGATCA
AATCTATAATGTTATTGTAACCGCCCATGCATTCGTAATAATCTTCTTTATAGTTATACCCGTAATAATTGGCGGATTTGGAAACTGA
CTAGTGCCATTAATAATTGGTGCACCAGACATAGCTTTTCCACGAATAAATAATATAAGCTTTTGACTCCTTCCCCCCTCTTTTCTT
TTACTTCTAGCTTCAGCTGGAGTTGAAGCCGGAGCCGGTACTGGTTGAACAGTTTATCCTCCATTAGCTGGCAATTTAGCACATG
CTGGGGCATCCGTTGACTTAGCTATTTTCTCTCTCCATTTAGCAGGTATTTCATCAATTTTAGCCTCAATCAACTTTATTACAACTAT
TATTAATATAAAACCCCCTGCAATCTCCCAATATCAAACACCACTATTTGTGTGATCAATTCTAGTAACAACTATCCTCCTTCTATTAT
CCCTCCCAGTACTTGCAGCCGGCATTACAATACTACT

No.2025-166-27-35

TGCATGAGCAGGTATAGTTGGAACAGCCCTAAGTCTTCTAATTCGAGCTGAGCTTGGACAACCTGGATCACTTTTAGGGGATGA
TCAGATTTATAATGTAATCGTAACCGCCCACGCTTTTGTAATAATCTTTTTTATGGTTATGCCAATCATAATTGGTGGTTTCGGAAAT
TGACTAGTTCCTTTAATAATTGGTGCACCAGATATAGCCTTCCCACGAATAAATAACATAAGTTTCTGACTTCTTCCACCATCATTT
CTTCTTCTCCTCGCCTCTGCTGGAGTAGAAGCTGGAGCAGGTACTGGTTGAACAGTTTATCCTCCATTAGCTAGTAACCTAGCAC
ATGCTGGACCATCTGTTGATTTAGCTATTTTCTCTCTTCACTTAGCCGGTGTGTCATCTATTCTAGCTTCAATTAATTTTATTACAACT
ATTATCAATATAAAACCACCAGCCATTTCCCAATATCAAACACCATTATTTGTTTGATCTATTCTTGTAACCACTATTCTTCTCCTCCT
ATCACTTCCAGTTCTTGCAGCAGGGATTACAATATTACTTACAGATCG

No.2025-166-27-36

TGAGCAGGTATAGTTGGAACAGCCCTAAGTCTTCTAATTCGAGCTGAGCTTGGACAACCTGGATCACTTTTAGGGGATGATCAG
ATTTATAATGTAATCGTAACCGCCCACGCTTTTGTAATAATCTTTTTTATGGTTATGCCAATCATAATTGGTGGTTTCGGAAATTGAC
TAGTTCCTTTAATAATTGGTGCACCAGATATAGCCTTCCCACGAATAAATAACATAAGTTTCTGACTTCTTCCACCATCATTTCTTCT
TCTCCTCGCCTCTGCTGGAGTAGAAGCTGGAGCAGGTACTGGTTGAACAGTTTATCCTCCATTAGCTAGTAACCTAGCACATGCT
GGACCATCTGTTGATTTAGCTATTTTCTCTCTTCACTTAGCCGGTGTGTCATCTATTCTAGCTTCAATTAATTTTATTACAACTATTAT
CAATATAAAACCACCAGCCATTTCCCAATATCAAACACCATTATTTGTTTGATCTATTCTTGTAACCACTATTCTTCTCCTCCTATCAC
TTCCAGTTCTTGCAGCAGGGATTACAATATTACTTACAGATCG

No.2025-166-27-37

GCCTTCTAATTCGAGCCGAGTTAGGCCAGCCCGGATCACTCCTAGGGGATGATCAGGTCTATAATGTTATCGTAACCGCCCATGC
ATTTGTAATAATCTTCTTCATGGTTATACCCGTAATAATTGGGGGATTTGGAAACTGATTAGTACCCTTAATAATTGGTGCACCAGA
CATGGCCTTCCCGCGAATAAATAACATAAGCTTTTGACTCCTTCCCCCTTCTTTTCTCTTACTCCTAGCTTCAGCTGGGGTTGAAG
CTGGAGCTGGCACTGGTTGAACAGTTTATCCCCCCTTAGCTGGCAACTTAGCACATGCTGGGGCATCTGTTGACTTGGCCATTT
TCTCGCTTCATTTAGCAGGTATCTCATCAATTTTAGCTTCAATTAACTTTATTACAACTATCATTAATATAAAACCACCAGCCATCTCT
CAATATCAAACACCATTATTTGTATGATCAATCCTAGTAACAACCATCCTCCTCCTCTTATCCCTCCCAGTACTCGCAGCCGGCATCA
CAATATTATTAACTGATCGAAACCTAAACACAACATTCTTT

No.2025-166-27-38

GCCCTAAGCCTCTTAATTCGAGCCGAATTAGGACAGCCAGGATCACTTCTAGGAGATGATCAAATCTATAATGTTATTGTAACCGC
CCATGCATTCGTAATAATCTTCTTTATAGTTATACCCGTAATAATTGGCGGATTTGGAAACTGACTAGTGCCATTAATAATTGGTGC
ACCAGACATAGCTTTTCCACGAATAAATAATATAAGCTTTTGACTCCTTCCCCCCTCTTTTCTTTTACTTCTAGCTTCAGCTGGAGT
TGAAGCCGGAGCCGGTACTGGTTGAACAGTTTATCCTCCATTAGCTGGCAATTTAGCACATGCTGGGGCATCCGTTGACTTAGC
TATTTTCTCTCTCCATTTAGCAGGTATTTCATCAATTTTAGCCTCAATCAACTTTATTACAACTATTATTAATATAAAACCCCCTGCAA
TCTCCCAATATCAAACACCACTATTTGTGTGATCAATTCTAGTAACAACTATCCTCCTTCTATTATCCCTCCCAGTACTTGCAGCCGG
CATTACAATACTACTTACTGATCGAAACCTAAACACAACATTCT

No.2025-166-27-39

TGAGCAGGAATAGTGGGACCAGCCCTCAGCCTTCTAATTCGAGCCGAGTTAGGCCAGCCCGGATCACTCCTAGGGGATGATCA
GGTCTATAATGTTATCGTAACCGCCCATGCATTTGTAATAATCTTCTTCATGGTTATACCCGTAATAATTGGGGGATTTGGAAACTG
ATTAGTACCCTTAATAATTGGTGCACCAGACATGGCCTTCCCGCGAATAAATAACATAAGCTTTTGACTCCTTCCCCCTTCTTTTCT
CTTACTCCTAGCTTCAGCTGGGGTTGAAGCTGGAGCTGGCACTGGTTGAACAGTTTATCCCCCCTTAGCTGGCAACTTAGCACA
TGCTGGGGCATCTGTTGACTTGGCCATTTTCTCGCTTCATTTAGCAGGTATCTCATCAATTTTAGCTTCAATTAACTTTATTACAAC
TATCATTAATATAAAACCACCAGCCATCTCTCAATATCAAACACCATTATTTGTATGATCAATCCTAGTAACAACCATCCTCCTCCTCT
TATCCCTCCCAGTACTCGCAGCCGGCATCACAATATTAT

No.2025-166-27-40

GCCCTAAGCCTTTTAATTCGTGCCGAACTGGGTCAGCCTGGTTCCCTCCTAGGGGATGATCAGATTTATAATGTTATTGTAACCGC
CCATGCATTTGTAATAATTTTCTTTATGGTTATGCCCGTAATAATTGGAGGCTTTGGAAATTGACTAGTGCCTTTAATGATCGGAGC
ACCAGACATAGCCTTCCCCCGAATAAATAACATAAGTTTCTGGCTCCTACCCCCTTCTTTCCTTTTACTCTTAGCCTCAGCCGGAG
TTGAGTCAGGAGCCGGCACTGGTTGAACAGTCTACCCTCCCCTAGCTGGCAACTTAGCACACGCCGGAGCATCTGTTGATCTAG
CCATTTTTTCCCTCCACCTGGCTGGTATCTCGTCCATCCTAGCTTCCATTAACTTCATTACAACCATCATCAACATAAAACCCCCAG
CAATCTCCCAATACCAAACACCCCTGTTTGTCTGGTCCATTCTAGTGACAACCATCCTCCTTCTTTTAGCACTCCCAGTGCTCGCC
GCTGGCATTACAATACTACTTACGGACCGAAACCTAAACACAACATTCTTT

No.2025-166-27-41

TGAGCAGGAATAGTGGGGACGGCCCTAAGCCTCTTAATTCGAGCCGAATTAGGACAGCCAGGATCACTTCTAGGAGATGATCA
AATCTATAATGTTATTGTAACCGCCCATGCATTCGTAATAATCTTCTTTATAGTTATACCCGTAATAATTGGCGGATTTGGAAACTGA
CTAGTGCCATTAATAATTGGTGCACCAGACATAGCTTTTCCACGAATAAATAATATAAGCTTTTGACTCCTTCCCCCCTCTTTTCTT
TTACTTCTAGCTTCAGCTGGAGTTGAAGCCGGAGCCGGTACTGGTTGAACAGTTTATCCTCCATTAGCTGGCAATTTAGCACATG
CTGGGGCATCCGTTGACTTAGCTATTTTCTCTCTCCATTTAGCAGGTATTTCATCAATTTTAGCCTCAATCAACTTTATTACAACTAT
TATTAATATAAAACCCCCTGCAATCTCCCAATATCAAACACCACTATTTGTGTGATCAATTCTAGTAACAACTATCCTCCTTCTATTAT
CCCTCCCAGTACTTGCAGCCGGCATTACAATACTACT

No.2025-166-27-42

GCCCTAAGCCTTTTAATTCGTGCCGAACTGGGTCAGCCTGGTTCCCTCCTAGGGGATGATCAGATTTATAATGTTATTGTAACCGC
CCATGCATTTGTAATAATTTTCTTTATGGTTATGCCCGTAATAATTGGAGGCTTTGGAAATTGACTAGTGCCTTTAATGATCGGAGC
ACCAGACATAGCCTTCCCCCGAATAAATAACATAAGTTTCTGGCTCCTACCCCCTTCTTTCCTTTTACTCTTAGCCTCAGCCGGAG
TTGAGTCAGGAGCCGGCACTGGTTGAACAGTCTACCCTCCCCTAGCTGGCAACTTAGCACACGCCGGAGCATCTGTTGATCTAG
CCATTTTCTCCCTCCACCTGGCTGGTATCTCGTCCATCCTAGCTTCCATTAACTTCATTACAACCATCATCAACATAAAACCCCCAG
CAATCTCCCAATACCAAACACCCCTGTTTGTCTGGTCCATTCTAGTGACAACCATCCTCCTTCTTTTAGCACTCCCAGTGCTCGCC
GCTGGCATTACAATACTACTTACGGACCGAAACCTAAACACAACATTCTTT

No.2025-166-27-43

CAGCCCTAAGTCTTCTAATTCGAGCTGAGCTTGGACAACCTGGATCACTTTTAGGGGATGATCAGATTTATAATGTAATCGTAACC
GCCCACGCTTTTGTAATAATCTTTTTTATGGTTATGCCAATCATAATTGGTGGTTTCGGAAATTGACTAGTTCCTTTAATAATTGGT
GCACCAGATATAGCCTTCCCACGAATAAATAACATAAGTTTCTGACTTCTTCCACCATCATTTCTTCTTCTCCTCGCCTCTGCTGGA
GTAGAAGCTGGAGCAGGTACTGGTTGAACAGTTTATCCTCCATTAGCTAGTAACCTAGCACATGCTGGACCATCTGTTGATTTAG
CTATTTTCTCTCTTCACTTAGCCGGTGTGTCATCTATTCTAGCTTCAATTAATTTTATTACAACTATTATCAATATAAAACCACCAGCC
ATTTCCCAATATCAAACACCATTATTTGTTTGATCTATTCTTGTAACCACTATTCTTCTCCTCCTATCACTTCCAGTTCTTGCAGCAG
GGATTACAATATTACTTACAGATCG

No.2025-166-27-44

TGAGCAGGTATAGTTGGAACAGCCCTAAGTCTTCTAATTCGAGCTGAGCTTGGACAACCTGGATCACTTTTAGGGGATGATCAG
ATTTATAATGTAATCGTAACCGCCCACGCTTTTGTAATAATCTTTTTTATGGTTATGCCAATCATAATTGGTGGTTTCGGAAATTGAC
TAGTTCCTTTAATAATTGGTGCACCAGATATAGCCTTCCCACGAATAAATAACATAAGTTTCTGACTTCTTCCACCATCATTTCTTCT
TCTCCTCGCCTCTGCTGGAGTAGAAGCTGGAGCAGGTACTGGTTGAACAGTTTATCCTCCATTAGCTAGTAACCTAGCACATGCT
GGACCATCTGTTGATTTAGCTATTTTCTCTCTTCACTTAGCCGGTGTGTCATCTATTCTAGCTTCAATTAATTTTATTACAACTATTAT
CAATATAAAACCACCAGCCATTTCCCAATATCAAACACCATTATTTGTTTGATCTATTCTTGTAACCACTATTCTTCTCCTCCTATCAC
TTCCAGTTCTTGCAGCAGGGATTACAATATTACTTACAGATCG

No.2025-166-27-45

TGAGCAGGTATAGTTGGAACAGCCCTAAGTCTTCTAATTCGAGCTGAGCTTGGACAACCTGGATCACTTTTAGGGGATGATCAG
ATTTATAATGTAATCGTAACCGCCCACGCTTTTGTAATAATCTTTTTTATGGTTATGCCAATCATAATTGGTGGTTTCGGAAATTGAC
TAGTTCCTTTAATAATTGGTGCACCAGATATAGCCTTCCCACGAATAAATAACATAAGTTTCTGACTTCTTCCACCATCATTTCTTCT
TCTCCTCGCCTCTGCTGGAGTAGAAGCTGGAGCAGGTACTGGTTGAACAGTTTATCCTCCATTAGCTAGTAACCTAGCACATGCT
GGACCATCTGTTGATTTAGCTATTTTCTCTCTTCACTTAGCCGGTGTGTCATCTATTCTAGCTTCAATTAATTTTATTACAACTATTAT
CAATATAAAACCACCAGCCATTTCCCAATATCAAACACCATTATTTGTTTGATCTATTCTTGTAACCACTATTCTTCTCCTCCTATCAC
TTCCAGTTCTTGCAGCAGGGATTACAATATTACTTACAGATCG

No.2025-166-27-46

CAGCCCTAAGTCTTCTAATTCGAGCTGAGCTTGGACAACCTGGATCACTTTTAGGGGATGATCAGATTTATAATGTAATCGTAACC
GCCCACGCTTTTGTAATAATCTTTTTTATGGTTATGCCAATCATAATTGGTGGTTTCGGAAATTGACTAGTTCCTTTAATAATTGGT
GCACCAGATATAGCCTTCCCACGAATAAATAACATAAGTTTCTGACTTCTTCCACCATCATTTCTTCTTCTCCTCGCCTCTGCTGGA
GTAGAAGCTGGAGCAGGTACTGGTTGAACAGTTTATCCTCCATTAGCTAGTAACCTAGCACATGCTGGACCATCTGTTGATTTAG
CTATTTTCTCTCTTCACTTAGCCGGTGTGTCATCTATTCTAGCTTCAATTAATTTTATTACAACTATTATCAATATAAAACCACCAGCC
ATTTCCCAATATCAAACACCATTATTTGTTTGATCTATTCTTGTAACCACTATTCTTCTCCTCCTATCACTTCCAGTTCTTGCAGCAG
GGATTACAATATTACTTACAGATCG

No.2025-166-27-47

CAGCCCTAAGTCTTCTAATTCGAGCTGAGCTTGGACAACCTGGATCACTTTTAGGGGATGATCAGATTTATAATGTAATCGTAACC
GCCCACGCTTTTGTAATAATCTTTTTTATGGTTATGCCAATCATAATTGGTGGTTTCGGAAATTGACTAGTTCCTTTAATAATTGGT
GCACCAGATATAGCCTTCCCACGAATAAATAACATAAGTTTCTGACTTCTTCCACCATCATTTCTTCTTCTCCTCGCCTCTGCTGGA
GTAGAAGCTGGAGCAGGTACTGGTTGAACAGTTTATCCTCCATTAGCTAGTAACCTAGCACATGCTGGACCATCTGTTGATTTAG
CTATTTTCTCTCTTCACTTAGCCGGTGTGTCATCTATTCTAGCTTCAATTAATTTTATTACAACTATTATCAATATAAAACCACCAGCC
ATTTCCCAATATCAAACACCATTATTTGTTTGATCTATTCTTGTAACCACTATTCTTCTCCTCCTATCACTTCCAGTTCTTGCAGCAG
GGATTACAATATTACTTACAGATCG

No.2025-166-27-48

TGAGCAGGTATAGTTGGAACAGCCCTAAGTCTTCTAATTCGAGCTGAGCTTGGACAACCTGGATCACTTTTAGGGGATGATCAG
ATTTATAATGTAATCGTAACCGCCCACGCTTTTGTAATAATCTTTTTTATGGTTATGCCAATCATAATTGGTGGTTTCGGAAATTGAC
TAGTTCCTTTAATAATTGGTGCACCAGATATAGCCTTCCCACGAATAAATAACATAAGTTTCTGACTTCTTCCACCATCATTTCTTCT
TCTCCTCGCCTCTGCTGGAGTAGAAGCTGGAGCAGGTACTGGTTGAACAGTTTATCCTCCATTAGCTAGTAACCTAGCACATGCT
GGACCATCTGTTGATTTAGCTATTTTCTCTCTTCACTTAGCCGGTGTGTCATCTATTCTAGCTTCAATTAATTTTATTACAACTATTAT
CAATATAAAACCACCAGCCATTTCCCAATATCAAACACCATTATTTGTTTGATCTATTCTTGTAACCACTATTCTTCTCCTCCTATCAC
TTCCAGTTCTTGCAGCAGGGATTACAATATTACTTACAGATCG

No.2025-166-27-49

TGAGCAGGTATAGTTGGAACAGCCCTAAGTCTTCTAATTCGAGCTGAGCTTGGACAACCTGGATCACTTTTAGGGGATGATCAG
ATTTATAATGTAATCGTAACCGCCCACGCTTTTGTAATAATCTTTTTTATGGTTATGCCAATCATAATTGGTGGTTTCGGAAATTGAC
TAGTTCCTTTAATAATTGGTGCACCAGATATAGCCTTCCCACGAATAAATAACATAAGTTTCTGACTTCTTCCACCATCATTTCTTCT
TCTCCTCGCCTCTGCTGGAGTAGAAGCTGGAGCAGGTACTGGTTGAACAGTTTATCCTCCATTAGCTAGTAACCTAGCACATGCT
GGACCATCTGTTGATTTAGCTATTTTCTCTCTTCACTTAGCCGGTGTGTCATCTATTCTAGCTTCAATTAATTTTATTACAACTATTAT
CAATATAAAACCACCAGCCATTTCCCAATATCAAACACCATTATTTGTTTGATCTATTCTTGTAACCACTATTCTTCTCCTCCTATCAC
TTCCAGTTCTTGCAGCAGGGATTACAATAT

No.2025-166-27-50

GTATAGTTGGAACAGCCCTAAGTCTTCTAATTCGAGCTGAGCTTGGACAACCTGGATCACTTTTAGGGAATGATCAGATTTATAA
TGTAATCGTAACCGCCCACGCTTTTGTAATAATCTTTTTTATGGTTATGCCAATCATAATTGGTGGTTTCGGAAATTGACTAGTTCC
TTTAATAATTGGTGCACCAGATATAGCCTTCCCACGAATAAATAACATAAGTTTCTGACTTCTTCCACCATCATTTCTTCTTCTCCTC
GCCTCTGCTGGAGTAGAAGCTGGAGCAGGTACTGGTTGAACAGTTTATCCTCCATTAGCTAGTAACCTAGCACATGCTGGACCA
TCTGTTGATTTAGCTATTTTCTCTCTTCACTTAGCCGGTGTGTCATCTATTCTAGCTTCAATTAATTTTATTACAACTATTATCAATATA
AAACCACCAGCCATTTCCCAATATCAAACACCATTATTTGTTTGATCTATTCTTGTAACCACTATTCTTCTCCTCCTATCACTTCCAG
TTCTTGCAGCAGGGATTACAATATTACTTACAGATC

No.2025-166-27-51

TGAGCAGGTATAGTTGGAACAGCCCTAAGTCTTCTAATTCGAGCTGAGCTTGGACAACCTGGATCACTTTTAGGGGATGATCAG
ATTTATAATGTAATCGTAACCGCCCACGCTTTTGTAATAATCTTTTTTATGGTTATGCCAATCATAATTGGTGGTTTCGGAAATTGAC
TAGTTCCTTTAATAATTGGTGCACCAGATATAGCCTTCCCACGAATAAATAACATAAGTTTCTGACTTCTTCCACCATCATTTCTTCT
TCTCCTCGCCTCTGCTGGAGTAGAAGCTGGAGCAGGTACTGGTTGAACAGTTTATCCTCCATTAGCTAGTAACCTAGCACATGCT
GGACCATCTGTTGATTTAGCTATTTTCTCTCTTCACTTAGCCGGTGTGTCATCTATTCTAGCTTCAATTAATTTTATTACAACTATTAT
CAATATAAAACCACCAGCCATTTCCCAATATCAAACACCATTATTTGTTTGATCTATTCTTGTAACCACTATTCTTCTCCTCCTATCAC
TTCCAGTTCTTGCAGCAGGGATTACAATAT

No.2025-166-27-52

GCCCTAAGCCTTTTAATTCGTGCCGAACTGGGTCAGCCTGGTTCCCTCCTAGGGGATGATCAAATTTATAATGTTATTGTAACCGC
CCATGCATTTGTAATAATTTTCTTTATGGTTATGCCCGTAATAATTGGAGGCTTTGGAAATTGACTAGTGCCTTTAATGATCGGAGC
ACCAGACATAGCCTTCCCCCGAATAAATAACATAAGTTTCTGGCTCCTACCCCCTTCTTTCCTTTTACTCTTAGCCTCAGCCGGAG
TTGAGTCAGGAGCCGGCACTGGTTGAACAGTCTACCCTCCCCTAGCTGGCAACTTAGCACACGCCGGAGCATCTGTTGATCTAG
CCATTTTCTCCCTCCACCTGGCTGGTATCTCGTCCATCCTAGCTTCCATTAACTTCATTACAACCATCATCAACATAAAACCCCCAG
CAATCTCCCAATACCAAACACCCCTGTTTGTCTGGTCCATTCTAGTGACAACCATCCTCCTTCTTTTAGCACTCCCAGTGCTCGCC
GCTGGCATTACAATACTACTTACGGACCGAAACCTAAACACAACATTCT

No.2025-166-27-53

GCCCTAAGCCTTTTAATTCGTGCCGAACTGGGTCAGCCTGGTTCCCTCCTAGGGGATGATCAGATTTATAATGTTATTGTAACCGC
CCATGCATTTGTAATAATTTTCTTTATGGTCATGCCCGTAATAATTGGAGGCTTTGGAAATTGACTAGTACCTTTAATGATCGGAGC
ACCAGACATAGCCTTCCCCCGAATAAATAACATAAGTTTCTGGCTCCTACCCCCTTCTTTCCTTCTACTCTTGGCCTCAGCCGGAG
TTGAGTCAGGAGCCGGCACTGGCTGAACAGTCTACCCTCCCCTAGCTGGCAACTTAGCACACGCCGGAGCATCTGTTGATCTAG
CCATTTTCTCCCTCCACCTGGCTGGTATCTCGTCCATCCTAGCTTCCATTAACTTCATTACAACCATCATCAACATAAAACCCCCAG
CAATCTCCCAATACCAGACACCCCTGTTTGTCTGGTCCATTCTAGTGACAACCATCCTCCTTCTTTTAGCACTCCCAGTGCTCGCC
GCTGGCATTACAATACTACTTACGGACCGAAACCTAAACACAACATTCT

No.2025-166-27-54

GCCCTAAGCCTTTTAATTCGTGCCGAACTGGGTCAGCCTGGTTCCCTCCTAGGGGATGATCAGATTTATAATGTTATTGTAACCGC
CCATGCATTTGTAATAATTTTCTTTATGGTCATGCCCGTAATAATTGGAGGCTTTGGAAATTGACTAGTACCTTTAATGATCGGAGC
ACCAGACATAGCCTTCCCCCGAATAAATAACATAAGTTTCTGGCTCCTACCCCCTTCTTTCCTTCTACTCTTGGCCTCAGCCGGAG
TTGAGTCAGGAGCCGGCACTGGCTGAACAGTCTACCCTCCCCTAGCTGGCAACTTAGCACACGCCGGAGCATCTGTTGATCTAG
CCATTTTCTCCCTCCACCTGGCTGGTATCTCGTCCATCCTAGCTTCCATTAACTTCATTACAACCATCATCAACATAAAACCCCCAG
CAATCTCCCAATACCAAACACCCCTGTTTGTCTGGTCCATTCTAGTGACAACCATCCTCCTTCTTTTAGCACTCCCAGTGCTCGCC
GCTGGCATTACAATACTACTTACGGACCGAAACCTAAACACAACATTCT

No.2025-166-27-55

GCCCTAAGCCTTTTAATTCGTGCCGAACTGGGTCAGCCTGGTTCCCTCCTAGGAGATGATCAGATTTATAATGTTATTGTAACCGC
CCATGCATTTGTAATAATTTTCTTTATGGTTATGCCCGTAATAATTGGAGGCTTTGGAAATTGACTAGTGCCTTTAATGATCGGAGC
ACCAGACATAGCCTTCCCCCGAATAAATAACATAAGTTTCTGGCTCCTACCCCCTTCTTTCCTTTTACTCTTAGCCTCAGCCGGAG
TTGAATCAGGAGCCGGCACTGGTTGAACAGTCTACCCTCCCCTAGCTGGCAACTTAGCACACGCCGGAGCATCTGTTGATCTAG
CCATTTTCTCCCTCCACCTGGCTGGTATCTCGTCCATCCTAGCTTCCATTAACTTCATTACAACCATCATCAACATAAAACCCCCAG
CAATCTCCCAATACCAAACACCCCTGTTTGTCTGGTCCATTCTAGTGACAACCATCCTCCTTCTTTTAGCACTCCCAGTGCTCGCC
GCTGGCATTACAATACTACTTACGGACCGAAACCTAAA

No.2025-166-27-56

GCCCTAAGCCTTTTAATTCGTGCCGAACTGGGTCAGCCTGGTTCCCTCCTAGGGGATGATCAGATTTATAATGTTATTGTAACCGC
CCATGCATTTGTAATAATTTTCTTTATGGTTATGCCCGTAATAATTGGAGGCTTTGGAAATTGACTAGTGCCTTTAATGATCGGAGC
ACCAGACATAGCCTTCCCCCGAATAAATAACATAAGTTTCTGGCTCCTACCCCCTTCTTTCCTTTTACTCTTAGCCTCAGCCGGAG
TTGAGTCAGGAGCCGGCACTGGTTGAACAGTCTACCCTCCCCTAGCTGGCAACTTAGCACACGCCGGAGCATCTGTTGATCTAG
CCATTTTCTCCCTCCACCTGGCTGGTATCTCGTCCATCCTAGCTTCCATTAACTTCATTACAACCATCATCAACATAAAACCCCCAG
CAATCTCCCAATACCAAACACCCCTGTTTGTCTGGTCCATTCTAGTGACAACCATCCTCCTTCTTTTAGCACTCCCAGTGCTCGCC
GCTGGCATTACAATACTACTTACGGACCGAAACCTAAACACAACATTCT

No.2025-166-27-57

TGAGCAGGTATAGTTGGAACAGCCCTAAGTCTTCTAATTCGAGCTGAGCTTGGACAACCTGGATCACTTTTAGGGGATGATCAG
ATTTATAATGTAATCGTAACCGCCCACGCTTTTGTAATAATCTTTTTTATGGTTATGCCAATCATAATTGGTGGTTTCGGAAATTGAC
TAGTTCCTTTAATAATTGGTGCACCAGATATAGCCTTCCCACGAATAAATAACATAAGTTTCTGACTTCTTCCACCATCATTTCTTCT
TCTCCTCGCCTCTGCTGGAGTAGAAGCTGGAGCAGGTACTGGTTGAACAGTTTATCCTCCATTAGCTAGTAACCTAGCACATGCT
GGACCATCTGTTGATTTAGCTATTTTCTCTCTTCACTTAGCCGGTGTGTCATCTATTCTAGCTTCAATTAATTTTATTACAACTATTAT
CAATATAAAACCACCAGCCATTTCCCAATATCAAACACCATTATTTGTTTGATCTATTCTTGTAACCACTATTCTTCTCCTCCTATCAC
TTCCAGTTCTTGCAGCAGGGATTACAATATTACTTACAGATCG

No.2025-166-27-58

TGAGCAGGTATAGTTGGAACAGCCCTAAGTCTTCTAATTCGAGCTGAGCTTGGACAACCTGGATCACTTTTAGGGGATGATCAG
ATTTATAATGTAATCGTAACCGCCCACGCTTTTGTAATAATCTTTTTTATGGTTATGCCAATCATAATTGGTGGTTTCGGAAATTGAC
TAGTTCCTTTAATAATTGGTGCACCAGATATAGCCTTCCCACGAATAAATAACATAAGTTTCTGACTTCTTCCACCATCATTTCTTCT
TCTCCTCGCCTCTGCTGGAGTAGAAGCTGGAGCAGGTACTGGTTGAACAGTTTATCCTCCATTAGCTAGTAACCTAGCACATGCT
GGACCATCTGTTGATTTAGCTATTTTCTCTCTTCACTTAGCCGGTGTGTCATCTATTCTAGCTTCAATTAATTTTATTACAACTATTAT
CAATATAAAACCACCAGCCATTTCCCAATATCAAACACCATTATTTGTTTGATCTATTCTTGTAACCACTATTCTTCTCCTCCTATCAC
TTCCAGTTCTTGCAGCAGGGATTACAATAT

No.2025-166-27-59

TGAGCAGGTATAGTTGGAACAGCCCTAAGTCTTCTAATTCGAGCTGAGCTTGGACAACCTGGATCACTTTTAGGGGATGATCAG
ATTTATAATGTAATCGTAACCGCCCACGCTTTTGTAATAATCTTTTTTATGGTTATGCCAATCATAATTGGTGGTTTCGGAAATTGAC
TAGTTCCTTTAATAATTGGTGCACCAGATATAGCCTTCCCACGAATAAATAACATAAGTTTCTGACTTCTTCCACCATCATTTCTTCT
TCTCCTCGCCTCTGCTGGAGTAGAAGCTGGAGCAGGTACTGGTTGAACAGTTTATCCTCCATTAGCTAGTAACCTAGCACATGCT
GGACCATCTGTTGATTTAGCTATTTTCTCTCTTCACTTAGCCGGTGTGTCATCTATTCTAGCTTCAATTAATTTTATTACAACTATTAT
CAATATAAAACCACCAGCCATTTCCCAATATCAAACACCATTATTTGTTTGATCTATTCTTGTAACCACTATTCTTCTCCTCCTATCAC
TTCCAGTTCTTGCAGCAGGGATTACAATAT

No.2025-166-27-60

TGAGCAGGTATAGTTGGAACAGCCCTAAGTCTTCTAATTCGAGCTGAGCTTGGACAACCTGGATCACTTTTAGGGGATGATCAG
ATTTATAATGTAATCGTAACCGCCCACGCTTTTGTAATAATCTTTTTTATGGTTATGCCAATCATAATTGGTGGTTTCGGAAATTGAC
TAGTTCCTTTAATAATTGGTGCACCAGATATAGCCTTCCCACGAATAAATAACATAAGTTTCTGACTTCTTCCACCATCATTTCTTCT
TCTCCTCGCCTCTGCTGGAGTAGAAGCTGGAGCAGGTACTGGTTGAACAGTTTATCCTCCATTAGCTAGTAACCTAGCACATGCT
GGACCATCTGTTGATTTAGCTATTTTCTCTCTTCACTTAGCCGGTGTGTCATCTATTCTAGCTTCAATTAATTTTATTACAACTATTAT
CAATATAAAACCACCAGCCATTTCCCAATATCAAACACCATTATTTGTTTGATCTATTCTTGTAACCACTATTCTTCTCCTCCTATCAC
TTCCAGTTCTTGCAGCAGGGATTACAATATTACTTACAGATCG

No.2025-166-27-61

TGAGCAGGTATAGTTGGAACAGCCCTAAGTCTTCTAATTCGAGCTGAGCTTGGACAACCTGGATCACTTTTAGGGGATGATCAG
ATTTATAATGTAATCGTAACCGCCCACGCTTTTGTAATAATCTTTTTTATGGTTATGCCAATCATAATTGGTGGTTTCGGAAATTGAC
TAGTTCCTTTAATAATTGGTGCACCAGATATAGCCTTCCCACGAATAAATAACATAAGTTTCTGACTTCTTCCACCATCATTTCTTCT
TCTCCTCGCCTCTGCTGGAGTAGAAGCTGGAGCAGGTACTGGTTGAACAGTTTATCCTCCATTAGCTAGTAACCTAGCACATGCT
GGACCATCTGTTGATTTAGCTATTTTCTCTCTTCACTTAGCCGGTGTGTCATCTATTCTAGCTTCAATTAATTTTATTACAACTATTAT
CAATATAAAACCACCAGCCATTTCCCAATATCAAACACCATTATTTGTTTGATCTATTCTTGTAACCACTATTCTTCTCCTCCTATCAC
TTCCAGTTCTTGCAGCAGGGATTACAATAT

No.2025-166-27-62

TGAGCAGGAATAGTGGGGACAGCCCTAAGCCTCCTTAATTCGAGCCGAATTAGGACAGCCAGGATCACTTCTAGGAGATGATC
AAATCTATAATGTTATTGTAACCGCCCATGCATTCGTAATAATCTTCTTTATAGTTATACCCGTAATAATTGGCGGATTTGGAAACTG
ACTAGTGCCATTAATAATTGGTGCACCAGACATAGCTTTTCCACGAATAAATAATATAAGCTTTTGACTCCTTCCCCCCTCTTTTCT
TTTACTTCTAGCTTCAGCTGGAGTTGAAGCCGGAGCCGGTACTGGTTGAACAGTTTATCCTCCATTAGCTGGCAATTTAGCACAT
GCTGGGGCATCCGTTGACTTAGCTATTTTCTCTCTCCATTTAGCAGGTATTTCATCAATTTTAGCCTCAATCAACTTTATTACAACTA
TTATTAATATAAAACCCCCTGCAATCTCCCAATATCAAACACCACTATTTGTGTGATCAATTCTAGTAACAACTATCCTCCTTCTATTA
TCCCTCCCAGTACTTGCAGCCGGCATTACAATACTACT

No.2025-166-27-63

TGAGCAGGAATAGTGGGGACGGCCCTAAGCCTCTTAATTCGAGCCGAATTAGGACAGCCAGGATCACTTCTAGGAGATGATCA
AATCTATAATGTTATTGTAACCGCCCATGCATTCGTAATAATCTTCTTTATAGTTATACCCGTAATAATTGGCGGATTTGGAAACTGA
CTAGTGCCATTAATAATTGGTGCACCAGACATAGCTTTTCCACGAATAAATAATATAAGCTTTTGACTCCTTCCCCCCTCTTTTCTC
TTACTTCTAGCTTCAGCTGGAGTTGAAGCCGGAGCCGGTACTGGTTGAACAGTTTATCCTCCATTAGCTGGCAATTTAGCACATG
CTGGGGCATCCGTTGACTTAGCTATTTTCTCTCTCCATTTAGCAGGTATTTCATCAATTTTAGCCTCAATCAACTTTATTACAACTAT
TATTAATATAAAACCCCCTGCAATCTCCCAATATCAAACACCACTATTTGTGTGATCAATTCTAGTAACAACTATCCTCCTTCTATTAT
CCCTCCCAGTACTTGCAGCCGGCATTACAATACTACT

No.2025-166-27-64

TGAGCAGGAATAGTGGGAACAGCCCTCAGCCTTCTAATTCGAGCCGAGTTAGGCCAGCCCGGATCACTCCTAGGGGATGATCA
GGTCTATAATGTTATCGTAACCGCCCATGCATTTGTAATAATCTTCTTCATGGTTATACCCGTAATAATTGGGGGATTTGGAAACTG
ATTAGTACCCTTAATAATTGGTGCACCAGACATGGCCTTCCCGCGAATAAATAACATAAGCTTTTGACTCCTTCCCCCTTCTTTTCT
CTTACTCCTAGCTTCAGCTGGGGTTGAAGCTGGAGCTGGCACTGGTTGAACAGTTTATCCCCCCTTAGCTGGCAACTTAGCACA
TGCTGGGGCATCTGTTGACTTGGCCATTTTCTCGCTTCATTTAGCAGGTATCTCATCAATTTTAGCTTCAATTAACTTTATTACAAC
TATCATTAATATAAAACCACCAGCCATCTCTCAATATCAAACACCATTATTTGTATGATCAATCCTAGTAACAACCATCCTCCTCCTCT
TATCCCTCCCAGTACTCGCAGCCGGCATCACAATAT

Shark fin: 16S rRNA gene - Large-sized samples (n=64)

No.2025-166-27-1

CCGCCTGCCCTGTGACAATGTTTTAACGGCCGCGGTATTTTGACCGTGCAAAGGTAGCGTAATCACTTGTCTTTTAAATGAAGAC
CCGTATGAAAGGCATCACGAGAGTTCAACTGTCTCTATTTTCTAATCAATGAAATTGATCTACTCGTGCAGAAGCGAGTATAACTA
CATTAGACGAGAAGACCCTATGGAGCTTCAAACACATGAATTAAATATGTAAACTAACTACTCCCCGGACATAAATAAAATAATAT
TTTTAATTTAACTGTTTTTGGTTGGGGTGACCAAGGGGAAAAATAAATCCCCCTTATCGATTGAGTACTCAAGTACTTAAAAATC
AGAATTACAATTCTGATTAATAAAATATTTATCGAAAAATGACCCAGGATTTCCTGATCAATGAACCAAGTTACCCTAGGGATAAC
AGCGCAATCCTTTCTTAGAGTCCCTATCGCCGAAAGGGTTTACGACCTCGATGTTGGATCAGGACATCCTAATGATGCAACCGTT
ATTAAGGGTTCGTT

No.2025-166-27-2

TGTTTTAACGGCCGCGGTATTTTGACCGTGCAAAGGTAGCGTAATCACTTGTCTTTTAAATGAAGACCCGTATGAAAGGCATCAC
GAGAGTTCAACTGTCTCTATTTTCTAATCAATGAAATTGATCTACTCGTGCAGAAGCGAGTATAACTACATTAGACGAGAAGACC
CTATGGAGCTTCAAACACATGAATTAAATATGTAAACTAACTACTCCCCGGACATAAATAAAATAATATTTTTAATTTAACTGTTTTT
GGTTGGGGTGACCAAGGGGAAAAATAAATCCCCCTTATCGATTGAGTACTCAAGTACTTAAAAATCAGAATTACAATTCTGATTA
ATAAAATATTTATCGAAAAATGACCCAGGATTTCCTGATCAATGAACCAAGTTACCCTAGGGATAACAGCGCAATCCTTTCTTAGA
GTCCCTATCGCCGAAAGGGTTTACGACCTCGATGTTGGATCAGGACATCCTAATGATGCAACCGTTATTAAGGGTTCGTTTGTTC
AACGATTAATAGTCCTACGTGATCTG

No.2025-166-27-3

CCGCCTGCCCTGTGACAATGTTTAACGGCCGCGGTATTCTGACCGTGCAAAGGTAGCGTAATCACTTGTCTTTTAAATGAAGACC
CGTATGAAAGGCATCACGAGAGTTCAACTGTCTCTACTTTCCAATCAATGAAATTGATCCACCCGTGCAGAAGCGGGTATAAACA
CATCAGACGAGAAGACCCTATGGAGCTTCAAACACATGAATTAATTATGTAGACTAACTGCTCCACGGACATAAATAAGAATACA
ACACTTTTAATTTAACTGTTTTGGTTGGGGTGACCGAGGGGAAAAATCAATCCCCCTTATCGACCGAGTGTTCTCAAGCACTTAA
AAATTAGAATTACAATTCTAATTAGTAAAATATTTACCGAAAAATGACCCAGAATTTTCTGATCAATGAACCAAGTTACCCTAGGG
ATAACAGCGCAATCCTTTCCTAGAGTCCCTATCGACGAAAGGGTTTACGACCTCGATGTTGGATCAGGACATCCTAATGATGTAG
CCGTTATTAAGGGTTCGTT

No.2025-166-27-4

AGAGGTCCCGCCTGCCCTGTGACAATGTTTTAACGGCCGCGGTATTTTGACCGTGCAAAGGTAGCGTAATCACTTGTCTTTTAAA
TGAAGACCCGTATGAAAGGCATCACGAGAGTTCAACTGTCTCTATTTTCTAATCAATGAAATTGATCTACTCGTGCAGAAGCGAG
TATAACTACATTAGACGAGAAGACCCTATGGAGCTTCAAACACATGAATTAAATATGTAAACTAACTACTCCCCGGACATAAATAA
AATAATATTTTTAATTTAACTGTTTTTGGTTGGGGTGACCAAGGGGAAAAATAAATCCCCCTTATCGATTGAGTACTCAAGTACTT
AAAAATCAGAATTACAATTCTGATTAATAAAATATTTATCGAAAAATGACCCAGGATTTCCTGATCAATGAACCAAGTTACCCTAG
GGATAACAGCGCAATCCTTTCTTAGAGTCCCTATCGCCGAAAGGGTTTACGACCTCGATGTTGGATCAGGACATCCTAATGATGC
AACCGTTATTAAGGGT

No.2025-166-27-5

TGTGACAACGTTCAACGGCCGCGGTATTTTGACCGTGCAAAGGTAGCGTAATCACTTGTCTTTTAAATGAAGACCTGTATGAAA
GGCATCACGAGAGTTTAACTGTCTCTATTTTCTAATCAATGAAATTGATCTATTCGTGCAGAAGCGAATATAATAACATTAGACGA
GAAGACCCTATGGAGCTTCAAACACTTAAATTAATTATGTAATCCTCCACCTCCCAGGGAATAAACAAAATATACAATACTTCTAAT
TTAACTGTTTTTGGTTGGGGTGACCAAGGGGAAAAACAAATCCCCCTCATCGATTGAGTACTAAGTACTTAAAAATTAGAATGA
CAATTCTAATTAATAAAACATTTATCGAAAAATGACCCAGGATTTCCTGATCAATGAACCAAGTTACCCTAGGGATAACAGCGCAA
TCCTTTCTCAGAGTCCCTATCGAAGAAAGGGTTTACGACCTCGATGTTGGATCAGGACATCCTAATGGTGCAACCGCTATTAAGG
GTTCGTTTGTTCAACGATTAACAGTCC

No.2025-166-27-6

CCGCCTGCCCTGTGACAACGTTCAACGGCCGCGGTATTTTGACCGTGCAAAGGTAGCGTAATCACTTGTCTTTTAAATGAAGAC
CTGTATGAAAGGCATCACGAGAGTTTAACTGTCTCTATTTTCTAATCAATGAAATTGATCTATTCGTGCAGAAGCGAATATAATAA
CATTAGACGAGAAGACCCTATGGAGCTTCAAACACTTAAATTAATTATGTAATCCTCCACCTCCCAGGGAATAAACAAAATATACA
ATACTTCTAATTTAACTGTTTTTGGTTGGGGTGACCAAGGGGAAAAACAAATCCCCCTCATCGATTGAGTACTAAGTACTTAAAA
ATTAGAATGACAATTCTAATTAATAAAACATTTATCGAAAAATGACCCAGGATTTCCTGATCAATGAACCAAGTTACCCTAGGGAT
AACAGCGCAATCCTTTCTCAGAGTCCCTATCGAAGAAAGGGTTTACGACCTCGATGTTGGATCAGGACATCCTAATGGTGCAAC
CGCTATTAAGGGT

No.2025-166-27-7

CCGCCTGCCCTGTGACAACGTTCAACGGCCGCGGTATTTTGACCGTGCAAAGGTAGCGTAATCACTTGTCTTTTAAATGAAGAC
CTGTATGAAAGGCATCACGAGAGTTTAACTGTCTCTATTTTCTAATCAATGAAATTGATCTATTCGTGCAGAAGCGAATATAATAA
CATTAGACGAGAAGACCCTATGGAGCTTCAAACACTTAAATTAATTATGTAATCCTCCACCTCCCAGGGAATAAACAAAATATACA
ATACTTCTAATTTAACTGTTTTTGGTTGGGGTGACCAAGGGGAAAAACAAATCCCCCTCATCGATTGAGTACTAAGTACTTAAAA
ATTAGAATGACAATTCTAATTAATAAAACATTTATCGAAAAATGACCCAGGATTTCCTGATCAATGAACCAAGTTACCCTAGGGAT
AACAGCGCAATCCTTTCTCAGAGTCCCTATCGAAGAAAGGGTTTACGACCTCGATGTTGGATCAGGACATCCTAATGGTGCAAC
CGCTATTAAGGGTTCGTT

No.2025-166-27-8

TGTGACAACGTTCAACGGCCGCGGTATTTTGACCGTGCAAAGGTAGCGTAATCACTTGTCTTTTAAATGAAGACCTGTATGAAA
GGCATCACGAGAGTTTAACTGTCTCTATTTTCTAATCAATGAAATTGATCTATTCGTGCAGAAGCGAATATAATAACATTAGACGA
GAAGACCCTATGGAGCTTCAAACACTTAAATTAATTATGTAATCCTCCACCTCCCAGGGAATAAACAAAATATACAATACTTCTAAT

TTAACTGTTTTTGGTTGGGGTGACCAAGGGGAAAAACAAATCCCCCTCATCGATTGAGTACTAAGTACTTAAAAATTAGAATGA
CAATTCTAATTAATAAAACATTTATCGAAAAATGACCCAGGATTTCCTGATCAATGAACCAAGTTACCCTAGGGATAACAGCGCAA
TCCTTTCTCAGAGTCCCTATCGAAGAAAGGGTTTACGACCTCGATGTTGGATCAGGACATCCTAATGGTGCAACCGCTATTAAGG
GTTCGTTTGTTCAACGATTAACAGTCCTACG

No.2025-166-27-9

CCGCCTGCCCTGTGACAATGTTTTAACGGCCGCGGTATTTTGACCGTGCAAAGGTAGCGTAATCACTTGTCTTTTAAATGAAGAC
CCGTATGAAAGGCATCACGAGAGTTCAACTGTCTCTATTTTCTAATCAATGAAATTGATCTACTCGTGCAGAAGCGAGTATAACTA
CATTAGACGAGAAGACCCTATGGAGCTTCAAACACATGAATTAAATATGTAAACTAACTACTCCCCGGACATAAATAAAATAATAT
TTTTAATTTAACTGTTTTTGGTTGGGGTGACCAAGGGGAAAAATAAATCCCCCTTATCGATTGAGTACTCAAGTACTTAAAAATC
AGAATTACAATTCTGATTAATAAAATATTTATCGAAAAATGACCCAGGATTTCCTGATCAATGAACCAAGTTACCCTAGGGATAAC
AGCGCAATCCTTTCTTAGAGTCCCTATCGCCGAAAGGGTTTACGACCTCGATGTTGGATCAGGACATCCTAATGATGCAACCGTT
ATTAAGGGTTCGTTTGTTCAAC

No.2025-166-27-10

CCGCCTGCCCTGTGACAATGTTTTAACGGCCGCGGTATTTTGACCGTGCAAAGGTAGCGTAATCACTTGTCTTTTAAATGAAGAC
CCGTATGAAAGGCATCACGAGAGTTTAACTGTCTCTATTTTCTAATCAATGAAATTGATCTACTCGTGCAGAAGCGAGTATAATCA
CATCAGACGAGAAGACCCTATGGAGCTTCAAACACATAAATTAACTACATAAATTAATTATTCCACGGATATAAATAAAAATACAG
TACCTTTAATTTAACTGTTTTTGGTTGGGGTGACCAAGGGGAAAAACAAATCCCCCTTATCGACTGAGTACTCAAGTACTTAAAA
ATTAGATTTACAATTCTAATTAATAAAATATTTATCGAACAATGACCCAGGATTTCCTGATCAATGAACCAAGTTACCCTAGGGATA
ACAGCGCAATCCTTTCTCAGAGTCCCTATCGCCGAAAGGGTTTACGACCTCGATGTTGGATCAGGACATCCTAATGATGCAACC
GTTATTAAGGGTTCGTT

No.2025-166-27-11

CCGCCTGCCCTGTGACAATGTTTTAACGGCCGCGGTATTTTGACCGTGCAAAGGTAGCGTAATCACTTGTCTTTTAAATGAAGAC
CCGTATGAAAGGCATCACGAGAGTTCAACTGTCTCTATTTTCTAATCAATGAAATTGATCTACTCGTGCAGAAGCGAGTATAACTA
CATTAGACGAGAAGACCCTATGGAGCTTCAAACACATGAATTAAATATGTAAACTAACTACTCCCCGGACATAAATAAAATAATAT
TTTTAATTTAACTGTTTTTGGTTGGGGTGACCAAGGGGAAAAATAAATCCCCCTTATCGATTGAGTACTCAAGTACTTAAAAATC
AGAATTACAATTCTGATTAATAAAATATTTATCGAAAAATGACCCAGGATTTCCTGATCAATGAACCAAGTTACCCTAGGGATAAC
AGCGCAATCCTTTCTTAGAGTCCCTATCGCCGAAAGGGTTTACGACCTCGATGTTGGATCAGGACATCCTAATGATGCAACCGTT
ATTAAGGGTTCGTT

No.2025-166-27-12

CCGCCTGCCCTGTGACAATGTTTTAACGGCCGCGGTATTTTGACCGTGCAAAGGTAGCGTAATCACTTGTCTTTTAAATGAAGAC
CCGTATGAAAGGCATCACGAGAGTTTAACTGTCTCTATTTTCTAATCAATGAAATTGATCTACTCGTGCAGAAGCGAGTATAATCA
CATCAGACGAGAAGACCCTATGGAGCTTCAAACACATAAATTAACTACATAAATTAATTATTCCACGGATATAAATAAAAATACAG
TACCTTTAATTTAACTGTTTTTGGTTGGGGTGACCAAGGGGAAAAACAAATCCCCCTTATCGACTGAGTACTCAAGTACTTAAAA
ATTAGATTTACAATTCTAATTAATAAAATATTTATCGAACAATGACCCAGGATTTCCTGATCAATGAACCAAGTTACCCTAGGGATA
ACAGCGCAATCCTTTCTCAGAGTCCCTATCGCCGAAAGGGTTTACGACCTCGATGTTGGATCAGGACATCCTAATGATGCAACC
GTTATTAAGGGTTCGTT

No.2025-166-27-13

CCCTGTGACAATGTTTTAACGGCCGCGGTATTTTGACCGTGCAAAGGTAGCGTAATCACTTGTCTTTTAAATGAAGACCCGTATG
AAAGGCATCACGAGAGTTTAACTGTCTCTATTTTCTAATCAATGAAATTGATCTACTCGTGCAGAAGCGAGTATAATCACATCAGA
CGAGAAGACCCTATGGAGCTTCAAACACATAAATTAACTACATAAATTAATTATTCCACGGATATAAATAAAAATACAGTACCTTTA
ATTTAACTGTTTTTGGTTGGGGTGACCAAGGGGAAAAACAAATCCCCCTTATCGACTGAGTACTCAAGTACTTAAAAATTAGATT
TACAATTCTAATTAATAAAATATTTATCGAACAATGACCCAGGATTTCCTGATCAATGAACCAAGTTACCCTAGGGATAACAGCGC
AATCCTTTCTCAGAGTCCCTATCGCCGAAAGGGTTTACGACCTCGATGTTGGATCAGGACATCCTAATGATGCAACCGTTATTAA
GGGTTCGTT

No.2025-166-27-14

CCCTGTGACAATGTTTTAACGGCCGCGGTATTTTGACCGTGCAAAGGTAGCGTAATCACTTGTCTTTTAAATGAAGACCCGTATG
AAAGGCATCACGAGAGTTTAACTGTCTCTATTTTCTAATCAATGAAATTGATCTACTCGTGCAGAAGCGAGTATAATCACATCAGA
CGAGAAGACCCTATGGAGCTTCAAACACATAAATTAACTACATAAATTAATTATTCCACGGATATAAATAAAAATACAGTACCTTTA
ATTTAACTGTTTTTGGTTGGGGTGACCAAGGGGAAAAACAAATCCCCCTTATCGACTGAGTACTCAAGTACTTAAAAATTAGATT
TACAATTCTAATTAATAAAATATTTATCGAACAATGACCCAGGATTTCCTGATCAATGAACCAAGTTACCCTAGGGATAACAGCGC
AATCCTTTCTCAGAGTCCCTATCGCCGAAAGGGTTTACGACCTCGATGTTGGATCAGGACATCCTAATGATGCAACCGTTATTAA
GGGTTC

No.2025-166-27-15

AGAGGTCCCGCCTGCCCTGTGACAACGTTCAACGGCCGCGGTATTTTGACCGTGCAAAGGTAGCGTAATCACTTGTCTTTTAAA
TGAAGACCTGTATGAAAGGCATCACGAGAGTTTAACTGTCTCTATTTTCTAATCAATGAAATTGATCTATTCGTGCAGAAGCGAAT
ATAATAACATTAGACGAGAAGACCCTATGGAGCTTCAAACACTTAAATTAATTATGTAATCCTCCACCTCCCAGGGAATAAACAAA
ATATACAATACTTCTAATTTAACTGTTTTTGGTTGGGGTGACCAAGGGGAAAAACAAATCCCCCTCATCGATTGAGTACTAAGTAC
TTAAAAATTAGAATGACAATTCTAATTAATAAAACATTTATCGAAAAATGACCCAGGATTTCCTGATCAATGAACCAAGTTACCCT
AGGGATAACAGCGCAATCCTTTCTCAGAGTCCCTATCGAAGAAAGGGTTTACGACCTCGATGTTGGATCAGGACATCCTAATGG
TGCAACCGCTATTAAGGGTTCGTT

No.2025-166-27-16

CCGCCTGCCCTGTGACAATGTTTTAACGGCCGCGGTATTTTGACCGTGCAAAGGTAGCGTAATCACTTGTCTTTTAAATGAAGAC
CCGTATGAAAGGCATCACGAGAGTTTAACTGTCTCTATTTTCTAATCAATGAAATTGATCTACTCGTGCAGAAGCGAGTATAATCA

CATCAGACGAGAAGACCCTATGGAGCTTCAAACACATAAATTAACTACATAAATTAATTATTCCACGGATATAAATAAAAATACAG
TACCTTTAATTTAACTGTTTTTGGTTGGGGTGACCAAGGGGAAAAACAAATCCCCCTTATCGACTGAGTACTCAAGTACTTAAAA
ATTAGATTTACAATTCTAATTAATAAAATATTTATCGAACAATGACCCAGGATTTCCTGATCAATGAACCAAGTTACCCTAGGGATA
ACAGCGCAATCCTTTCTCAGAGTCCCTATCGCCGAAAGGGTTTACGACCTCGATGTTGGATCAGGACATCCTAATGATGCAACC
GTTATTAAGGGTTCGTT

No.2025-166-27-17

CCGCCTGCCCTGTGACAATGTTTTAACGGCCGCGGTATTTTGACCGTGCAAAGGTAGCGTAATCACTTGTCTTTTAAATGAAGAC
CCGTATGAAAGGCATCACGAGAGTTCAACTGTCTCTATTTTCTAATCAATGAAATTGATCTACTCGTGCAGAAGCGAGTATAACTA
CATTAGACGAGAAGACCCTATGGAGCTTCAAACACATGAATTAAATATGTAAACTAACTACTCCCCGGACATAAATAAAATAATAT
TTTTAATTTAACTGTTTTTGGTTGGGGTGACCAAGGGGAAAAATAAATCCCCCTTATCGATTGAGTACTCAAGTACTTAAAAATC
AGAATTACAATTCTGATTAATAAAATATTTATCGAAAAATGACCCAGGATTTCCTGATCAATGAACCAAGTTACCCTAGGGATAAC
AGCGCAATCCTTTCTTAGAGTCCCTATCGCCGAAAGGGTTTACGACCTCGATGTTGGATCAGGACATCCTAATGATGCAACCGTT
ATTAAGGGTTCGTT

No.2025-166-27-18

AGAGGTCCCGCCTGCCCTGTGACAACGTTCAACGGCCGCGGTATTTTGACCGTGCAAAGGTAGCGTAATCACTTGTCTTTTAAA
TGAAGACCTGTATGAAAGGCATCACGAGAGTTTAACTGTCTCTATTTTCTAATCAATGAAATTGATCTATTCGTGCAGAAGCGAAT
ATAATAACATTAGACGAGAAGACCCTATGGAGCTTCAAACACTTAAATTAATTATGTAATCCTCCACCTCCCAGGGAATAAACAAA
ATATACAATACTTCTAATTTAACTGTTTTTGGTTGGGGTGACCAAGGGGAAAAACAAATCCCCCTCATCGATTGAGTACTAAGTAC
TTAAAAATTAGAATGACAATTCTAATTAATAAAACATTTATCGAAAAATGACCCAGGATTTCCTGATCAATGAACCAAGTTACCCT
AGGGATAACAGCGCAATCCTTTCTCAGAGTCCCTATCGAAGAAAGGGTTTACGACCTCGATGTTGGATCAGGACATCCTAATGG
TGCAACCGCTATTAAGGGTTCGTT

No.2025-166-27-19

AGAGGTCCCGCCTGCCCTGTGACAATGTTTTAACGGCCGCGGTATTTTGACCGTGCAAAGGTAGCGTAATCACTTGTCTTTTAAA
TGAAGACCCGTATGAAAGGCATCACGAGAGTTTAACTGTCTCTATTTTCTAATCAATGAAATTGATCTACTCGTGCAGAAGCGAG
TATAATCACATCAGACGAGAAGACCCTATGGAGCTTCAAACACATAAATTAACTACATAAATTAATTATTCCACGGATATAAATAAA
AATATAGTACCTTTAATTTAACTGTTTTTGGTTGGGGTGACCAAGGGGAAAAACAAATCCCCCTTATCGACTGAGTACTCAAGTA
CTTAAAAATTAGATTTACAATTCTAATTAATAAAATATTTATCGAACAATGACCCAGGATTTCCTGATCAATGAACCAAGTTACCCT
AGGGATAACAGCGCAATCCTTTCTCAGAGTCCCTATCGCCGAAAGGGTTTACGACCTCGATGTTGGATCAGGACATCCTAATGAT
GCAACCGTTATTAAGGGTTCGTTTGTTCAACGATTA

No.2025-166-27-20

CCGCCTGCCCTGTGACAATGTTTTAACGGCCGCGGTATTTTGACCGTGCAAAGGTAGCGTAATCACTTGTCTTTTAAATGAAGAC
CCGTATGAAAGGCATCACGAGAGTTTAACTGTCTCTATTTTCTAATCAATGAAATTGATCTACTCGTGCAGAAGCGAGTATAATCA
CATCAGACGAGAAGACCCTATGGAGCTTCAAACACATAAATTAACTACATAAATTAATTATTCCACGGATATAAATAAAAATACAG
TACCTTTAATTTAACTGTTTTTGGTTGGGGTGACCAAGGGGAAAAACAAATCCCCCTTATCGACTGAGTACTCAAGTACTTAAAA
ATTAGATTTACAATTCTAATTAATAAAATATTTATCGAACAATGACCCAGGATTTCCTGATCAATGAACCAAGTTACCCTAGGGATA
ACAGCGCAATCCTTTCTCAGAGTCCCTATCGCCGAAAGGGTTTACGACCTCGATGTTGGATCAGGACATCCTAATGATGCAACC
GTTATTAAGGGTTCGTT

No.2025-166-27-21

CCGCCTGCCCTGTGACAATGTTTTAACGGCCGCGGTATTTTGACCGTGCAAAGGTAGCGTAATCACTTGTCTTTTAAATGAAGAC
CCGTATGAAAGGCATCACGAGAGTTCAACTGTCTCTATTTTCTAATCAATGAAATTGATCTACTCGTGCAGAAGCGAGTATAACTA
CATTAGACGAGAAGACCCTATGGAGCTTCAAACACATGAATTAAATATGTAAACTAACTACTCCCCGGACATAAATAAAATAATAT
TTTTAATTTAACTGTTTTTGGTTGGGGTGACCAAGGGGAAAAATAAATCCCCCTTATCGATTGAGTACTCAAGTACTTAAAAATC
AGAATTACAATTCTGATTAATAAAATATTTATCGAAAAATGACCCAGGATTTCCTGATCAATGAACCAAGTTACCCTAGGGATAAC
AGCGCAATCCTTTCTTAGAGTCCCTATCGCCGAAAGGGTTTACGACCTCGATGTTGGATCAGGACATCCTAATGATGCAACCGTT
ATTAAGGGTTCGTT

No.2025-166-27-22

AGAGGTCCCGCCTGCCCTGTGACAATGTTTTAACGGCCGCGGTATTTTGACCGTGCAAAGGTAGCGTAATCACTTGTCTTTTAAA
TGAAGACCCGTATGAAAGGCATCACGAGAGTTTAACTGTCTCTATTTTCTAATCAATGAAATTGATCTACTCGTGCAGAAGCGAG
TATAATCACATCAGACGAGAAGACCCTATGGAGCTTCAAACACATAAATTAACTACATAAATTAATTATTCCACGGATATAAATAAA
AATACAGTACCTTTAATTTAACTGTTTTTGGTTGGGGTGACCAAGGGGAAAAACAAATCCCCCTTATCGACTGAGTACTCAAGTA
CTTAAAAATTAGATTTACAATTCTAATTAATAAAATATTTATCGAACAATGACCCAGGATTTCCTGATCAATGAACCAAGTTACCCT
AGGGATAACAGCGCAATCCTTTCTCAGAGTCCCTATCGCCGAAAGGGTTTACGACCTCGATGTTGGATCAGGACATCCTAATGAT
GCAACCGTTATTAAGGGTTCGTT

No.2025-166-27-23

CCGCCTGCCCTGTGACAATGTTTTAACGGCCGCGGTATTTTGACCGTGCAAAGGTAGCGTAATCACTTGTCTTTTAAATGAAGAC
CCGTATGAAAGGCATCACGAGAGTTTAACTGTCTCTATTTTCTAATCAATGAAATTGATCTACTCGTGCAGAAGCGAGTATAATCA
CATCAGACGAGAAGACCCTATGGAGCTTCAAACACATAAATTAACTACATAAATTAATTATTCCACGGATATAAATAAAAATACAG
TACCTTTAATTTAACTGTTTTTGGTTGGGGTGACCAAGGGGAAAAACAAATCCCCCTTATCGACTGAGTACTCAAGTACTTAAAA
ATTAGATTTACAATTCTAATTAATAAAATATTTATCGAACAATGACCCAGGATTTCCTGATCAATGAACCAAGTTACCCTAGGGATA
ACAGCGCAATCCTTTCTCAGAGTCCCTATCGCCGAAAGGGTTTACGACCTCGATGTTGGATCAGGACATCCTAATGATGCAACC
GTTATTAAGGGTTCGTT

No.2025-166-27-24

AGAGGTCCCGCCTGCCCTGTGACAATGTTTTAACGGCCGCGGTATTTTGACCGTGCAAAGGTAGCGTAATCACTTGTCTTTTAAA

TGAAGACCCGTATGAAAGGCATCACGAGAGTTCAACTGTCTCTATTTTCTAATCAATGAAATTGATCTACTCGTGCAGAAGCGAG
TATAACTACATTAGACGAGAAGACCCTATGGAGCTTCAAACACATGAATTAAATATGTAAACTAACTACTCCCCGGACATAAATAA
AATAATATTTTTAATTTAACTGTTTTTGGTTGGGGTGACCAAGGGGAAAAATAAATCCCCCTTATCGATTGAGTACTCAAGTACTT
AAAAATCAGAATTACAATTCTGATTAATAAAATATTTATCGAAAAATGACCCAGGATTTCCTGATCAATGAACCAAGTTACCCTAG
GGATAACAGCGCAATCCTTTCTTAGAGTCCCTATCGCCGAAAGGGTTTACGACCTCGATGTTGGATCAGGACATCCTAATGATGC
AACCGTTATTAAGGGTTCGTT

No.2025-166-27-25

CCGCCTGCCCTGTGACAACGTTCAACGGCCGCGGTATTTTGACCGTGCAAAGGTAGCGTAATCACTTGTCTTTTAAATGAAGAC
CTGTATGAAAGGCATCACGAGAGTTTAACTGTCTCTATTTTCTAATCAATGAAATTGATCTATTCGTGCAGAAGCGAATATAATAA
CATTAGACGAGAAGACCCTATGGAGCTTCAAACACTTAAATTAATTATGTAATCCTCCACCTCCCAGGGAATAAACAAAATATACA
ATACTTCTAATTTAACTGTTTTTGGTTGGGGTGACCAAGGGGAAAAACAAATCCCCCTCATCGATTGAGTACTAAGTACTTAAAA
ATTAGAATGACAATTCTAATTAATAAAACATTTATCGAAAAATGACCCAGGATTTCCTGATCAATGAACCAAGTTACCCTAGGGAT
AACAGCGCAATCCTTTCTCAGAGTCCCTATCGAAGAAAGGGTTTACGACCTCGATGTTGGATCAGGACATCCTAATGGTGCAAC
CGCTATTAAGGGTTCGTT

No.2025-166-27-26

CCGCCTGCCCTGTGACAACGTTCAACGGCCGCGGTATTTTGACCGTGCAAAGGTAGCGTAATCACTTGTCTTTTAAATGAAGAC
CTGTATGAAAGGCATCACGAGAGTTTAACTGTCTCTATTTTCTAATCAATGAAATTGATCTATTCGTGCAGAAGCGAATATAATAA
CATTAGACGAGAAGACCCTATGGAGCTTCAAACACTTAAATTAATTATGTAATCCTCCACCTCCCAGGGAATAAACAAAATATACA
ATACTTCTAATTTAACTGTTTTTGGTTGGGGTGACCAAGGGGAAAAACAAATCCCCCTCATCGATTGAGTACTAAGTACTTAAAA
ATTAGAATGACAATTCTAATTAATAAAACATTTATCGAAAAATGACCCAGGATTTCCTGATCAATGAACCAAGTTACCCTAGGGAT
AACAGCGCAATCCTTTCTCAGAGTCCCTATCGAAGAAAGGGTTTACGACCTCGATGTTGGATCAGGACATCCTAATGGTGCAAC
CGCTATTAAGGGT

No.2025-166-27-27

AGAGGTCCCGCCTGCCCTGTGACAATGTTTTAACGGCCGCGGTATTTTGACCGTGCAAAGGTAGCGTAATCACTTGTCTTTTAAA
TGAAGACCCGTATGAAAGGCATCACGAGAGTTCAACTGTCTCTATTTTCTAATCAATGAAATTGATCTACTCGTGCAGAAGCGAG
TATAACTACATTAGACGAGAAGACCCTATGGAGCTTCAAACACATGAATTAAATATGTAAACTAACTACTCCCCGGACATAAATAA
AATAATATTTTTAATTTAACTGTTTTTGGTTGGGGTGACCAAGGGGAAAAATAAATCCCCCTTATCGATTGAGTACTCAAGTACTT
AAAAATCAGAATTACAATTCTGATTAATAAAATATTTATCGAAAAATGACCCAGGATTTCCTGATCAATGAACCAAGTTACCCTAG
GGATAACAGCGCAATCCTTTCTTAGAGTCCCTATCGCCGAAAGGGTTTACGACCTCGATGTTGGATCAGGACATCCTAATGATGC
AACCGTTATTAAGGGTTCGTT

No.2025-166-27-28

CCGCCTGCCCTGTGACAATGTTTTAACGGCCGCGGTATTTTGACCGTGCAAAGGTAGCGTAATCACTTGTCTTTTAAATGAAGAC
CCGTATGAAAGGCATCACGAGAGTTCAACTGTCTCTATTTTCTAATCAATGAAATTGATCTACTCGTGCAGAAGCGAGTATAACTA
CATTAGACGAGAAGACCCTATGGAGCTTCAAACACATGAATTAAATATGTAAACTAACTACTCCCCGGACATAAATAAAATAATAT
TTTTGATTTAACTGTTTTTGGTTGGGGTGACCAAGGGGAAAAATAAATCCCCCTTATCGATTGAGTACTCAAGTACTTAAAAATC
AGAATTACAATTCTGATTAATAAAATATTTATCGAAAAATGACCCAGGATTTCCTGATCAATGAACCAAGTTACCCTAGGGATAAC
AGCGCAATCCTTTCTTAGAGTCCCTATCGCCGAAAGGGTTTACGACCTCGATGTTGGATCAGGACATCCTAATGATGCAACCGTT
ATTAAGGGTTCGTT

No.2025-166-27-29

CCGCCTGCCCTGTGACAATGTTTTAACGGCCGCGGTATTTTGACCGTGCAAAGGTAGCGTAATCACTTGTCTTTTAAATGAAGAC
CCGTATGAAAGGCATCACGAGAGTTTAACTGTCTCTATTTTCTAATCAATGAAATTGATCTACTCGTGCAGAAGCGAGTATAATCA
CATCAGACGAGAAGACCCTATGGAGCTTCAAACACATAAATTAACTACATAAATTAATTATTCCACGGATATAAATAAAAATACAG
TACCTTTAATTTAACTGTTTTTGGTTGGGGTGACCAAGGGGAAAAACAAATCCCCCTTATCGACTGAGTACTCAAGTACTTAAAA
ATTAGATTTACAATTCTAATTAATAAAATATTTATCGAACAATGACCCAGGATTTCCTGATCAATGAACCAAGTTACCCTAGGGATA
ACAGCGCAATCCTTTCTCAGAGTCCCTATCGCCGAAAGGGTTTACGACCTCGATGTTGGATCAGGACATCCTAATGATGCAACC
GTTATTAAGGGT

No.2025-166-27-30

CCGCCTGCCCTGTGACAACGTTCAACGGCCGCGGTATTTTGACCGTGCAAAGGTAGCGTAATCACTTGTCTTTTAAATGAAGAC
CTGTATGAAAGGCATCACGAGAGTTTAACTGTCTCTATTTTCTAATCAATGAAATTGATCTATTCGTGCAGAAGCGAATATAATAA
CATTAGACGAGAAGACCCTATGGAGCTTCAAACACTTAAATTAATTATGTAATCCTCCACCTCCCAGGGAATAAACAAAATATACA
ATACTTCTAATTTAACTGTTTTTGGTTGGGGTGACCAAGGGGAAAAACAAATCCCCCTCATCGATTGAGTACTAAGTACTTAAAA
ATTAGAATGACAATTCTAATTAATAAAACATTTATCGAAAAATGACCCAGGATTTCCTGATCAATGAACCAAGTTACCCTAGGGAT
AACAGCGCAATCCTTTCTCAGAGTCCCTATCGAAGAAAGGGTTTACGACCTCGATGTTGGATCAGGACATCCTAATGGTGCAAC
CGCTATTAAGGGTTCGTT

No.2025-166-27-31

CCGCCTGCCCTGTGACAACGTTCAACGGCCGCGGTATTTTGACCGTGCAAAGGTAGCGTAATCACTTGTCTTTTAAATGAAGAC
CTGTATGAAAGGCATCACGAGAGTTTAACTGTCTCTATTTTCTAATCAATGAAATTGATCTATTCGTGCAGAAGCGAATATAATAA
CATTAGACGAGAAGACCCTATGGAGCTTCAAACACTTAAATTAATTATGTAATCCTCCACCTCCCAGGGAATAAACAAAATATACA
ATACTTCTAATTTAACTGTTTTTGGTTGGGGTGACCAAGGGGAAAAACAAATCCCCCTCATCGATTGAGTACTAAGTACTTAAAA
ATTAGAATGACAATTCTAATTAATAAAACATTTATCGAAAAATGACCCAGGATTTCCTGATCAATGAACCAAGTTACCCTAGGGAT
AACAGCGCAATCCTTTCTCAGAGTCCCTATCGAAGAAAGGGTTTACGACCTCGATGTTGGATCAGGACATCCTAATGGTGCAAC
CGCTATTAAGGGT

No.2025-166-27-32

CCGCCTGCCCTGTGACAACGTTCAACGGCCGCGGTATTTTGACCGTGCAAAGGTAGCGTAATCACTTGTCTTTTAAATGAAGAC
CTGTATGAAAGGCATCACGAGAGTTTAACTGTCTCTATTTTCTAATCAATGAAATTGATCTATTCGTGCAGAAGCGAATATAATAA
CATTAGACGAGAAGACCCTATGGAGCTTCAAACACTTAAATTAATTATGTAATCCTCCACCTCCCAGGGAATAAACAAAATATACA
ATACTTCTAATTTAACTGTTTTTGGTTGGGGTGACCAAGGGGAAAAACAAATCCCCCTCATCGATTGAGTACTAAGTACTTAAAA
ATTAGAATGACAATTCTAATTAATAAAACATTTATCGAAAAATGACCCAGGATTTCCTGATCAATGAACCAAGTTACCCTAGGGAT
AACAGCGCAATCCTTTCTCAGAGTCCCTATCGAAGAAAGGGTTTACGACCTCGATGTTGGATCAGGACATCCTAATGGTGCAAC
CGCTATTAAGGGTTCGTT

No.2025-166-27-33

CATAAGAGGTCCCGCCTGCCCTGTGACAACGTTCAACGGCCGCGGTATTTTGACCGTGCAAAGGTAGCGTAATCACTTGTCTTT
TAAATGAAGACCTGTATGAAAGGCATCACGAGAGTTTAACTGTCTCTATTTTCTAATCAATGAAATTGATCTATTCGTGCAGAAGC
GAATATAATAACATTAGACGAGAAGACCCTATGGAGCTTCAAACACTTAAATTAATTATGTAATCCTCCACCTCCCAGGGAATAAA
CAAAATATACAATACTTCTAATTTAACTGTTTTTGGTTGGGGTGACCAAGGGGAAAAACAAATCCCCCTCATCGATTGAGTACTA
AGTACTTAAAAATTAGAATGACAATTCTAATTAATAAAACATTTATCGAAAAATGACCCAGGATTTCCTGATCAATGAACCAAGTT
ACCCTAGGGATAACAGCGCAATCCTTTCTCAGAGTCCCTATCGAAGAAAGGGTTTACGACCTCGATGTTGGATCAGGACATCCT
AATGGTGCAACCGCTATTAAGGGTTCGTT

No.2025-166-27-34

CCGCCTGCCCTGTGACAATGTTTTAACGGCCGCGGTATTTTGACCGTGCAAAGGTAGCGTAATCACTTGTCTTTTAAATGAAGAC
CCGTATGAAAGGCATCACGAGAGTTTAACTGTCTCTATTTTCTAATCAATGAAATTGATCTACTCGTGCAGAAGCGAGTATAATCA
CATCAGACGAGAAGACCCTATGGAGCTTCAAACACATAAATTAACTACATAAATTAATTATTCCACGGATATAAATAAAAATACAG
TACCTTTAATTTAACTGTTTTTGGTTGGGGTGACCAAGGGGAAAAACAAATCCCCCTTATCGACTGAGTACTCAAGTACTTAAAA
ATTAGATTTACAATTCTAATTAATAAAATATTTATCGAACAATGACCCAGGATTTCCTGATCAATGAACCAAGTTACCCTAGGGATA
ACAGCGCAATCCTTTCTCAGAGTCCCTATCGCCGAAAGGGTTTACGACCTCGATGTTGGATCAGGACATCCTAATGATGCAACC
GTTATTAAGGGTTCGTT

No.2025-166-27-35

CCGCCTGCCCTGTGACAACGTTCAACGGCCGCGGTATTTTGACCGTGCAAAGGTAGCGTAATCACTTGTCTTTTAAATGAAGAC
CTGTATGAAAGGCATCACGAGAGTTTAACTGTCTCTATTTTCTAATCAATGAAATTGATCTATTCGTGCAGAAGCGAATATAATAA
CATTAGACGAGAAGACCCTATGGAGCTTCAAACACTTAAATTAATTATGTAATCCTCCACCTCCCAGGGAATAAACAAAATATACA
ATACTTCTAATTTAACTGTTTTTGGTTGGGGTGACCAAGGGGAAAAACAAATCCCCCTCATCGATTGAGTACTAAGTACTTAAAA
ATTAGAATGACAATTCTAATTAATAAAACATTTATCGAAAAATGACCCAGGATTTCCTGATCAATGAACCAAGTTACCCTAGGGAT
AACAGCGCAATCCTTTCTCAGAGTCCCTATCGAAGAAAGGGTTTACGACCTCGATGTTGGATCAGGACATCCTAATGGTGCAAC
CGCTATTAAGGGTTCGTT

No.2025-166-27-36

CCGCCTGCCCTGTGACAACGTTCAACGGCCGCGGTATTTTGACCGTGCAAAGGTAGCGTAATCACTTGTCTTTTAAATGAAGAC
CTGTATGAAAGGCATCACGAGAGTTTAACTGTCTCTATTTTCTAATCAATGAAATTGATCTATTCGTGCAGAAGCGAATATAATAA
CATTAGACGAGAAGACCCTATGGAGCTTCAAACACTTAAATTAATTATGTAATCCTCCACCTCCCAGGGAATAAACAAAATATACA
ATACTTCTAATTTAACTGTTTTTGGTTGGGGTGACCAAGGGGAAAAACAAATCCCCCTCATCGATTGAGTACTAAGTACTTAAAA
ATTAGAATGACAATTCTAATTAATAAAACATTTATCGAAAAATGACCCAGGATTTCCTGATCAATGAACCAAGTTACCCTAGGGAT
AACAGCGCAATCCTTTCTCAGAGTCCCTATCGAAGAAAGGGTTTACGACCTCGATGTTGGATCAGGACATCCTAATGGTGCAAC
CGCTATTAAGGGTTCGTT

No.2025-166-27-37

ATTATAAGAGGTCCCGCCTGCCCTGTGACAATGTTTTAACGGCCGCGGTATTTTGACCGTGCAAAGGTAGCGTAATCACTTGTCT
TTTAAATGAAGACCCGTATGAAAGGCATCACGAGAGTTCAACTGTCTCTATTTTCTAATCAATGAAATTGATCTACTCGTGCAGAA
GCGAGTATAACTACATTAGACGAGAAGACCCTATGGAGCTTCAAACACATGAATTAAATATGTAAACTAACTACTCCCCGGACAT
AAATAAAATAATATTTTTAATTTAACTGTTTTTGGTTGGGGTGACCAAGGGGAAAAATAAATCCCCCTTATCGATTGAGTACTCAA
GTACTTAAAAATCAGAATTACAATTCTGATTAATAAAATATTTATCGAAAAATGACCCAGGATTTCCTGATCAATGAACCAAGTTAC
CCTAGGGATAACAGCGCAATCCTTTCTTAGAGTCCCTATCGCCGAAAGGGTTTACGACCTCGATGTTGGATCAGGACATCCTAAT
GATGCAACCGTTATTAAGGGTTCGTT

No.2025-166-27-38

AGAGGTCCCGCCTGCCCTGTGACAATGTTTTAACGGCCGCGGTATTTTGACCGTGCAAAGGTAGCGTAATCACTTGTCTTTTAAA
TGAAGACCCGTATGAAAGGCATCACGAGAGTTTAACTGTCTCTATTTTCTAATCAATGAAATTGATCTACTCGTGCAGAAGCGAG
TATAATCACATCAGACGAGAAGACCCTATGGAGCTTCAAACACATAAATTAACTACATAAATTAATTATTCCACGGATATAAATAAA
AATACAGTACCTTTAATTTAACTGTTTTTGGTTGGGGTGACCAAGGGGAAAAACAAATCCCCCTTATCGACTGAGTACTCAAGTA
CTTAAAAATTAGATTTACAATTCTAATTAATAAAATATTTATCGAACAATGACCCAGGATTTCCTGATCAATGAACCAAGTTACCCT
AGGGATAACAGCGCAATCCTTTCTCAGAGTCCCTATCGCCGAAAGGGTTTACGACCTCGATGTTGGATCAGGACATCCTAATGAT
GCAACCGTTATTAAGGGTTCGTT

No.2025-166-27-39

AGAGGTCCCGCCTGCCCTGTGACAATGTTTTAACGGCCGCGGTATTTTGACCGTGCAAAGGTAGCGTAATCACTTGTCTTTTAAA
TGAAGACCCGTATGAAAGGCATCACGAGAGTTCAACTGTCTCTATTTTCTAATCAATGAAATTGATCTACTCGTGCAGAAGCGAG
TATAACTACATTAGACGAGAAGACCCTATGGAGCTTCAAACACATGAATTAAATATGTAAACTAACTACTCCCCGGACATAAATAA
AATAATATTTTTAATTTAACTGTTTTTGGTTGGGGTGACCAAGGGGAAAAATAAATCCCCCTTATCGATTGAGTACTCAAGTACTT
AAAAATCAGAATTACAATTCTGATTAATAAAATATTTATCGAAAAATGACCCAGGATTTCCTGATCAATGAACCAAGTTACCCTAG
GGATAACAGCGCAATCCTTTCTTAGAGTCCCTATCGCCGAAAGGGTTTACGACCTCGATGTTGGATCAGGACATCCTAATGATGC
AACCGTTATTAAGGGTTCGTT

No.2025-166-27-40

CATAAGAGGTCCCGCCTGCCCTGTGACAATGTTTAACGGCCGCGGTATTCTGACCGTGCAAAGGTAGCGTAATCACTTGTCTTTT
AAATGAAGACCCGTATGAAAGGCATCACGAGAGTTCAACTGTCTCTACTTTCCAATCAATGAAATTGATCCACCCGTGCAGAAG
CGGGTATAAACACATCAGACGAGAAGACCCTATGGAGCTTCAAACACATGAATTAATTATGTAGACTAACTGCTCCACGGACATA
AATAAAAAATACAACACTTTTAATTTAACTGTTTTGGTTGGGGTGACCGAGGGGAAAAATCAATCCCCCCTATCGACCGAGTGTTC
TCAAGCACTTAAAAATTAGAATTACAATTCTAATTAGTAAAATATTTACCGAAAAATGACCCAGAATTTTCTGATCAATGAACCAA
GTTACCCTAGGGATAACAGCGCAATCCTTTCCTAGAGTCCCTATCGACGAAAGGGTTTACGACCTCGATGTTGGATCAGGACATC
CTAATGATGTAGCCGTTATTAAGGGT

No.2025-166-27-41

CGCCTGCCCTGTGACAATGTTTTAACGGCCGCGGTATTTTGACCGTGCAAAGGTAGCGTAATCACTTGTCTTTTAAATGAAGACC
CGTATGAAAGGCATCACGAGAGTTTAACTGTCTCTATTTTCTAATCAATGAAATTGATCTACTCGTGCAGAAGCGAGTATAATCAC
ATCAGACGAGAAGACCCTATGGAGCTTCAAACACATAAATTAACTACATAAATTAATTATTCCACGGATATAAATAAAAATACAGT
ACCTTTAATTTAACTGTTTTTGGTTGGGGTGACCAAGGGGAAAAACAAATCCCCCTTATCGACTGAGTACTCAAGTACTTAAAAA
TTAGATTTACAATTCTAATTAATAAAATATTTATCGAACAATGACCCAGGATTTCCTGATCAATGAACCAAGTTACCCTAGGGATAA
CAGCGCAATCCTTTCTCAGAGTCCCTATCGCCGAAAGGGTTTACGACCTCGATGTTGGATCAGGACATCCTAATGATGCAACCGT
TATTAAGGGT

No.2025-166-27-42

CCGCCTGCCCTGTGACAATGTTTAACGGCCGCGGTATTCTGACCGTGCAAAGGTAGCGTAATCACTTGTCTTTTAAATGAAGACC
CGTATGAAAGGCATCACGAGAGTTCAACTGTCTCTACTTTCCAATCAATGAAATTGATCCACCCGTGCAGAAGCGGGTATAAACA
CATCAGACGAGAAGACCCTATGGAGCTTCAAACACATGAATTAATTATGTAGACTAACTGCTCCACGGACATAAATAAAAATACA
ACACTTTTAATTTAACTGTTTTGGTTGGGGTGACCGAGGGGAAAAATCAATCCCCCTTATCGACCGAGTGTTCTCAAGCACTTAA
AAATTAGAATTACAATTCTAATTAGTAAAATATTTACCGAAAAATGACCCAGAATTTTCTGATCAATGAACCAAGTTACCCTAGGG
ATAACAGCGCAATCCTTTCCTAGAGTCCCTATCGACGAAAGGGTTTACGACCTCGATGTTGGATCAGGACATCCTAATGATGTAG
CCGTTATTAAGGGTTCGTT

No.2025-166-27-43

CCGCCTGCCCTGTGACAACGTTCAACGGCCGCGGTATTTTGACCGTGCAAAGGTAGCGTAATCACTTGTCTTTTAAATGAAGAC
CTGTATGAAAGGCATCACGAGAGTTTAACTGTCTCTATTTTCTAATCAATGAAATTGATCTATTCGTGCAGAAGCGAATATAATAA
CATTAGACGAGAAGACCCTATGGAGCTTCAAACACTTAAATTAATTATGTAATCCTCCACCTCCCAGGGAATAAACAAAATATACA
ATACTTCTAATTTAACTGTTTTTGGTTGGGGTGACCAAGGGGAAAAACAAATCCCCCTCATCGATTGAGTACTAAGTACTTAAAA
ATTAGAATGACAATTCTAATTAATAAAACATTTATCGAAAAATGACCCAGGATTTCCTGATCAATGAACCAAGTTACCCTAGGGAT
AACAGCGCAATCCTTTCTCAGAGTCCCTATCGAAGAAAGGGTTTACGACCTCGATGTTGGATCAGGACATCCTAATGGTGCAAC
CGCTATTAAGGGT

No.2025-166-27-44

CCGCCTGCCCTGTGACAACGTTCAACGGCCGCGGTATTTTGACCGTGCAAAGGTAGCGTAATCACTTGTCTTTTAAATGAAGAC
CTGTATGAAAGGCATCACGAGAGTTTAACTGTCTCTATTTTCTAATCAATGAAATTGATCTATTCGTGCAGAAGCGAATATAATAA
CATTAGACGAGAAGACCCTATGGAGCTTCAAACACTTAAATTAATTATGTAATCCTCCACCTCCCAGGGAATAAACAAAATATACA
ATACTTCTAATTTAACTGTTTTTGGTTGGGGTGACCAAGGGGAAAAACAAATCCCCCTCATCGATTGAGTACTAAGTACTTAAAA
ATTAGAATGACAATTCTAATTAATAAAACATTTATCGAAAAATGACCCAGGATTTCCTGATCAATGAACCAAGTTACCCTAGGGAT
AACAGCGCAATCCTTTCTCAGAGTCCCTATCGAAGAAAGGGTTTACGACCTCGATGTTGGATCAGGACATCCTAATGGTGCAAC
CGCTATTAAGGGT

No.2025-166-27-45

ACCATAAGAGGTCCCGCCTGCCCTGTGACAACGTTCAACGGCCGCGGTATTTTGACCGTGCAAAGGTAGCGTAATCACTTGTCT
TTTAAATGAAGACCTGTATGAAAGGCATCACGAGAGTTTAACTGTCTCTATTTTCTAATCAATGAAATTGATCTATTCGTGCAGAA
GCGAATATAATAACATTAGACGAGAAGACCCTATGGAGCTTCAAACACTTAAATTAATTATGTAATCCTCCACCTCCCAGGGAATA
AACAAAATATACAATACTTCTAATTTAACTGTTTTTGGTTGGGGTGACCAAGGGGAAAAACAAATCCCCCTCATCGATTGAGTAC
TAAGTACTTAAAAATTAGAATGACAATTCTAATTAATAAAACATTTATCGAAAAATGACCCAGGATTTCCTGATCAATGAACCAAG
TTACCCTAGGGATAACAGCGCAATCCTTTCTCAGAGTCCCTATCGAAGAAAGGGTTTACGACCTCGATGTTGGATCAGGACATC
CTAATGGTGCAACCGCTATTAAGGGTTCGTT

No.2025-166-27-46

CATAAGAGGTCCCGCCTGCCCTGTGACAACGTTCAACGGCCGCGGTATTTTGACCGTGCAAAGGTAGCGTAATCACTTGTCTTT
TAAATGAAGACCTGTATGAAAGGCATCACGAGAGTTTAACTGTCTCTATTTTCTAATCAATGAAATTGATCTATTCGTGCAGAAGC
GAATATAATAACATTAGACGAGAAGACCCTATGGAGCTTCAAACACTTAAATTAATTATGTAATCCTCCACCTCCCAGGGAATAAA
CAAAATATACAATACTTCTAATTTAACTGTTTTTGGTTGGGGTGACCAAGGGGAAAAACAAATCCCCCTCATCGATTGAGTACTA
AGTACTTAAAAATTAGAATGACAATTCTAATTAATAAAACATTTATCGAAAAATGACCCAGGATTTCCTGATCAATGAACCAAGTT
ACCCTAGGGATAACAGCGCAATCCTTTCTCAGAGTCCCTATCGAAGAAAGGGTTTACGACCTCGATGTTGGATCAGGACATCCT
AATGGTGCAACCGCTATTAAGGGT

No.2025-166-27-47

CCGCCTGCCCTGTGACAACGTTCAACGGCCGCGGTATTTTGACCGTGCAAAGGTAGCGTAATCACTTGTCTTTTAAATGAAGAC
CTGTATGAAAGGCATCACGAGAGTTTAACTGTCTCTATTTTCTAATCAATGAAATTGATCTATTCGTGCAGAAGCGAATATAATAA
CATTAGACGAGAAGACCCTATGGAGCTTCAAACACTTAAATTAATTATGTAATCCTCCACCTCCCAGGGAATAAACAAAATATACA
ATACTTCTAATTTAACTGTTTTTGGTTGGGGTGACCAAGGGGAAAAACAAATCCCCCTCATCGATTGAGTACTAAGTACTTAAAA
ATTAGAATGACAATTCTAATTAATAAAACATTTATCGAAAAATGACCCAGGATTTCCTGATCAATGAACCAAGTTACCCTAGGGAT
AACAGCGCAATCCTTTCTCAGAGTCCCTATCGAAGAAAGGGTTTACGACCTCGATGTTGGATCAGGACATCCTAATGGTGCAAC
CGCTATTAAGGGTTCGTT

No.2025-166-27-48

CATAAGAGGTCCCGCCTGCCCTGTGACAACGTTCAACGGCCGCGGTATTTTGACCGTGCAAAGGTAGCGTAATCACTTGTCTTT
TAAATGAAGACCTGTATGAAAGGCATCACGAGAGTTTAACTGTCTCTATTTTCTAATCAATGAAATTGATCTATTCGTGCAGAAGC
GAATATAATAACATTAGACGAGAAGACCCTATGGAGCTTCAAACACTTAAATTAATTATGTAATCCTCCACCTCCCAGGGAATAAA
CAAAATATACAATACTTCTAATTTAACTGTTTTTGGTTGGGGTGACCAAGGGGAAAAACAAATCCCCCTCATCGATTGAGTACTA
AGTACTTAAAAATTAGAATGACAATTCTAATTAATAAAACATTTATCGAAAAATGACCCAGGATTTCCTGATCAATGAACCAAGTT
ACCCTAGGGATAACAGCGCAATCCTTTCTCAGAGTCCCTATCGAAGAAAGGGTTTACGACCTCGATGTTGGATCAGGACATCCT
AATGGTGTAACCGCTATTAAGGGTTCGTT

No.2025-166-27-49

CCGCCTGCCCTGTGACAACGTTCAACGGCCGCGGTATTTTGACCGTGCAAAGGTAGCGTAATCACTTGTCTTTTAAATGAAGAC
CTGTATGAAAGGCATCACGAGAGTTTAACTGTCTCTATTTTCTAATCAATGAAATTGATCTATTCGTGCAGAAGCGAATATAATAA
CATTAGACGAGAAGACCCTATGGAGCTTCAAACACTTAAATTAATTATGTAATCCTCCACCTCCCAGGGAATAAACAAAATATACA
ATACTTCTAATTTAACTGTTTTTGGTTGGGGTGACCAAGGGGAAAAACAAATCCCCCTCATCGATTGAGTACTAAGTACTTAAAA
ATTAGAATGACAATTCTAATTAATAAAACATTTATCGAAAAATGACCCAGGATTTCCTGATCAATGAACCAAGTTACCCTAGGGAT
AACAGCGCAATCCTTTCTCAGAGTCCCTATCGAAGAAAGGGTTTACGACCTCGATGTTGGATCAGGACATCCTAATGGTGCAAC
CGCTATTAAGGGTTCGTT

No.2025-166-27-50

GTGACAACGTTCAACGGCCGCGGTATTTTGACCGTGCAAAGGTAGCGTAATCACTTGTCTTTTAAATGAAGACCTGTATGAAAG
GCATCACGAGAGTTTAACTGTCTCTATTTTCTAATCAATGAAATTGATCTATTCGTGCAGAAGCGAATATAATAACATTAGACGAG
AAGACCCTATGGAGCTTCAAACACTTAAATTAATTATGTAATCCTCCACCTCCCAAGGAATAAACAAAATATACAATACTTCTAATT
TAACTGTTTTTGGTTGGGGTGACCAAGGGGAAAAACAAATCCCCCTCATCGATTGAGTACTAAGTACTTAAAAATTAGAATGAC
AATTCTAATTAATAAAACATTTATCGAAAAATGACCCAGGATTTCCTGATCAATGAACCAAGTTACCCTAGGGATAACAGCGCAAT
CCTTTCTCAGAGTCCCTATCGAAGAAAGGGTTTACGACCTCGATGTTGGATCAGGACATCCTAATGGTGCAACCGCTATTAAGG
GTTCGTTTGTTCAACGATTAACAGTCCTACGT

No.2025-166-27-51

GTGACAACGTTCAACGGCCGCGGTATTTTGACCGTGCAAAGGTAGCGTAATCACTTGTCTTTTAAATGAAGACCTGTATGAAAG
GCATCACGAGAGTTTAACTGTCTCTATTTTCTAATCAATGAAATTGATCTATTCGTGCAGAAGCGAATATAATAACATTAGACGAG
AAGACCCTATGGAGCTTCAAACACTTAAATTAATTATGTAATCCTCCACCTCCCAGGGAATAAACAAAATATACAATACTTCTAATT
TAACTGTTTTTGGTTGGGGTGACCAAGGGGAAAAACAAATCCCCCTCATCGATTGAGTACTAAGTACTTAAAAATTAGAATGAC
AATTCTAATTAATAAAACATTTATCGAAAAATGACCCAGGATTTCCTGATCAATGAACCAAGTTACCCTAGGGATAACAGCGCAAT
CCTTTCTCAGAGTCCCTATCGAAGAAAGGGTTTACGACCTCGATGTTGGATCAGGACATCCTAATGGTGCAACCGCTATTAAGG
GTTCGTTTGTTCAACGATTAACAGTCCTACGTGA

No.2025-166-27-52

GTGACAATGTTTAACGGCCGCGGTATTCTGACCGTGCAAAGGTAGCGTAATCACTTGTCTTTTAAATGAAGACCCGTATGAAAG
GCATCACGAGAGTTCAACTGTCTCTACTTTCCAATCAATGAAATTGATCCACCCGTGCAGAAGCGGGTATAAACACATCAGACG
AGAAGACCCTATGGAGCTTCAAACACATGAATTAATTATGTAGACTAACTGCTCCACGGACATAAATAAAAATACAACACTTTTAA
TTTAACTGTTTTGGTTGGGGTGACCGAGGGGAAAAATCAATCCCCCTTATCGACCGAGTGTTCTCAAGCACTTAAAAATTAGAA
TTACAATTCTAATTAGTAAAATATTTACCGAAAAATGACCCAGAATTTTCTGATCAATGAACCAAGTTACCCTAGGGATAACAGCG
CAATCCTTTCCTAGAGTCCCTATCGACGAAAGGGTTTACGACCTCGATGTTGGATCAGGACATCCTAATGATGTAGCCGTTATTAA
GGGTTCGTTTGTTCAACGATTAACAGTCCTACGTGA

No.2025-166-27-53

CCGCCTGCCCTGTGACAATGTTTAACGGCCGCGGTATTCTGACCGTGCAAAGGTAGCGTAATCACTTGTCTTTTAAATGAAGACC
CGTATGAAAGGCATCACGAGAGTTCAACTGTCTCTACTTTCCAATCAATGAAATTGATCCACCCGTGCAGAAGCGGGTATAAACA
CATCAGACGAGAAGACCCTATGGAGCTTCAAACACATGAATTAATTATGTAGACTAACTACTCCACGGACATAAATAAAAATACA
ACACTTTTAATTTAACTGTTTTGGTTGGGGTGACCGAGGGGAAAAATCAATCCCCCTTATCGACCGAGTGTTCTCAAGCACTTAA
AAATTAGAATTACAATTCTAATTAGTAAAATATTTACCGAAAAATGACCCAGAATTTTCTGATCAATGAACCAAGTTACCCTAGGG
ATAACAGCGCAATCCTTTCCTAGAGTCCCTATCGACGAAAGGGTTTACGACCTCGATGTTGGATCAGGACATCCTAATGATGTAG
CCGTTATTAAGGGTTCGTT

No.2025-166-27-54

GGTCCCGCCTGCCCTGTGACAATGTTTAACGGCCGCGGTATTCTGACCGTGCAAAGGTAGCGTAATCACTTGTCTTTTAAATGAA
GACCCGTATGAAAGGCATCACGAGAGTTCAACTGTCTCTACTTTCCAATCAATGAAATTGATCCACCCGTGCAGAAGCGGGTATA
AACACATCAGACGAGAAGACCCTATGGAGCTTCAAACACATGAATTAATTATGTAGACTAACTACTCCACGGACATAAATAAAAA
TACAACACTTTTAATTTAACTGTTTTGGTTGGGGTGACCGAGGGGAAAAATCAATCCCCCTTATCGACCGAGTGTTCTCAAGCA
CTTAAAAATTAGAATTACAATTCTAATTAGTAAAATATTTACCGAAAAATGACCCAGAATTTTCTGATCAATGAACCAAGTTACCCT
AGGGATAACAGCGCAATCCTTTCCTAGAGTCCCTATCGACGAAAGGGTTTACGACCTCGATGTTGGATCAGGACATCCTAATGAT
GTAGCCGTTATTAAGGGTTCGTT

No.2025-166-27-55

CATAAGAGGTCCCGCCTGCCCTGTGACAATGTTTAACGGCCGCGGTATTCTGACCGTGCAAAGGTAGCGTAATCACTTGTCTTTT
AAATGAAGACCCGTATGAAAGGCATCACGAGAGTTCAACTGTCTCTACTTTCCAATCAATGAAATTGATCCACCCGTGCAGAAG
CGGGTATAAACACATCAGACGAGAAGACCCTATGGAGCTTCAAACACATGAATTAATTATGTAGACTAACTGCTCCACGGACATA
AATAAAAATACAACACTTTTAATTTAACTGTTTTGGTTGGGGTGACCGAGGGGAAAAATCAATCCCCCTTATCGACCGAGTGTTC
TCAAGCACTTAAAAATTAGAATTACAATTCTAATTAGTAAAATATTTACCGAAAAATGACCCAGAATTTTCTGATCAATGAACCAA
GTTACCCTAGGGATAACAGCGCAATCCTTTCCTAGAGTCCCTATCGACGAAAGGGTTTACGACCTCGATGTTGGATCAGGACATC
CTAATGATGTAGCCGTTATTAAGGGTTCGTT

No.2025-166-27-56

AGAGGTCCCGCCTGCCCTGTGACAATGTTTAACGGCCGCGGTATTCTGACCGTGCAAAGGTAGCGTAATCACTTGTCTTTTAAAT
GAAGACCCGTATGAAAGGCATCACGAGAGTTCAACTGTCTCTACTTTCCAATCAATGAAATTGATCCACCCGTGCAGAAGCGGG
TATAAACACATCAGACGAGAAGACCCTATGGAGCTTCAAACACATGAATTAATTATGTAGACTAACTGCTCCACGGACATAAATA
AAAATACAACACTTTTAATTTAACTGTTTTGGTTGGGGTGACCGAGGGGAAAAATCAATCCCCCTTATCGACCGAGTGTTCTCAA
GCACTTAAAAATTAGAATTACAATTCTAATTAGTAAAATATTTACCGAAAAATGACCCAGAATTTTCTGATCAATGAACCAAGTTA
CCCTAGGGATAACAGCGCAATCCTTTCCTAGAGTCCCTATCGACGAAAGGGTTTACGACCTCGATGTTGGATCAGGACATCCTAA
TGATGTAGCCGTTATTAAGGGTTCGTT

No.2025-166-27-57

CCGCCTGCCCTGTGACAACGTTCAACGGCCGCGGTATTTTGACCGTGCAAAGGTAGCGTAATCACTTGTCTTTTAAATGAAGAC
CTGTATGAAAGGCATCACGAGAGTTTAACTGTCTCTATTTTCTAATCAATGAAATTGATCTATTCGTGCAGAAGCGAATATAATAA
CATTAGACGAGAAGACCCTATGGAGCTTCAAACACTTAAATTAATTATGTAATCCTCCACCTCCCAGGGAATAAACAAAATATACA
ATACTTCTAATTTAACTGTTTTTGGTTGGGGTGACCAAGGGGAAAAACAAATCCCCCTCATCGATTGAGTACTAAGTACTTAAAA
ATTAGAATGACAATTCTAATTAATAAAACATTTATCGAAAAATGACCCAGGATTTCCTGATCAATGAACCAAGTTACCCTAGGGAT
AACAGCGCAATCCTTTCTCAGAGTCCCTATCGAAGAAAGGGTTTACGACCTCGATGTTGGATCAGGACATCCTAATGGTGCAAC
CGCTATTAAGGGTTCGTT

No.2025-166-27-58

CCGCCTGCCCTGTGACAACGTTCAACGGCCGCGGTATTTTGACCGTGCAAAGGTAGCGTAATCACTTGTCTTTTAAATGAAGAC
CTGTATGAAAGGCATCACGAGAGTTTAACTGTCTCTATTTTCTAATCAATGAAATTGATCTATTCGTGCAGAAGCGAATATAATAA
CATTAGACGAGAAGACCCTATGGAGCTTCAAACACTTAAATTAATTATGTAATCCTCCACCTCCCAGGGAATAAACAAAATATACA
ATACTTCTAATTTAACTGTTTTTGGTTGGGGTGACCAAGGGGAAAAACAAATCCCCCTCATCGATTGAGTACTAAGTACTTAAAA
ATTAGAATGACAATTCTAATTAATAAAACATTTATCGAAAAATGACCCAGGATTTCCTGATCAATGAACCAAGTTACCCTAGGGAT
AACAGCGCAATCCTTTCTCAGAGTCCCTATCGAAGAAAGGGTTTACGACCTCGATGTTGGATCAGGACATCCTAATGGTGCAAC
CGCTATTAAGGGTTCGTT

No.2025-166-27-59

GCCCTGTGACAACGTTCAACGGCCGCGGTATTTTGACCGTGCAAAGGTAGCGTAATCACTTGTCTTTTAAATGAAGACCTGTAT
GAAAGGCATCACGAGAGTTTAACTGTCTCTATTTTCTAATCAATGAAATTGATCTATTCGTGCAGAAGCGAATATAATAACATTAG
ACGAGAAGACCCTATGGAGCTTCAAACACTTAAATTAATTATGTAATCCTCCACCTCCCAGGGAATAAACAAAATATACAATACTT
CTAATTTAACTGTTTTTGGTTGGGGTGACCAAGGGGAAAAACAAATCCCCCTCATCGATTGAGTACTAAGTACTTAAAAATTAGA
ATGACAATTCTAATTAATAAAACATTTATCGAAAAATGACCCAGGATTTCCTGATCAATGAACCAAGTTACCCTAGGGATAACAGC
GCAATCCTTTCTCAGAGTCCCTATCGAAGAAAGGGTTTACGACCTCGATGTTGGATCAGGACATCCTAATGGTGCAACCGCTATT
AAGGGTTCGTTTGTTCAACGATTAACAGTCCTACGT

No.2025-166-27-60

GCCCTGTGACAACGTTCAACGGCCGCGGTATTTTGACCGTGCAAAGGTAGCGTAATCACTTGTCTTTTAAATGAAGACCTGTAT
GAAAGGCATCACGAGAGTTTAACTGTCTCTATTTTCTAATCAATGAAATTGATCTATTCGTGCAGAAGCGAATATAATAACATTAG
ACGAGAAGACCCTATGGAGCTTCAAACACTTAAATTAATTATGTAATCCTCCACCTCCCAGGGAATAAACAAAATATACAATACTT
CTAATTTAACTGTTTTTGGTTGGGGTGACCAAGGGGAAAAACAAATCCCCCTCATCGATTGAGTACTAAGTACTTAAAAATTAGA
ATGACAATTCTAATTAATAAAACATTTATCGAAAAATGACCCAGGATTTCCTGATCAATGAACCAAGTTACCCTAGGGATAACAGC
GCAATCCTTTCTCAGAGTCCCTATCGAAGAAAGGGTTTACGACCTCGATGTTGGATCAGGACATCCTAATGGTGCAACCGCTATT
AAGGGTTCGTTTGTTCAACGATTAACAGTCCTACGTGATCTG

No.2025-166-27-61

CCGCCTGCCCTGTGACAACGTTCAACGGCCGCGGTATTTTGACCGTGCAAAGGTAGCGTAATCACTTGTCTTTTAAATGAAGAC
CTGTATGAAAGGCATCACGAGAGTTTAACTGTCTCTATTTTCTAATCAATGAAATTGATCTATTCGTGCAGAAGCGAATATAATAA
CATTAGACGAGAAGACCCTATGGAGCTTCAAACACTTAAATTAATTATGTAATCCTCCACCTCCCAGGGAATAAACAAAATATACA
ATACTTCTAATTTAACTGTTTTTGGTTGGGGTGACCAAGGGGAAAAACAAATCCCCCTCATCGATTGAGTACTAAGTACTTAAAA
ATTAGAATGACAATTCTAATTAATAAAACATTTATCGAAAAATGACCCAGGATTTCCTGATCAATGAACCAAGTTACCCTAGGGAT
AACAGCGCAATCCTTTCTCAGAGTCCCTATCGAAGAAAGGGTTTACGACCTCGATGTTGGATCAGGACATCCTAATGGTGCAAC
CGCTATTAAGGGTTCGTT

No.2025-166-27-62

GCCGCGGTATTTTGACCGTGCAAAGGTAGCGTAATCACTTGTCTTTTAAATGAAGACCCGTATGAAAGGCATCACGAGAGTTTA
ACTGTCTCTATTTTCTAATCAATGAAATTGATCTACTCGTGCAGAAGCGAGTATAATCACATCAGACGAGAAGACCCTATGGAGCT
TCAAACACATAAATTAACTACATAAATTAATTATTCCACGGATATAAATAAAAATACAGTACCTTTAATTTAACTGTTTTTGGTTGGG
GTGACCAAGGGGAAAAACAAATCCCCCTTATCGACTGAGTACTCAAGTACTTAAAAATTAGATTTACAATTCTAATTAATAAAATA
TTTATCGAACAATGACCCAGGATTTCCTGATCAATGAACCAAGTTACCCTAGGGATAACAGCGCAATCCTTTCTCAGAGTCCCTAT
CGCCGAAAGGGTTTACGACCTCGATGTTGGATCAGGACATCCTAATGATGCAACCGTTATTAAGGGTTCGTTTGTTCAACGATTA
ATAGTCCTACGTG

No.2025-166-27-63

CCGCCTGCCCTGTGACAATGTTTTAACGGCCGCGGTATTTTGACCGTGCAAAGGTAGCGTAATCACTTGTCTTTTAAATGAAGAC
CCGTATGAAAGGCATCACGAGAGTTTAACTGTCTCTATTTTCTAATCAATGAAATTGATCTACTCGTGCAGAAGCGAGTATAATCA
CATCAGACGAGAAGACCCTATGGAGCTTCAAACACATAAATTAACTACATAAATTAATTATTCCACGGATATAAATAAAAATACAG
TACCTTTAATTTAACTGTTTTTGGTTGGGGTGACCAAGGGGAAAAACAAATCCCCCTTATCGACTGAGTACTCAAGTACTTAAAA
ATTAGATTTACAATTCTAATTAATAAAATATTTATCGAACAATGACCCAGGATTTCCTGATCAATGAACCAAGTTACCCTAGGGATA
ACAGCGCAATCCTTTCTCAGAGTCCCTATCGCCGAAAGGGTTTACGACCTCGATGTTGGATCAGGACATCCTAATGATGCAACC
GTTATTAAGGGTTCGTT

No.2025-166-27-64

CCGCCTGCCCTGTGACAATGTTTTAACGGCCGCGGTATTTTGACCGTGCAAAGGTAGCGTAATCACTTGTCTTTTAAATGAAGAC
CCGTATGAAAGGCATCACGAGAGTTCAACTGTCTCTATTTTCTAATCAATGAAATTGATCTACTCGTGCAGAAGCGAGTATAACTA
CATTAGACGAGAAGACCCTATGGAGCTTCAAACACATGAATTAAATATGTAAACTAACTACTCCCCGGACATAAATAAAATAATAT
TTTTAATTTAACTGTTTTTGGTTGGGGTGACCAAGGGGAAAAATAAATCCCCCTTATCGATTGAGTACTCAAGTACTTAAAAATC
AGAATTACAATTCTGATTAATAAAATATTTATCGAAAAATGACCCAGGATTTCCTGATCAATGAACCAAGTTACCCTAGGGATAAC
AGCGCAATCCTTTCTTAGAGTCCCTATCGCCGAAAGGGTTTACGACCTCGATGTTGGATCAGGACATCCTAATGATGCAACCGTT
ATTAAGGGTTCGTT
