# Supplementary figures and images for: Forensic Species Identification: A Case Involving Trafficked Fish Maws and Shark Fins
Source: Int J Mol Sci. 2026 Jun 27;27(13):5813. doi: 10.3390/ijms27135813 (PMC13361761; doi:10.3390/ijms27135813)

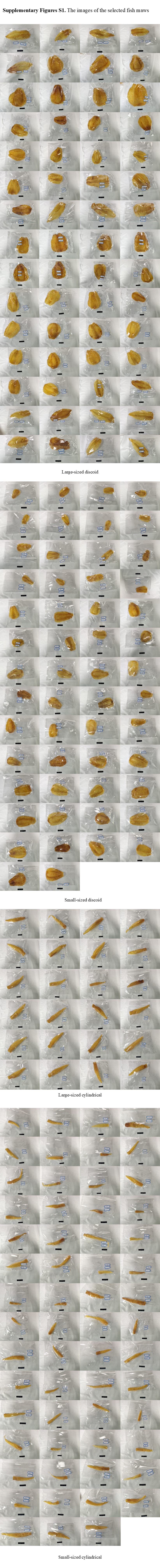

Supplement: Supplementary file 1 [file ijms-27-05813-s001.zip › Figure S1.tif]

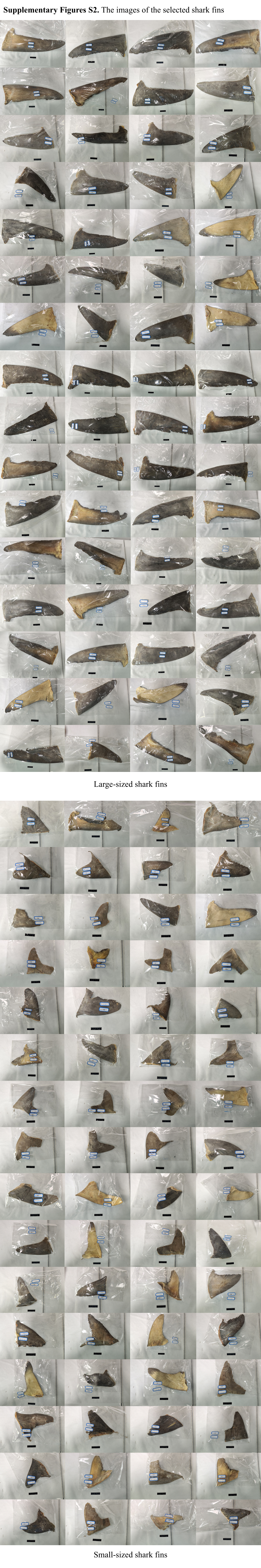

Supplement: Supplementary file 1 [file ijms-27-05813-s001.zip › Figure S2.tif]
